# Flavichalasines A–M, cytochalasan alkaloids from *Aspergillus flavipes*

**DOI:** 10.1038/srep42434

**Published:** 2017-02-13

**Authors:** Guangzheng Wei, Dongdong Tan, Chunmei Chen, Qingyi Tong, Xiao-Nian Li, Jinfeng Huang, Junjun Liu, Yongbo Xue, Jianping Wang, Zengwei Luo, Hucheng Zhu, Yonghui Zhang

**Affiliations:** 1 Hubei Key Laboratory of Natural Medicinal Chemistry and Resource Evaluation, School of Pharmacy, Tongji Medical College, Huazhong University of Science and Technology, Wuhan 430030, People’s Republic of China; 2 State Key Laboratory of Phytochemistry and Plant Resources in West China, Kunming Institute of Botany, Chinese Academy of Sciences, Kunming 650204, China

## Abstract

Two new tetracyclic cytochalasans, flavichalasines A and B (**1** and **2**), three new pentacyclic cytochalasans, flavichalasines C–E (**3**–**5**), and eight new tricyclic cytochalasans, flavichalasines F–M (**6**–**13**), together with eight known analogues (**14**–**21**), were isolated from the solid culture of *Aspergillus flavipes*. Structures of these new compounds were elucidated on the basis of extensive spectroscopic analyses including 1D, 2D NMR and HRESIMS data. Their absolute configurations were determined by comparison of their experimental ECD with either computed ECD or experimental ECD spectrum of known compound. The structure and absolute configuration of **2** were further determined by X-ray crystallographic diffraction. Flavichalasine A (**1**) represents the first example of cytochalasan with a terminal double bond at the macrocyclic ring and flavichalasine E (**5**) is the only cytochalasan with an *α*-oriented oxygen-bridge in D ring. These new compounds were evaluated for their cytotoxic activities against seven human cancer cell lines, of which, **6** and **14** displayed moderate inhibitory activities against tested cell lines. In addition, compounds **6** and **14** induced apoptosis of HL60 cells by activation of caspase-3 and degradation of PARP.

Cytochalasans are a group of mycotoxins well known for the wide ranges of biological activities such as cytotoxic, antimicrobial, antiviral, and phytotoxic activities^1^. It is estimated that more than 300 cytochalasan analogues have been isolated from genera of *Aspergillus*^2^,^3^,^4^,^5^, *Chaetomium*^6^,^7^,^8^,^9^, *Spicaria*^10^,^11^,^12^, *Phomopsis*^13^,^14^, and so on^1^. In general, cytochalasans are characterized by a highly substituted perhydro-isoindolone moiety to which typically a macrocyclic ring is fused. Isotope labeling experiments and biosynthesis studies have revealed that biosynthetic pathways of cytochalasans involve the formation of an acetyl- and methionine-derived polyketide chain and the attachment of an amino acid such as phenylalanine, leucine, or tryptophan^15^,^16^,^17^,^18^,^19^. Aspochalasins are a subgroup of cytochalasans with leucine as the original precursor. The structures and biological activities of aspochalasins have attracted great interest from synthetic and pharmacological communities^20^,^21^.

As part of our ongoing search for novel bioactive secondary metabolites from fungi, dozens of cytochalasans with distinctive cytotoxic or anti-HIV activities have been isolated from the arthropod-derived fungus *Chaetomium globosum*^22^,^23^. In order to find more structure intriguing and bioactive cytochalasans, secondary metabolites of the fungus *Aspergillus flavipes* have been systemically investigated. In the previous researches on *A. flavipes*, a series of cytochalasan dimmers were isolated, with asperchalasine A^24^ and epicochalasines A and B^25^ as their representatives, in addition to monomeric aspochalasin derivatives^5^. Further investigation on this fungus led to the isolation of thirteen new (**1**–**13**) and eight known (**14**–**21**) cytochalasans belonging to the aspochalasin group from the EtOH extract of *A. flavipes* (Fig. 1), including two new tetracyclic (**1** and **2**) and three new pentacyclic cytochalasans (**3**–**5**).

## Results and Discussion

### Structure Elucidation

Flavichalasine A (**1**) had a molecular formula of C_24_H_33_NO_5_, requiring nine degrees of unsaturation, as deduced from its HRESIMS ion peak at *m/z* 416.2433 ([M + H]^+^, calcd for C_24_H_34_NO_5_, 416.2437). The ^1^H NMR (Table 1) along with HSQC spectra showed resonances for an olefinic proton (*δ*_H_ 5.54, br s), two terminal double bond protons (*δ*_H_ 5.08, s and 4.79, s), two oxygenated methine protons (*δ*_H_ 5.19, d, *J* = 11.7 Hz and 4.51, dd, *J* = 7.2, 4.2 Hz), and four methyls (*δ*_H_ 1.76, s; 1.25, d, *J* = 7.3 Hz; 0.94, d, *J* = 6.4 Hz; and 0.92, d, *J* = 6.5 Hz). The ^13^C NMR (Table 2) and DEPT data of **1** displayed two carbonyls (*δ*_C_ 211.4 and 205.3), an amide carbonyl (*δ*_C_ 175.0), two double bonds (*δ*_C_ 149.6, 141.5, 124.9, and 115.7), a quaternary carbon (*δ*_C_ 67.9), nine methines including two oxygenated ones (*δ*_C_ 75.6 and 74.5), three methylenes, and four methyl groups. Besides five degrees of unsaturation occupied by carbonyls and double bonds, the remaining four suggested **1** to be a cytochalasan possessing a tetracyclic ring system.

**Figure 2:**
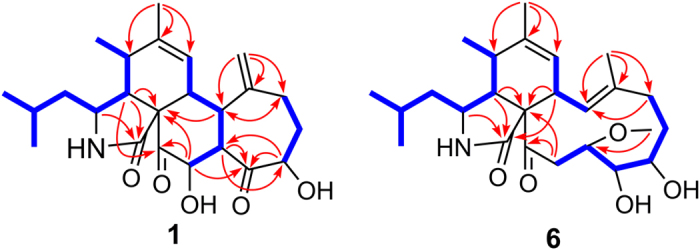
^1^H–^1^H COSY and key HMBC correlations of 1 and 6.

**Figure 3:**
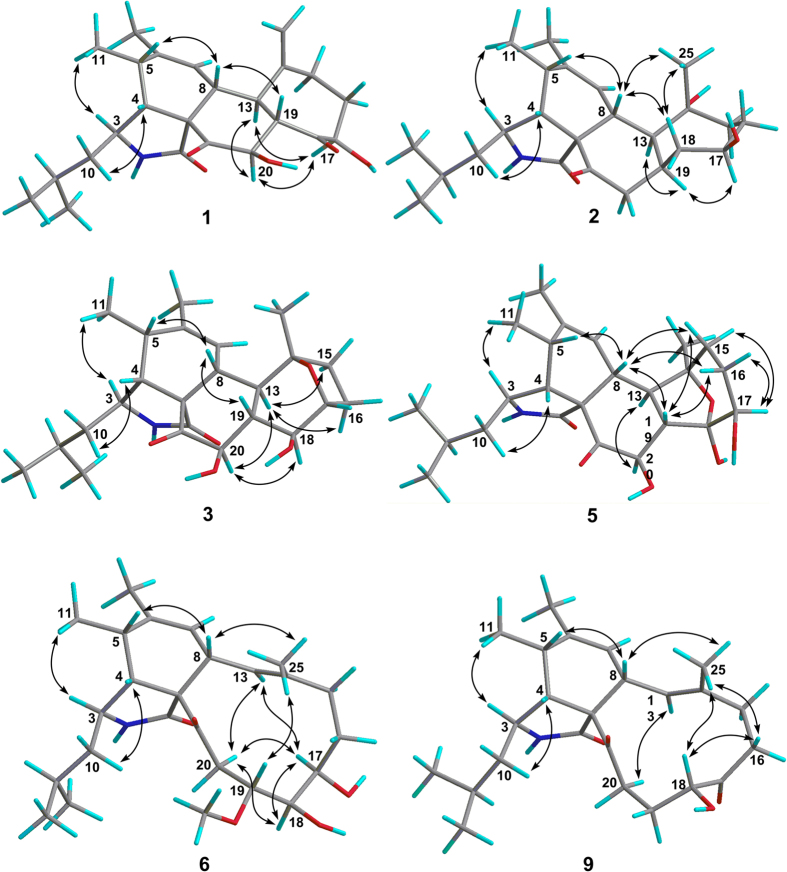
Key NOESY correlations of compounds 1, 2, 3, 5, 6, and 9.

**Figure 4:**
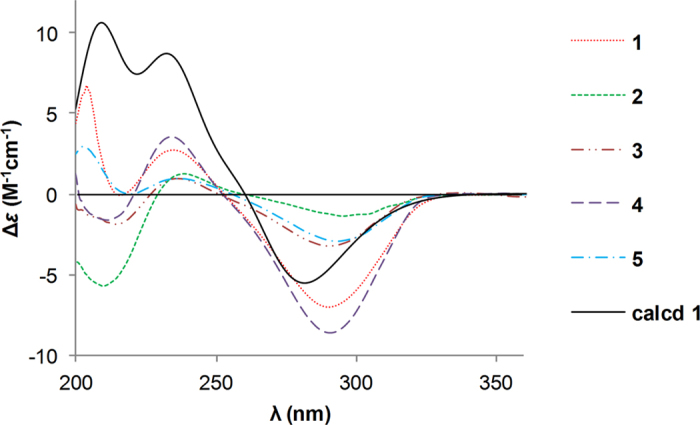
Calculated ECD spectrum of 1 and experimental ECD curves of 1–5.

Comparison of its ^1^H and ^13^C NMR data (Tables 1 and 2) with those of aspergillin PZ (**21**)^26^ indicated that **1** possessed a similar planar structure with that of **21**, except for the presence of an additional carbonyl, a terminal double bond, and a hydroxyl group in the former, instead of a methyl group and two oxygen-bridged sp^3^ carbons. Detailed analyses of ^1^H–^1^H COSY and HMBC spectra of **1** revealed that it has the same carbon rings as **21**. The cleavage of the oxygen-bridge and further oxidation at C-18 (*δ*_C_ 211.4, carbonyl) and C-25 (*δ*_C_ 115.7; *δ*_H_ 5.08 and 4.79, CH_2_) of **1** was determined by HMBC correlations from H-17 and H-19 to C-18 and from H-25 to C-13, C-14, and C-15 (Fig. 2). Moreover, the hydroxylation at C-20 was revealed by its chemical shifts (*δ*_C_ 74.5; *δ*_H_ 5.19, CH) and HMBC correlations from H-20 to C-18 and C-21. The NOESY (Fig. 3) correlation between H-8 and H-19 indicated their cofacial and *β*-orientations. Consequently, H-13 was determined to be *α*-oriented by its splitting pattern and large coupling constants (t, *J* = 11.7 Hz) with H-8 and H-19, suggesting a *trans*-fused C/D rings of **1**. Inaddition, NOESY correlations from H-17 and H-20 to H-13, together with the large coupling constants between H-19 and H-20 (*J* = 11.7 Hz), revealed that they were also *α*-oriented. Thus, the relative configuration of **1** was finally determined. It is noteworthy that compound **1** is the first cytochalasan with a terminal double bond at the macrocyclic ring. The absolute configuration of **1** was established by ECD calculation (Fig. 4), and the calculated ECD spectrum of **1** was comparable with that of the experimental ECD curve of **1**.

**Figure 5:**
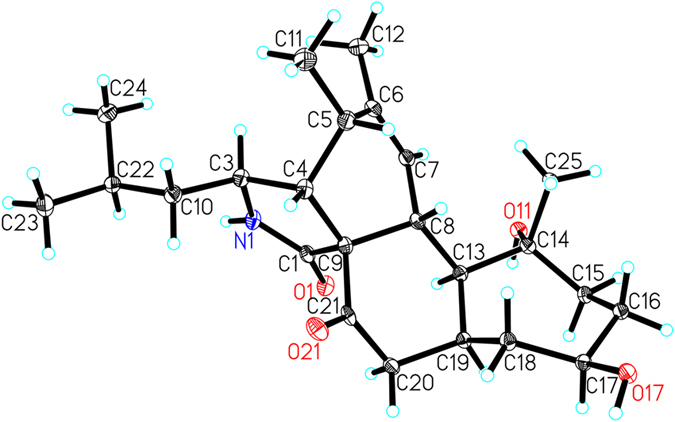
X-ray structure of compound 2.

The molecular formula of C_24_H_37_NO_4_ was determined for flavichalasine B (**2**) by ^13^C NMR data and an ion peak at *m/z* 426.2607 [M + Na]^+^ in the HRESIMS spectrum. Comparison of the ^1^H and ^13^C NMR data of **2** (Tables 1 and 2) with **1** aided by analyses of its ^1^H–^1^H COSY and HMBC spectra revealed that they shared the same ring system and carbon skeleton. Detailed elucidation of the 2D NMR date of **2** suggested that the main differences between **2** and **1** rested in the hydrolyzation of the terminal double bond and the reduction of C-18 and C-20 in the former. NOESY correlations between H-13 and H-19, together with the coupling constant (*J* = 12.0 Hz) between H-8 and H-13, confirmed the *trans*-diaxial relationship between these hydrogens, as found in **1**, and, consequently, the *α*-orientation of H-13 and H-19, thus indicating a *cis*-fused junction of C/D rings (Fig. 3). In addition, NOESY correlations of H-8/Me-25 determined the *β*-orientation of Me-25 while correlation of H-19/H-17 confirmed the *α*-orientation of H-17. X-ray crystallographic analysis of **2** was performed (Fig. 5, CCDC 1502872), which confirmed the former elucidated structure of **2** as well as its absolute configuration (Flack parameter = 0.11(5))^27^.

The molecular formula of flavichalasine C (**3**) was determined to be C_24_H_35_NO_5_ by HRESIMS peak at *m/z* 440.2396 [M + Na]^+^, with one oxygen atom more than that of **21**. The presence of an additional hydroxyl group at C-20 was deduced from its chemical shifts (*δ*_C_ 73.7 and *δ*_H_ 5.16) and confirmed by analyses of 2D NMR spectra. Furthermore, the oxygen-bridge in D ring was formed between C-14 and C-17 rather than C-14 and C-18, which was established by HMBC correlations from H-17 to C-14. NOESY correlations of H-8/H-19 and H-13/H-20 as well as the coupling patterns of H-8, H-13, and H-20 were similar to those of **1**, indicating identical configurations at C-13, C-19, and C-20 (Fig. 3). In addition, NOESY correlations of H-18/H-20 indicated that the hydroxyl group at C-18 was *β*-oriented. Finally, NOESY interactions from H-13 to H-15 and H-16 suggested that the oxygen-bridge should adopt a *β*-configuration.

The only difference between flavichalasine D (**4**) and **3** was that the oxygenated methine of C-18 in **3** was substituted by a carbonyl group (*δ*_C_ 215.5) in **4**, which was supported by 2D NMR and HRESIMS data [(M + H)^+^ ion peak at *m/z* 416.2433]. Moreover, the relative configuration of **4** was also identical with that of **3** as revealed by NOESY correlations of H-8/H-19, H-13/H-20, H-13/H-15, and H-13/H-16 as well the coupling patterns of H-8, H-13, and H-20.

The overall NMR spectra of flavichalasine E (**5**) (Tables 1 and 2) closely resembled those of **3** excepting the presence of a hemiketal carbon resonance at *δ*_C_ 103.6 in **5**, instead of the oxygenated methane carbon resonance of the oxacyclic ring at *δ*_C_ 76.4. This carbon signal was determined to be C-18 by HMBC correlations from H-13, H-19, and H-20 to C-18. The oxygen-bridge between C-14 and C-18 was deduced by taking the chemical shift of C-14 (*δ*_C_ 82.9), which was similar to that of the same carbon in **3** and **4**, and the stability of the hemiketal group into consideration. Thus, the planar structure of **5** was determined. The relative configuration of rings A–C in **5** was shown to be identical to those of **3** by analyses of the NOESY data (Fig. 3) and ^1^H–^1^H coupling constants. However, NOESY correlations from H-8 and H-19 to H-16*β (δ*_H_ 1.49) suggested that the oxygen-bridge in **5** should be *α*-oriented. In addition, the coupling constant values of H-17 (9.2 and 6.9 Hz) along with its NOESY interactions with H-16*α (δ*_H_ 1.97), indicated it should be *α*-axially located. Therefore, the structure of **5** with relative configuration was established. This is the only cytochalasan with an *α*-oriented oxygen-bridge in D ring.

The absolute configurations of **3**–**5** were determined as shown by comparison of their ECD spectra with those of **1** and **2** (Fig. 4).

Flavichalasine F (**6**) was also isolated as colorless powder. The molecular formula of C_25_H_39_NO_5_ was assigned by the positive HRESIMS, which indicated 14 mass units more than aspochalasin E (**14**)^28^. The ^1^H and ^13^C NMR data of **6** (Tables 2 and 3) closely resembled those of **14** with the presence of an additional methoxyl (*δ*_H_ 3.46; *δ*_C_ 58.2) and a downfield shifted C-19 (*δ*_H_ 3.06/*δ*_C_ 79.7 for **6**; *δ*_H_ 3.17/*δ*_C_ 67.7 for **14**). These analyses indicated that the 19-OH in **14** was replaced by the methoxyl group in **6**, which was confirmed by HMBC correlations from 19-OCH_3_ to C-19 (Fig. 2). NOESY correlation (Fig. 3) between H-8 and Me-25, together with the large coupling constant (*J* = 11.0 Hz) between H-8 and H-13, revealed the (E)-substitution of the double bond as well as the *α*-orientation of H-13. Moreover, NOESY correlations from H-13 to H-17 and H-20*α* confirmed the conformation of the macrocyclic ring. Therefore, based on the molecular modeling, orientations of H-18 and H-19 were assigned as shown by NOESY interactions of H-18/H-17, H-18/H-20*α*, and Me-25/H-19. Thus, the relative configuration of **6** was defined.

Flavichalasine G (**7**) had the molecular formula of C_24_H_37_NO_4_, with one oxygen atom less than **14**, as evidenced by the HRESIMS ion at *m/z* 426.2602 [M + Na]^+^ (calcd for C_24_H_37_NO_4_Na, 426.2620). The ^1^H and ^13^C NMR data (Tables 2 and 3) of **7** showed similarities to compound **14** except for the absence of a hydroxyl group at C-19, which was further confirmed by ^1^H–^1^H COSY cross-peaks of H-17/H-18/H-19/H-20 and HMBC correlations from H-19 to C-17 and H-20 to C-18. All of chiral centers of **7** were in complete agreement with those of **6**, as demonstrated by the key correlations observed in the NOESY spectrum of **7**.

Flavichalasine H (**8**) gave a HRESIMS ion peak at *m/z* 410.2678 [M + Na]^+^ (calcd for C_24_H_37_NO_3_Na, 410.2671). The ^1^H and ^13^C NMR spectra of **8** (Tables 2 and 3) showed remarkable similarities to those of compound **7**, but bearing only one oxygenated methine group (*δ*_H_ 3.61; *δ*_C_ 70.4) in the macrocyclic ring. The hydroxyl group was located at C-17 by ^1^H–^1^H COSY cross-peaks of H-15/H-16/H-17/H-18/H-19/H-20 and HMBC correlations from H-16 and H-19 to C-17. Compound **8** had the same relative configuration as **7** revealed by its NOESY spectrum. The 17-OH group was established to be *β*-oriented by the key NOESY correlation of H-13/H-17. Therefore, the structure of **8** was established.

Flavichalasine I (**9**) had a molecular formula of C_24_H_35_NO_4_ as determined by the sodium adduct ion in HRESIMS [M + Na + H]^+^ at *m/z* 425.2526. Analyses of the 1D and 2D NMR data (Tables 2 and 3) suggested that it shared the same planar structure with aspochalasin M (**18**)^29^. The manifest difference is the relative configuration of C-18, which was evidenced by the NOESY correlations of Me-25 to H-18 and H-16*β* and H-18 to H-16*β*. Therefore, compound **9** was determined to be the C-18 epimer of **18**.

Flavichalasines J (**10**) and K (**11**) possessed the same molecular formula of C_24_H_35_NO_5_ as assigned by their HRESIMS data, and they shared closely resembled ^1^H and ^13^C NMR data. Further analyses of their 2D NMR confirmed them to be C-20 hydroxylated derivatives of **9** by the ^1^H–^1^H COSY cross-peaks of H-18/H-19/H-20 and HMBC correlations from H-18 and H-19 to C-20 and H-20 to C-21. The relative configurations of **10** and **11** were determined by NOESY experiments. NOESY interactions of Me-25 to H-18 and H-13 to H-20 of **10** suggested the *β* and *α* orientations of H-18 and H-20, respectively. The relative configuration of **11** was similar to that of **10** except for the orientation of 18-OH, which is evidenced by the NOESY interactions of H-13 to H-15*α* and H-15*α* to H-18.

The molecular formula of flavichalasine L (**12**) was determined to be C_25_H_37_NO_5_ with eight degrees of unsaturation. The ^1^H and ^13^C NMR spectra of **12** (Tables 2 and 3) showed remarkable similarities to those of aspochalasin R^11^. Comprehensive analyses of the NMR data showed that the only difference between **12** and aspochalasin R was the 19-OCH_3_ in aspochalasin R transferred to C-20 in **12.** This conclusion was confirmed by HMBC correlations from H-19 to C-17, C-18, C-20, and C-21 and from H-20 to C-18, C-19, and C-21. The relative configurations of H-17 and H-20 of **12** were determined to be *α*-oriented by NOESY correlations of H-13 to H-17 and H-20 and H-17 to H-13.

Flavichalasine M (**13**) possessed the molecular formula of C_24_H_35_NO_5_ as deduced from the HRESIMS. Comparison of its 1D and 2D NMR data (Tables 2 and 3) with those of **12** showed that the only difference between them was the absence of the methoxyl group in **13**. The relative configurations of all stereocenters of **13** were identical to those of **12**, as established by analysis of the NOESY spectrum and by comparison of their NMR data.

**Figure 6:**
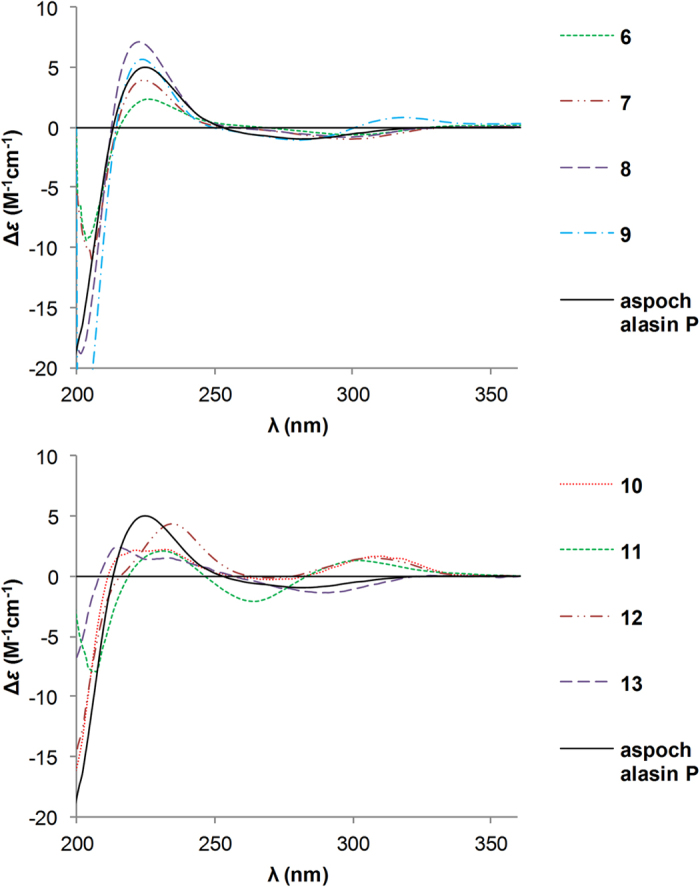
Experimental ECD spectra of 6–13 and aspochalasin P.

The absolute configurations of **6**–**13** were identified by comparisons of their ECD spectra with that of aspochalasin P (Fig. 6), whose absolute configuration was determined by X-ray diffraction analysis in our previous research^24^.

Compound **14** was elucidated to have the same planar structrue as that of aspochalasin E by analyzing its ^1^H and ^13^C NMR as well as 2D NMR including ^1^H–^1^H COSY and HMBC spectra. To verify if it is aspochalasin E, ^1^H and ^13^C NMR of **14** were further recorded in DMSO-*d*_6_, and these spectra were identical with aspochalasin E. The relative configuration of aspochalasin E was not determined in the literature, and in this case, NOESY experiment was performed to establish its relative configuration. The NOESY interactions of H-13/H-17, H-13/H-20*α*, H-17/H-20*α*, H-18/H-17, H-18/H-20*α*, and Me-25/H-19 were similar to those of **6**, suggesting the same relative configurations for **14** and **6**.

Compound **15** was determined to be aspochalasin T by comparisons of its ^1^H and ^13^C NMR data with those reported in literature^11^. Its relative configuration, which was not determined in the literature, was elucidated by NOESY spectrum in this study. Based on the aforementioned conformation of the macrocyclic ring, NOESY correlations of H-13/H-17 and Me-25/H-19 revealed the *α*-orientation of H-17 and *α*-orientation of H-19, respectively.

Six other known analogues (**16**−**21**) were identified as aspochalasins D (**16**)^30^, aspochalasins H (**17**)^31^, aspochalasins M (**18**)^29^, aspochalasins Q (**19**)^29^, trichalasin H (**20**)^32^, and aspergillin PZ (**21**)^26^ by comparison of their NMR spectroscopic data with the literature values.

### Activities Evaluation

**Figure 7:**
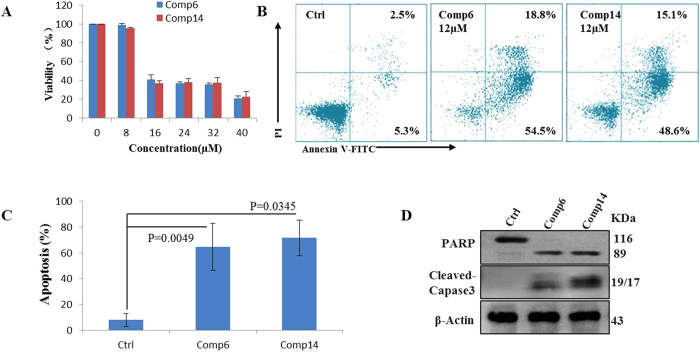
(**A**) The viability of HL60 cells after treated with compounds 6 and 14 were determined by CCK-8 kit. Mean ± SD of three independent experiments. (**B**) Cell apoptosis was determined by Annexin V-FITC and PI staining using flow cytometric analysis. (**C**) Columns, means of three independent FACS assay for apoptosis; bars, SD, P value were calculated by two-tailed student’s t-test. (**D**) Western blot analysis for the apoptosis marker PARP and cleaved-caspase-3, *β*-Actin was used as a loading control.

Compounds (**1**−**14**) were biologically evaluated for *in vitro* cytotoxicity against seven human cancer cell lines (Jurkat, HL60, NB4, 231, HEP-3B, HCT116, and RKO). Taxol were used as positive controls for antitumor activity. Compounds **6** and **14** exhibited moderate cytotoxic activities, with IC_50_ values ranging from 9.6 to 26.6 *μ*M (Table 4). The other compounds showed no significant inhibitory effects on the proliferation of the tested cancer cells. To analyze the apoptosis induction potential of compounds **6** and **14**, an apoptosis assay was performed by using flow cytometry analysis (Fig. 7). As shown in Fig. 7b, compounds **6** and **14** induced significant apoptosis of HL60 cells compared to the control group. Moreover, treatment with both compounds altered the expression levels of caspase-3 and PARP (Fig. 7d). These data suggest that compounds **6** and **14** induced apoptosis by activation of caspase-3 and degradation of PARP.

## Experimental Section

### General

Optical rotations were determined with an AUTOPOL IV-T Automatic polarimeter. The UV and ECD spectra and FT-IR spectra were measured using a Varian Cary 50 instrument or LabRAM HR800 instrument, a JASCO-810 ECD spectrometer, and a Bruker Vertex 70 instrument, respectively. The NMR spectra were recorded on a Bruker AM-400 spectrometer. The ^1^H and ^13^C NMR chemical shifts were referenced to the solvent or solvent impurity peaks for CD_3_OD (*δ*_H_ 3.31 and *δ*_C_ 49.0) and DMSO-*d*_6_ (*δ*_H_ 2.50 and *δ*_C_ 39.5). HRESIMS data were obtained in the positive ion mode on a Thermo Fisher LTQ XL spectrometer. Semipreparative HPLC was carried out using a Dionex HPLC system equipped with an Ultimate 3000 pump, an Ultimate 3000 autosampler injector, and an Ultimate 3000 DAD detector controlled by Chromeleon software (version 6.80), using a reversed-phase C_18_ column (5 *μ*m, 10 × 250 mm, Welch Ultimate XB-C_18_). Column chromatography (CC) was performed using silica gel (100–200 and 200–300 mesh; Qingdao Marine Chemical Inc., China), ODS (50 *μ*m, Merck, Germany), and Sephadex LH-20 (GE Healthcare Bio-Sciences AB, Sweden). Thin-layer chromatography (TLC) was performed on silica gel 60 F_254_ (Yantai Chemical Industry Research Institute) and RP-C_18_ F_254_ plates (Merck, Germany).

### Fungal Material

The fungus *Aspergillus flavipes* was derived from the intertidal zone of the Yangtze River, Wuhan, Hubei Province, P. R. China. The sequence data for this strain have been submitted to the DDBJ/EMBL/GenBank under accession No. KP339510. A voucher sample (ID: QM507) was preserved in the herbarium of the Huazhong University of Science and Technology, P. R. China.

### Fermentation and Isolation

The strain was cultured on potato dextrose agar (PDA) at 28 °C for 7 days to prepare the seed culture. Agar plugs were cut into small pieces (approximately 0.5 × 0.5 × 0.5 cm^3^) and inoculated into 200 Erlenmeyer flasks (1 L), previously sterilized by autoclaving, each containing 200 g rice and 200 mL distilled water. All flasks were incubated at 28 °C for 28 days. After that, the culture broth was extracted with ethyl alcohol, and the ethyl alcohol was removed under reduced pressure to yield a crude extract (3.3 kg). The crude extract was partitioned with ethyl acetate against water to obtain the ethyl acetate soluble part (1.5 kg). The ethyl acetate extract was subjected to chromatography on a silica gel column (100–200 mesh) eluting with CH_2_Cl_2_–MeOH (100:1–1:1) progressively to obtain four fractions (Fr. A–Fr. D) based on their TLC profiles. Fr. B (253 g) was subjected to column chromatography (CC, petroleum ether/ethylacetate, 20:1 to 1:1) over silica gel (200–300 mesh) to yield five subfractions (Fr. B.1–Fr. B.5). Fr. B.3 (13.6 g) was purified over ODS CC (MeOH–H_2_O, 20%) to give four subfractions (Fr. B.3.1–Fr. B.3.4), Fr. B.3.2 (303.6 mg) was fractionated on semipreparative RP-18 HPLC (65% MeCN in H_2_O, 2 ml/min) to afford compounds **3** (8.0 mg), **14** (15 mg), **6** (10 mg), **12** (7.8 mg), and **16** (5.1 mg). Fr. B.4 (25.1 g) was fractioned on Sephadex LH-20 (MeOH) to get three subfractions (Fr. B.4.1–Fr. B.4.3). Fr. B.4.2 (264,5 mg) was further purified by semipreparative RP-18 HPLC(69% MeOH in H_2_O, 2 ml/min) to afford compounds **1** (5.4 mg), **4** (12.5 mg), **9** (5.6 mg), **11** (8.8 mg), and **22** (30 mg). Compounds **13** (9.5 mg), **17** (16.5 mg), and **18** (5.6 mg) were obtained from subfraction Fr. B.4.3 (3.7 g) by silica gel (200–300 mesh) CC (PE–acetone, 3:1) and semipreparative RP-18 HPLC. Fr. C (233 g) was subjected to an RP-18 silica gel CC (MeOH/H_2_O, 40–100%) to yield four main fractions (Fr. C.1–Fr. C.4). compounds **7** (20.8 mg), **8** (13.1 mg), **15** (10.1 mg), and **19** (18 mg) were obtained from Fr. C.2 (45.7 g) by Sephadex LH-20 (MeOH), silica gel (200–300 mesh) CC (petroleum ether/acetone, 5:1), and semipreparative RP-18 HPLC(52% MeCN in H_2_O, 2 ml/min). By using the same method applied to Fr. C.2, Fr. C.3 (51.3 g) afforded compounds **2** (7.8 mg), **5** (10.5 mg), **10** (8.4 mg), **20** (11.4 mg), and **21** (18 mg).

#### Flavichalasine A (**1**)

Colorless powder, 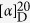 −88.1 (*c* = 0.19, MeOH); UV (MeOH) *λ*_max_ (log *ε*) = 204 (4.53) nm; IR *v*_max_ = 3357, 2959, 2933, 1713, 1689, 1439, 1364, 1224, 1083 cm^−1^; ECD (MeOH) *λ* (Δ*ε*) 204 (+6.8), 235 (+2.7), 290 (−7.0) nm; for ^1^H NMR (400 MHz) and ^13^C NMR (100 MHz) data, see Tables 1 and 2; HRESIMS [M + H]^+^ *m/z* 416.2433 (calcd for C_24_H_34_NO_5_, 416.2437).

#### Flavichalasine B (**2**)

Colorless powder, 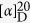 −39.7 (*c* = 0.18, MeOH); IR *v*_max_ = 3433, 1690, 1630, 1384 cm^−1^; UV (MeOH) *λ*_max_ (log *ε*) = 203 (3.38) nm; ECD (MeOH) *λ* (Δ*ε*) 210 (−5.7), 239 (+1.3), 295 (−1.3) nm; for ^1^H NMR (400 MHz) and ^13^C NMR (100 MHz) data see Tables 1 and 2; HRESIMS [M + Na]^+^ *m/z* 426.2607 (calcd for C_24_H_37_NO_4_Na, 426.2620). Crystal data for flavichalasine B (**2**): C_24_H_37_NO_4_, *M* = 403.54, *a* = 15.2860(2) Å, *b* = 7.86750(10) Å, *c* = 18.8029(3) Å, *α* = 90°, *β* = 92.76°, *γ* = 90°, *V* = 2258.67(5) Å^3^, *T* = 100(2) K, space group *P*21, *Z* = 4, *μ*(CuKα) = 0.632 mm^−1^, 23347 reflections measured, 6689 independent reflections (*R*_*int*_ = 0.0315). The final *R*_*1*_ values were 0.0338 (*I* > 2*σ(I*)). The final *wR(F*^2^) values were 0.0900 (*I* > 2*σ(I*)). The final *R*_*1*_ values were 0.0340 (all data). The final *wR(F*^2^) values were 0.0903 (all data). The goodness of fit on *F*^2^ was 1.090. Flack parameter = 0.11(5).

#### Flavichalasine C (**3**)

Colorless powder, 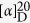 −28.2 (*c* = 0.28, MeOH); IR *v*_max_ = 3433, 1694, 1631, 1384 cm^−1^; UV (MeOH) *λ*_max_ (log *ε*) = 202 (3.7) nm; ECD (MeOH) *λ* (Δ*ε*) 215 (−1.9), 236 (+1.0), 291 (−3.2) nm; for ^1^H NMR (400 MHz) and ^13^C NMR (100 MHz) data see Tables 1 and 2; HRESIMS [M + Na]^+^ *m/z* 440.2396 (calcd for C_24_H_35_NO_5_Na, 440.2413).

#### Flavichalasine D (**4**)

Colorless powder, 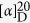 −28.7 (*c* = 0.12, MeOH); UV (MeOH) *λ*_max_ (log *ε*) = 202 (3.15) nm; IR *v*_max_ = 3191, 2960, 1736, 1690, 1461, 1383, 1363, 1224, 1097, 1050 cm^−1^; ECD (MeOH) *λ* (Δ*ε*) 211 (−1.6), 234 (+3.5), 291 (−8.6) nm; for ^1^H NMR (400 MHz) and ^13^C NMR (100 MHz) data, see Tables 1 and 2; HRESIMS [M + H]^+^ *m/z* 416.2433 (calcd for C_24_H_34_NO_5_, 416.2437).

#### Flavichalasine E (**5**)

Colorless powder, 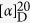 −23.8 (*c* = 0.21, MeOH); IR *v*_max_ = 3433, 1690, 1631, 1384 cm^−1^; UV (MeOH) *λ*_max_ (log *ε*) = 203 (3.7) nm; ECD (MeOH) *λ* (Δ*ε*) 203 (+2.9), 237 (+1.0), 293 (−2.9) nm; for ^1^H NMR (400 MHz) and ^13^C NMR (100 MHz) data see Tables 1 and 2; HRESIMS [M + Na]^+^ *m/z* 456.2362 (calcd for C_24_H_35_NO_6_Na, 456.2362).

#### Flavichalasine F (**6**)

Colorless powder, 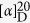 −62.6 (*c* = 0.38, MeOH); UV (MeOH) *λ*_max_ (log *ε*) = 203 (4.04) nm; ECD (MeOH) *λ* (Δ*ε*) 204 (−9.3), 226 (+2.4) nm; IR *v*_max_ = 3422, 2956, 2933, 1688, 1448, 1367, 1103 cm^−1^; for ^1^H NMR (400 MHz) and ^13^C NMR (100 MHz) data see Tables 2 and 3; HRESIMS [M + Na]^+^ *m/z* 456.2705 (calcd for C_25_H_39_NO_5_Na, 456.2726).

#### Flavichalasine G (**7**)

Colorless powder, 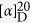 + 64.6 (*c* = 0.40, MeOH); UV (MeOH) *λ*_max_ (log *ε*) = 203 (3.64) nm; ECD (MeOH) *λ* (Δ*ε*) 206 (−11.0), 224 (+3.9) nm; IR *v*_max_ = 3436, 2928, 1691, 1633, 1455, 1386, 1112,1054 cm^−1^; for ^1^H NMR (400 MHz) and ^13^C NMR (100 MHz) data see Tables 2 and 3; HRESIMS [M + Na]^+^ *m/z* 426.2602 (calcd for C_24_H_37_NO_5_Na, 426.2620).

#### Flavichalasine H (**8**)

Colorless powder, 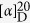 −22.7 (*c* = 0.39, MeOH); UV (MeOH) *λ*_max_ (log *ε*) = 202 (3.75) nm; IR *v*_max_ = 3201, 2932, 1689, 1444, 1363, 1302, 1221, 1106 cm^−1^; ECD (MeOH) *λ* (Δ*ε*) 222 (+7.1) nm; for ^1^H NMR (400 MHz) and ^13^C NMR (100 MHz) data see Tables 2 and 3; HRESIMS [M + Na]^+^ *m/z* 410.2678 (calcd for C_24_H_37_NO_3_Na, 410.2671).

#### Flavichalasine I (**9**)

Colorless powder, 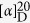 −85.5 (*c* = 0.11, MeOH); UV (MeOH) *λ*_max_ (log *ε*) = 205 (4.15) nm; ECD (MeOH) *λ* (Δ*ε*) 225 (+5.6) nm; IR *v*_max_ = 3422, 2959, 2933, 1766, 1693, 1444, 1384, 1299, 1083 cm^−1^; for ^1^H NMR (400 MHz) and ^13^C NMR (100 MHz) data see Tables 2 and 3; HRESIMS [M + Na + H]^+^ *m/z* 425.2526 (calcd for C_24_H_36_NO_4_Na, 425.2542).

#### Flavichalasine J (**10**)

Colorless powder, 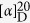 + 44.1 (*c* = 0.11 MeOH); UV (MeOH) *λ*_max_ (log *ε*) = 203 (4.08) nm; ECD (MeOH) *λ* (Δ*ε*) 225 (+2.1), 308 (+1.6) nm; IR *v*_max_ = 3394, 2959, 2926, 1709, 1687, 1441, 1385, 1058 cm^−1^; for ^1^H NMR (400 MHz) and ^13^C NMR (100 MHz) data see Tables 2 and 3; HRESIMS [M + Na]^+^ *m/z* 440.2407 (calcd for C_24_H_35_NO_5_Na, 440.2413).

#### Flavichalasine K (**11**)

Colorless powder, 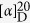 −11.3 (*c* = 0.12, MeOH); UV (MeOH) *λ*_max_ (log *ε*) = 202 (3.93) nm; ECD (MeOH) *λ* (Δ*ε*) 206 (−8.0), 232 (+2.1), 264 (−2.1), 302 (+1.3) nm; IR *v*_max_ = 3346, 2959, 2931, 1766, 1690, 1442, 1364, 1223, 1078 cm^−1^; ECD (MeOH) *λ* (Δ*ε*) 205 (−7.9), 231 (+2.1), 264 (−2.1), 302 (+1.3) nm; for ^1^H NMR (400 MHz) and ^13^C NMR (100 MHz) data see Tables 2 and 3; HRESIMS [M + Na]^+^ *m/z* 440.2412 (calcd for C_24_H_35_NO_5_Na, 440.2413).

#### Flavichalasine L (**12**)

Colorless powder, 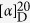 −10.7 (*c* = 0.59, MeOH); UV (MeOH) *λ*_max_ (log *ε*) = 203 (3.88) nm; IR *v*_max_ = 3316, 2956, 1718, 1687, 1442, 1364, 1222, 1088 cm^−1^; ECD (MeOH) *λ* (Δ*ε*) 235 (+4.3), 307 (+1.5) nm; for ^1^H NMR (400 MHz) and ^13^C NMR (100 MHz) data see Tables 2 and 3; HRESIMS [M + H]^+^ *m/z* 432.2737 (calcd for C_25_H_38_NO_5_, 432.2750).

#### Flavichalasine M (**13**)

Colorless powder, 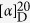 + 18.3 (*c* = 0.11, MeOH); UV (MeOH) *λ*_max_ (log *ε*) = 203 (4.16) nm; ECD (MeOH) *λ* (Δ*ε*) 215 (+2.4), 233 (+1.5), 288 (−1.3) nm; IR *v*_max_ = 3434, 2959, 2926, 1718, 1687, 1440, 1385, 1089 cm^−1^; for ^1^H NMR (400 MHz) and ^13^C NMR (100 MHz) data see Tables 2 and 3; HRESIMS [M + Na]^+^ *m/z* 440.2411 (calcd for C_24_H_35_NO_5_Na, 440.2413).

### Computational details

Conformational analyses were carried out for compound **1** using both BALLOON (Vainio and Johnson, 2007) and confab (O′Boyle *et al*., 2011) programs. The BALLOON program searches conformational space with genetic algorithm, whereas the confab program systematically generates diverse low energy conformations that are supposed to be close to crystal structures. The conformations generated by both programs were grouped together by removing duplicated conformations whose root mean square (RMS) distance was less than 0.2 Å. Semi-empirical PM3 quantum mechanical geometry optimizations were performed on conformations using the Gaussian 09 (Frisch *et al*., 2009) program. Duplicated conformations after geometry optimization were then identified and removed. Remaining conformations were further optimized at B3LYP/6–31 G* level of theory in methanol solvent with IEFPCM3 (Tomasi *et al*., 2005) solvation model using Gaussian 09 program, and duplicated conformations emerging after these calculations were removed according to the same RMS criteria above. Harmonic vibrational frequencies were performed to confirm the stability of the finally obtained conformers. Oscillator strengths and rotational strengths of 20 weakest electronic excitations of each conformer were calculated using the TDDFT methodology at the B3LYP/6-311++G** level of theory with methanol as solvent by the IEFPCM solvation model implemented in Gaussian 09 program. ECD spectra for each conformer were then simulated by using a Gaussian function with a bandwidth *σ* of 0.45 eV. Calculated spectra for each conformation were combined after Boltzmann weighting according to their population contribution.

### Cytotoxicity assay

Seven human cancer cell lines (HL-60 and NB4: human promyelocytic leukemia cell lines; Jurkat: human T lymphocyte cell line; MDA-MB-231: human breast cancer cell line; HEP-3B: human liver cancer cell line; HCT116 and RKO: human colon cancer cell lines) were seeded at a density of 3–5 × 10^3^ per well in 96-well plates and incubated overnight, and then treated with compounds at various concentrations. DMSO (<0.1%) was used as a negative control and taxol (2 *μ*M) was used as a positive control. After 48 h treated, the viability was determined using a CCK-8 kit according to the manufacturer’s instructions. The 50% inhibiting concentration (IC_50_) was calculated by SPSS software version13.0.

### Apoptosis analysis

Cell morphological changes were observed with inverted light microscopy (NIKON, Tokyo, Japan). To identification of the apoptotic induction effect of compounds **6** and **14**, a FITC-labeled Annexin V/PI apoptosis detection Kit (Keygen, Nanjing, China) was used according to the manufacturer’s instructions. Briefly, HL60 cells were exposed to vehicle control (DMSO, <0.1%), compounds **6** (12 μM) and **14** (12 μM). After 48 h, cells were harvested and washed with PBS and resuspended in binding buffer, and then, AnnexinV-FITC and PI were added. After staining for 15 minutes, the cells were immediately analyzed using flow cytometry (Becton Dickinson, USA).

### Western blot analysis

Western blot analysis was conducted as described previously^24^. Briefly, HL60 cells were incubated with DMSO, compounds **6** (12 μM) and **14** (12 μM) for 48 h, and then lysed in a radio immune-precipitation assay buffer. Protein concentrations were determined using a BCA protein assay kit and equalized before loading. Samples were denatured and subjected to electrophoresis in 10% SDS-PAGE gels followed by transfer to PVDF membrane and probed with specific antibodies, including PARP, Cleaved Caspase 3 and β-Actin (Cell Signaling Technology, Inc.). Blots bands were visualized using the horseradish peroxidase conjugated secondary antibodies and chemiluminescent substrate.

## Additional Information

**How to cite this article**: Wei, G. *et al*. Flavichalasines A–M, cytochalasan alkaloids from *Aspergillus flavipes. Sci. Rep.* **7**, 42434; doi: 10.1038/srep42434 (2017).

**Publisher's note:** Springer Nature remains neutral with regard to jurisdictional claims in published maps and institutional affiliations.

## Supplementary Material

Supplementary Information

## Figures and Tables

**Figure 1:**
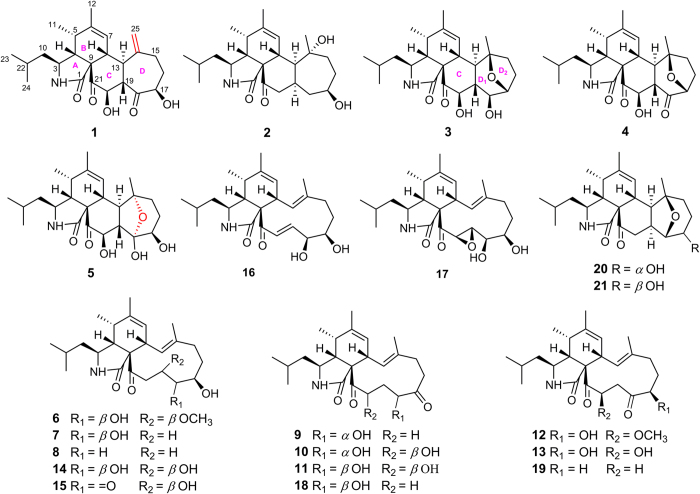
Structures of isolated compounds.

**Table 1:** ^1^H NMR data of flavichalasines A–E (1–5) in CD_3_OD (*J* in Hz).

*no.*	1	2	3	4	5
3	3.26 m	3.23 ddd (8.7, 5.1, 2.3)	3.26 ddd (8.7, 5.6, 2.0)	3.26 ddd (8.7, 5.2, 2.5)	3.26 ddd (8.6, 5.2, 2.2)
4	3.11 m	2.92 dd (5.9, 2.3)	3.06 dd (6.0, 2.0)	3.11 dd (6.1, 3.7)	2.96 dd (5.9, 2.2)
5	2.40 m	2.41 m	2.39 m	2.45 m	2.48 m
7	5.54 br s	6.14 br s	5.52 br s	5.53 br s	5.74 br s
8	2.40 m	2.46 br d (12.0)	2.25 br d (12.4)	2.53 m	2.50 m
10	1.31 m	1.32 ddd (13.7, 8.7, 5.1) 1.21 ddd (13.7, 8.7, 5.1)	1.23 m	1.26 m	1.40 m; 1.29 m
11	1.25 d (7.3)	1.24 d (7.2)	1.24 d (7.3)	1.26 d (7.3)	1.26 d (7.2)
12	1.76 s	1.79 br s	1.79 br s	1.82 s	1.80 br s
13	4.02 t (11.7)	3.35 dd (12.0, 5.3)	3.01 t (12.4)	2.99 dd (13.3, 12.1)	3.87 t (11.9)
15a	2.43 m	2.06 m	1.83 m	1.92 m	1.64 m
15b	2.28 m	1.50 m	1.47 m	1.65 m	
16a	1.87 m	1.96 m	2.64 m	2.48 m	1.97 m
16b	1.80 m	1.50 m	1.86 m	2.01 m	1.49 m
17	4.51 dd (7.2, 4.2)	3.75 m	4.41 td (8.3, 1.9)	4.36 dd (9.6, 2.2)	3.68 dd (9.2, 6.9)
18		1.55 m	4.35 dd (8.1, 5.2)		
19	3.08 t (11.7)	2.76 m	1.79 m	3.08 dd (13.3, 10.2)	3.26 ddd (8.6, 5.2, 2.2)
20	5.19 d (11.7)	3.53 dd (12.8, 6.2) 2.14 dd (12.8, 3.4)	5.16 d (11.6)	5.08 d (10.2)	4.88 d (12.1)
22	1.64 m	1.60 m	1.61 m	1.61 m	1.63 m
23	0.94 d (6.4)	0.91 d (6.3)	0.91 d (6.2)	0.91 d (6.6)	0.93 d (6.4)
24	0.92 d (6.5)	0.93 d (6.3)	0.92 d (6.2)	0.93 d (6.6)	0.94 d (6.4)
25	5.08 s 4.79 s	1.28 s	1.22 s	1.42 s	1.42 s

**Table 2:** ^13^C NMR for compounds 1–13 (100 MHz).

*no.*	1^a^	2^a^	3^a^	4^a^	5^a^	6^a^	7^b^	8^a^	9^a^	10^a^	11^a^	12^a^	13^a^
1	175.0	175.8	175.3	174.9	175.1	177.6	174.1	177.6	176.8	176.7	175.8	176.6	176.9
3	52.7	52.5	52.6	52.9	52.55	52.3	49.7	52.1	51.9	52.2	52.3	52.3	52.4
4	48.5	47.9	47.6	47.7	48.5	54.4	50.7	52.9	52.3	53.6	51.2	55.2	56.9
5	35.8	35.3	35.3	35.3	36.2	36.6	34.8	36.4	36.4	36.7	36.3	36.6	36.7
6	141.5	139.4	140.7	141.8	143.5	140.9	139.4	141.0	141.4	141.5	142.2	141.3	141.1
7	124.9	128.4	127.2	126.5	123.2	126.7	125.4	127.1	126.3	126.2	126.3	126.4	126.6
8	47.3	43.7	46.6	45.6	44.3	44.6	43.0	44.1	44.7	45.5	44.3	44.2	43.4
9	67.9	68.6	68.0	67.8	69.2	69.2	67.6	69.3	68.9	66.8	68.0	67.8	68.5
10	49.1	48.8	49.0	49.1	48.7	50.0	48.7	49.6	49.8	49.4	49.2	49.9	50.0
11	13.8	13.6	13.6	13.6	13.8	13.8	13.1	13.8	13.8	13.8	13.9	13.8	13.8
12	20.0	20.1	19.8	19.9	20.1	19.8	19.6	19.8	19.7	19.8	19.8	19.8	19.8
13	40.3	46.7	38.0	39.3	48.3	125.5	124.2	125.7	126.3	126.0	126.5	125.4	126.0
14	149.6	76.2	82.9	83.9	82.9	138.0	135.6	138.1	136.3	136.4	136.8	139.1	138.7
15	32.7	39.1	42.0	41.9	31.8	38.9	38.8	39.9	38.2	38.8	37.6	37.0	35.0
16	38.6	34.5	23.1	31.1	29.4	30.2	28.6	30.8	36.8	37.3	33.2	31.1	32.7
17	75.6	72.5	76.4	81.1	73.0	71.5	72.1	70.4	212.7	212.4	215.4	74.7	75.6
18	211.4	40.2	67.6	215.5	103.6	80.2	73.5	32.8	76.4	74.6	77.0	206.2	210.0
19	59.4	36.4	48.0	53.6	52.6	79.7	27.3	19.5	25.5	34.0	35.9	41.0	42.8
20	74.5	48.1	73.7	72.7	77.5	43.8	33.8	36.7	36.3	69.1	67.1	81.0	74.4
21	205.3	208.3	208.0	205.8	206.5	212.4	212.0	213.6	213.2	209.2	209.2	207.6	211.6
22	25.7	25.7	25.7	25.7	25.7	25.8	24.0	25.7	25.7	25.7	25.8	25.7	25.8
23	23.9	23.9	23.8	23.8	23.8	23.8	23.5	23.8	23.8	23.8	23.9	23.9	23.9
24	22.1	22.2	22.2	22.1	22.2	22.2	21.6	22.2	22.2	22.1	22.0	22.1	22.0
25	115.7	27.6	21.0	21.3	27.4	16.1	15.5	15.6	15.2	15.3	15.4	15.9	16.8
OCH_3_						58.2						58.0	

^a^In CD_3_OD.

^b^In DMSO–*d*_6_.

**Table 3:** ^1^H NMR data of flavichalasines F–M (6–13) (*J* in Hz).

*no.*	6^a^	7^b^	8^a^	9^a^	10^a^	11^a^	12^a^	13^a^
3	3.25 m	3.03 m	3.26 dd (8.2, 5.6)	3.22 m	3.23 ddd (9.1, 5.0, 2.0)	3.18 ddd (9.0, 4.6, 2.7)	3.21 ddd (8.9, 4.8, 2.3)	3.20 ddd (9.1, 4.7, 2.6)
4	2.53 dd (6.1, 1.8)	2.49 m	2.58 m	2.69 dd (6.2, 2.2)	2.60 dd (6.0, 1.8)	2.87 dd (5.9, 2.7)	2.46 dd (5.9, 2.4)	2.42 dd (5.8, 2.6)
5	2.59 m	2.40 m	2.58 m	2.52 m	2.54 m	2.46 m	2.56 m	2.61 m
7	5.41 br s	5.31 br s	5.41 brs	5.31 br s	5.30 br s	5.28 br s	5.28 br s	5.31 br s
8	3.29 m	2.93br d (10.6)	3.18 br d (9.0)	2.99 m	3.06 br d (10.3)	2.86 m	3.14 br d (10.2)	3.32 m
10a	1.22 ddd (13.7, 8.6, 5.3)	1.00 m	1.19 ddd (13.8, 8.7, 5.4)	1.14 ddd (13.8, 8.4, 5.4)	1.35 ddd (14.0, 9.2, 5.2)	1.26 m	1.27 m	1.27 m
10b	1.13 ddd (13.7, 8.6, 5.2)		1.09 ddd (13.8, 8.5, 5.3)	1.07 ddd (13.8, 8.4, 5.4)	1.13 ddd (14.0, 8.7, 5.0)	1.17 m	1.15 ddd (13.6, 8.8, 4.9)	1.16 dd (9.0, 4.7)
11	1.25 d (7.1)	1.15 d (7.1)	1.26 d (6.7)	1.25 d (7.3)	1.26 d (7.1)	1.26 d (7.4)	1.23 d (7.2)	1.24 d (7.2)
12	1.78 br s	1.70 br s	1.78 br s	1.75 br s	1.77 br s	1.78 br s	1.76br s	1.77 br s
13	6.04 d (11.0)	6.11 d (10.7)	6.15 d (10.1)	6.16 d (11.0)	6.20 d (10.8)	6.18 d (10.8)	6.18 d (11.0)	6.12 d (11.0)
15a	2.09 m	2.01 m	2.16 m	2.68 td (12.9, 2.6)	2.56 m	2.72 td (13.1, 2.3)	2.13 m	2.15 m
15b			2.06 m	2.22 dd (11.6, 5.0)	2.25 m	2.14 m	2.10 m	2.09 m
16a	1.61 m	1.69 m	1.78 m	2.97 m	2.98 m	3.26 m	2.31 m	2.17 m
16b	1.48 m	1.25 m	1.52 m	2.11 m	2.15 m	1.96 m	1.75 m	1.98 m
17	3.85 m	3.56 m	3.61 m				4.09 dd (9.2, 1.5)	4.10 m
18	3.57 m	3.45 m	1.67 m; 1.42 m	4.16 m	3.92 dd (8.5, 3.8)	4.04 dd (12.2, 4.5)		
19	3.06 m	1.59 m; 0.98 m	1.59 m; 1.52 m	2.03 m; 1.93 m	2.08 m	1.83 m; 1.61 m	3.78 dd (15.5, 2.8) 2.85 dd (15.5, 8.5)	4.22 d (1.2)
20a	3.95 d (18.3)	3.51 m	3.33 m	3.41 m	4.87 m	4.92 d (9.5)	4.60 dd (8.5, 2.8)	4.51 d (2.2)
20b	1.99 dd (18.3, 4.1)	1.89 m	2.14 m	2.04 m				
22	1.60 m	1.56 m	1.61 m	1.57 m	1.59 m	1.62 m	1.62 m	1.62 m
23	0.92 d (6.6)	0.82 d (6.5)	0.90 d (6.6)	0.89d (6.6)	0.88 d (6.6)	0.89 d (6.7)	0.89 d (6.6)	0.91 d (6.6)
24	0.91 d (6.6)	0.82d (6.5)	0.90 d (6.6)	0.89d (6.6)	0.89 d (6.6)	0.89 d (6.5)	0.89 d (6.6)	0.91 d (6.6)
25	1.50 br s	1.40 br s	1.53br s	1.61 br s	1.61 br s	1.56 br s	1.31 br s	1.35 br s
	3.46 s (OCH_3_)	4.41 d (5.6) (OH-17)					3.32 s (OCH_3_)	
		4.24 d (3.3) (OH-18)						

^a^In CD_3_OD.

^b^In DMSO–*d*_6_.

**Table 4:** Cytotoxicity of compounds 6 and 14 (*μ*M).

Compound	Jurkat	HL60	NB4	231	HEP-3B	HCT116	RKO
**6**	10.5	12.8	12.4	>40	13.6	26.6	11.6
**14**	9.6	12.5	12.8	>40	13.2	25.1	15.2
**Taxol**	<0.064	0.076	<0.064	0.943	0.071	0.169	<0.064
