# Supplementary material for: Flavichalasines A–M, cytochalasan alkaloids from Aspergillus flavipes
Source: Sci Rep. 2017 Feb 13;7:42434. doi: 10.1038/srep42434 (PMC5304325; doi:10.1038/srep42434)

Flavichalasines A–M, cytochalasan alkaloids from *Aspergillus flavipes*

Guangzheng Wei[1], Dongdong Tan[1], Chunmei Chen[1], Qingyi Tong[1], Xiao-Nian Li[2], Jinfeng Huang[1], Junjun Liu[1], Yongbo Xue[1], Jianping Wang[1], Zengwei Luo[1], Hucheng Zhu[1,*], and Yonghui Zhang[1,*]

[1] Hubei Key Laboratory of Natural Medicinal Chemistry and Resource Evaluation, School of Pharmacy, Tongji Medical College, Huazhong University of Science and Technology, Wuhan 430030, People's Republic of China

[2] State Key Laboratory of Phytochemistry and Plant Resources in West China, Kunming Institute of Botany, Chinese Academy of Sciences, Kunming 650204, China

* **Corresponding Authors**

* Tel./fax: +86-027-83692892

Emails: zhangyh@mails.tjmu.edu.cn (Y.Z.);

zhuhucheng@hust.edu.cn (H.Z.)

CONTENTS

Details for ECD calculation 1

HRESIMS,UV, IR, and NMR for compound **1** 15

HRESIMS,UV, IR, and NMR for compound **2** 19

HRESIMS,UV, IR, and NMR for compound **3** 24

HRESIMS,UV, IR, and NMR for compound **4** 28

HRESIMS,UV, IR, and NMR for compound **5** 33

HRESIMS,UV, IR, and NMR for compound **6** 37

HRESIMS,UV, IR, and NMR for compound **7** 42

HRESIMS,UV, IR, and NMR for compound **8** 46

HRESIMS,UV, IR, and NMR for compound **9** 51

HRESIMS,UV, IR, and NMR for compound **10** 55

HRESIMS,UV, IR, and NMR for compound **11** 60

HRESIMS,UV, IR, and NMR for compound **12** 64

HRESIMS,UV, IR, and NMR for compound **13** 69

NMR for compound **14** 76

NMR for compound **15** 79

Details for ECD calculation

Important thermodynamic parameters (a.u.) and Boltzmann distributions of the optimized **1**

Conformations	⊿G	%
**1a**	4.5527	0.04%
**1b**	5.4409	0.01%
**1c**	0	97.08%
**1d**	3.2483	0.40%
**1e**	8.5621	0.00%
**1f**	4.0613	0.10%
**1g**	5.0649	0.02%
**1h**	5.7444	0.01%
**1i**	6.0319	0.00%
**1j**	8.5141	0.00%
**1k**	9.2623	0.00%
**1l**	6.5735	0.00%
**1m**	2.4691	1.51%
**1n**	4.5459	0.05%
**1o**	3.0793	0.54%
**1p**	3.5966	0.22%
**1q**	7.8883	0.00%
**1r**	5.214	0.01%

Optimized Z-matrixes of compound**1**

**1a**				**1b**			
C	0.120863	2.309146	0.091257	C	0.070024	2.439857	0.272057
C	1.403493	2.640867	-0.11649	C	1.288291	2.909863	-0.0388
C	-2.842647	1.745674	-0.50293	C	-2.900811	1.707838	-0.11311
C	-3.696652	-1.101906	-0.46543	C	-3.55833	-1.141371	-0.59903
C	0.122015	-1.473888	-0.41936	C	0.28903	-1.193291	-0.86172
C	0.713523	-0.138001	1.615701	C	0.857581	-0.295618	1.403309
C	-2.741298	2.428522	-1.64847	C	-2.942787	2.602915	-1.10591
C	-3.99492	1.965383	0.455226	C	-3.982574	1.638517	0.945619
C	-5.030539	0.822507	0.479864	C	-4.905961	0.404441	0.850388
C	-0.381792	0.98598	-0.42727	C	-0.389092	1.149066	-0.35848
C	2.224583	1.651262	-0.94196	C	2.098159	2.057777	-1.015
C	-1.849511	0.666801	-0.07197	C	-1.79903	0.659379	0.03232
C	-2.242166	-0.708236	-0.67245	C	-2.143048	-0.599951	-0.80912
C	-4.490796	-0.604594	0.750242	C	-4.211222	-0.977883	0.778647
C	-1.325577	-1.833106	-0.08101	C	-1.104295	-1.741275	-0.5496
C	2.094864	0.186845	-0.38968	C	2.139069	0.580784	-0.51032
C	2.979955	-0.128984	0.857818	C	3.051934	0.412053	0.747516
C	0.634202	-0.123881	0.06076	C	0.728154	0.054975	-0.10663
C	2.002875	3.923384	0.395607	C	1.833284	4.196679	0.520856

C	3.672045	2.078678	-1.22082	C	3.490002	2.592401	-1.37976
C	4.769797	-1.825745	-1.42719	C	5.453832	-2.367755	-0.61623
C	6.082417	-0.817527	0.498928	C	3.892274	-2.842421	1.300655
C	3.643177	-1.527164	0.893473	C	4.328536	-0.427044	0.535222
C	4.948439	-1.775043	0.098791	C	4.180429	-1.907699	0.113429
N	2.012394	-0.028533	1.953434	N	2.144037	-0.120983	1.756008
O	-4.272867	-1.825204	-1.26478	O	-4.16595	-1.663402	-1.51453
O	0.773973	-2.255147	-1.09663	O	0.950986	-1.732822	-1.73623
O	-0.231878	-0.225421	2.398192	O	-0.040064	-0.690082	2.148384
O	-5.572311	-1.490172	0.979188	O	-5.163226	-2.02409	0.913782
O	-1.637489	-3.107588	-0.57982	O	-1.358241	-2.8851	-1.32651
H	-0.554414	2.950202	0.652316	H	-0.591688	2.96499	0.956029
H	-3.482671	3.174419	-1.92471	H	-3.758128	3.318	-1.18463
H	-1.925317	2.281814	-2.35078	H	-2.173407	2.6662	-1.87067
H	-4.522335	2.890198	0.196853	H	-4.612703	2.532995	0.892793
H	-3.594987	2.100882	1.470262	H	-3.50736	1.643656	1.936956
H	-5.766419	1.023839	1.266178	H	-5.556061	0.394011	1.734583
H	-5.57672	0.7963	-0.47192	H	-5.559964	0.489871	-0.02592
H	-0.298171	0.974873	-1.52605	H	-0.388679	1.286875	-1.45209
H	1.736692	1.603463	-1.92823	H	1.531712	2.027366	-1.95932
H	-1.9004	0.585859	1.018347	H	-1.756928	0.367593	1.085502
H	-2.087158	-0.696603	-1.75838	H	-2.084711	-0.348164	-1.87507
H	-3.856876	-0.600437	1.646369	H	-3.443192	-1.050383	1.563662
H	-1.431343	-1.822893	1.011962	H	-1.131942	-1.999449	0.516917
H	2.366032	-0.490942	-1.19988	H	2.523174	-0.039068	-1.32424
H	3.74309	0.639649	1.001421	H	3.382306	1.401138	1.087278
H	1.26193	4.499625	0.958634	H	2.770786	4.040678	1.07029
H	2.859897	3.737091	1.055606	H	2.056201	4.91973	-0.27474
H	2.371148	4.555749	-0.42264	H	1.11419	4.659152	1.204362
H	3.690152	3.028572	-1.76478	H	3.971937	1.927358	-2.10507
H	4.179502	1.336709	-1.84554	H	4.155218	2.681035	-0.51431
H	4.264662	2.213654	-0.31043	H	3.415503	3.583502	-1.83881
H	5.682587	-2.20302	-1.90357	H	6.333937	-2.272137	0.033513
H	3.943499	-2.487783	-1.71062	H	5.376756	-3.419006	-0.91811
H	4.569409	-0.837258	-1.8546	H	5.63762	-1.770103	-1.51699
H	7.027359	-1.126824	0.036958	H	3.818222	-3.883051	0.962466
H	6.229989	-0.804448	1.585749	H	2.957713	-2.591315	1.81054
H	5.884597	0.211001	0.174726	H	4.703247	-2.790938	2.039384
H	2.895148	-2.27233	0.594575	H	4.934132	-0.375732	1.451856
H	3.871942	-1.730819	1.948969	H	4.906713	0.107145	-0.23038
H	5.262111	-2.783579	0.405793	H	3.34537	-1.991503	-0.5949
H	2.287221	-0.108844	2.925604	H	2.427893	-0.275488	2.71626
H	-5.717794	-1.930003	0.117695	H	-5.559167	-1.931987	1.79528

H	-0.872637	-3.361966	-1.13493	H	-0.615699	-2.935535	-1.96169
**1c**				**1d**			
C	0.065531	2.301654	0.257078	C	-0.035109	2.310458	0.188336
C	1.281586	2.725938	-0.12085	C	1.166538	2.67085	-0.28814
C	-2.956166	1.742282	0.053803	C	-3.030311	1.596016	0.224784
C	-3.775496	-1.083097	-0.40369	C	-3.744187	-1.198532	0.348758
C	0.015461	-1.30627	-0.99588	C	0.070288	-1.512499	-0.10245
C	0.852571	-0.457746	1.190409	C	0.889847	-0.055153	1.744568
C	-3.028719	2.656747	-0.91941	C	-3.276413	2.542923	1.139154
C	-3.958041	1.707172	1.189695	C	-3.893752	1.495592	-1.02234
C	-4.966501	0.54286	1.114419	C	-5.127524	0.585693	-0.80715
C	-0.508357	1.070213	-0.39846	C	-0.565143	0.9391	-0.14207
C	1.957848	1.907242	-1.21904	C	1.868106	1.642794	-1.172
C	-1.903728	0.634906	0.095004	C	-1.940455	0.556384	0.473021
C	-2.373577	-0.592386	-0.73237	C	-2.321141	-0.850747	-0.0717
C	-4.380012	-0.886324	0.992931	C	-4.836634	-0.925419	-0.69265
C	-1.370413	-1.784343	-0.56012	C	-1.308718	-1.906872	0.416586
C	1.95419	0.391278	-0.8427	C	1.921488	0.244728	-0.47057
C	3.005517	0.016037	0.250473	C	3.008477	0.111452	0.643555
C	0.573608	-0.076413	-0.29238	C	0.567713	-0.106685	0.222056
C	1.942222	3.941167	0.472947	C	1.786096	4.012638	-0.00156
C	3.338888	2.399291	-1.67394	C	3.218784	2.078115	-1.75606
C	5.785743	-2.770096	0.264709	C	6.105348	-0.244632	-0.22454
C	5.96873	-0.462502	1.275307	C	5.451665	-2.622043	-0.76032
C	3.976719	-1.083047	-0.21275	C	3.854476	-1.175569	0.55251
C	5.004479	-1.572371	0.82691	C	4.937081	-1.190066	-0.5461
N	2.18282	-0.399871	1.382693	N	2.218108	0.121667	1.872124
O	-4.460384	-1.643785	-1.24682	O	-4.017965	-1.59553	1.46701
O	0.583717	-1.896134	-1.90229	O	0.694188	-2.311878	-0.78681
O	0.018341	-0.777143	2.036875	O	0.086362	-0.164178	2.669557
O	-5.40746	-1.842804	1.1824	O	-4.376831	-1.472059	-1.92944
O	-1.745186	-2.918509	-1.29831	O	-1.647415	-3.199202	-0.03764
H	-0.509489	2.814149	1.024007	H	-0.626403	2.974957	0.811593
H	-3.807958	3.415064	-0.91979	H	-2.729143	2.59357	2.077299
H	-2.323558	2.694595	-1.74525	H	-4.040871	3.300907	0.982379
H	-4.527175	2.643	1.21244	H	-4.259717	2.493739	-1.28483
H	-3.415024	1.642609	2.143433	H	-3.319241	1.134568	-1.884
H	-5.580665	0.543342	2.021718	H	-5.658688	0.911108	0.095179
H	-5.646828	0.695869	0.266681	H	-5.824488	0.714361	-1.64676
H	-0.594632	1.262417	-1.48015	H	-0.679398	0.866675	-1.23644
H	1.309577	1.978633	-2.10683	H	1.209014	1.482826	-2.0401
H	-1.793039	0.319902	1.136908	H	-1.808531	0.469516	1.55433
H	-2.390588	-0.332105	-1.79778	H	-2.29286	-0.83837	-1.16521

H	-3.618196	-1.045349	1.767006	H	-5.744428	-1.440592	-0.35166
H	-1.316788	-2.032912	0.508121	H	-1.286175	-1.88612	1.51462
H	2.178472	-0.173752	-1.75134	H	2.123515	-0.498035	-1.24645
H	3.573955	0.90153	0.550382	H	3.667961	0.983713	0.652737
H	1.309663	4.388899	1.245881	H	2.757196	3.914783	0.500814
H	2.911953	3.698717	0.926566	H	1.133409	4.61224	0.640806
H	2.138197	4.707177	-0.28883	H	1.968235	4.58114	-0.92296
H	3.273953	3.418145	-2.06916	H	3.957442	2.331841	-0.98933
H	3.723614	1.758332	-2.47525	H	3.09403	2.959976	-2.39284
H	4.079955	2.40728	-0.86796	H	3.640332	1.280634	-2.37779
H	5.113058	-3.587066	-0.02196	H	6.609149	-0.554162	0.700411
H	6.35858	-2.479675	-0.6257	H	5.780904	0.79359	-0.09497
H	6.494539	-3.160105	1.004534	H	6.848889	-0.258179	-1.02999
H	6.696283	-0.850954	1.997393	H	5.89962	-3.017834	0.160606
H	5.449252	0.3757	1.753276	H	6.218655	-2.651869	-1.5432
H	6.528653	-0.064319	0.419061	H	4.640418	-3.297904	-1.05521
H	4.515109	-0.699162	-1.09139	H	4.342271	-1.339022	1.524604
H	3.379009	-1.936603	-0.56027	H	3.167562	-2.018316	0.4027
H	4.455651	-1.927475	1.712317	H	4.479082	-0.859434	-1.48991
H	2.567109	-0.610109	2.295461	H	2.643624	0.11252	2.79157
H	-5.679751	-2.095127	0.277438	H	-5.049017	-1.273586	-2.60172
H	-1.057345	-3.017696	-1.98738	H	-0.894928	-3.479276	-0.59673
**1e**				**1f**			
C	-0.069971	2.200567	0.448444	C	-0.069971	2.200567	0.448444
C	1.098749	2.738905	0.068154	C	1.098749	2.738905	0.068154
C	-3.029163	1.540229	0.166614	C	-3.029163	1.540229	0.166614
C	-3.576105	-1.549079	0.082424	C	-3.576105	-1.549079	0.082424
C	0.056867	-1.236295	-1.20146	C	0.056867	-1.236295	-1.20146
C	0.882411	-0.63347	1.111571	C	0.882411	-0.63347	1.111571
C	-3.052822	2.48197	-0.78324	C	-3.052822	2.48197	-0.78324
C	-4.103127	1.523113	1.236611	C	-4.103127	1.523113	1.236611
C	-4.698007	0.145567	1.59851	C	-4.698007	0.145567	1.59851
C	-0.585835	0.97786	-0.27308	C	-0.585835	0.97786	-0.27308
C	1.79917	2.042739	-1.09563	C	1.79917	2.042739	-1.09563
C	-1.946948	0.461058	0.246385	C	-1.946948	0.461058	0.246385
C	-2.381905	-0.820238	-0.55226	C	-2.381905	-0.820238	-0.55226
C	-4.887766	-0.817756	0.417943	C	-4.887766	-0.817756	0.417943
C	-1.245473	-1.866666	-0.74319	C	-1.245473	-1.866666	-0.74319
C	1.906004	0.514923	-0.80475	C	1.906004	0.514923	-0.80475
C	2.980654	0.169487	0.276603	C	2.980654	0.169487	0.276603
C	0.564189	-0.097203	-0.30557	C	0.564189	-0.097203	-0.30557
C	1.68945	3.957054	0.726075	C	1.68945	3.957054	0.726075
C	3.135708	2.652978	-1.54094	C	3.135708	2.652978	-1.54094

C	5.016545	-2.324812	1.477288	C	5.016545	-2.324812	1.477288
C	6.613999	-1.15235	-0.06977	C	6.613999	-1.15235	-0.06977
C	4.136457	-0.674393	-0.2896	C	4.136457	-0.674393	-0.2896
C	5.281934	-1.030589	0.688773	C	5.281934	-1.030589	0.688773
N	2.206598	-0.493513	1.320586	N	2.206598	-0.493513	1.320586
O	-3.510658	-2.745062	0.342667	O	-3.510658	-2.745062	0.342667
O	0.635037	-1.597025	-2.20975	O	0.635037	-1.597025	-2.20975
O	0.084224	-1.117494	1.915118	O	0.084224	-1.117494	1.915118
O	-5.410484	-0.101805	-0.69532	O	-5.410484	-0.101805	-0.69532
O	-1.621537	-2.871939	-1.66478	O	-1.621537	-2.871939	-1.66478
H	-0.653132	2.618676	1.265391	H	-0.653132	2.618676	1.265391
H	-2.29034	2.576089	-1.5496	H	-2.29034	2.576089	-1.5496
H	-3.858128	3.211285	-0.82352	H	-3.858128	3.211285	-0.82352
H	-3.690598	1.951087	2.162315	H	-3.690598	1.951087	2.162315
H	-4.910756	2.190625	0.922165	H	-4.910756	2.190625	0.922165
H	-4.080837	-0.36415	2.348758	H	-4.080837	-0.36415	2.348758
H	-5.67441	0.306541	2.068565	H	-5.67441	0.306541	2.068565
H	-0.729645	1.235699	-1.33432	H	-0.729645	1.235699	-1.33432
H	1.127801	2.12388	-1.96547	H	1.127801	2.12388	-1.96547
H	-1.813585	0.173219	1.294327	H	-1.813585	0.173219	1.294327
H	-2.703591	-0.491653	-1.55026	H	-2.703591	-0.491653	-1.55026
H	-5.573793	-1.617478	0.72506	H	-5.573793	-1.617478	0.72506
H	-1.037932	-2.297667	0.246146	H	-1.037932	-2.297667	0.246146
H	2.177281	0.015563	-1.73879	H	2.177281	0.015563	-1.73879
H	3.404297	1.088273	0.701299	H	3.404297	1.088273	0.701299
H	2.687631	3.756564	1.136619	H	2.687631	3.756564	1.136619
H	1.05152	4.306031	1.544373	H	1.05152	4.306031	1.544373
H	1.807091	4.784046	0.013356	H	1.807091	4.784046	0.013356
H	3.894786	2.643312	-0.7515	H	3.894786	2.643312	-0.7515
H	3.001944	3.693487	-1.85441	H	3.001944	3.693487	-1.85441
H	3.536978	2.099054	-2.39705	H	3.536978	2.099054	-2.39705
H	4.945657	-3.180992	0.794012	H	4.945657	-3.180992	0.794012
H	4.086583	-2.291699	2.053411	H	4.086583	-2.291699	2.053411
H	5.833792	-2.525658	2.179922	H	5.833792	-2.525658	2.179922
H	6.557348	-1.939525	-0.83299	H	6.557348	-1.939525	-0.83299
H	7.434373	-1.407831	0.611324	H	7.434373	-1.407831	0.611324
H	6.8729	-0.214481	-0.57498	H	6.8729	-0.214481	-0.57498
H	3.724882	-1.590419	-0.73622	H	3.724882	-1.590419	-0.73622
H	4.552285	-0.087603	-1.1193	H	4.552285	-0.087603	-1.1193
H	5.386424	-0.203099	1.407724	H	5.386424	-0.203099	1.407724
H	2.594321	-0.728724	2.225228	H	2.594321	-0.728724	2.225228
H	-5.728794	-0.750554	-1.34376	H	-5.728794	-0.750554	-1.34376
H	-2.301294	-3.382295	-1.19086	H	-2.301294	-3.382295	-1.19086

**1g**				**1h**			
C	-0.069971	2.200567	0.448444	C	0.029434	2.243461	0.469034
C	1.098749	2.738905	0.068154	C	1.169417	2.745133	-0.03124
C	-3.029163	1.540229	0.166614	C	-2.924975	1.572528	0.558388
C	-3.576105	-1.549079	0.082424	C	-3.686771	-1.33154	0.094293
C	0.056867	-1.236295	-1.20146	C	0.055623	-1.33433	-0.92269
C	0.882411	-0.63347	1.111571	C	1.019281	-0.5271	1.242666
C	-3.052822	2.48197	-0.78324	C	-3.088613	2.293659	1.674178
C	-4.103127	1.523113	1.236611	C	-3.802573	1.789666	-0.65798
C	-4.698007	0.145567	1.59851	C	-4.949431	0.733348	-0.78382
C	-0.585835	0.97786	-0.27308	C	-0.547051	0.99018	-0.14218
C	1.79917	2.042739	-1.09563	C	1.777642	1.975296	-1.20035
C	-1.946948	0.461058	0.246385	C	-1.872412	0.469711	0.48861
C	-2.381905	-0.820238	-0.55226	C	-2.340977	-0.76654	-0.3429
C	-4.887766	-0.817756	0.417943	C	-4.893509	-0.40277	0.247812
C	-1.245473	-1.866666	-0.74319	C	-1.266229	-1.88701	-0.39927
C	1.906004	0.514923	-0.80475	C	1.89782	0.466281	-0.82828
C	2.980654	0.169487	0.276603	C	3.052935	0.169993	0.182581
C	0.564189	-0.097203	-0.30557	C	0.594238	-0.0974	-0.18686
C	1.68945	3.957054	0.726075	C	1.81257	3.995328	0.50744
C	3.135708	2.652978	-1.54094	C	3.077981	2.547505	-1.78131
C	5.016545	-2.324812	1.477288	C	5.114068	-2.30211	1.390972
C	6.613999	-1.15235	-0.06977	C	6.608405	-1.28719	-0.35722
C	4.136457	-0.674393	-0.2896	C	4.133578	-0.75033	-0.41258
C	5.281934	-1.030589	0.688773	C	5.345929	-1.07355	0.494493
N	2.206598	-0.493513	1.320586	N	2.355881	-0.38891	1.335564
O	-3.510658	-2.745062	0.342667	O	-3.859503	-2.52881	0.305637
O	0.635037	-1.597025	-2.20975	O	0.628387	-1.84264	-1.86737
O	0.084224	-1.117494	1.915118	O	0.282987	-0.94472	2.136987
O	-5.410484	-0.101805	-0.69532	O	-6.086948	-1.15988	0.175547
O	-1.621537	-2.871939	-1.66478	O	-1.65499	-2.95959	-1.23109
H	-0.653132	2.618676	1.265391	H	-0.488445	2.721443	1.294932
H	-2.29034	2.576089	-1.5496	H	-2.502508	2.10746	2.570971
H	-3.858128	3.211285	-0.82352	H	-3.818932	3.097525	1.728528
H	-3.690598	1.951087	2.162315	H	-4.244662	2.78862	-0.60592
H	-4.910756	2.190625	0.922165	H	-3.191354	1.771682	-1.56839
H	-4.080837	-0.36415	2.348758	H	-5.915897	1.22532	-0.63632
H	-5.67441	0.306541	2.068565	H	-4.970859	0.298332	-1.78905
H	-0.729645	1.235699	-1.33432	H	-0.773781	1.202893	-1.20059
H	1.127801	2.12388	-1.96547	H	1.042232	2.010059	-2.02018
H	-1.813585	0.173219	1.294327	H	-1.65021	0.136238	1.50539
H	-2.703591	-0.491653	-1.55026	H	-2.507451	-0.43369	-1.37998
H	-5.573793	-1.617478	0.72506	H	-4.789801	0.045637	1.250807

H	-1.037932	-2.297667	0.246146	H	-1.080326	-2.22188	0.632627
H	2.177281	0.015563	-1.73879	H	2.092694	-0.09012	-1.74881
H	3.404297	1.088273	0.701299	H	3.535073	1.103169	0.499168
H	2.687631	3.756564	1.136619	H	2.835394	3.809498	0.860264
H	1.05152	4.306031	1.544373	H	1.234856	4.400249	1.344338
H	1.807091	4.784046	0.013356	H	1.885863	4.775876	-0.2613
H	3.894786	2.643312	-0.7515	H	3.895147	2.579712	-1.05331
H	3.001944	3.693487	-1.85441	H	2.92279	3.568353	-2.14524
H	3.536978	2.099054	-2.39705	H	3.411404	1.941416	-2.63122
H	4.945657	-3.180992	0.794012	H	4.967374	-3.20053	0.777613
H	4.086583	-2.291699	2.053411	H	4.23504	-2.20207	2.035194
H	5.833792	-2.525658	2.179922	H	5.980698	-2.47854	2.038783
H	6.557348	-1.939525	-0.83299	H	6.471354	-2.12409	-1.05453
H	7.434373	-1.407831	0.611324	H	7.47556	-1.51809	0.272823
H	6.8729	-0.214481	-0.57498	H	6.847217	-0.39509	-0.94803
H	3.724882	-1.590419	-0.73622	H	3.660719	-1.68083	-0.75646
H	4.552285	-0.087603	-1.1193	H	4.495018	-0.23769	-1.31392
H	5.386424	-0.203099	1.407724	H	5.529272	-0.2024	1.142538
H	2.594321	-0.728724	2.225228	H	2.816144	-0.5766	2.217045
H	-5.728794	-0.750554	-1.34376	H	-5.828893	-2.07355	0.404113
H	-2.301294	-3.382295	-1.19086	H	-2.439644	-3.33411	-0.79139
**1i**				**1j**			
C	-0.073928	2.226243	0.511003	C	0.013552	2.266749	-0.35016
C	1.0701	2.772125	0.069419	C	-1.271932	2.625964	-0.22139
C	-3.071584	1.430975	0.43399	C	3.023548	1.804327	0.29073
C	-3.594537	-1.46961	0.19661	C	3.262826	-1.5136	-0.0237
C	0.079556	-1.30206	-0.94218	C	-0.030084	-1.33194	1.04486
C	0.99177	-0.491	1.233489	C	-0.38372	-0.64066	-1.26858
C	-3.101132	2.565734	-0.27315	C	2.898272	2.799039	1.175339
C	-4.22443	1.064169	1.359311	C	4.251379	1.676213	-0.58993
C	-5.369054	0.236015	0.722102	C	5.008078	0.329762	-0.53579
C	-0.601179	0.986068	-0.17245	C	0.530036	1.078992	0.425647
C	1.720908	2.070283	-1.11994	C	-2.098969	1.783751	0.746128
C	-1.920184	0.423967	0.39655	C	1.985792	0.709037	0.084347
C	-2.330638	-0.85774	-0.40899	C	2.451498	-0.58078	0.857946
C	-4.942721	-0.8998	-0.24562	C	4.234987	-0.90315	-1.04577
C	-1.213222	-1.92194	-0.43807	C	1.371783	-1.31693	1.659253
C	1.873124	0.546572	-0.81682	C	-1.950226	0.267815	0.402602
C	3.026482	0.228246	0.188952	C	-2.701302	-0.18534	-0.89549
C	0.577995	-0.06597	-0.20353	C	-0.448661	-0.12992	0.195108
C	1.680859	4.000261	0.689605	C	-1.873924	3.788347	-0.96497
C	3.016054	2.700172	-1.65144	C	-3.557187	2.223375	0.930545
C	5.047729	-2.30368	1.346424	C	-5.947945	-0.34391	-0.87152

C	6.579861	-1.22485	-0.33163	C	-5.604999	-2.46099	0.456503
C	4.103951	-0.68827	-0.41721	C	-3.628286	-1.40313	-0.69759
C	5.299486	-1.04373	0.500232	C	-4.969853	-1.13373	0.012669
N	2.326606	-0.34177	1.335414	N	-1.612839	-0.53357	-1.8057
O	-3.550594	-2.31758	1.071211	O	3.224137	-2.73151	0.081022
O	0.634235	-1.78342	-1.92037	O	-0.787546	-2.25565	1.291332
O	0.249115	-0.90891	2.12042	O	0.616405	-1.08769	-1.83304
O	-4.815183	-0.45383	-1.59299	O	5.157892	-1.90086	-1.44317
O	-1.583735	-3.01081	-1.25981	O	1.231437	-0.61979	2.910437
H	-0.620979	2.64166	1.353045	H	0.69173	2.802846	-1.00987
H	-2.303731	2.868451	-0.94406	H	2.013988	2.925561	1.793345
H	-3.945842	3.246989	-0.19714	H	3.685972	3.537065	1.307073
H	-3.827819	0.504236	2.21553	H	3.946461	1.84372	-1.63491
H	-4.667021	1.97851	1.76915	H	4.960108	2.473601	-0.34149
H	-5.967785	-0.19889	1.530008	H	5.885217	0.416291	-1.18627
H	-6.03608	0.899497	0.157865	H	5.388835	0.1385	0.475523
H	-0.785752	1.221258	-1.23329	H	0.470408	1.293907	1.501944
H	1.000775	2.123632	-1.95157	H	-1.62875	1.890179	1.736565
H	-1.729643	0.105362	1.427585	H	1.981645	0.468671	-0.98031
H	-2.553685	-0.55752	-1.43656	H	3.166684	-0.25559	1.628043
H	-5.672718	-1.71789	-0.1792	H	3.620163	-0.60113	-1.91231
H	-1.045855	-2.26723	0.590591	H	1.680503	-2.3533	1.825335
H	2.084978	0.039731	-1.76204	H	-2.354469	-0.30033	1.244889
H	3.513946	1.153284	0.519064	H	-3.263481	0.647495	-1.32996
H	2.695751	3.808328	1.061516	H	-2.719008	3.478017	-1.59325
H	1.074965	4.354295	1.529726	H	-1.129404	4.262723	-1.61226
H	1.765162	4.820259	-0.03562	H	-2.261195	4.553224	-0.27901
H	3.817638	2.729502	-0.90609	H	-4.137474	2.18272	0.003637
H	2.837771	3.728829	-1.9813	H	-3.603004	3.2529	1.300385
H	3.383601	2.135377	-2.51561	H	-4.057272	1.586877	1.668472
H	4.922027	-3.1804	0.697958	H	-6.202896	-0.91808	-1.77192
H	4.149298	-2.22751	1.96689	H	-5.536743	0.61773	-1.19763
H	5.896028	-2.50047	2.01241	H	-6.880216	-0.13708	-0.33301
H	6.458621	-2.03383	-1.06393	H	-5.800162	-3.1092	-0.40782
H	7.433274	-1.48018	0.307681	H	-6.56031	-2.28949	0.966461
H	6.830832	-0.31039	-0.88184	H	-4.948148	-3.00781	1.143034
H	3.627597	-1.60619	-0.78883	H	-3.842767	-1.83873	-1.6848
H	4.481202	-0.15734	-1.30137	H	-3.057245	-2.15648	-0.14056
H	5.468899	-0.19704	1.183685	H	-4.772689	-0.54374	0.919226
H	2.781802	-0.51983	2.221673	H	-1.789278	-0.90815	-2.73092
H	-5.66782	-0.07129	-1.85483	H	4.751944	-2.74605	-1.16426
H	-0.907989	-3.03754	-1.96659	H	0.664158	-1.16378	3.483054
**1k**				**1l**			

C	-0.186888	2.104663	-0.73842	C	0.096246	2.126275	0.476862
C	-1.440994	2.507225	-0.48341	C	1.26369	2.591613	0.005623
C	2.913242	1.819014	-0.54872	C	-2.949862	1.549453	0.49174
C	3.248421	-1.43333	0.163425	C	-3.688669	-1.30418	0.25323
C	0.171943	-0.84186	1.719393	C	-0.051222	-1.38565	-1.00961
C	-0.540145	-1.02973	-0.59575	C	0.978768	-0.679	1.147442
C	2.887825	3.045577	-0.01737	C	-2.921293	2.687354	-0.2103
C	3.98813	1.403162	-1.53487	C	-4.092782	1.259998	1.455706
C	4.799581	0.135481	-1.18511	C	-5.31561	0.521549	0.854911
C	0.514512	1.193273	0.240939	C	-0.538443	0.932916	-0.19674
C	-2.066352	1.969285	0.802315	C	1.831215	1.852255	-1.20438
C	1.90707	0.72399	-0.22056	C	-1.876848	0.462306	0.410261
C	2.55333	-0.25804	0.828099	C	-2.405501	-0.7787	-0.39174
C	4.009513	-1.18845	-1.14909	C	-5.006698	-0.63301	-0.1351
C	1.66838	-0.68249	2.006279	C	-1.368731	-1.91874	-0.4708
C	-1.894988	0.422999	0.850259	C	1.879739	0.319237	-0.91239
C	-2.796193	-0.32682	-0.18881	C	3.035688	-0.0879	0.061579
C	-0.425196	-0.01907	0.575052	C	0.559519	-0.20155	-0.27028
C	-2.197376	3.440706	-1.39084	C	1.975019	3.77133	0.613059
C	-3.511839	2.406093	1.079311	C	3.153394	2.392982	-1.76581
C	-5.909987	-0.63333	-1.01026	C	6.254223	-0.16841	0.25442
C	-4.569017	-2.73048	-1.47021	C	5.171896	-2.06046	1.542837
C	-3.898224	-1.1649	0.48822	C	4.011922	-1.09935	-0.57079
C	-5.034914	-1.73627	-0.3931	C	5.339707	-1.40076	0.163596
N	-1.836851	-1.13667	-0.93543	N	2.320833	-0.6136	1.221656
O	3.295686	-2.55007	0.659563	O	-3.676406	-2.16431	1.116949
O	-0.504183	-1.5611	2.434938	O	0.433532	-1.88786	-2.01417
O	0.390257	-1.66467	-1.09671	O	0.23244	-1.07197	2.043512
O	4.898494	-2.26944	-1.36442	O	-4.895589	-0.18366	-1.48277
O	1.75321	0.365856	2.989984	O	-1.842751	-2.96576	-1.29393
H	0.345773	2.434458	-1.62718	H	-0.396701	2.57562	1.334748
H	2.109922	3.371507	0.667158	H	-2.127045	2.934987	-0.90711
H	3.652676	3.779468	-0.26018	H	-3.711094	3.42782	-0.1035
H	3.512453	1.233285	-2.5136	H	-3.707257	0.665976	2.293779
H	4.693216	2.230154	-1.67231	H	-4.453462	2.200414	1.886265
H	5.558746	0.003628	-1.96385	H	-5.915236	0.123561	1.68095
H	5.343162	0.270711	-0.24162	H	-5.952232	1.236471	0.319012
H	0.631844	1.727854	1.194817	H	-0.738471	1.188948	-1.25006
H	-1.464726	2.356505	1.640308	H	1.095492	1.962781	-2.01664
H	1.739179	0.159766	-1.13992	H	-1.677444	0.120175	1.432094
H	3.376461	0.288431	1.312489	H	-2.642079	-0.45198	-1.40815
H	3.249575	-1.16912	-1.94996	H	-5.791598	-1.39653	-0.04818
H	2.037542	-1.62718	2.417235	H	-1.193384	-2.29063	0.546846

H	-2.175116	0.077916	1.849737	H	2.027369	-0.19607	-1.86514
H	-3.252019	0.394497	-0.87685	H	3.589745	0.801955	0.377791
H	-3.136988	2.997777	-1.74542	H	2.984371	3.508835	0.955653
H	-1.595989	3.704027	-2.26678	H	1.418042	4.161032	1.470968
H	-2.468286	4.371888	-0.87579	H	2.094924	4.587976	-0.11101
H	-4.212484	2.07392	0.30609	H	3.973202	2.364203	-1.04077
H	-3.583466	3.496756	1.142088	H	3.038156	3.432861	-2.08841
H	-3.853478	1.997493	2.036926	H	3.460368	1.808318	-2.64042
H	-5.353836	-0.03271	-1.74097	H	5.833637	0.603461	0.91107
H	-6.297272	0.046174	-0.24176	H	6.416548	0.28197	-0.73216
H	-6.768033	-1.06948	-1.53523	H	7.233654	-0.44317	0.663194
H	-4.062711	-2.22499	-2.30339	H	4.832933	-1.34354	2.30217
H	-5.428838	-3.25476	-1.90287	H	6.131977	-2.4546	1.895653
H	-3.890171	-3.48764	-1.05993	H	4.458822	-2.89239	1.51256
H	-3.405552	-1.98144	1.03298	H	3.464093	-2.03647	-0.73902
H	-4.364585	-0.52512	1.248888	H	4.270279	-0.71017	-1.56484
H	-5.672202	-2.30102	0.302614	H	5.851609	-2.13451	-0.47583
H	-2.111019	-1.78089	-1.66564	H	2.786487	-0.88904	2.076377
H	4.589907	-2.97393	-0.75976	H	-5.726565	0.26324	-1.70998
H	1.307555	0.043922	3.792205	H	-1.196979	-3.02532	-2.02626
**1m**				**1n**			
C	0.04052	2.301055	0.119151	C	0.130264	2.144955	0.408796
C	1.312767	2.675856	-0.07831	C	1.248342	2.56151	-0.20714
C	-2.888309	1.738124	-0.55043	C	-2.864143	1.554172	0.841268
C	-3.688311	-1.21365	-0.22638	C	-3.78368	-1.15277	0.392259
C	0.133685	-1.43203	-0.72096	C	-0.121763	-1.49138	-0.77271
C	0.614215	-0.31786	1.490059	C	1.053779	-0.63357	1.244681
C	-2.782424	2.396408	-1.70999	C	-2.872668	2.251558	1.984558
C	-4.044002	1.989401	0.391323	C	-3.924477	1.81096	-0.21716
C	-5.058015	0.832938	0.457182	C	-5.178731	0.92513	-0.01881
C	-0.439729	0.993482	-0.46179	C	-0.550918	0.898977	-0.09685
C	2.152529	1.736251	-0.93953	C	1.720014	1.704833	-1.38014
C	-1.901053	0.656381	-0.10236	C	-1.843225	0.438306	0.63489
C	-2.322434	-0.71433	-0.69148	C	-2.422376	-0.76174	-0.17088
C	-4.491003	-0.53983	0.889881	C	-5.001903	-0.57409	-0.33798
C	-1.298917	-1.8749	-0.41761	C	-1.440452	-1.95223	-0.15878
C	2.052413	0.264576	-0.41971	C	1.806306	0.210592	-0.94066
C	2.903072	-0.04317	0.854037	C	3.027289	-0.08402	-0.00638
C	0.588176	-0.12917	-0.04994	C	0.534496	-0.25172	-0.16981
C	1.882917	3.951163	0.482325	C	1.994255	3.801373	0.207945
C	3.59497	2.187556	-1.204	C	2.988799	2.182726	-2.10026
C	6.109127	-0.36081	0.539633	C	6.246522	-0.06012	0.044782
C	5.568487	-2.63235	-0.41672	C	5.298035	-1.916	1.482823

C	3.751998	-1.3274	0.749687	C	4.001256	-1.11017	-0.61708
C	5.02311	-1.22716	-0.11781	C	5.373611	-1.32512	0.064806
N	1.890444	-0.18565	1.897221	N	2.396137	-0.54078	1.228577
O	-4.16151	-2.23572	-0.72282	O	-3.90737	-1.81846	1.404539
O	0.850228	-2.08115	-1.45829	O	0.324865	-2.09427	-1.73836
O	-0.35024	-0.54986	2.220794	O	0.374613	-0.98615	2.208472
O	-5.552958	-1.38982	1.273251	O	-4.793923	-0.79938	-1.73293
O	-1.579616	-3.01862	-1.18952	O	-1.953419	-3.051	-0.88143
H	-0.648439	2.902232	0.707768	H	-0.297895	2.686717	1.247244
H	-1.970978	2.229497	-2.41277	H	-2.17665	2.040834	2.792979
H	-3.518899	3.142041	-1.99949	H	-3.582329	3.058444	2.154161
H	-3.655408	2.17322	1.403508	H	-4.244399	2.856374	-0.15451
H	-4.58116	2.89453	0.088031	H	-3.529828	1.662973	-1.22963
H	-5.830157	1.077716	1.194393	H	-5.527159	1.025535	1.016005
H	-5.563644	0.716746	-0.5096	H	-5.98914	1.29605	-0.66138
H	-0.367123	1.042872	-1.56029	H	-0.838688	1.078477	-1.14609
H	1.665778	1.707442	-1.92734	H	0.918359	1.733984	-2.13534
H	-1.944933	0.5842	0.987774	H	-1.556281	0.0689	1.622335
H	-2.385898	-0.62755	-1.78414	H	-2.570109	-0.46171	-1.21255
H	-3.82077	-0.39739	1.7516	H	-5.88363	-1.12277	0.01879
H	-1.321734	-2.09408	0.662357	H	-1.256398	-2.2398	0.884943
H	2.391067	-0.38659	-1.2293	H	1.901819	-0.39909	-1.8429
H	3.545394	0.803958	1.11116	H	3.565797	0.845373	0.207273
H	2.746678	3.761091	1.132517	H	3.034249	3.581201	0.481485
H	1.130711	4.488113	1.068887	H	1.512267	4.276238	1.068453
H	2.233521	4.622532	-0.31247	H	2.036181	4.538276	-0.60492
H	4.181828	2.30948	-0.28816	H	3.866067	2.21711	-1.44651
H	3.606636	3.148242	-1.72914	H	2.845666	3.187668	-2.51059
H	4.111666	1.459626	-1.83903	H	3.222361	1.51479	-2.937
H	6.425501	-0.79786	1.495781	H	5.82999	0.732596	0.678589
H	5.767187	0.660731	0.738169	H	6.347668	0.339845	-0.97113
H	6.993658	-0.29151	-0.10429	H	7.252623	-0.28022	0.420552
H	5.832271	-3.15449	0.51239	H	4.972256	-1.17265	2.222177
H	6.470575	-2.58124	-1.0377	H	6.288034	-2.25917	1.804916
H	4.827883	-3.24369	-0.94546	H	4.614973	-2.77201	1.531776
H	4.046464	-1.63251	1.764559	H	3.474269	-2.07093	-0.69357
H	3.10418	-2.12432	0.361383	H	4.196793	-0.78321	-1.64728
H	4.752552	-0.76688	-1.07937	H	5.879962	-2.07479	-0.56044
H	2.130637	-0.34603	2.868442	H	2.919636	-0.75649	2.066828
H	-5.569137	-2.1059	0.607499	H	-5.548108	-0.41055	-2.20531
H	-2.548993	-3.13646	-1.16328	H	-1.321245	-3.19159	-1.61474
**1o**				**1p**			
C	0.044328	2.299065	0.405619	C	0.287481	2.154449	0.328073

C	1.262417	2.770841	0.099351	C	1.521965	2.510675	-0.05814
C	-2.950319	1.763313	0.064586	C	-2.756833	1.907677	0.20112
C	-3.658621	-1.21627	-0.28513	C	-3.776436	-0.98111	-0.18617
C	0.061242	-1.11758	-1.30974	C	-0.133934	-1.19198	-1.4476
C	0.788164	-0.60745	1.04838	C	0.731263	-0.87218	0.902357
C	-3.001029	2.7069	-0.88192	C	-2.771395	2.88319	-0.71377
C	-3.979645	1.706389	1.171515	C	-3.71838	1.912339	1.36841
C	-4.95575	0.521376	1.061313	C	-4.814771	0.83503	1.281636
C	-0.505032	1.125021	-0.36884	C	-0.419445	1.043545	-0.40894
C	1.980906	2.055505	-1.04177	C	2.106979	1.718874	-1.22609
C	-1.898543	0.650796	0.089805	C	-1.822527	0.697294	0.127315
C	-2.379387	-0.5438	-0.77693	C	-2.474346	-0.41101	-0.74277
C	-4.313737	-0.88628	1.058516	C	-4.319175	-0.62817	1.201104
C	-1.316799	-1.68708	-0.96851	C	-1.543943	-1.63918	-1.05969
C	1.980442	0.517543	-0.78464	C	1.977619	0.194891	-0.92789
C	2.973715	0.077577	0.339873	C	2.949536	-0.28405	0.201766
C	0.579017	-0.0203	-0.37049	C	0.541013	-0.20764	-0.48322
C	1.889515	3.93812	0.813253	C	2.292426	3.621932	0.603944
C	3.373212	2.59061	-1.40435	C	3.523573	2.116675	-1.66241
C	4.734653	-2.60845	1.577187	C	6.103984	-0.36951	0.90195
C	6.49756	-1.53267	0.142917	C	4.836215	-2.31209	1.914443
C	4.080796	-0.85309	-0.1862	C	4.026448	-1.2572	-0.3148
C	5.142345	-1.3221	0.838525	C	5.221111	-1.59375	0.61007
N	2.100993	-0.53085	1.338293	N	2.047949	-0.88659	1.179402
O	-4.178104	-2.10362	-0.96187	O	-4.411304	-1.80303	-0.84695
O	0.685829	-1.51224	-2.27498	O	0.392893	-1.58154	-2.4713
O	-0.077223	-1.07904	1.787807	O	-0.151705	-1.33147	1.630065
O	-5.299888	-1.85744	1.343142	O	-5.381018	-1.50467	1.519333
O	-1.689587	-2.58116	-1.99078	O	-2.061985	-2.42499	-2.10805
H	-0.556218	2.737447	1.199045	H	-0.220412	2.652178	1.150409
H	-2.28172	2.769417	-1.69359	H	-2.10004	2.90278	-1.56769
H	-3.778859	3.466497	-0.8736	H	-3.46847	3.713812	-0.63493
H	-3.465657	1.647469	2.142036	H	-3.156819	1.765847	2.302635
H	-4.567787	2.630422	1.179847	H	-4.208093	2.889331	1.442291
H	-5.630232	0.534898	1.92417	H	-5.43507	0.885843	2.182978
H	-5.580298	0.626361	0.165363	H	-5.474581	1.032884	0.427654
H	-0.589834	1.418137	-1.4281	H	-0.537637	1.353234	-1.46037
H	1.36199	2.194954	-1.94256	H	1.457965	1.902566	-2.09731
H	-1.793389	0.304672	1.121374	H	-1.689716	0.306086	1.139336
H	-2.598105	-0.17688	-1.78841	H	-2.725107	0.020638	-1.72083
H	-3.536276	-0.92975	1.836756	H	-3.505679	-0.77298	1.928869
H	-1.196874	-2.20011	-0.0007	H	-1.428136	-2.22072	-0.13166
H	2.270138	0.025199	-1.71649	H	2.210798	-0.35142	-1.84601

H	3.451441	0.953553	0.795695	H	3.433155	0.582273	0.667399
H	2.850631	3.669248	1.270238	H	3.253909	3.273657	1.002923
H	1.231256	4.310092	1.604821	H	1.719484	4.053222	1.43088
H	2.094187	4.769276	0.125544	H	2.523492	4.429159	-0.1036
H	4.088304	2.517743	-0.57841	H	4.267927	1.976185	-0.87197
H	3.31951	3.643375	-1.70016	H	3.556406	3.169326	-1.9616
H	3.783428	2.029824	-2.25167	H	3.8357	1.518116	-2.52559
H	4.630241	-3.4401	0.868462	H	5.577796	0.377157	1.509491
H	3.782366	-2.51303	2.10809	H	6.429237	0.118898	-0.02431
H	5.496294	-2.891	2.31332	H	7.001479	-0.66502	1.457996
H	6.417781	-2.29552	-0.64263	H	4.366891	-1.63007	2.63586
H	7.259938	-1.86781	0.856076	H	5.729831	-2.71446	2.405433
H	6.854295	-0.60731	-0.32428	H	4.150795	-3.1484	1.732826
H	3.616076	-1.72269	-0.67163	H	3.522817	-2.18629	-0.6148
H	4.585511	-0.29053	-0.98293	H	4.43862	-0.81985	-1.23383
H	5.274808	-0.52261	1.584076	H	5.832838	-2.29995	0.030267
H	2.429226	-0.82954	2.247687	H	2.368367	-1.28346	2.052966
H	-5.39366	-2.38266	0.523567	H	-5.55714	-2.01018	0.700841
H	-2.640386	-2.76313	-1.85956	H	-3.011852	-2.54086	-1.91129
**1q**				**1r**			
C	-0.074991	2.230158	0.238971	C	-0.049883	2.263865	0.283223
C	1.163431	2.670247	-0.02669	C	1.175403	2.684054	-0.06588
C	-2.977408	1.556426	-0.38535	C	-3.038631	1.533682	-0.06967
C	-3.632841	-1.45698	0.173432	C	-3.667671	-1.33574	0.254032
C	0.149096	-1.48437	-0.629	C	0.12528	-1.49312	-0.37998
C	0.710491	-0.34716	1.560116	C	0.809328	-0.21846	1.65862
C	-2.845303	2.238343	-1.52882	C	-2.92328	2.48527	-1.00219
C	-4.17509	1.807134	0.509255	C	-4.314788	1.403645	0.751103
C	-4.868558	0.558303	1.095883	C	-5.408828	0.477885	0.16114
C	-0.521238	0.90148	-0.32188	C	-0.535389	0.926429	-0.22232
C	1.998764	1.779542	-0.94223	C	1.951355	1.761386	-1.00265
C	-1.960657	0.516166	0.091202	C	-1.942003	0.520671	0.26536
C	-2.335472	-0.91101	-0.44324	C	-2.306295	-0.89968	-0.28939
C	-4.944384	-0.65187	0.1537	C	-4.917096	-0.84707	-0.47952
C	-1.226008	-1.97716	-0.2174	C	-1.247151	-1.96014	0.076136
C	2.00053	0.301273	-0.43061	C	1.977661	0.299242	-0.44482
C	2.941488	0.027811	0.785982	C	2.992204	0.060763	0.719261
C	0.583167	-0.1657	0.025926	C	0.588895	-0.13139	0.121103
C	1.70018	3.969075	0.512881	C	1.756232	3.987832	0.412478
C	3.39956	2.304853	-1.28328	C	3.331704	2.26692	-1.44276
C	6.132555	-0.14968	0.266144	C	6.137365	-0.20889	0.006267
C	5.629858	-2.44068	-0.66482	C	5.533945	-2.52935	-0.78047
C	3.837275	-1.21786	0.622255	C	3.850849	-1.21078	0.558936

C	5.044777	-1.06124	-0.32381	C	4.997122	-1.12363	-0.46902
N	2.003043	-0.16047	1.888974	N	2.124909	-0.05022	1.889461
O	-3.653422	-2.55972	0.708264	O	-3.780444	-1.9721	1.287584
O	0.842464	-2.08953	-1.42594	O	0.771435	-2.19538	-1.1461
O	-0.197557	-0.61066	2.349646	O	-0.052649	-0.41005	2.515099
O	-5.290748	-0.20864	-1.1531	O	-4.594102	-0.70241	-1.85978
O	-1.51239	-3.1759	-0.91147	O	-1.560344	-3.20402	-0.51735
H	-0.758247	2.792048	0.871509	H	-0.689535	2.844411	0.942656
H	-1.991997	2.134208	-2.19135	H	-2.033507	2.617507	-1.60919
H	-3.6089	2.943934	-1.84684	H	-3.736616	3.183139	-1.18878
H	-3.862354	2.433575	1.357775	H	-4.055777	1.0386	1.752932
H	-4.907766	2.393272	-0.05326	H	-4.760112	2.394501	0.891158
H	-4.374846	0.232429	2.019788	H	-6.12161	0.238016	0.957788
H	-5.890082	0.834253	1.379635	H	-5.970577	1.015225	-0.61302
H	-0.501129	0.952539	-1.42158	H	-0.567249	0.949179	-1.32371
H	1.459781	1.733321	-1.90205	H	1.35677	1.687797	-1.92685
H	-1.984592	0.477421	1.185331	H	-1.900083	0.433291	1.357131
H	-2.506621	-0.82706	-1.52529	H	-2.375981	-0.83515	-1.37878
H	-5.696521	-1.35088	0.541333	H	-5.688938	-1.61686	-0.34398
H	-1.167518	-2.15273	0.866241	H	-1.227146	-2.05991	1.169222
H	2.318699	-0.32884	-1.26498	H	2.238031	-0.36059	-1.27614
H	3.561951	0.901071	1.006512	H	3.64283	0.929057	0.856116
H	2.604709	3.817827	1.116304	H	2.690369	3.837803	0.969132
H	0.954768	4.466182	1.141815	H	1.052437	4.50889	1.069243
H	1.976369	4.660878	-0.29369	H	1.996045	4.656702	-0.42438
H	4.030318	2.450788	-0.40058	H	4.010951	2.44948	-0.60429
H	3.333282	3.26819	-1.79949	H	3.236559	3.207441	-1.99526
H	3.915733	1.608085	-1.95281	H	3.809359	1.541079	-2.11008
H	6.532764	-0.57667	1.19509	H	6.587275	-0.59982	0.928263
H	5.759204	0.854225	0.495809	H	5.798065	0.812765	0.209106
H	6.967452	-0.03672	-0.43534	H	6.928008	-0.1481	-0.75067
H	5.977277	-2.95307	0.241905	H	5.928388	-3.00682	0.126047
H	6.485632	-2.3487	-1.34392	H	6.346648	-2.48691	-1.51515
H	4.882942	-3.08252	-1.14649	H	4.746739	-3.1767	-1.18423
H	4.208596	-1.51124	1.61521	H	4.280189	-1.46492	1.539044
H	3.200946	-2.04171	0.272793	H	3.178809	-2.03596	0.290064
H	4.693099	-0.61095	-1.26379	H	4.594134	-0.70977	-1.40523
H	2.308576	-0.30227	2.844448	H	2.489773	-0.13533	2.830703
H	-5.552547	-0.98592	-1.67264	H	-5.383905	-0.36246	-2.30979
H	-2.272384	-3.54877	-0.43117	H	-0.803834	-3.40274	-1.10483

HRESIMS for compound **1**

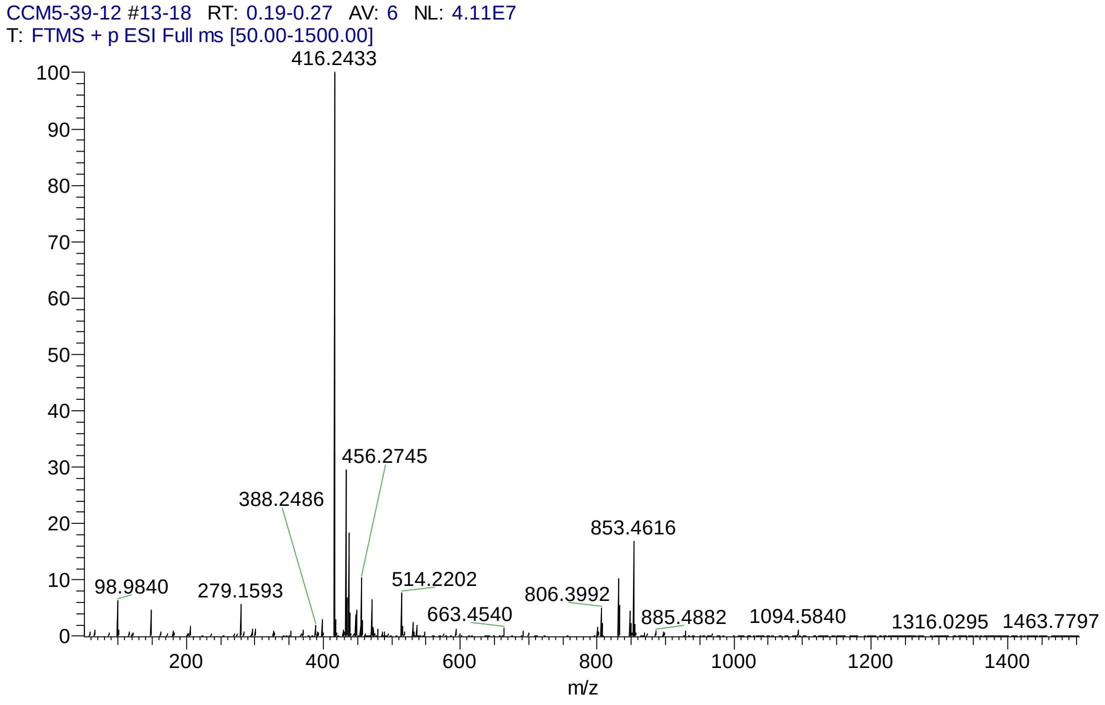

UV spectrum for compound **1**

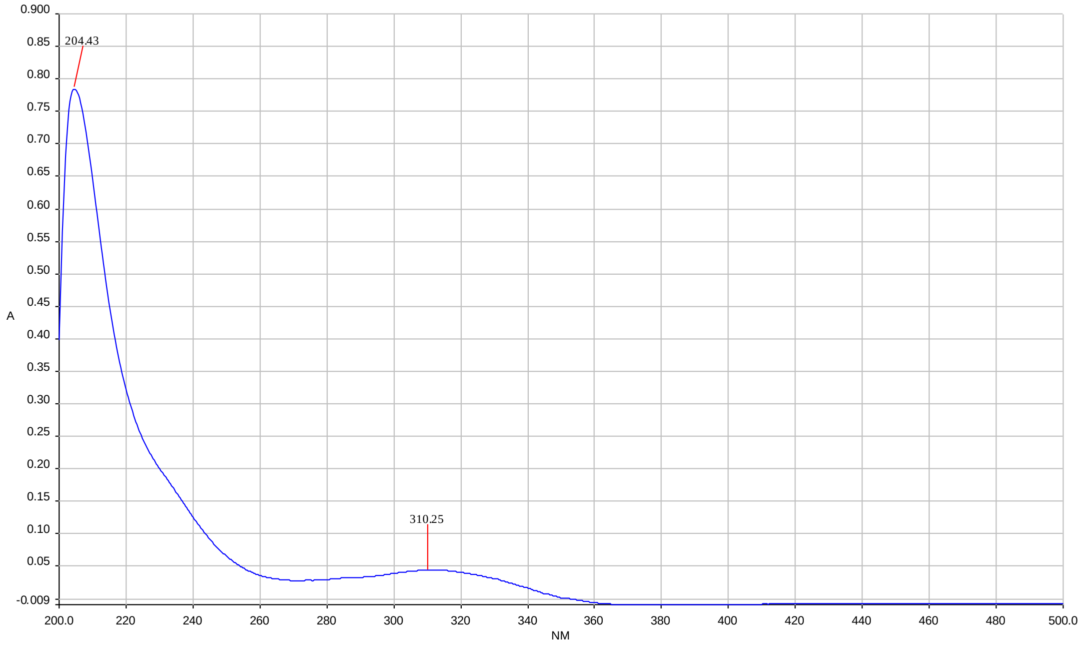

IR spectrum for compound **1**

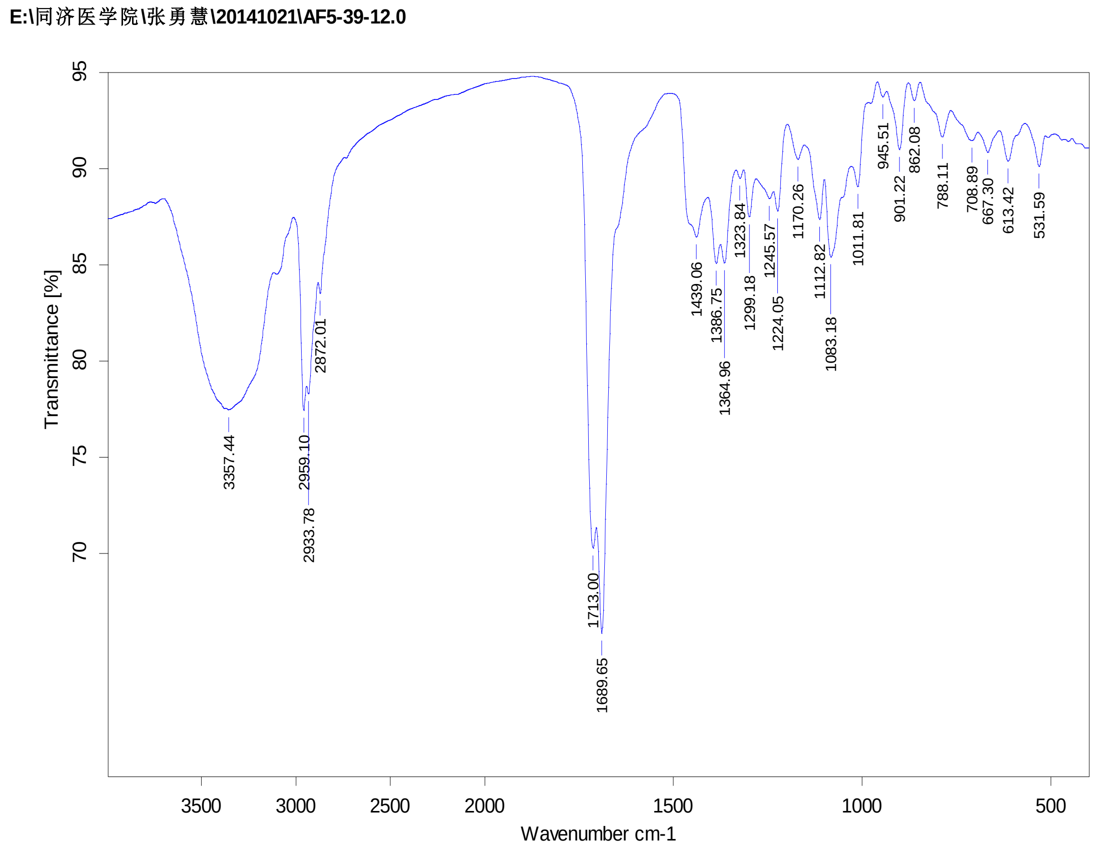

^{1}H NMR for compound **1** (in CD_3OD, 400 MHz)

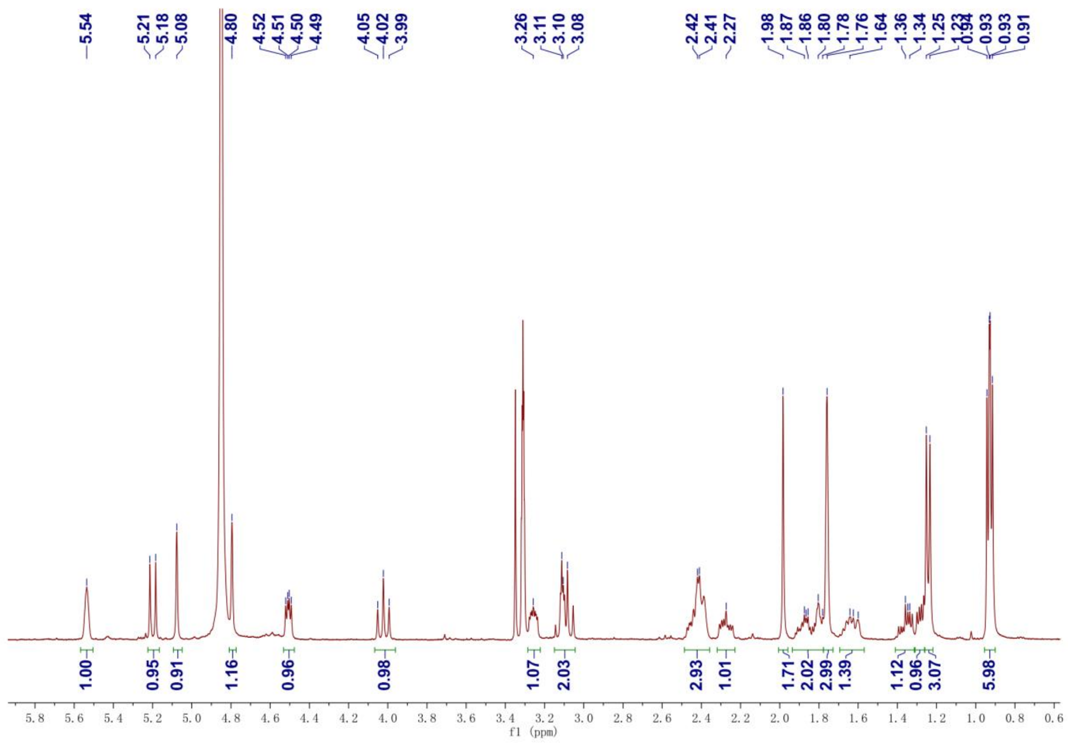

^{13}C NMR for compound **1** (in CD_3OD, 100 MHz)

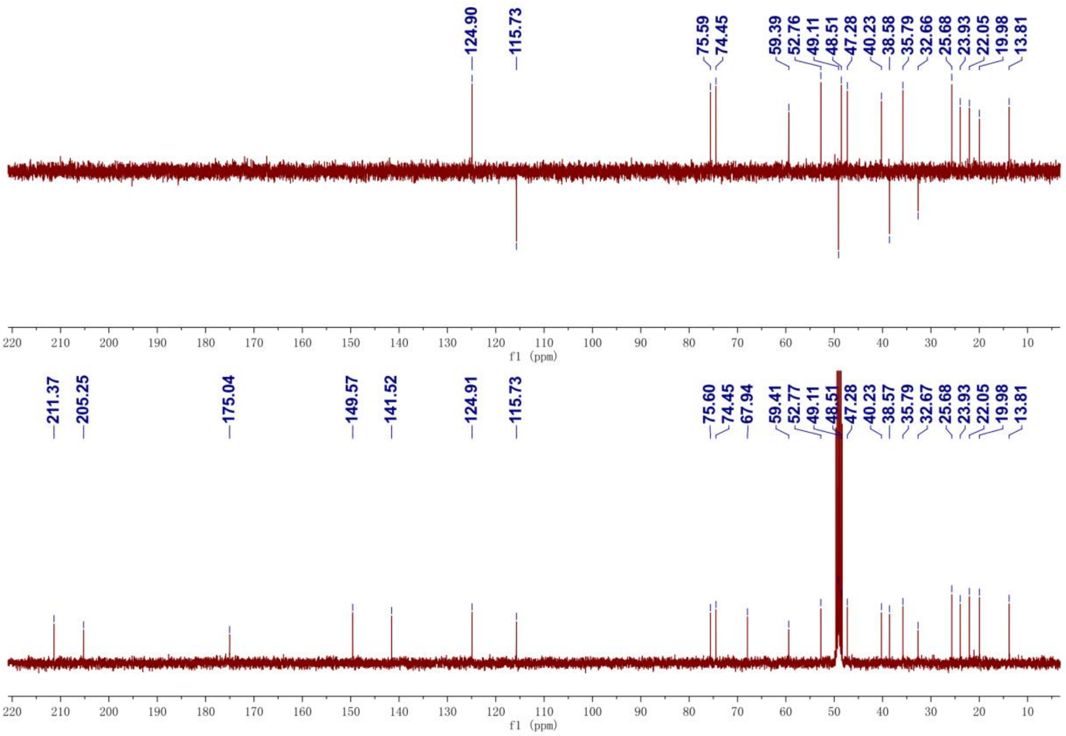

HSQC for compound **1** (in CD_3OD, 400 MHz)

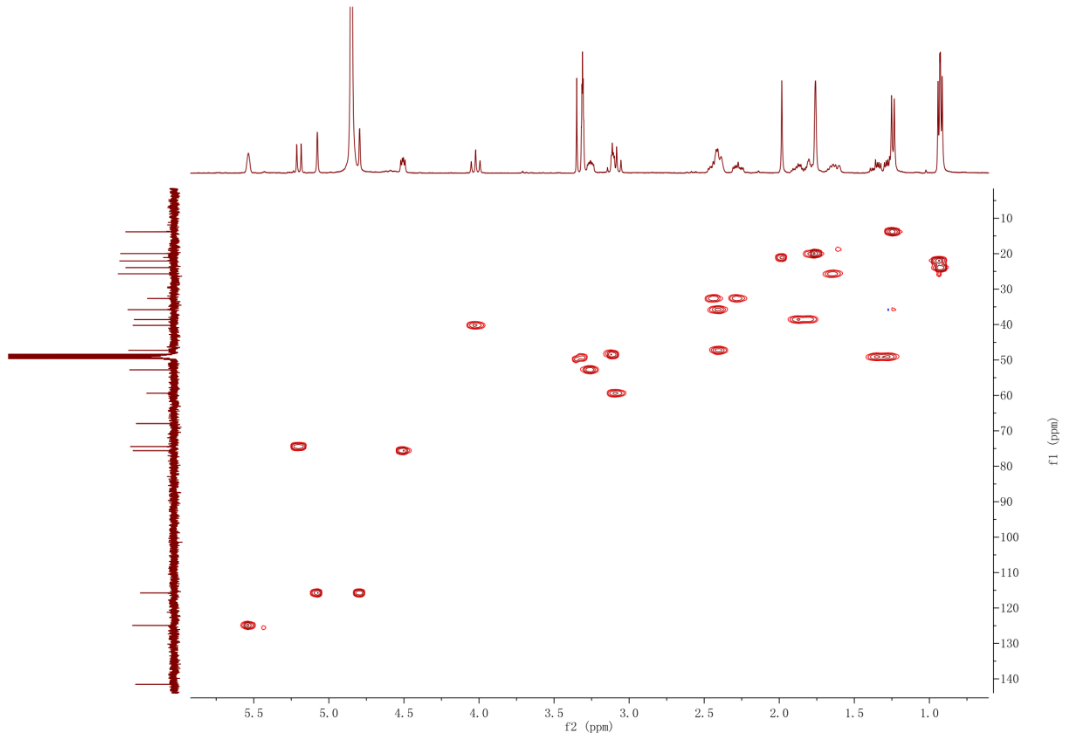

HMBC for compound **1** (in CD_3OD, 400 MHz)

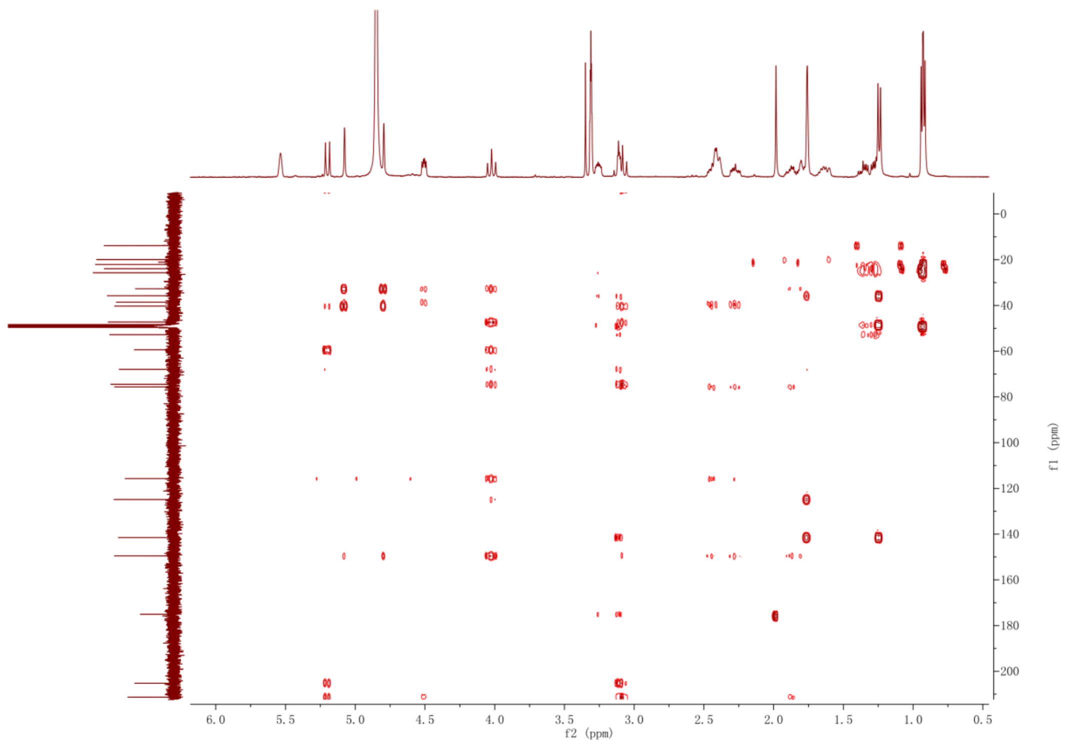

^{1}H–^{1}H COSY for compound **1** (in CD_3OD, 400 MHz)

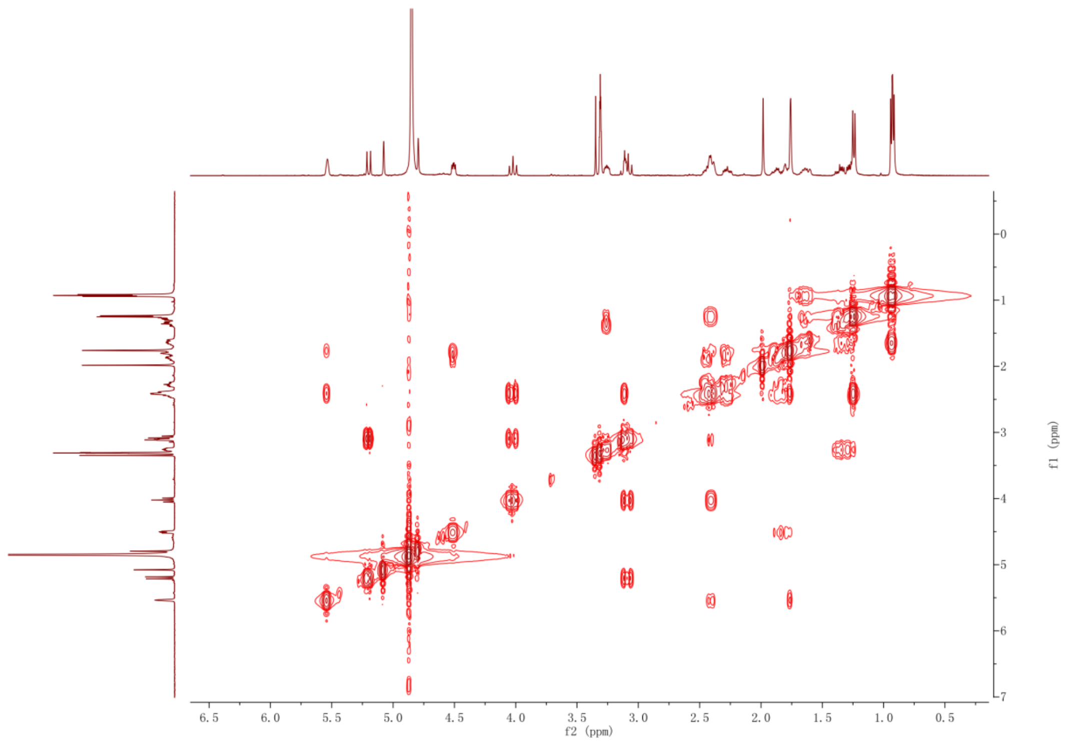

NOESY for compound **1** (in CD_3OD, 400 MHz)

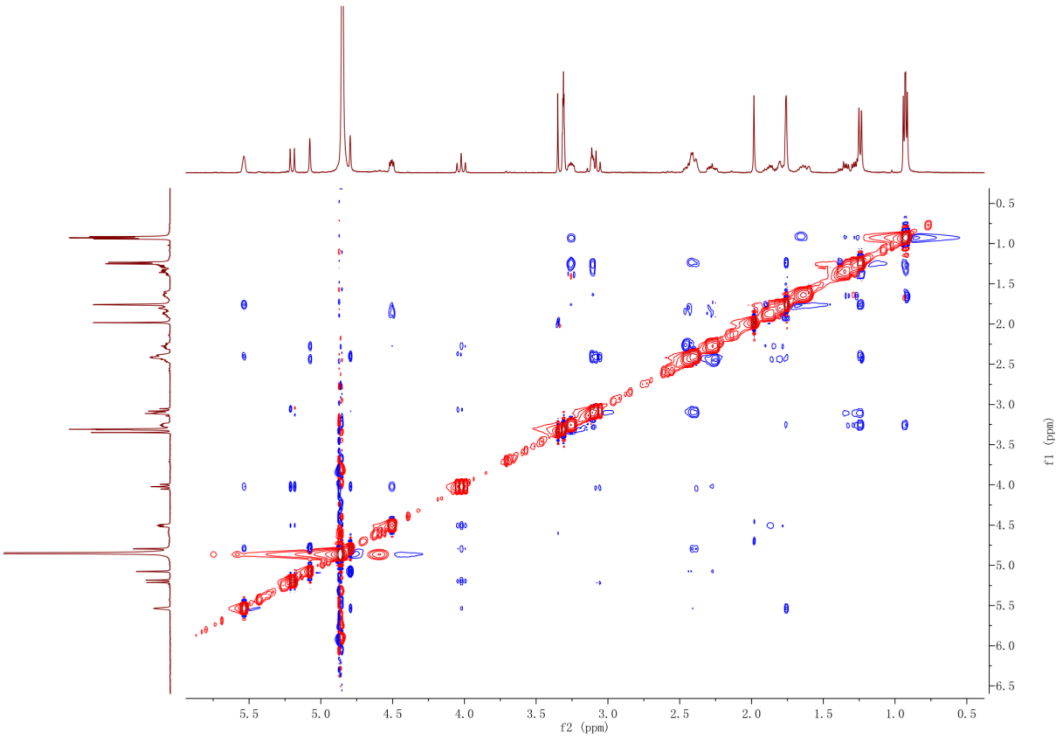

HRESIMS for compound **2**

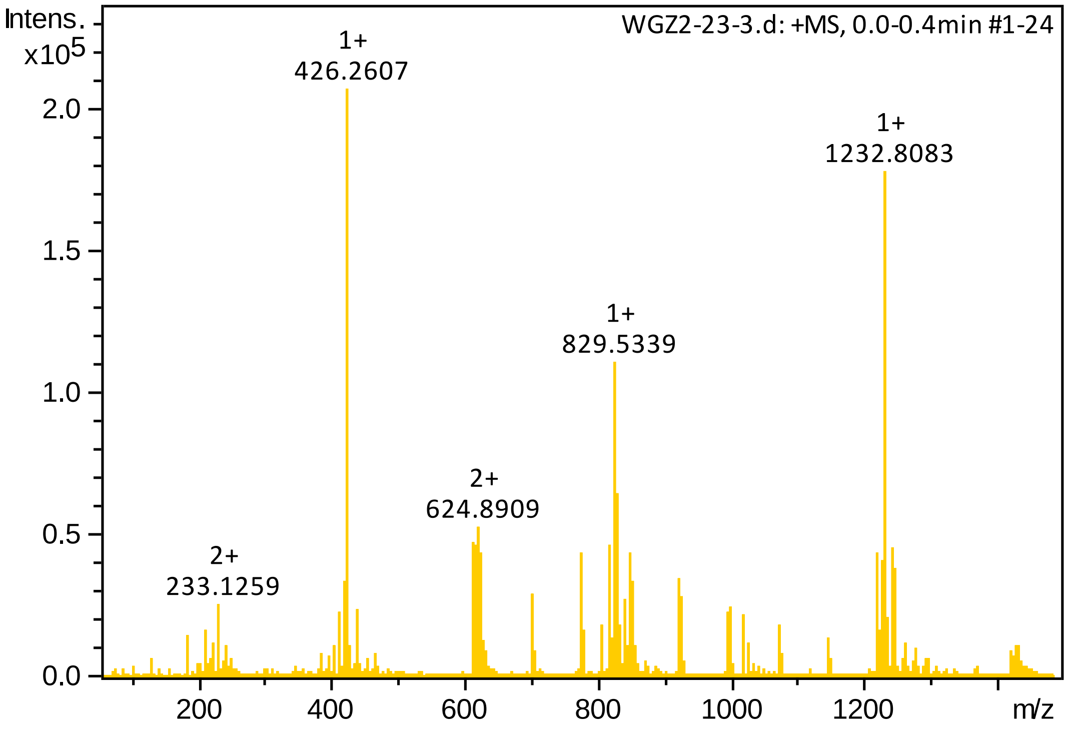

UV spectrum for compound **2**

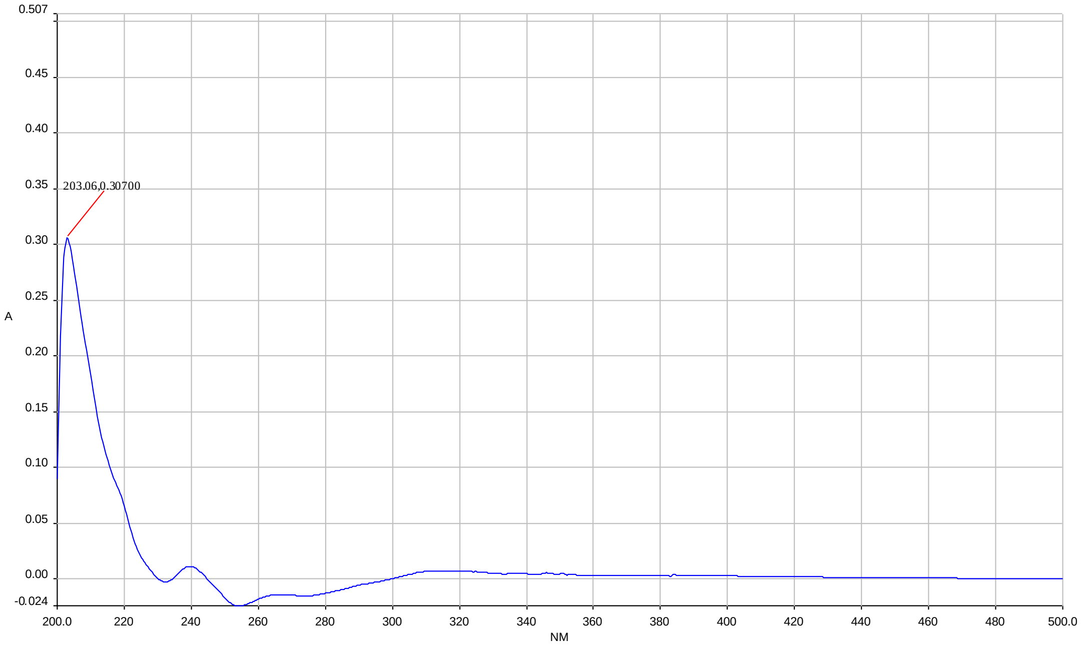

IR spectrum for compound **2**

E:\20160421\20160421魏广征\2-32-2.0

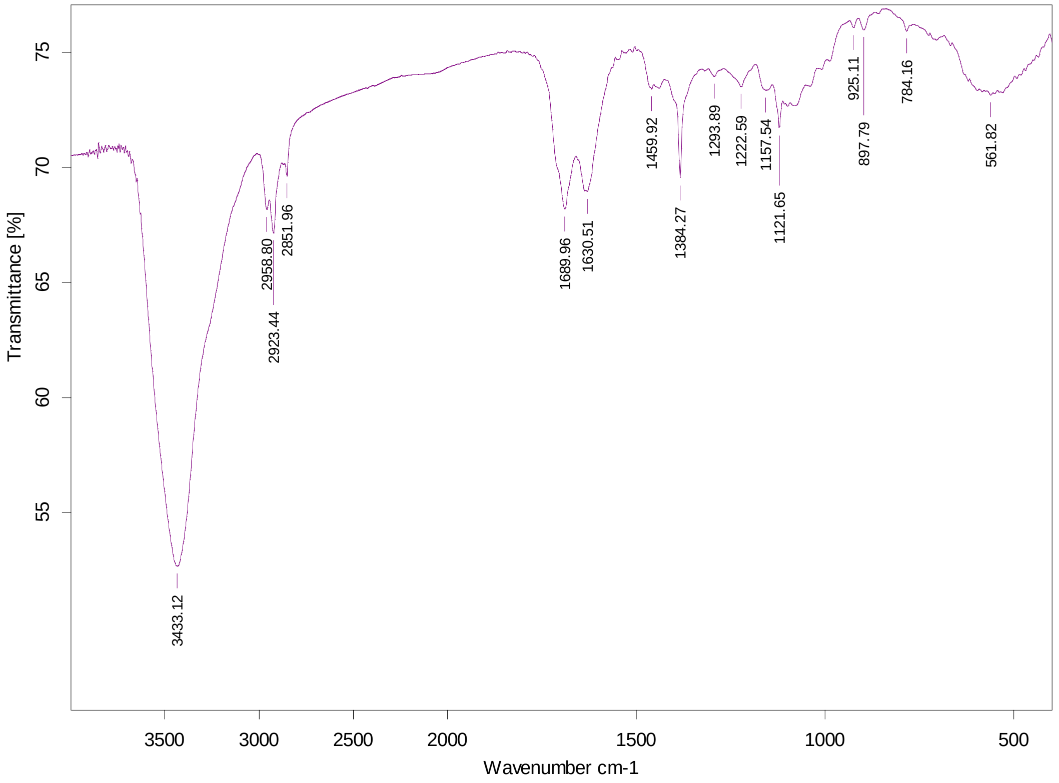

^{1}H NMR for compound **2** (in CD_3OD, 400 MHz)

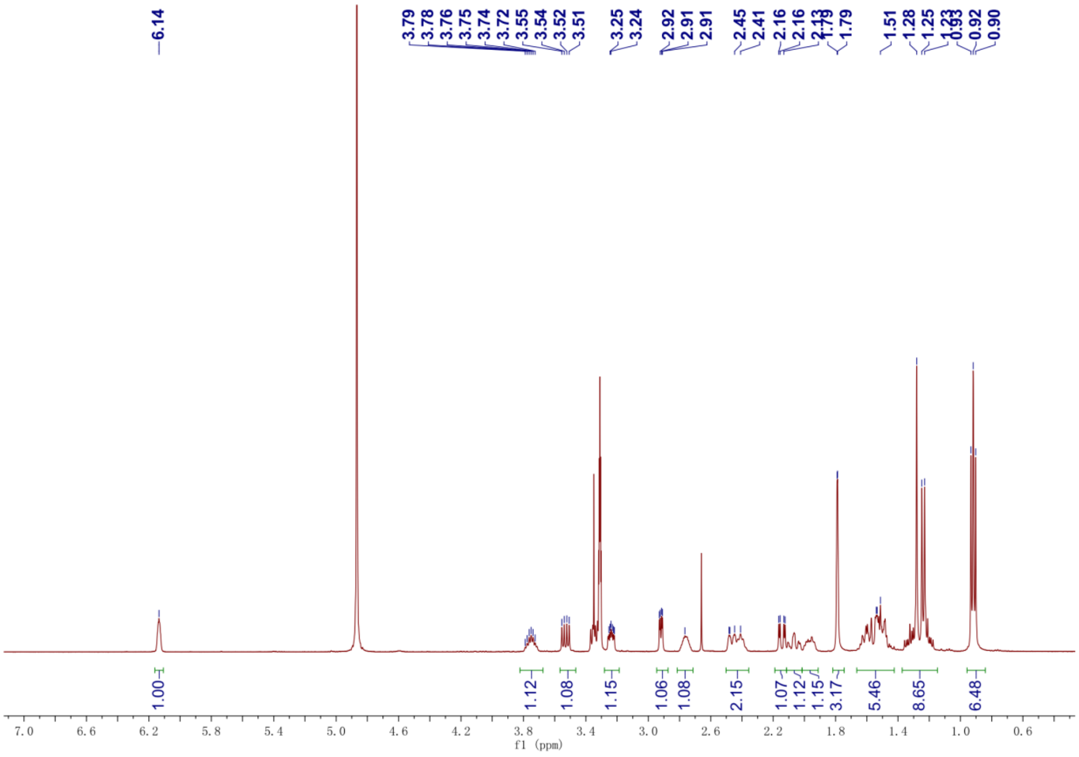

^{13}C NMR for compound **2** (in CD_3OD, 100 MHz)

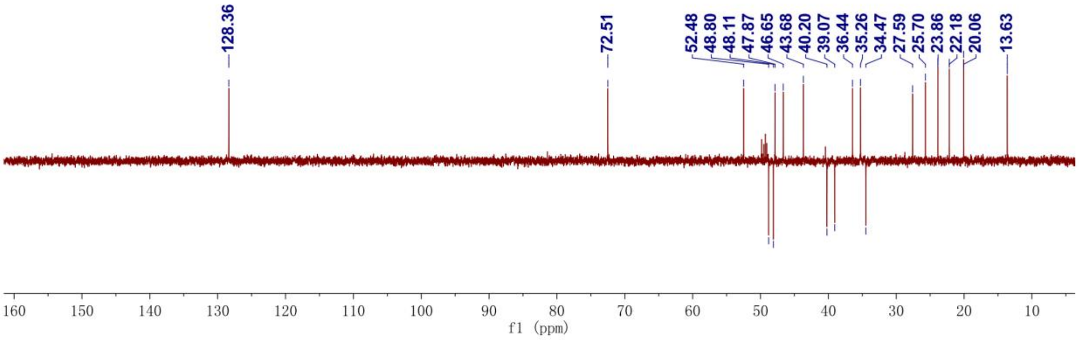

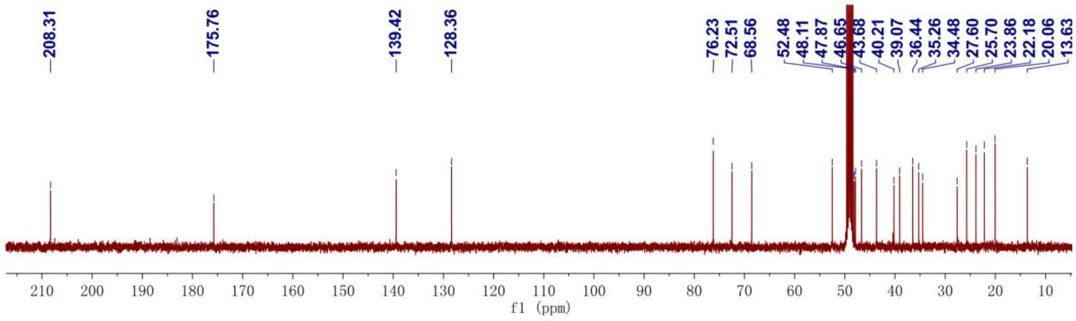

HSQC for compound **2** (in CD_3OD, 400 MHz)

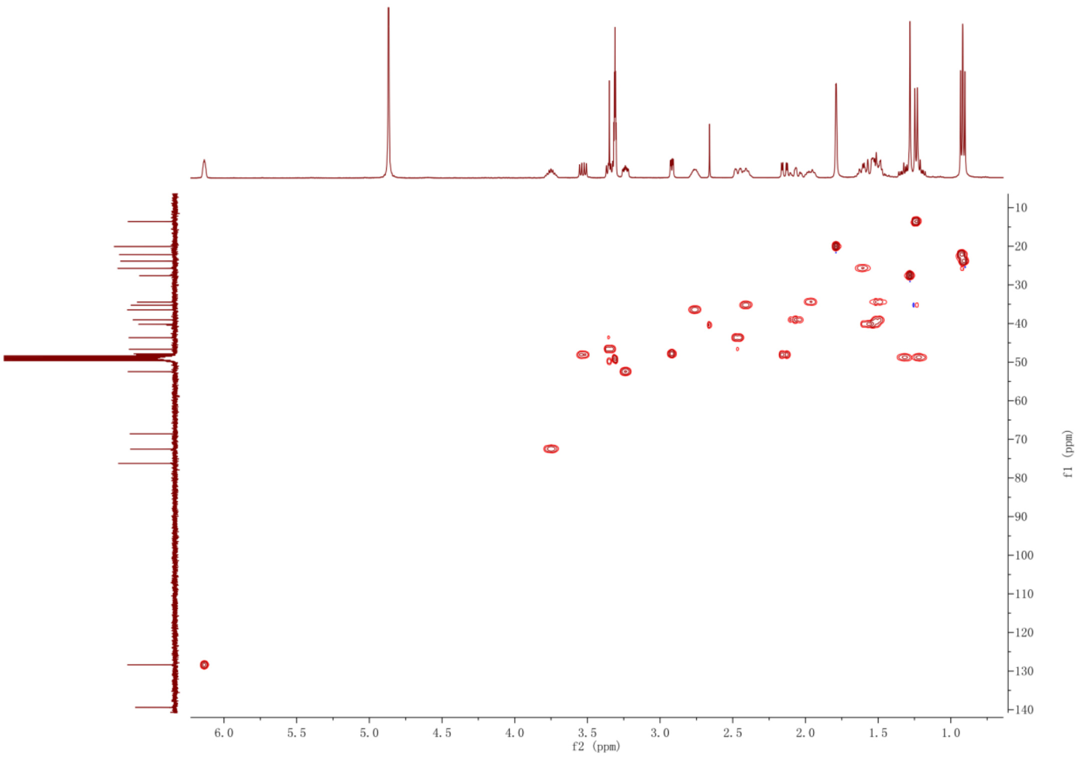

HMBC for compound **2** (in CD_3OD, 400 MHz)

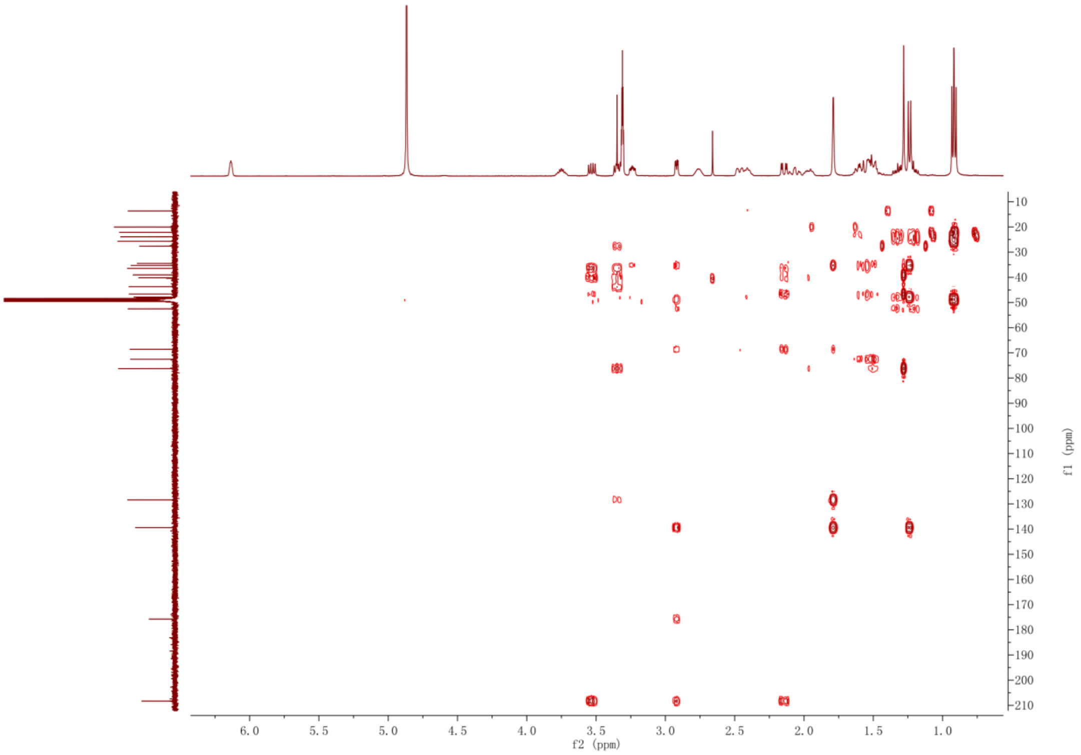

^1H–^1H COSY for compound **2** (in CD_3OD, 400 MHz)

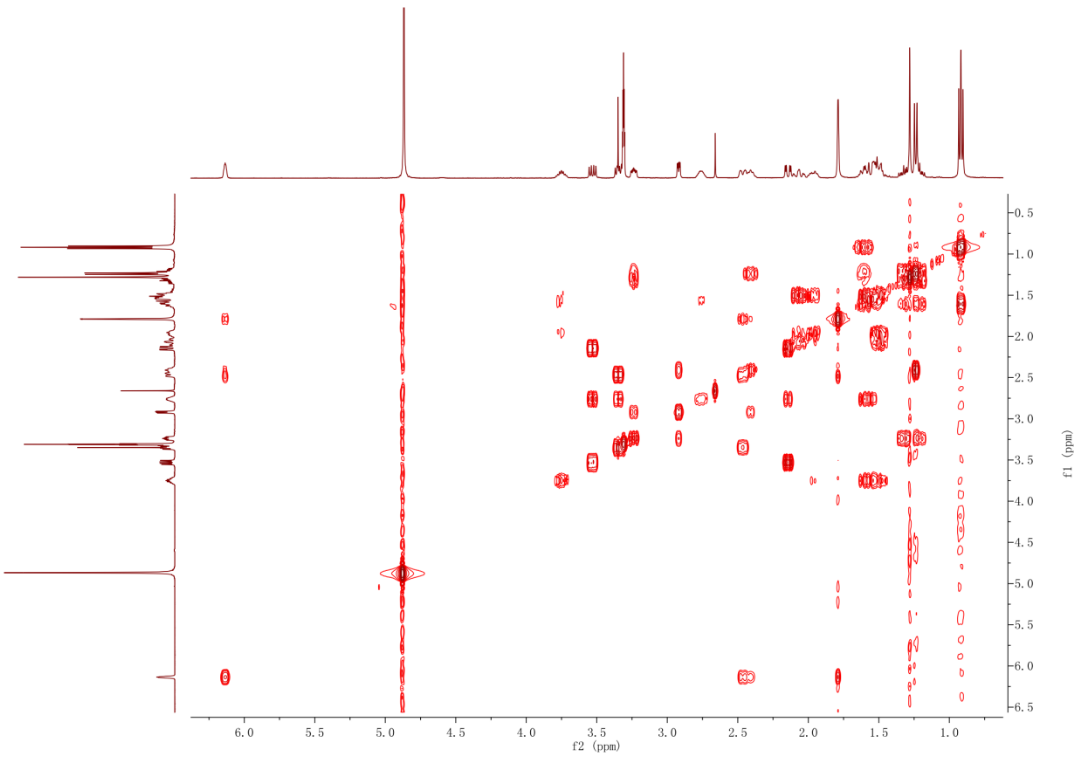

NOESY for compound **1** (in CD_3OD, 400 MHz)

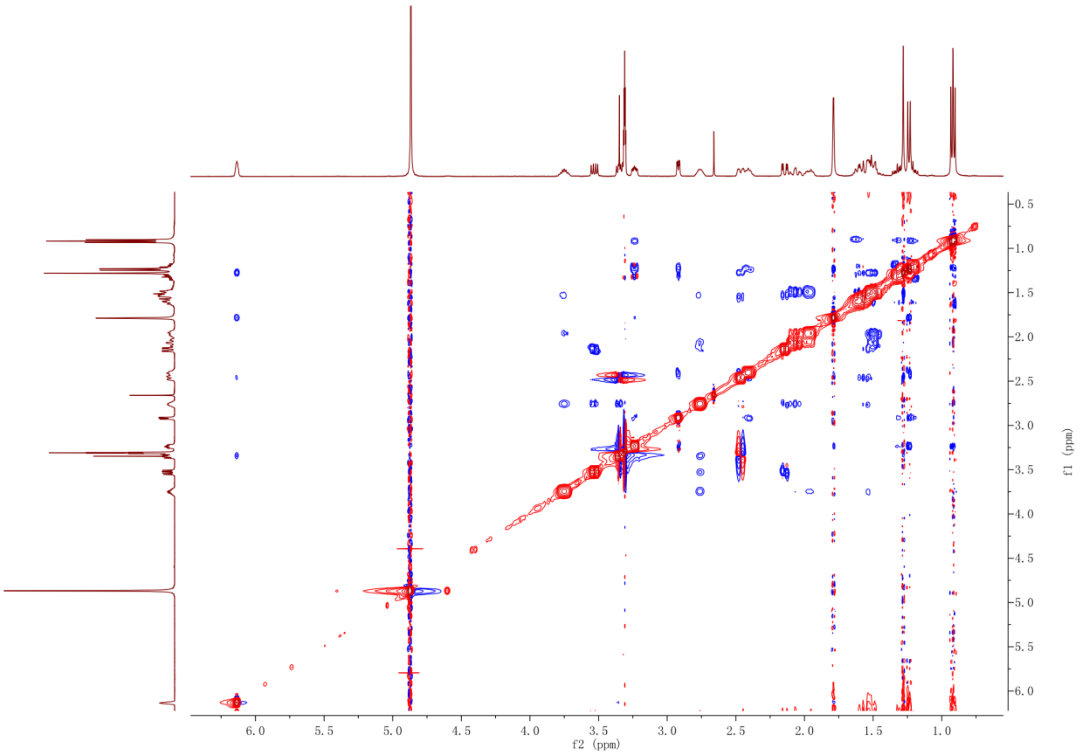

HRESIMS for compound **3**

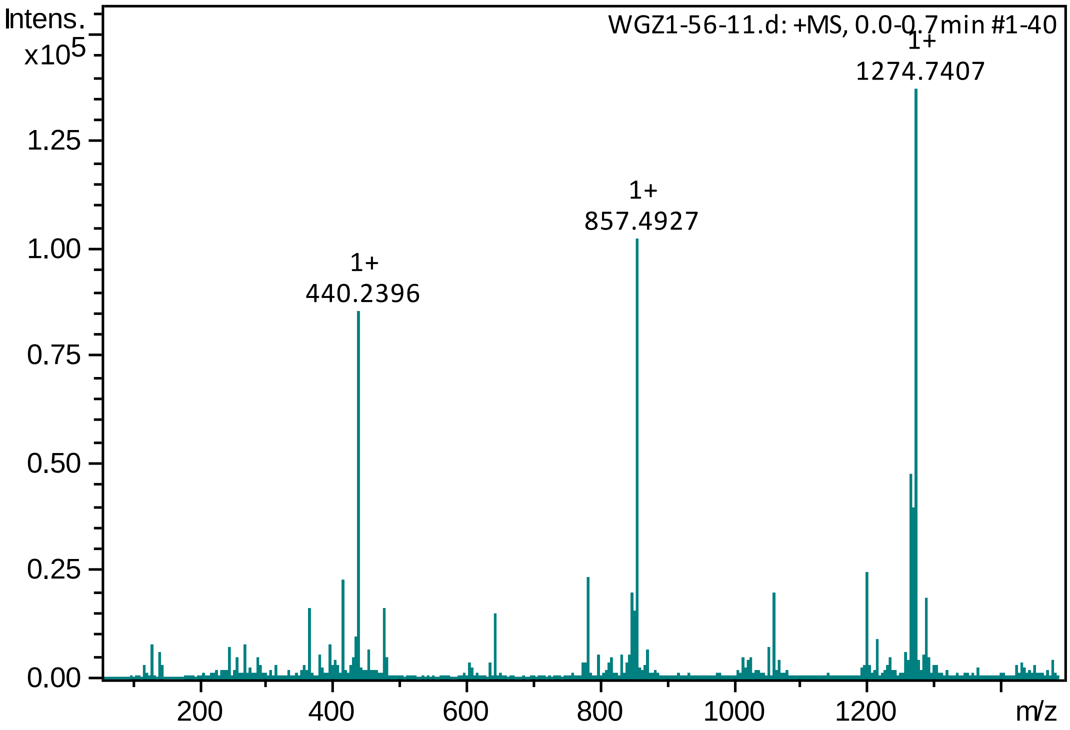

UV spectrum for compound **3**

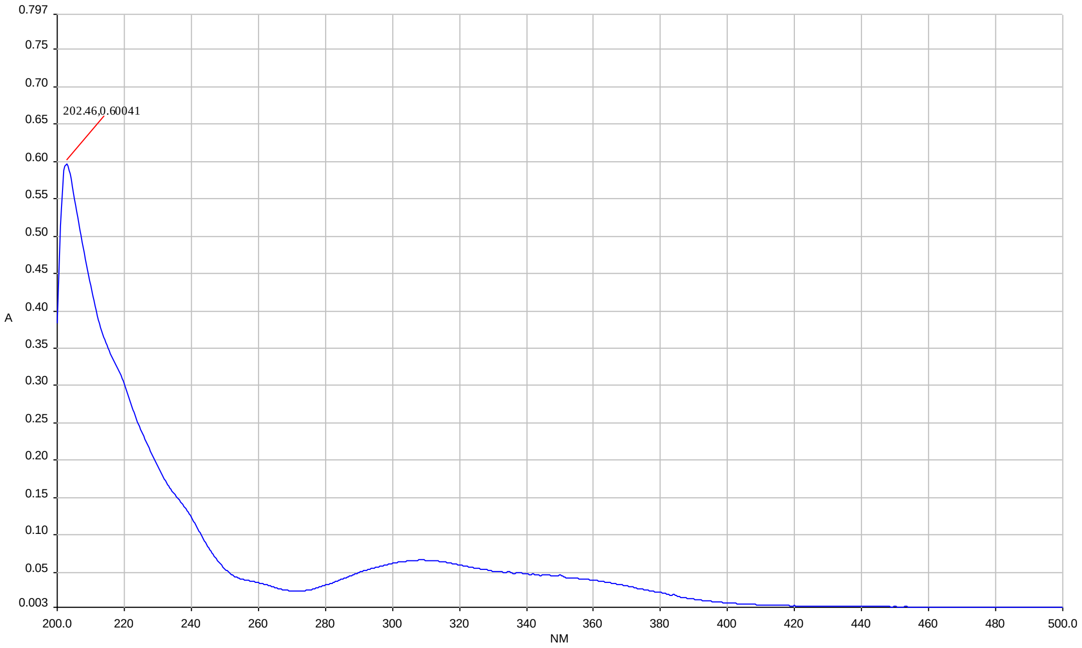

IR spectrum for compound **3**

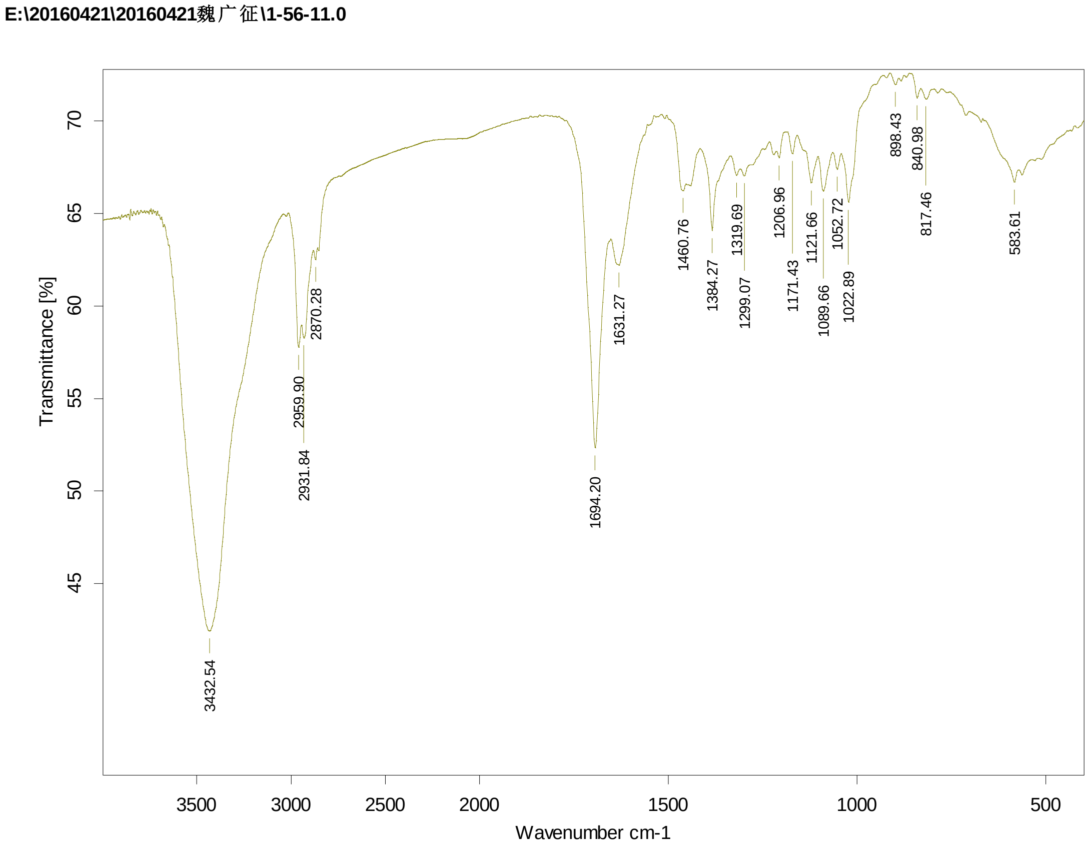

^{1}H NMR for compound **3** (in CD_3OD, 400 MHz)

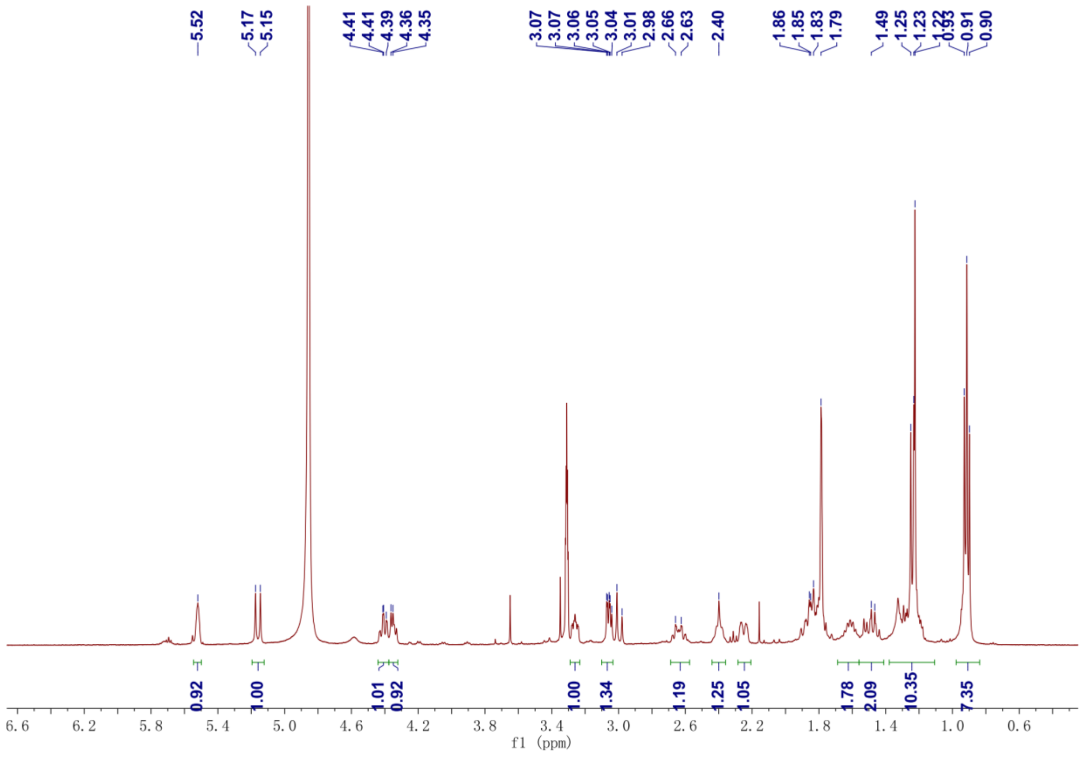

^{13}C NMR for compound **3** (in CD_3OD, 100 MHz)

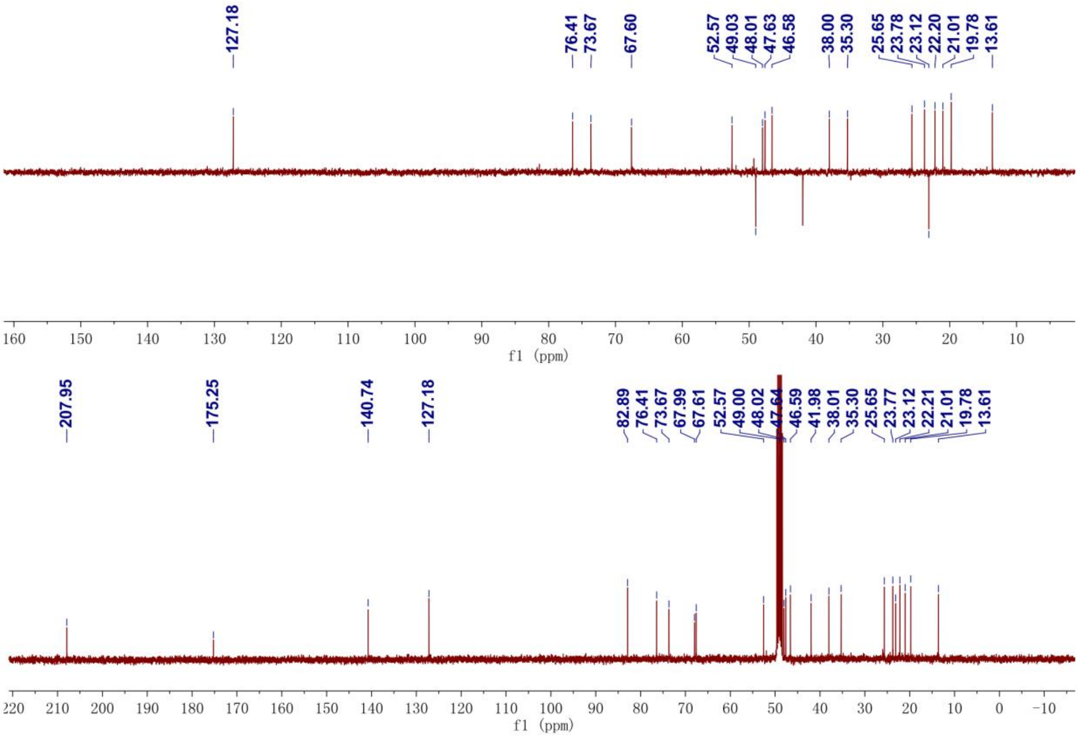

HSQC for compound **3** (in CD_3OD, 400 MHz)

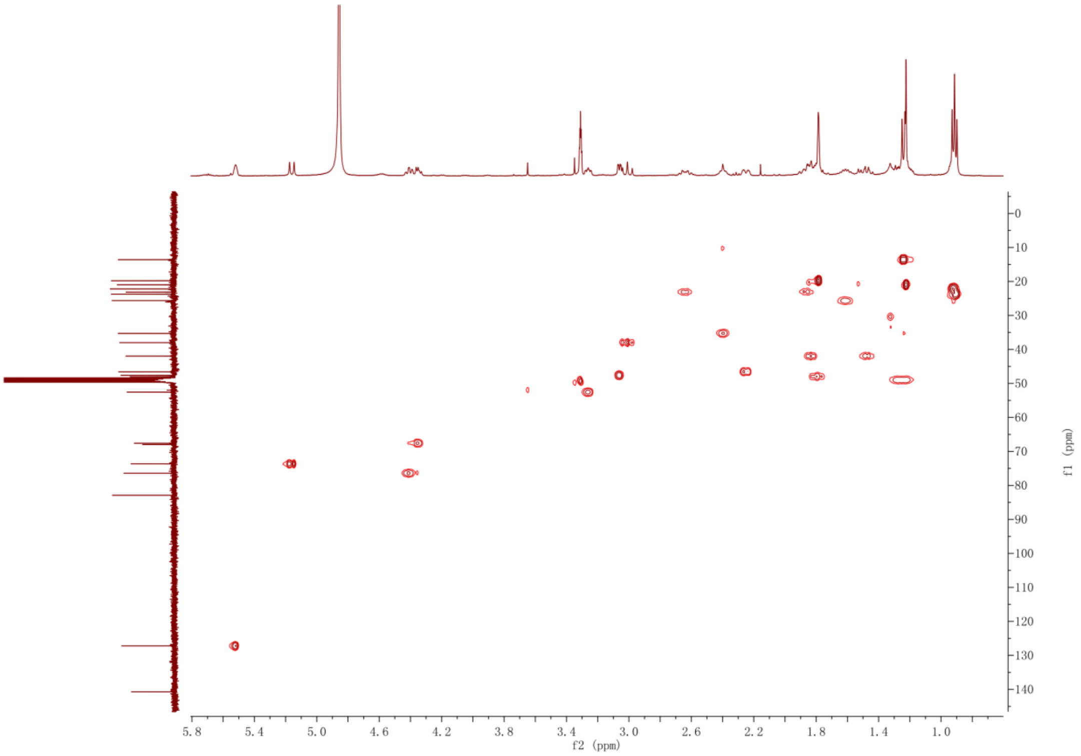

HMBC for compound **3** (in CD_3OD, 400 MHz)

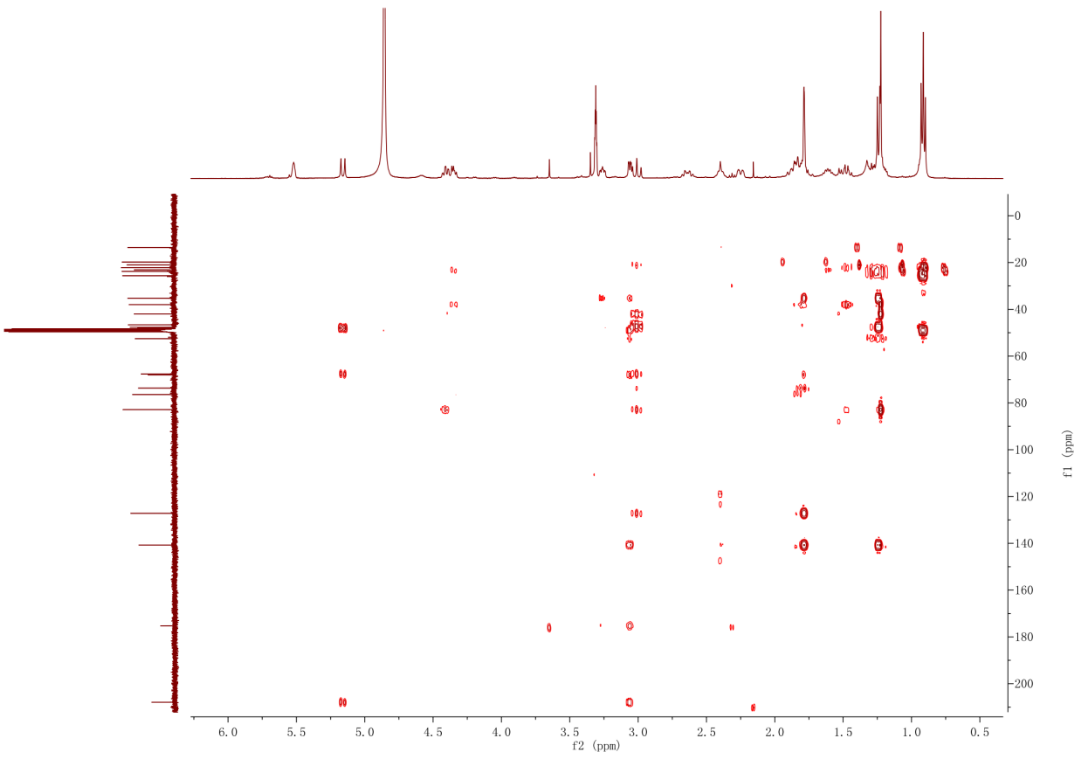

^{1}H–^{1}H COSY for compound **3** (in CD_3OD, 400 MHz)

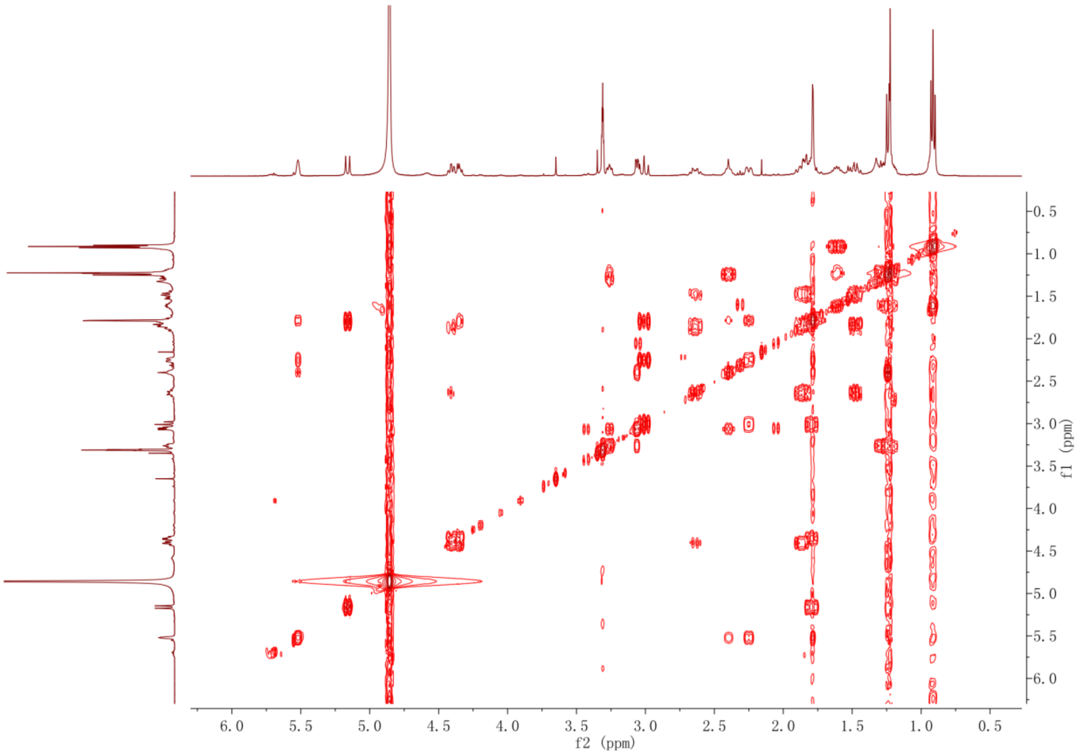

NOESY for compound **3** (in CD_3OD, 400 MHz)

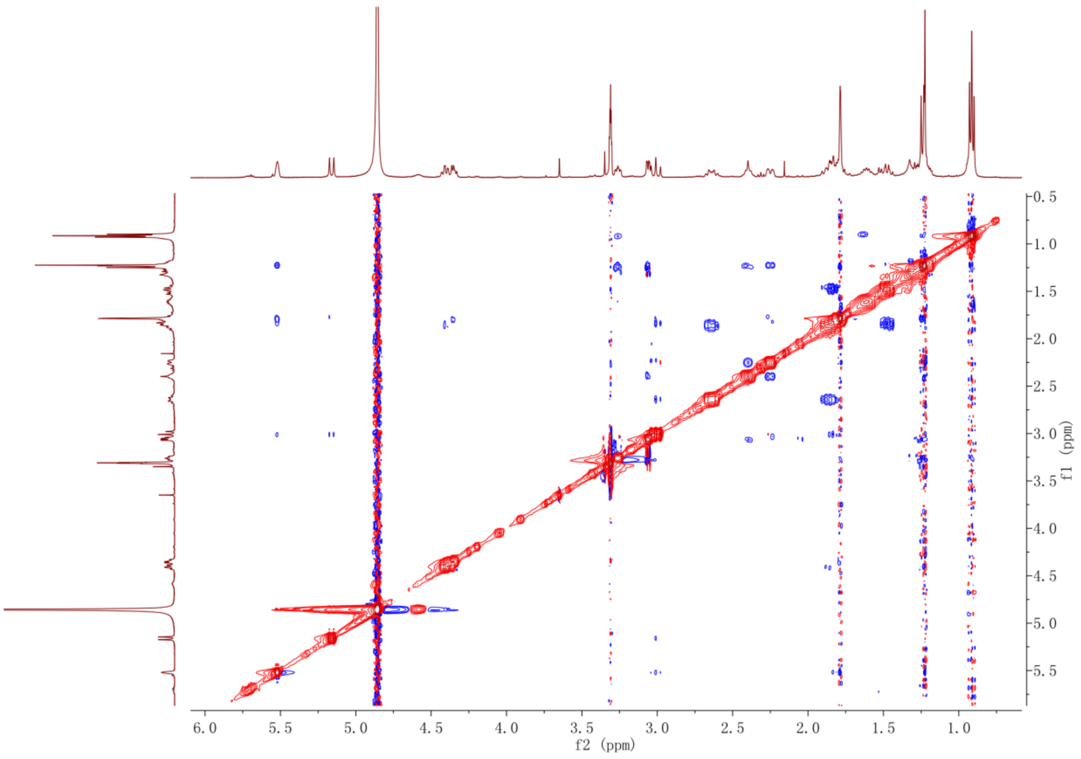

HRESIMS for compound **4**

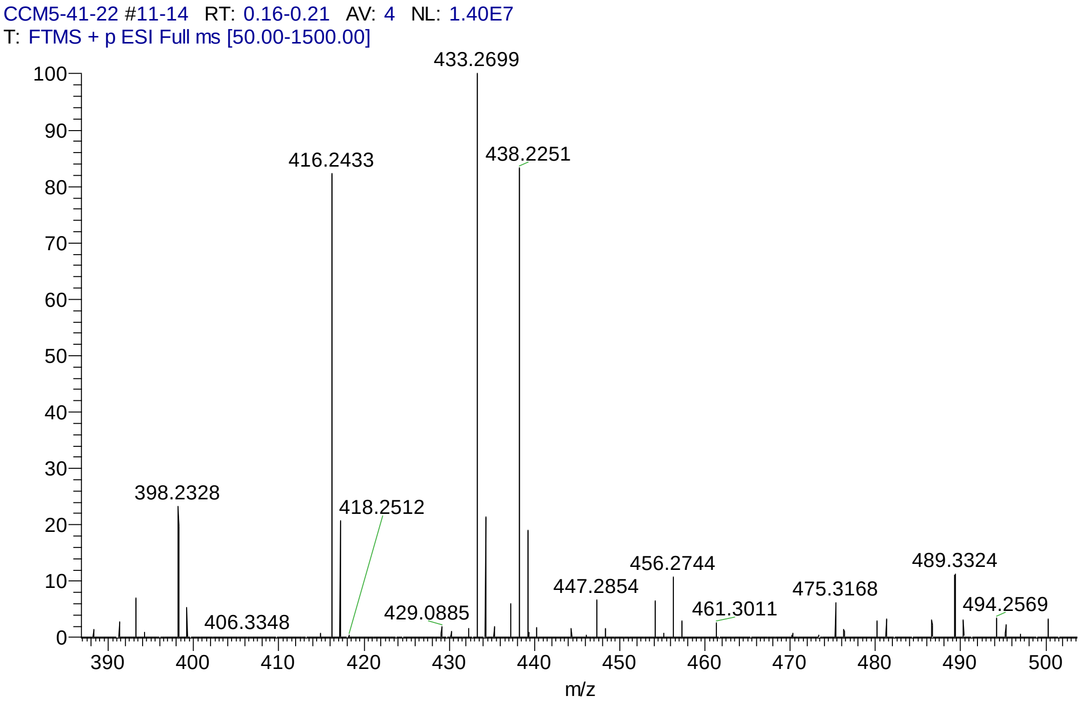

UV spectrum for compound **4**

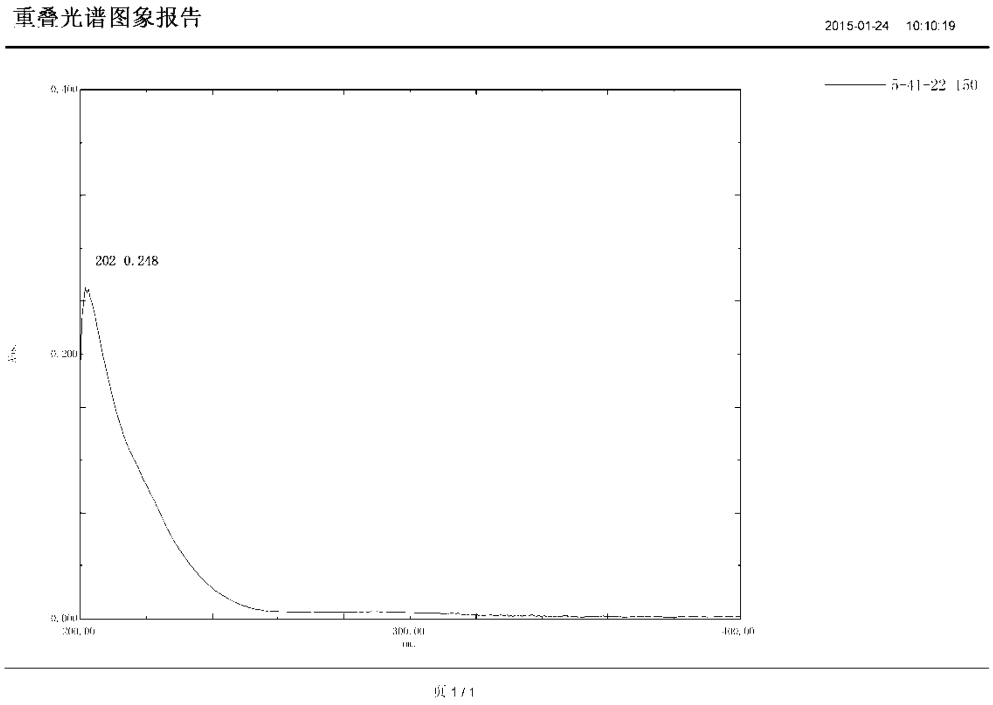

IR spectrum for compound **4**

E:\同济医学院\张勇慧\20150128\AF5-41-22.0

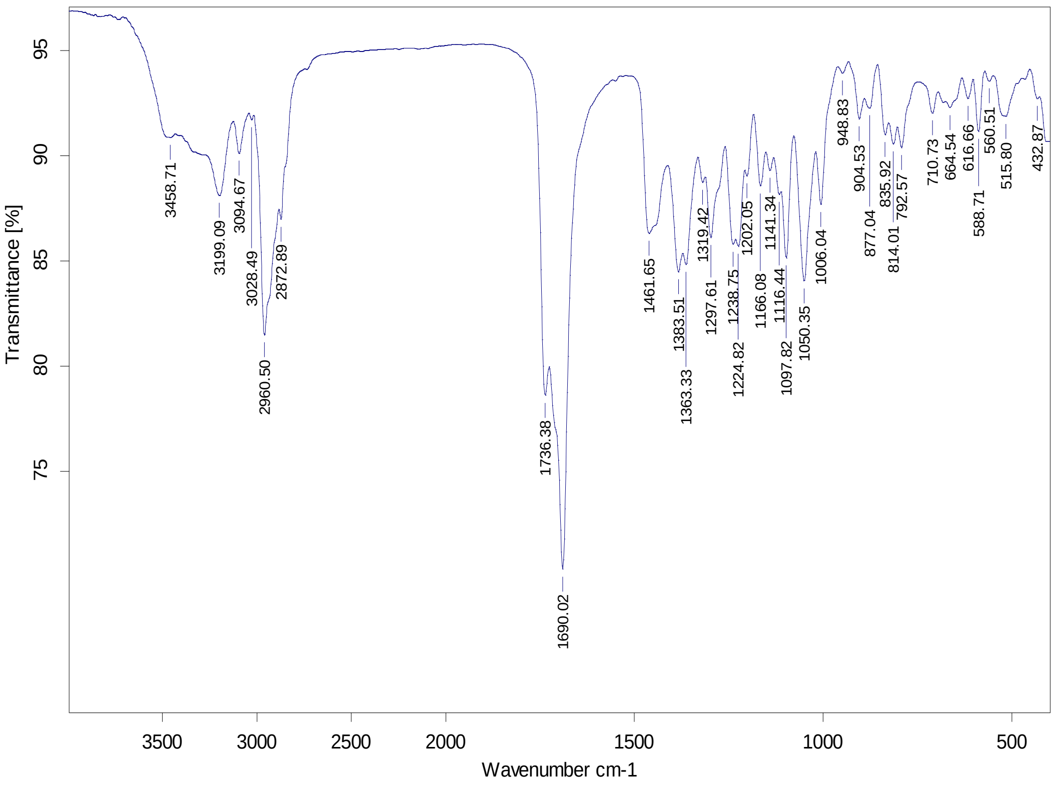

^{1}H NMR for compound **4** (in CD_3OD, 400 MHz)

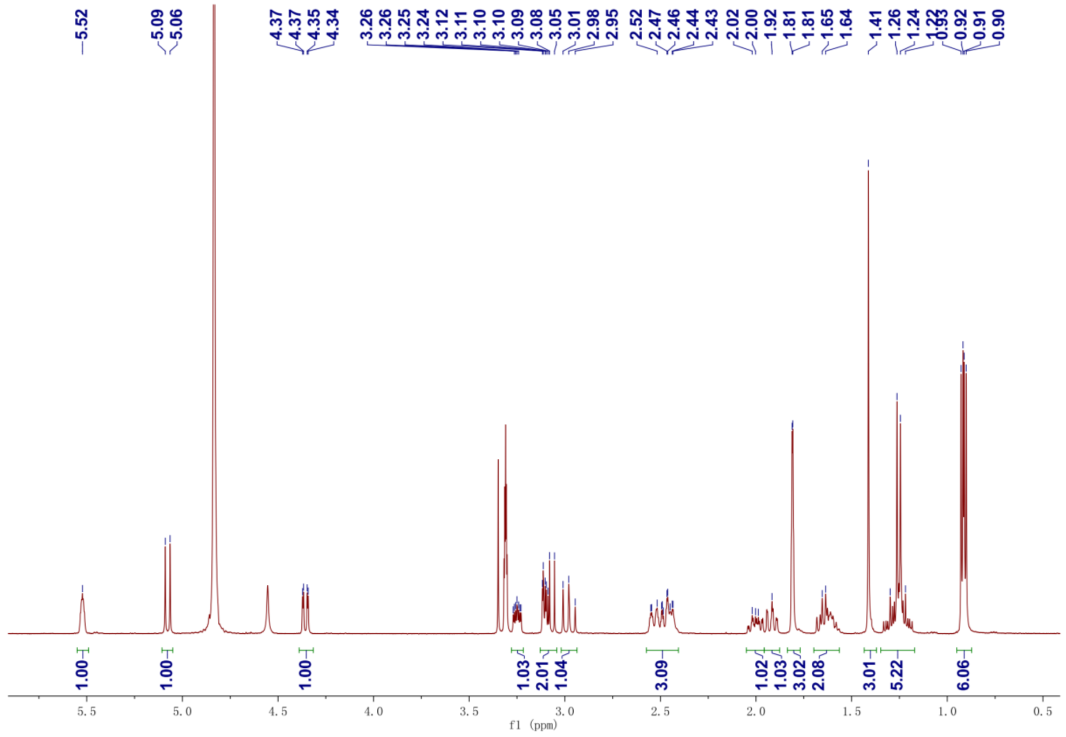

^{13}C NMR for compound **4** (in CD_3OD, 100 MHz)

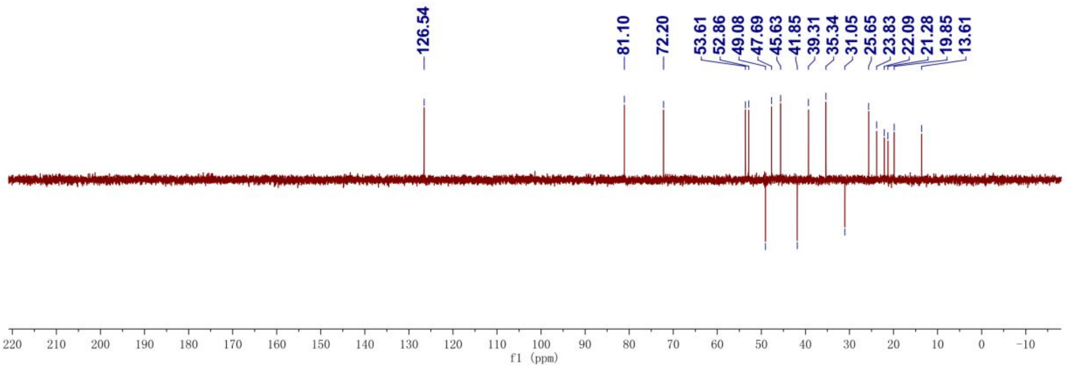

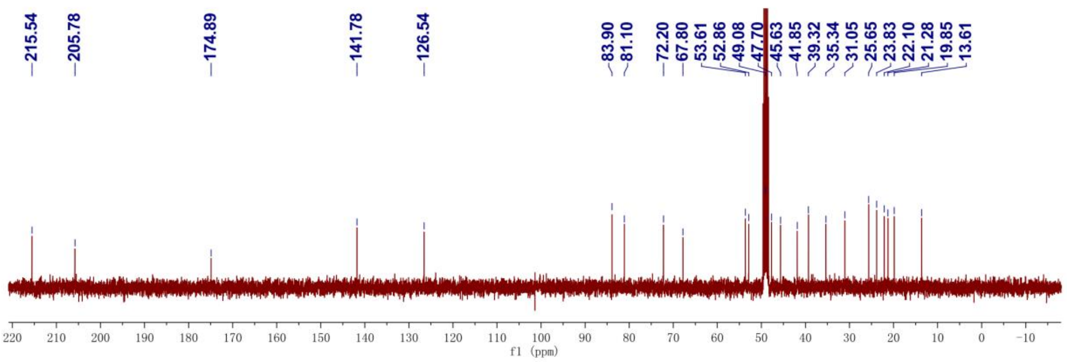

HSQC for compound **4** (in CD_3OD, 400 MHz)

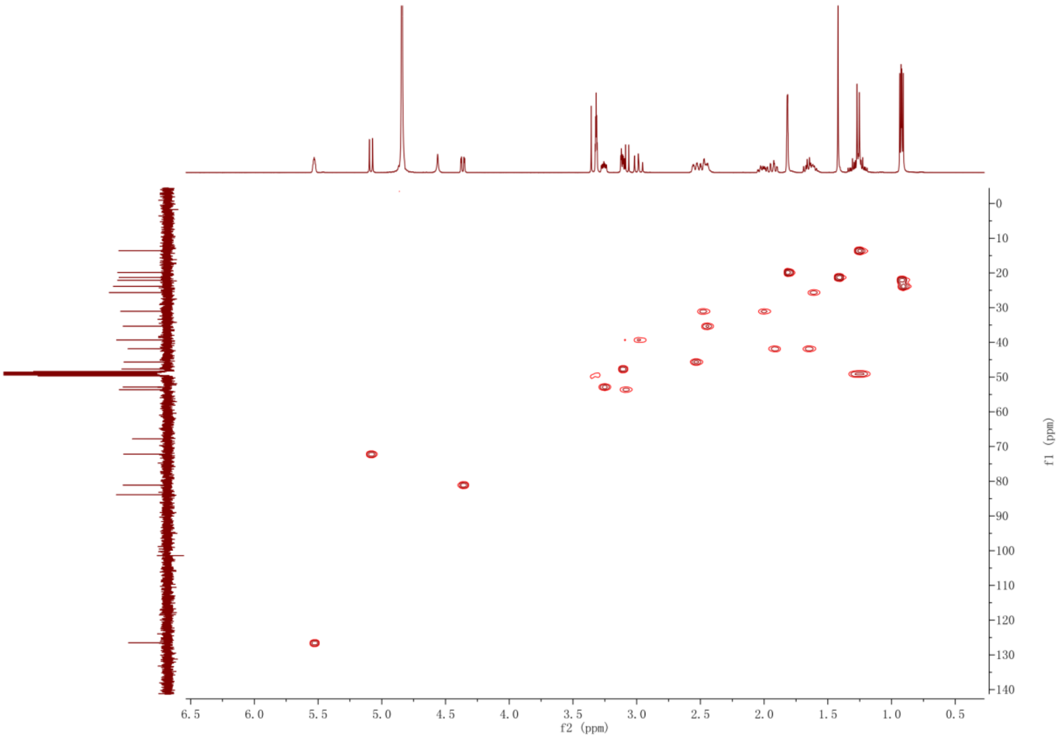

HMBC for compound **4** (in CD_3OD, 400 MHz)

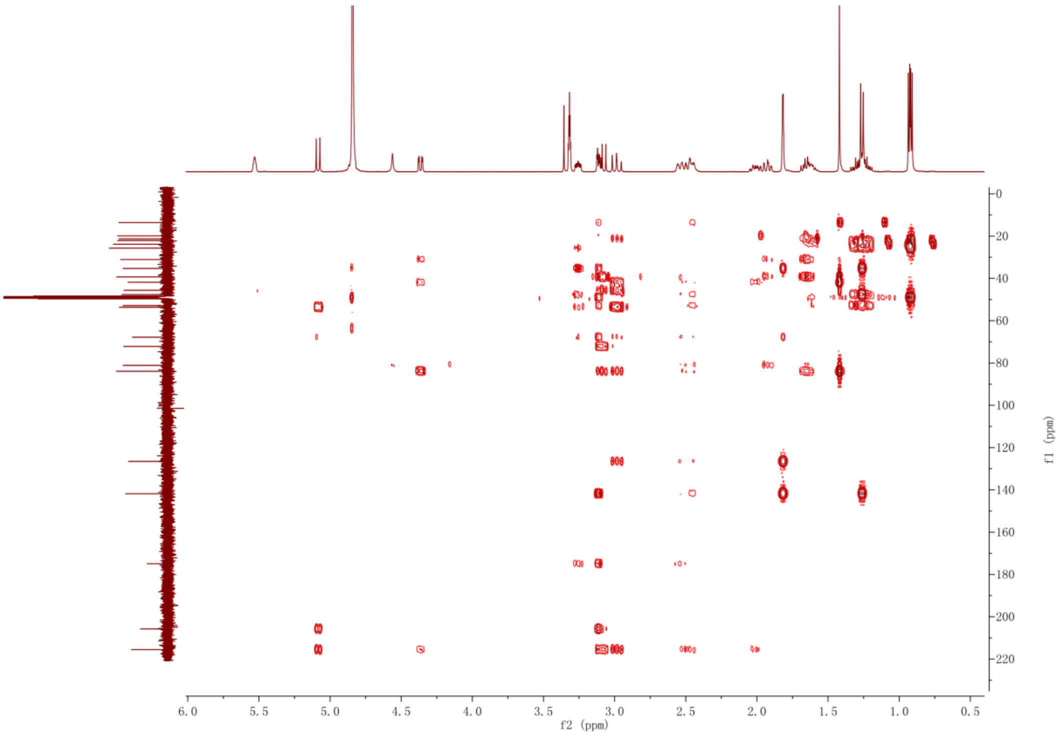

^{1}H–^{1}H COSY for compound **4** (in CD_3OD, 400 MHz)

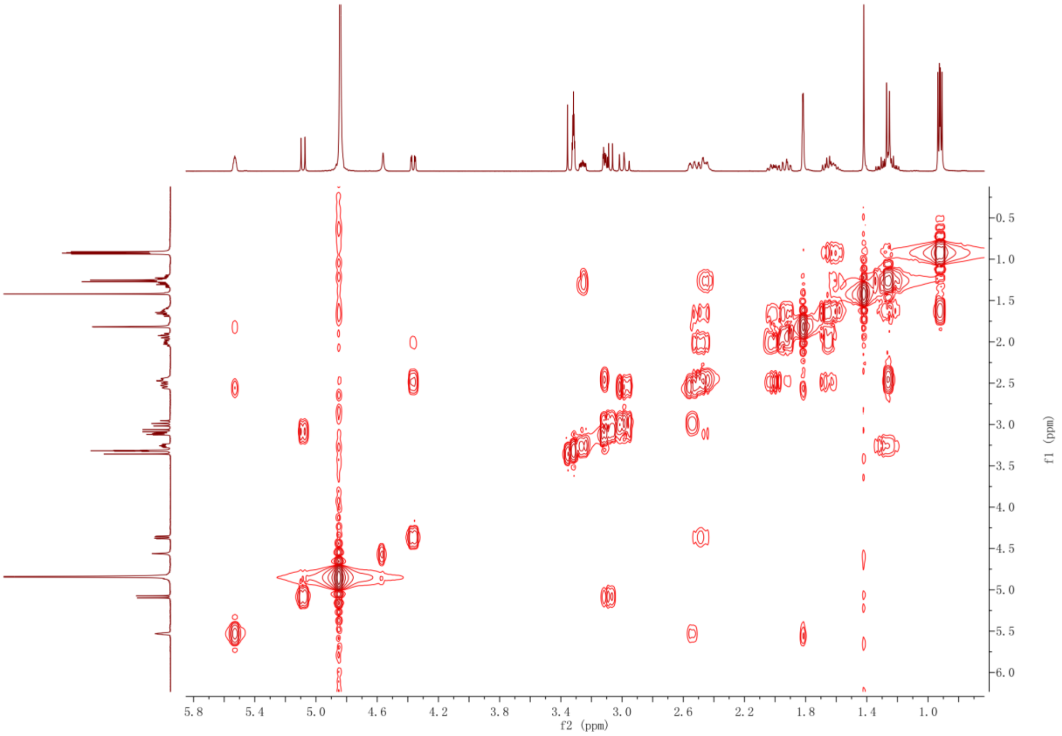

NOESY for compound **4** (in CD_3OD, 400 MHz)

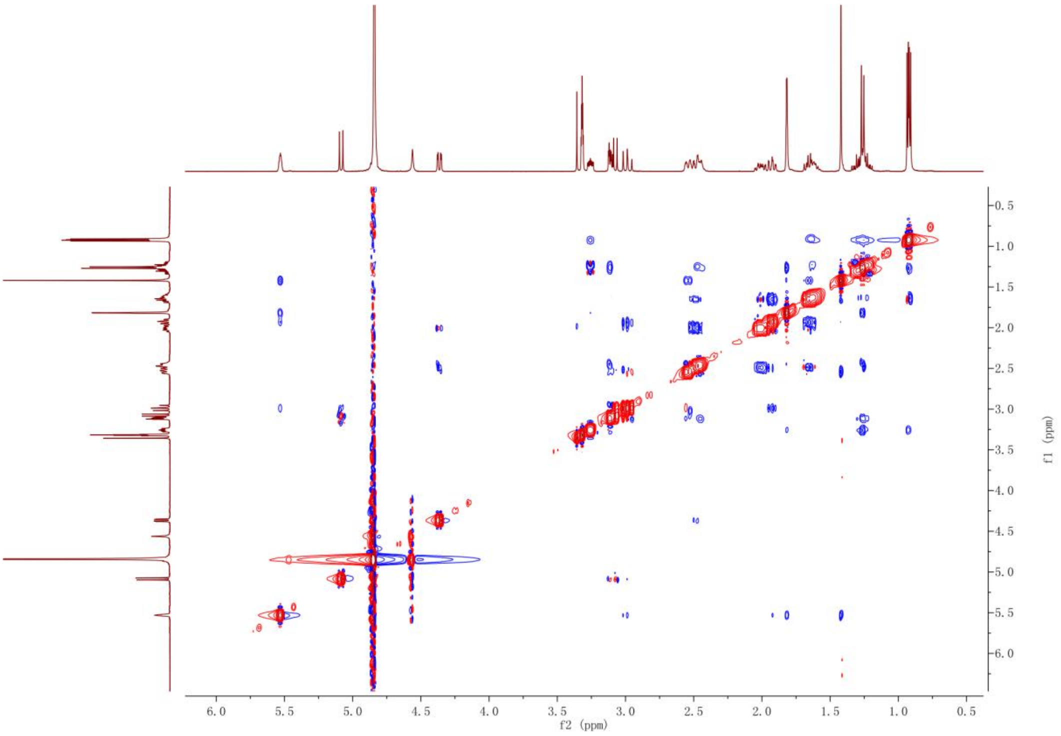

HRESIMS for compound **5**

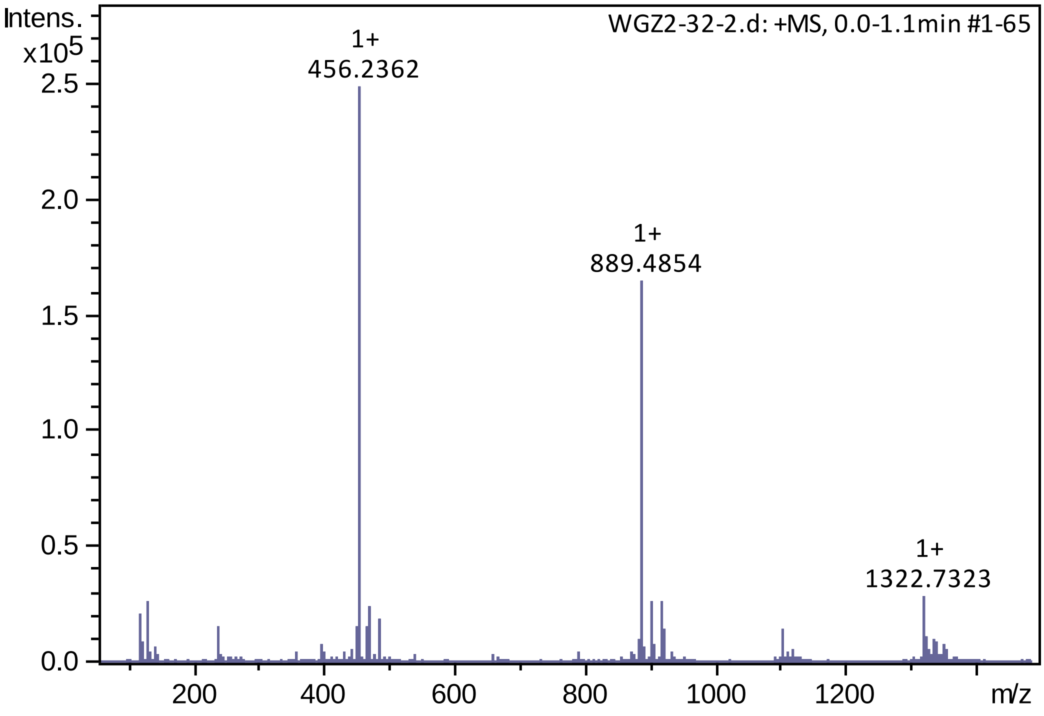

UV spectrum for compound **5**

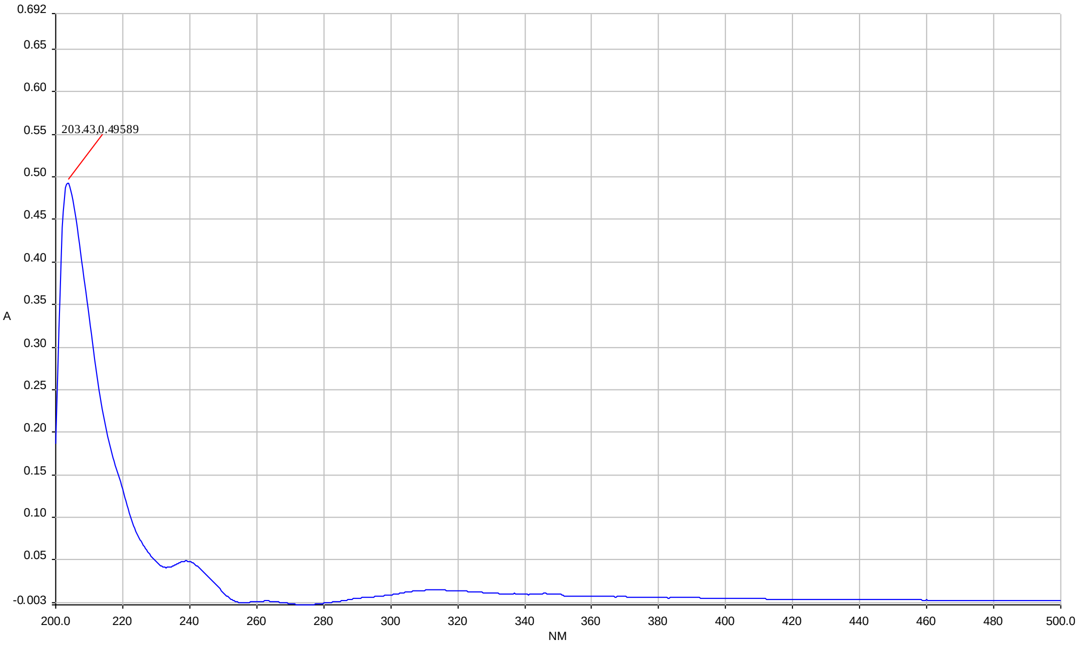

IR spectrum for compound **5**

E:\20160421\20160421魏广征\2-32-2.0

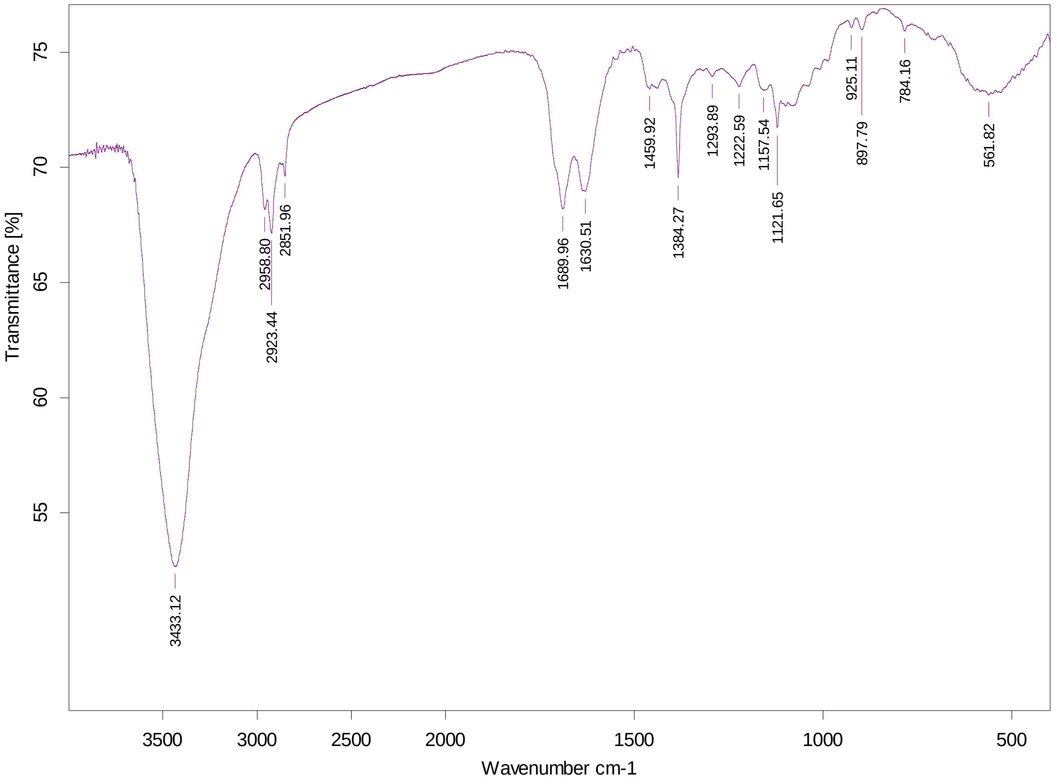

^{1}H NMR for compound **5** (in CD_3OD, 400 MHz)

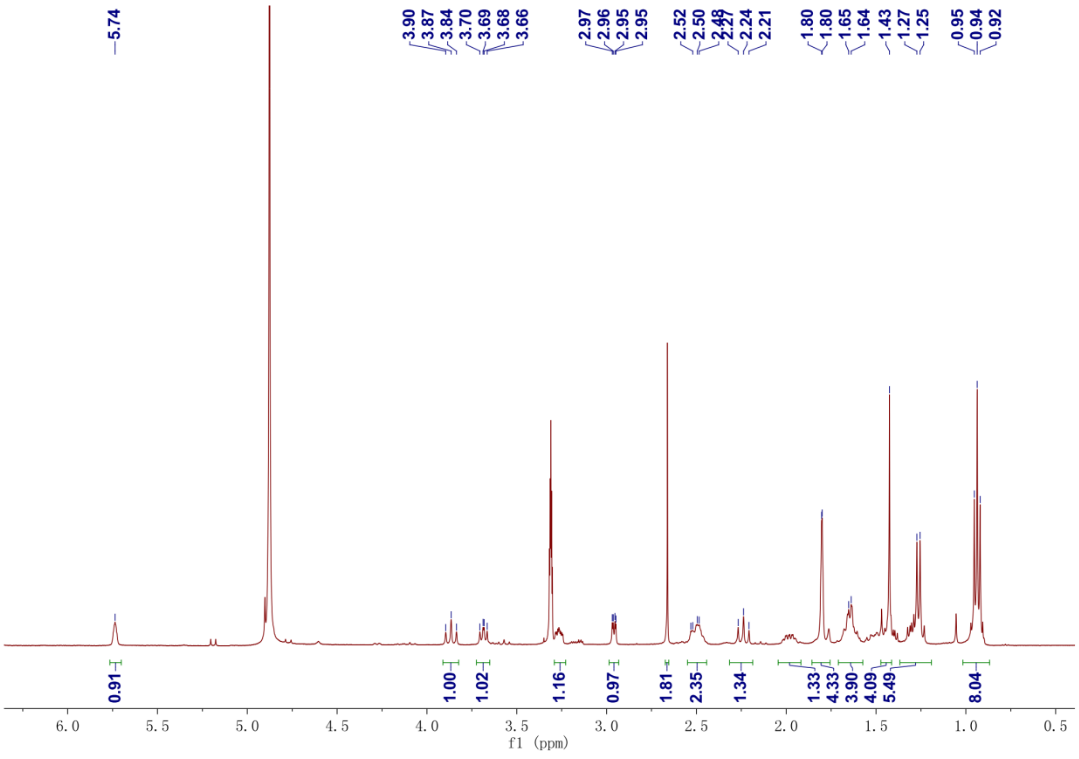

^{13}C NMR for compound **5** (in CD_3OD, 100 MHz)

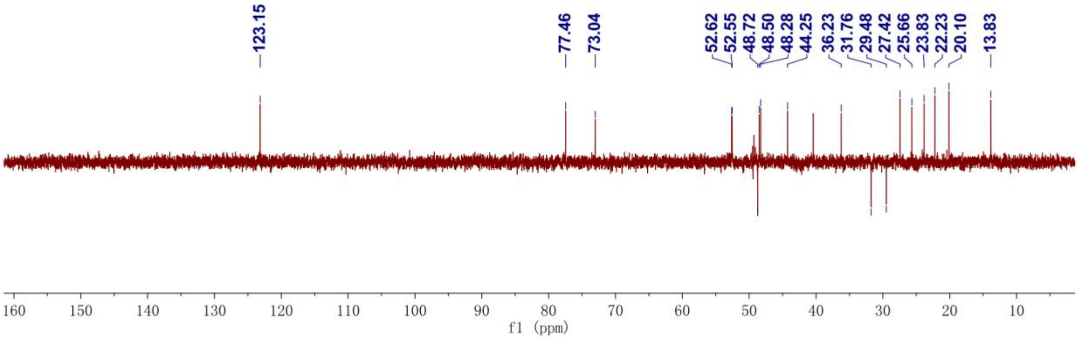

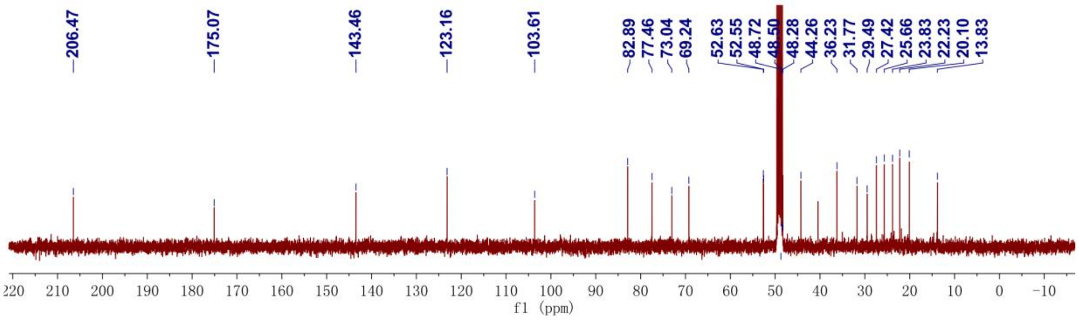

HSQC for compound **5** (in CD_3OD, 400 MHz)

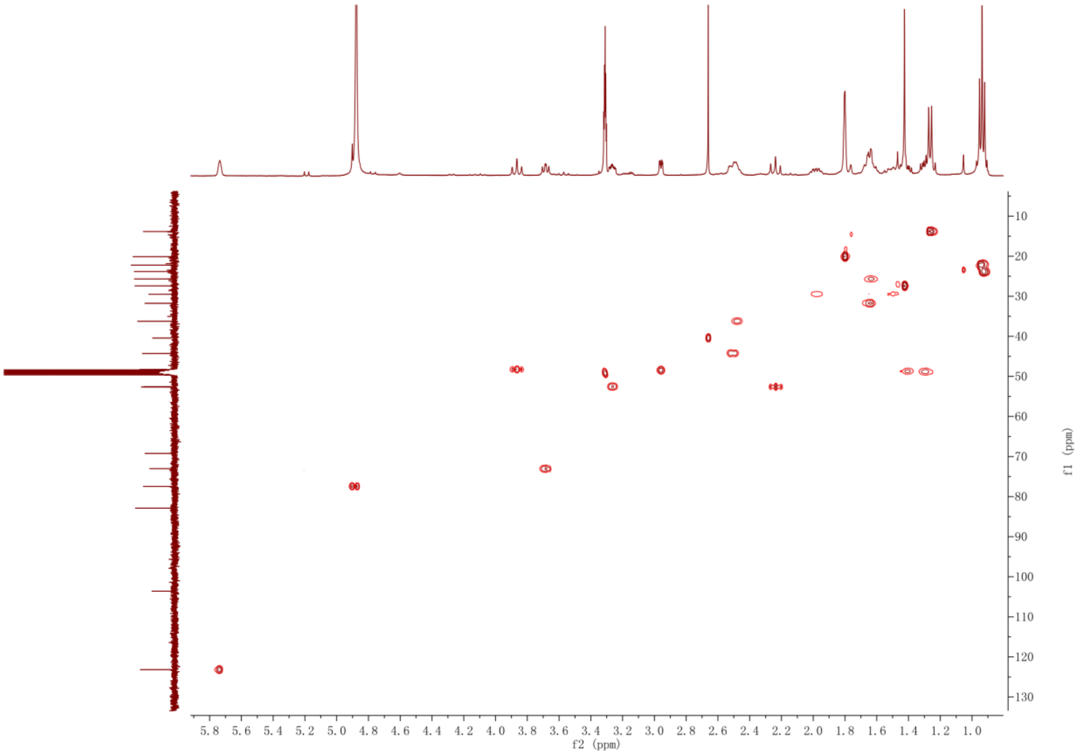

HMBC for compound **5** (in CD_3OD, 400 MHz)

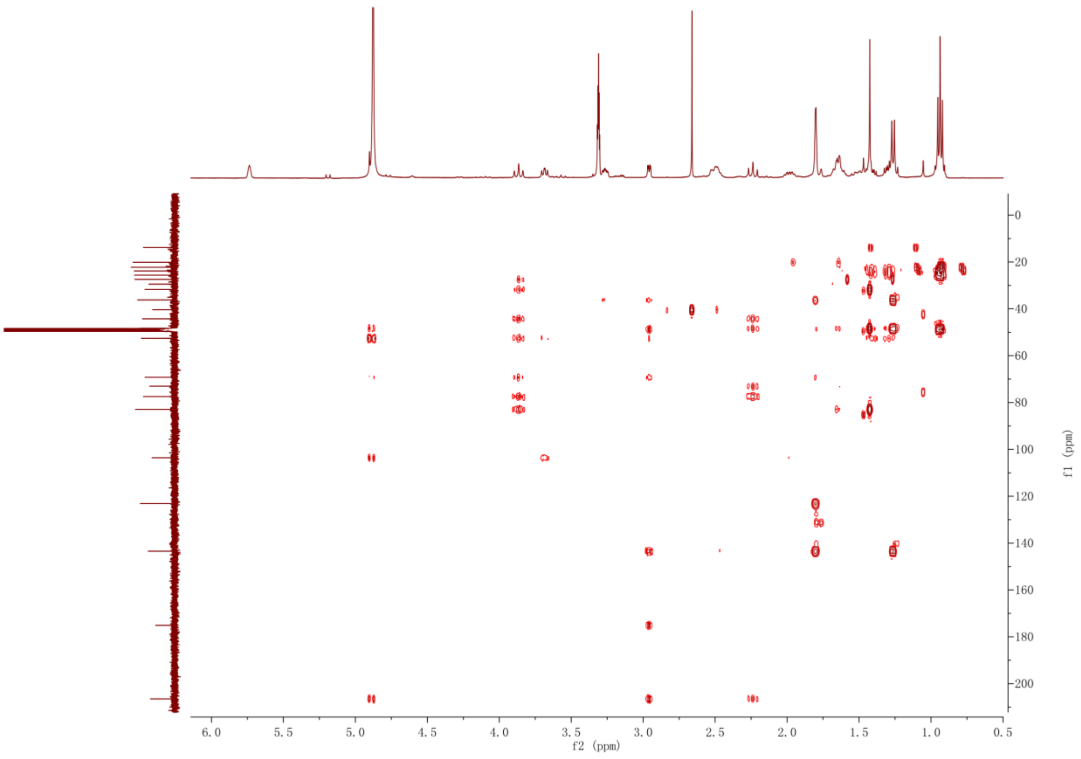

^1H–^1H COSY for compound **5** (in CD_3OD, 400 MHz)

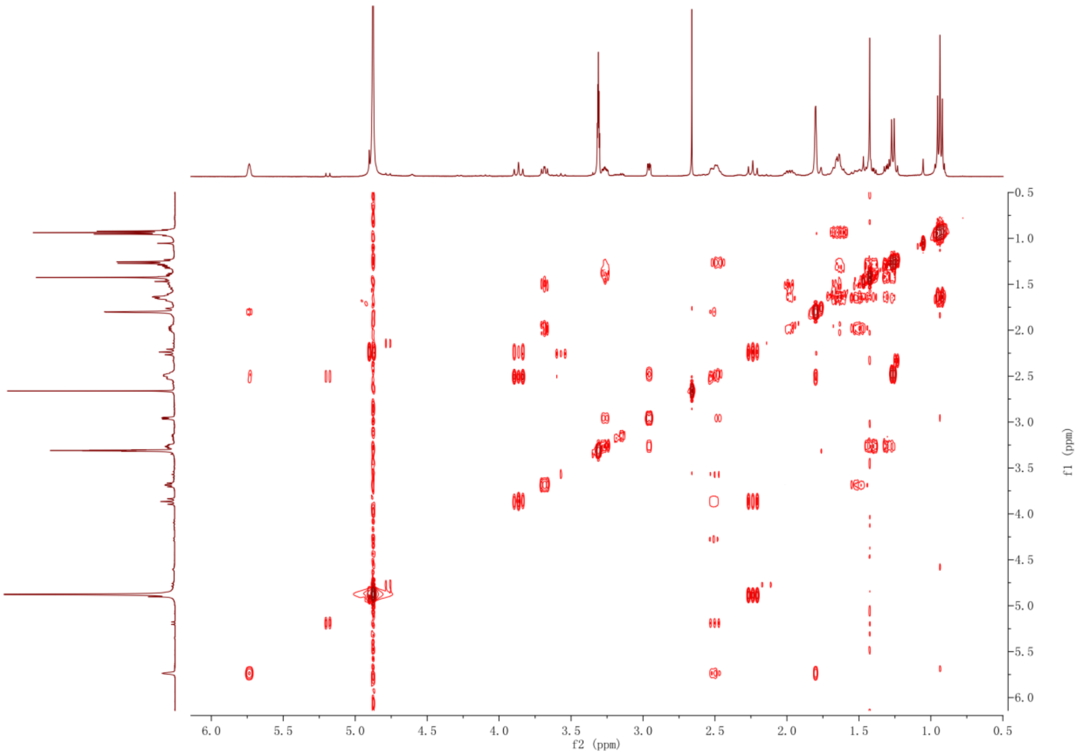

NOESY for compound **5** (in CD_3OD, 400 MHz)

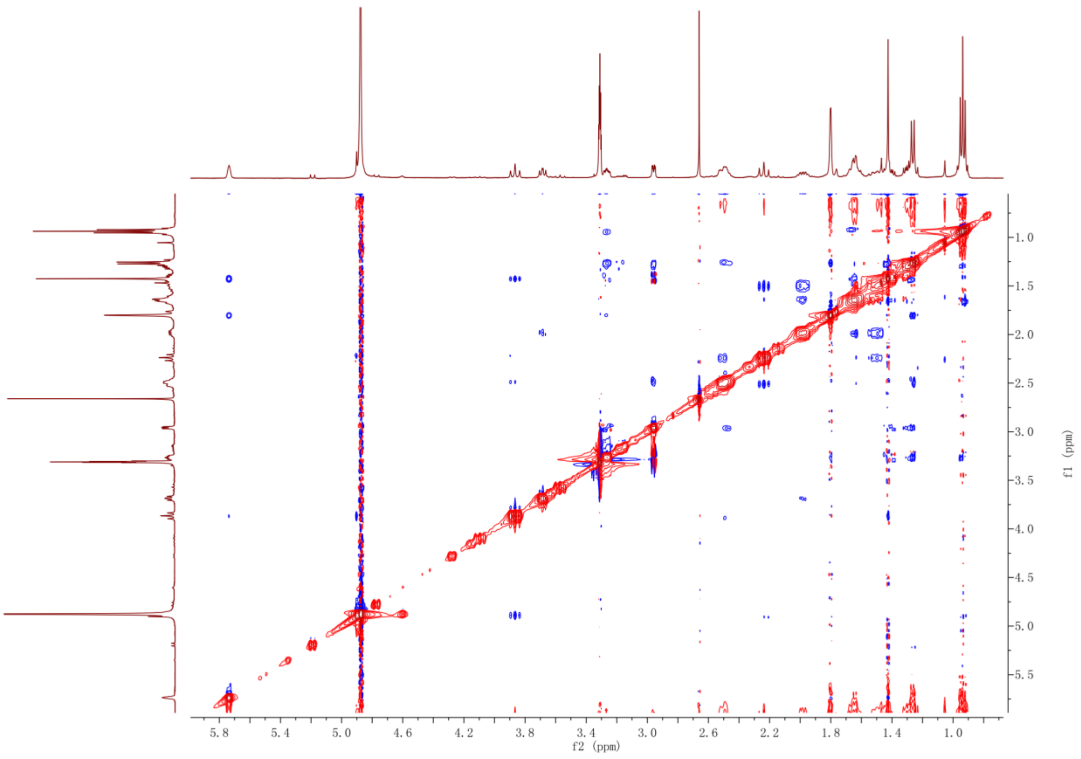

HRESIMS for compound **6**

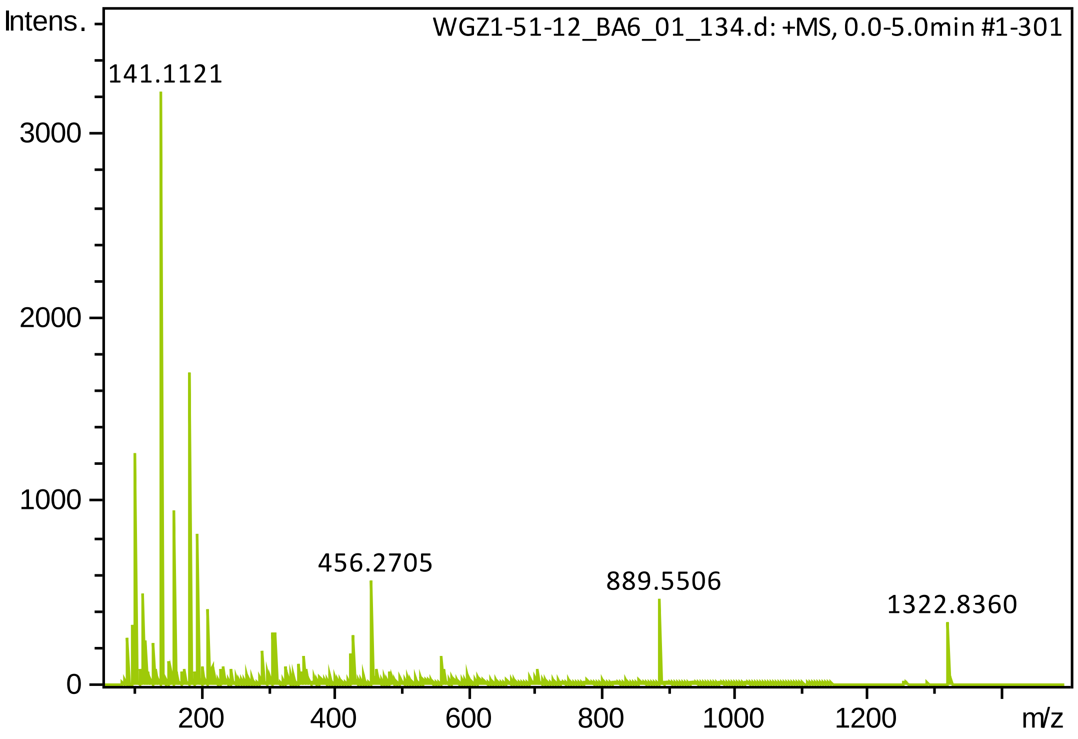

UV spectrum for compound **6**

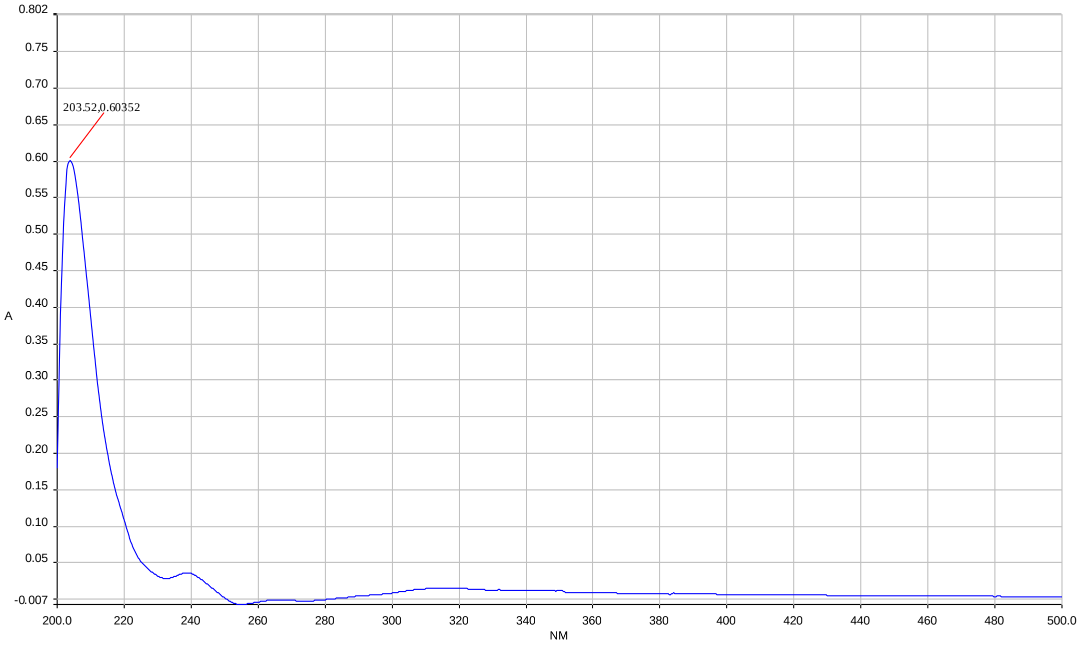

IR spectrum for compound **6**

E:\20160428\20160428谭冬冬\TDD1-34-11.0

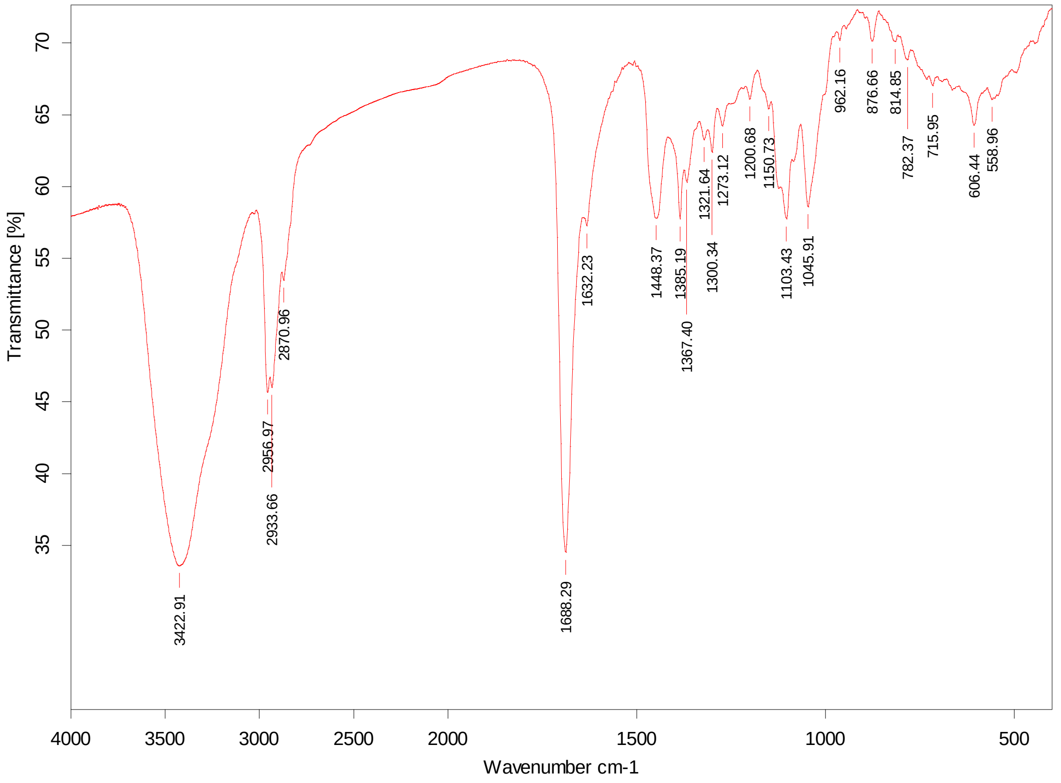

^{1}H NMR for compound **6** (in CD_3OD, 400 MHz)

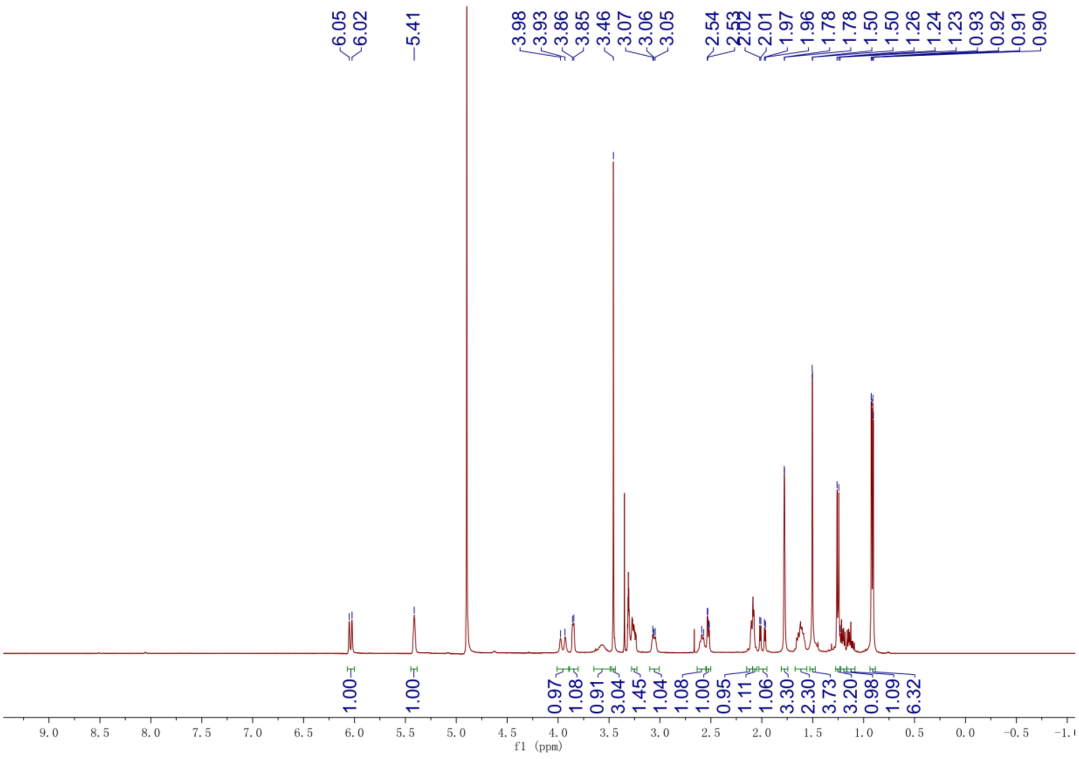

^{13}C NMR for compound **6** (in CD_3OD, 100 MHz)

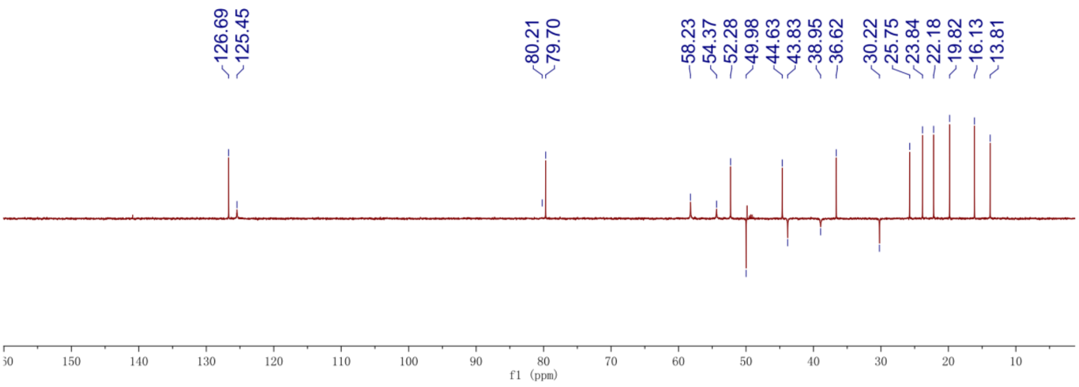

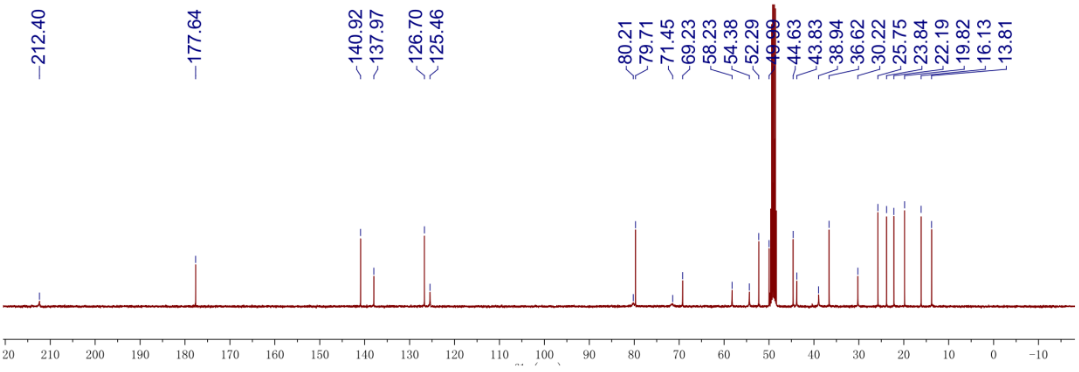

HSQC for compound **6** (in CD_3OD, 400 MHz)

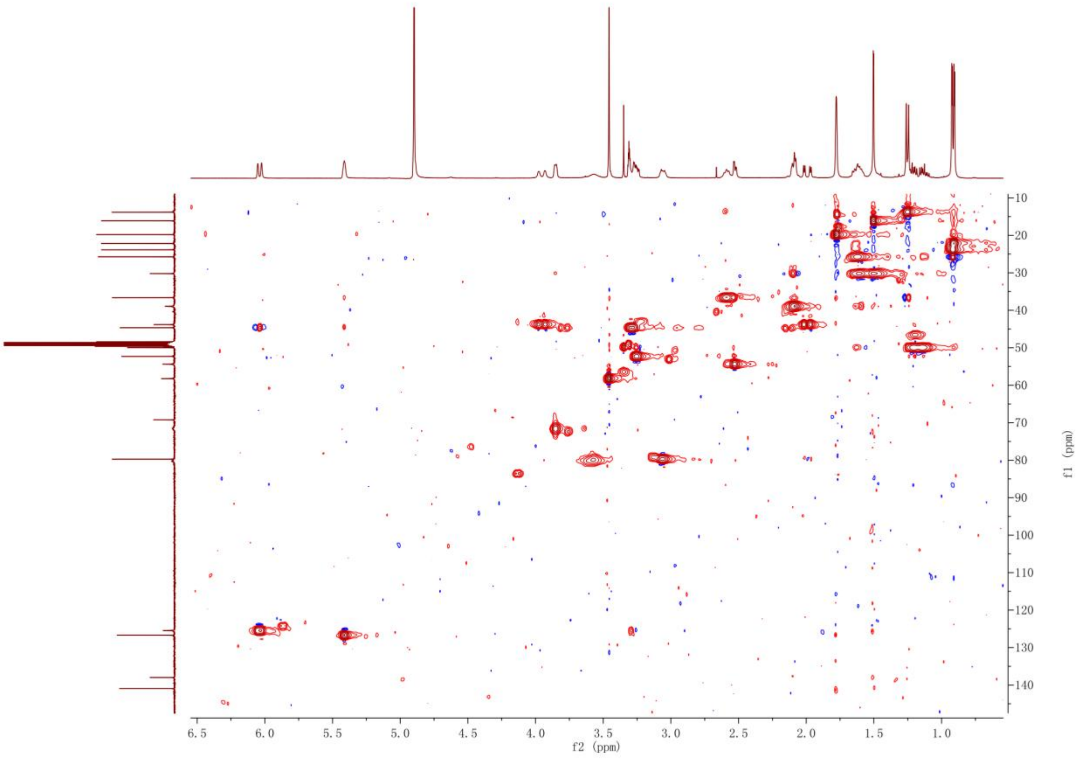

HMBC for compound **6** (in CD_3OD, 400 MHz)

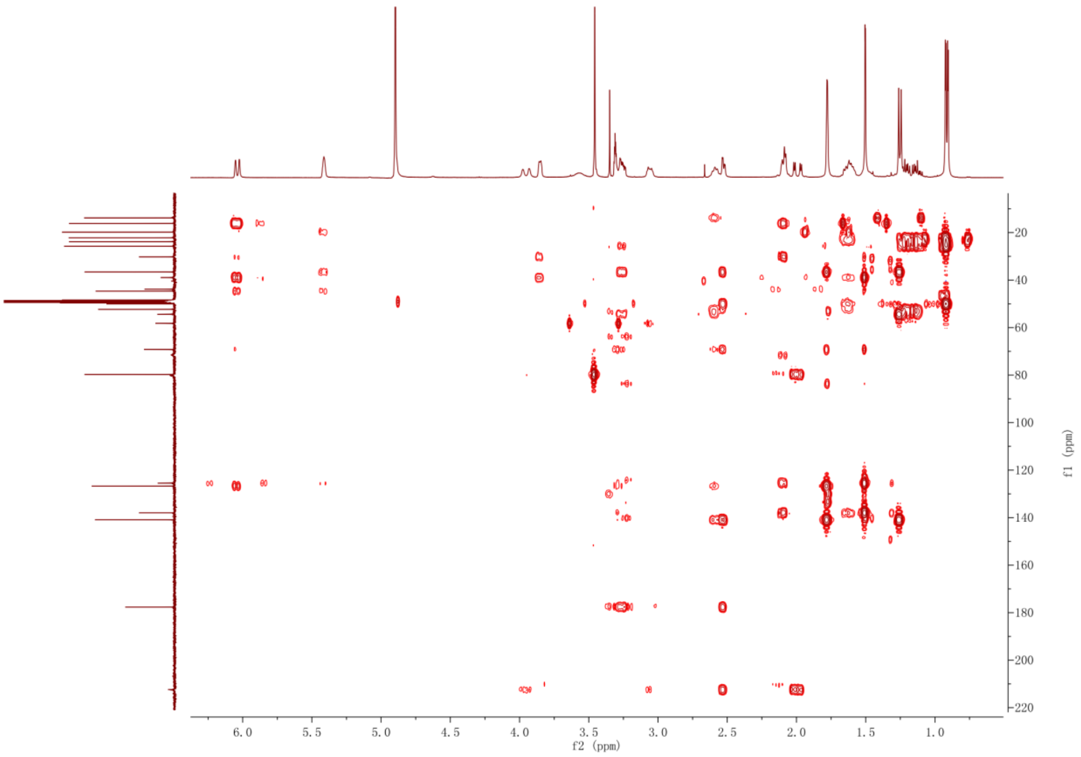

^{1}H–^{1}H COSY for compound **6** (in CD_3OD, 400 MHz)

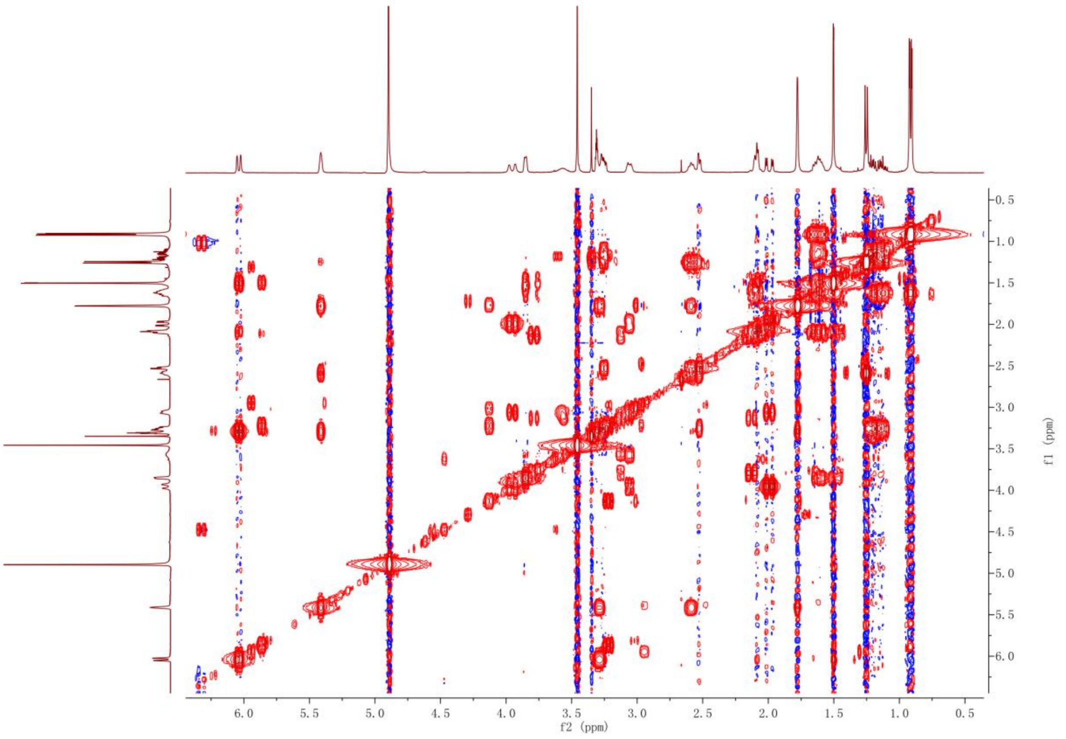

NOESY for compound **6** (in CD_3OD, 400 MHz)

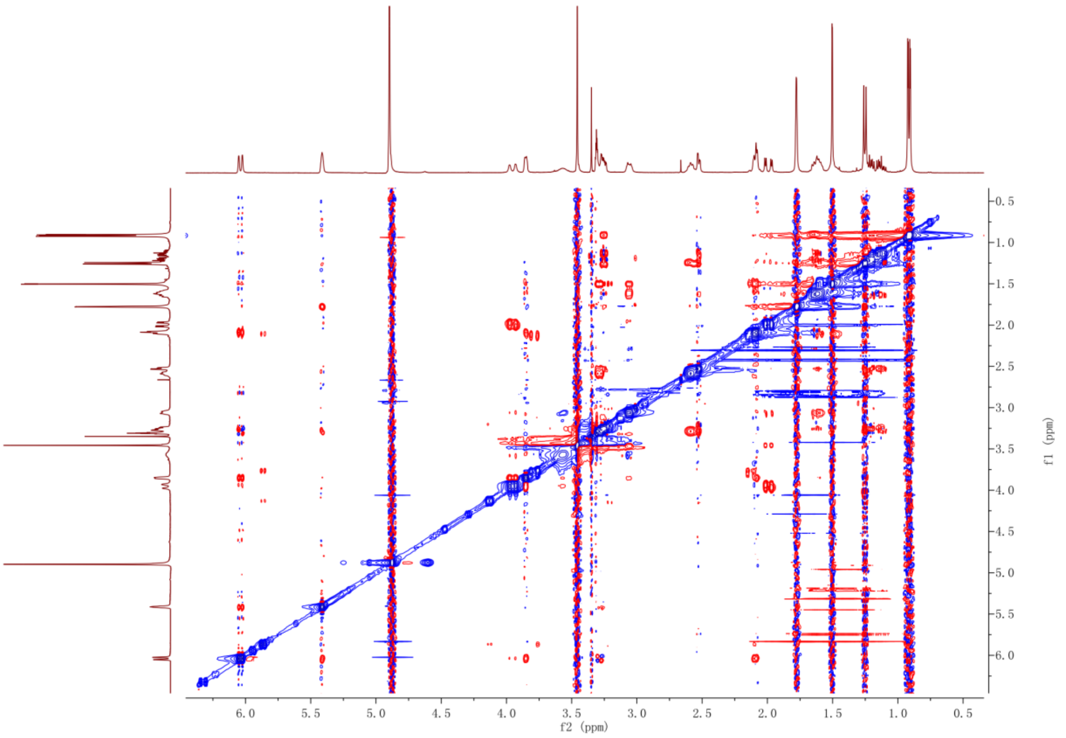

HRESIMS for compound **7**

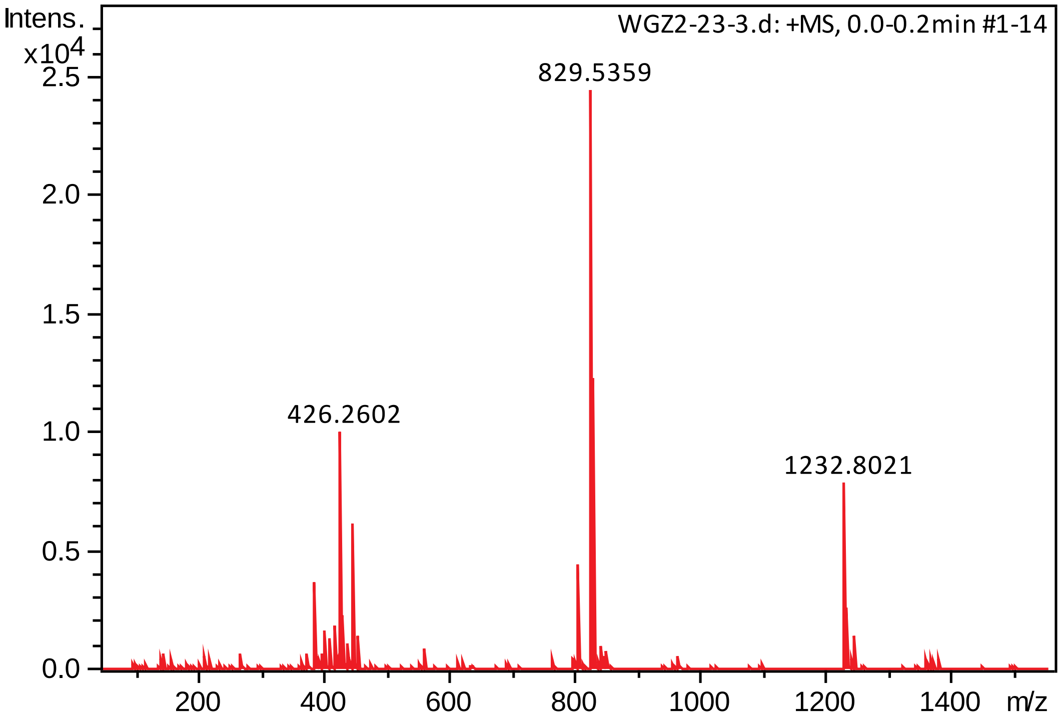

UV spectrum for compound **7**

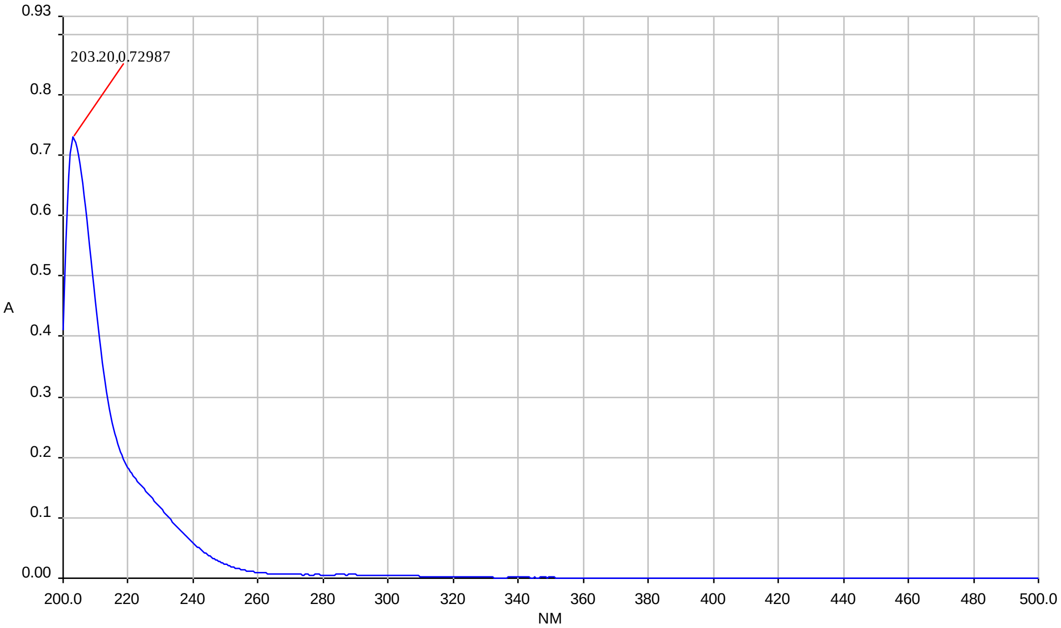

IR spectrum for compound **7**

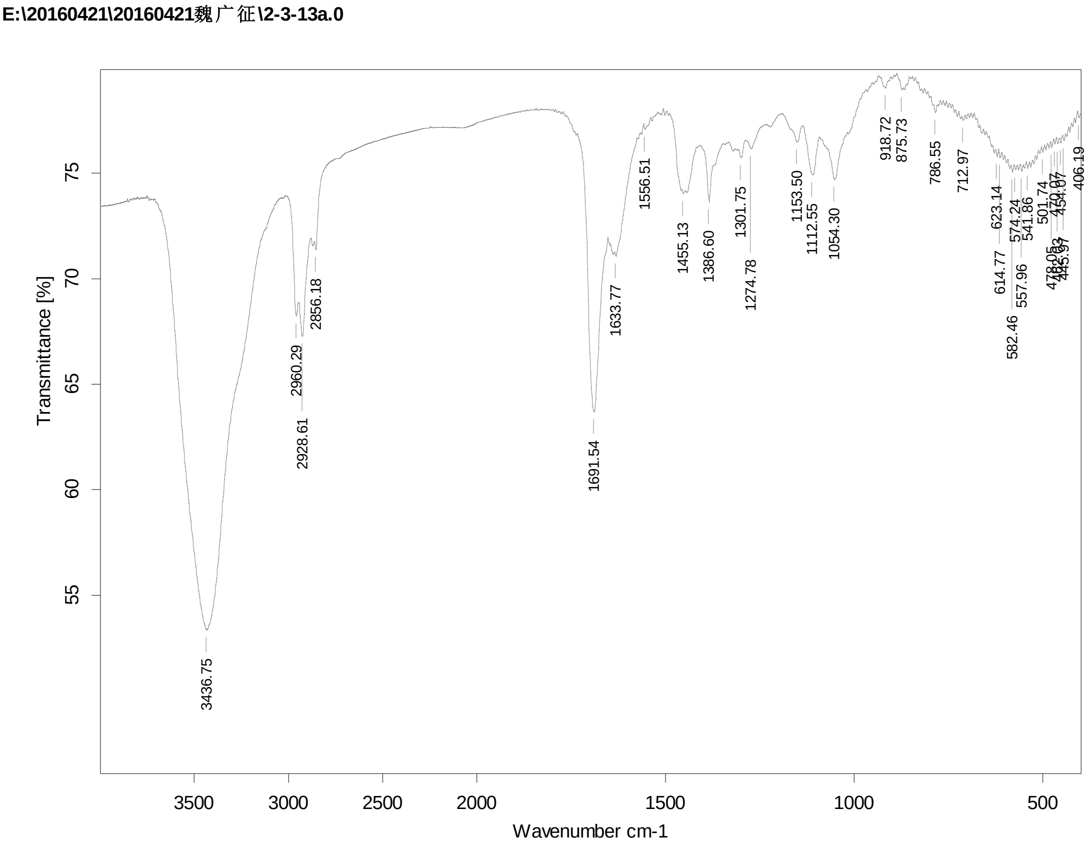

^{1}H NMR for compound **7** (in DMSO, 400 MHz)

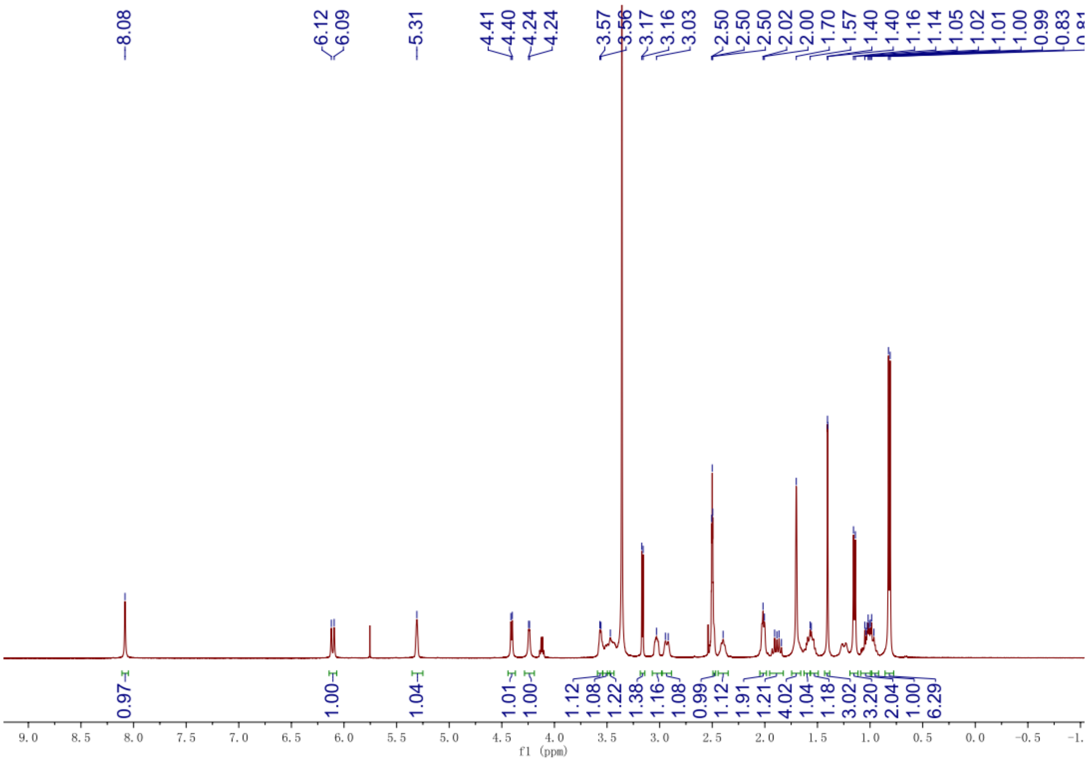

^{13}C NMR for compound **7** (in DMSO, 100 MHz)

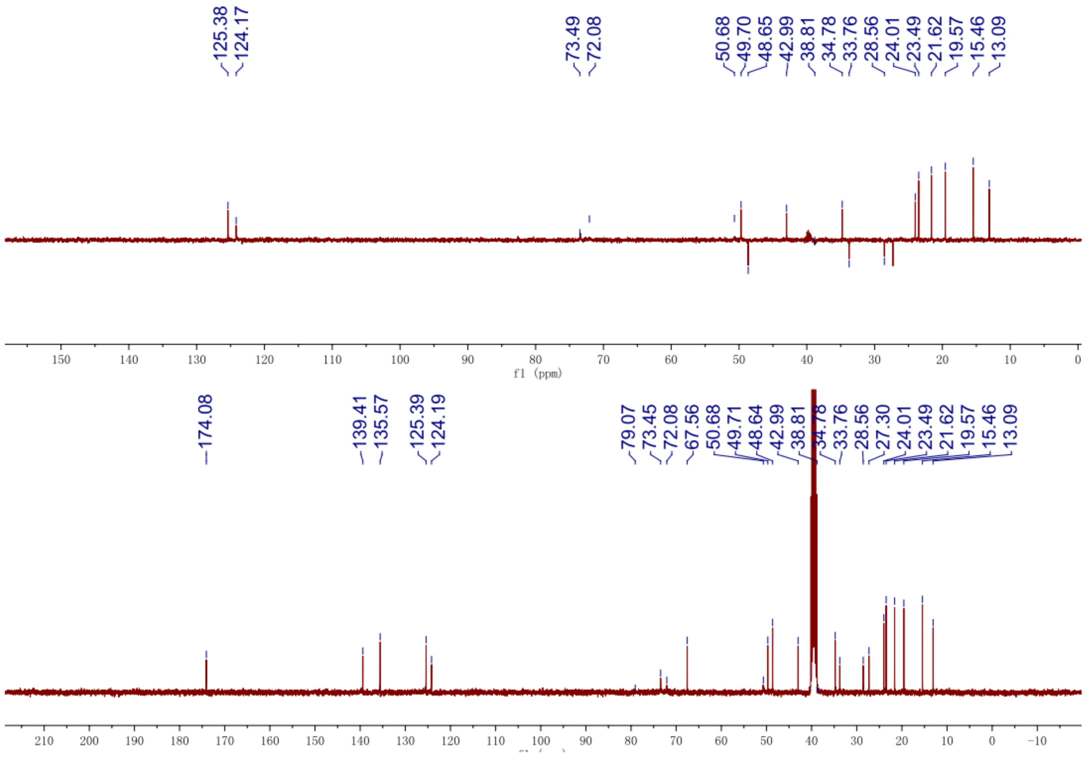

HSQC for compound **7** (in DMSO, 400 MHz)

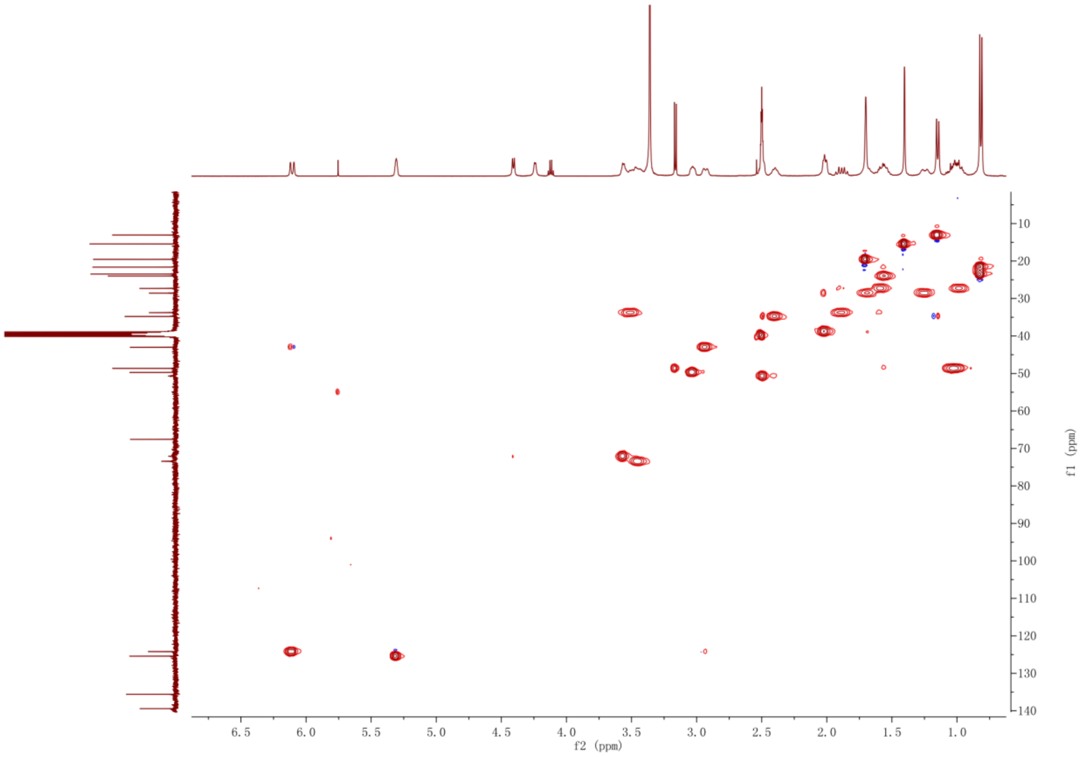

HMBC for compound **7** (in DMSO, 400 MHz)

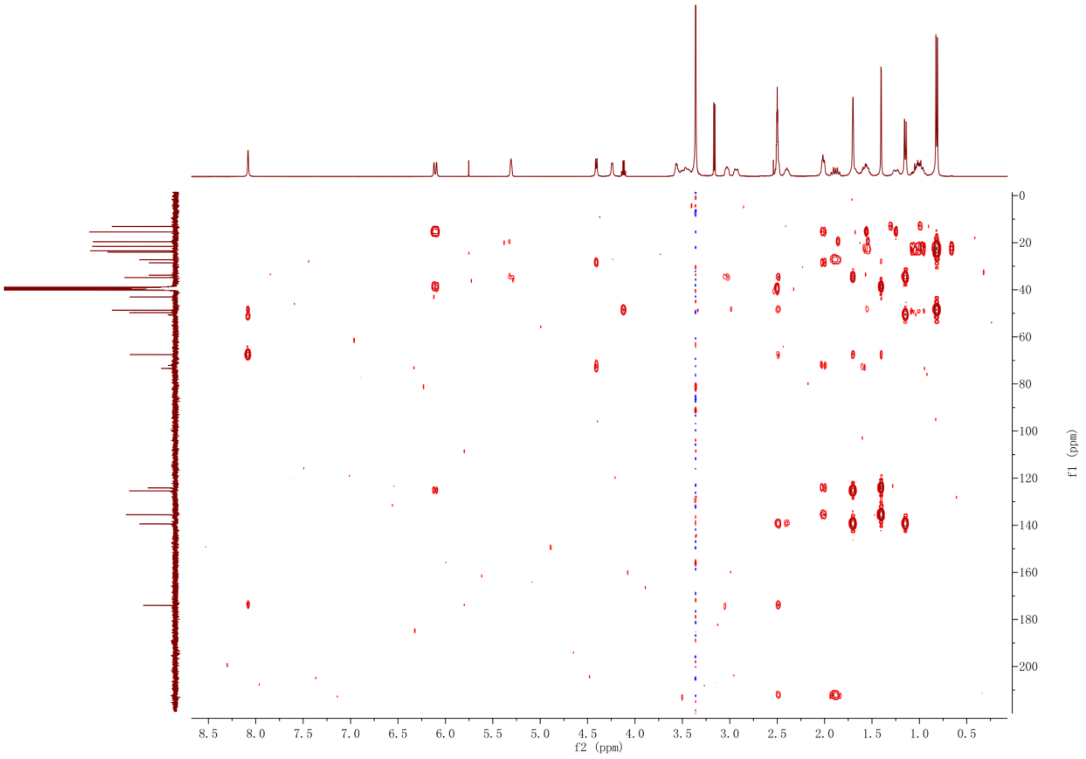

^{1}H–^{1}H COSY for compound **7** (in DMSO, 400 MHz)

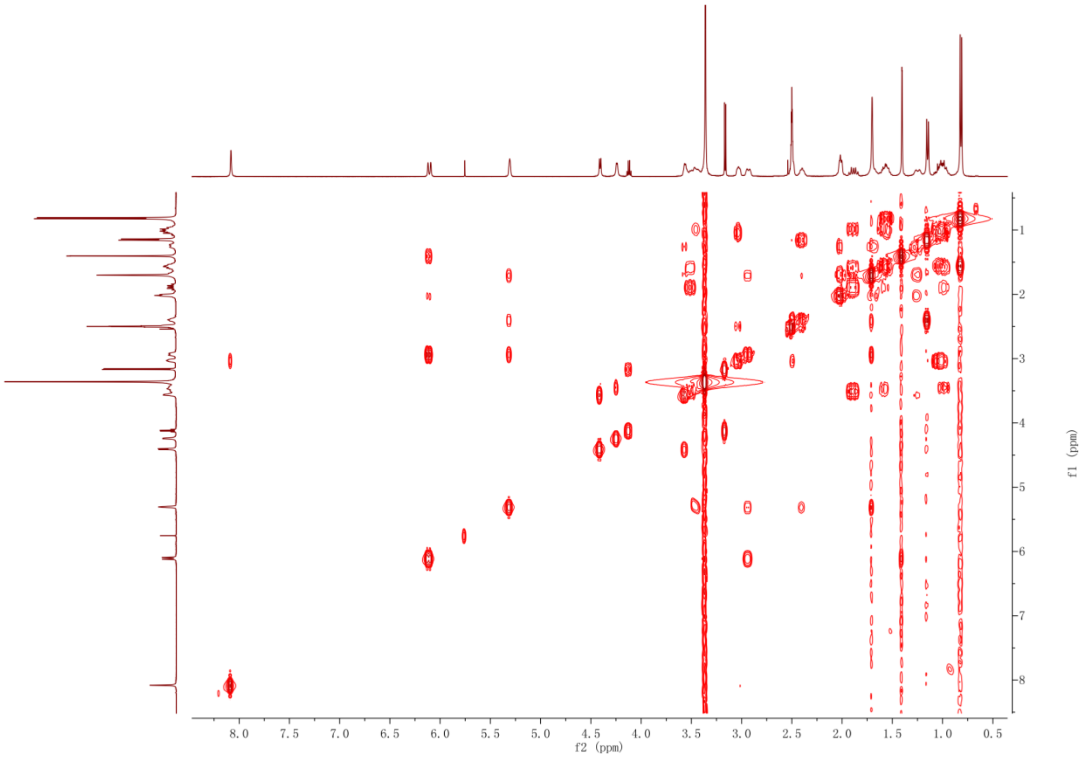

NOESY for compound **7** (in DMSO, 400 MHz)

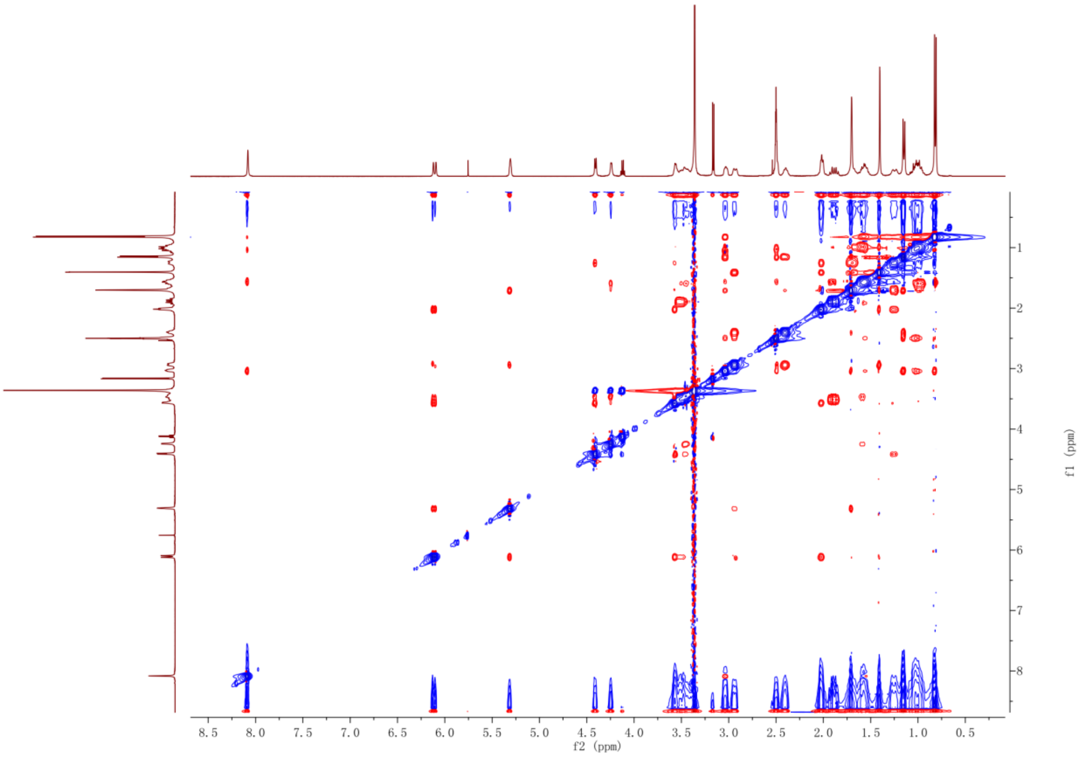

HRESIMS for compound **8**

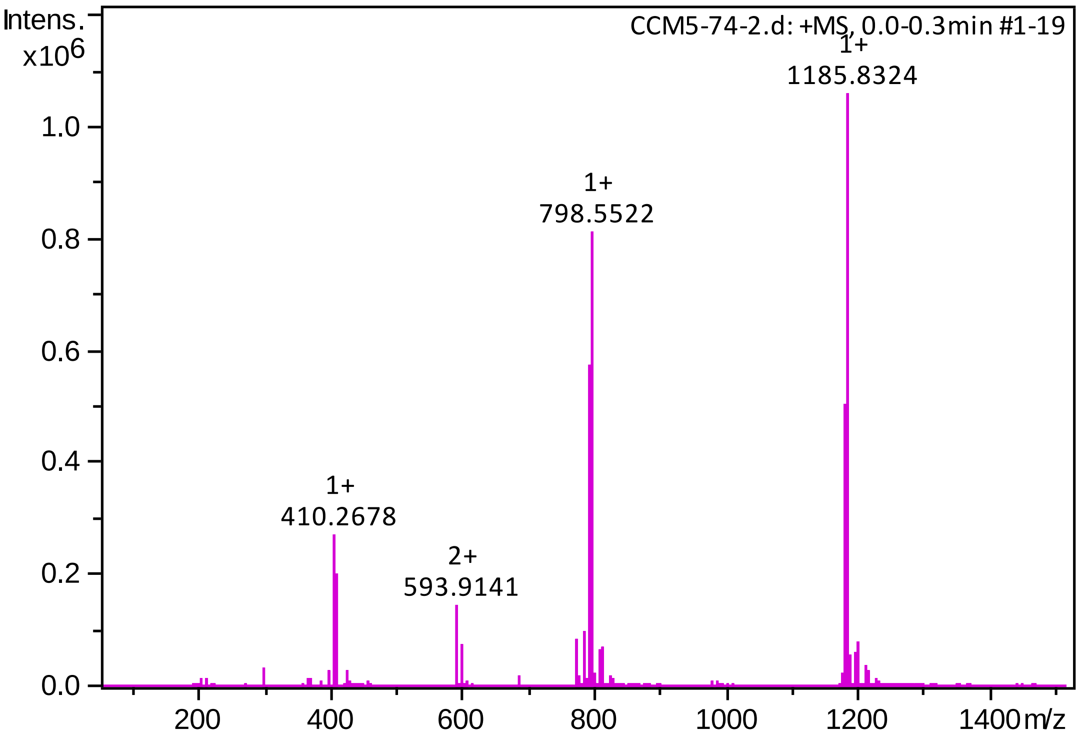

UV spectrum for compound **8**

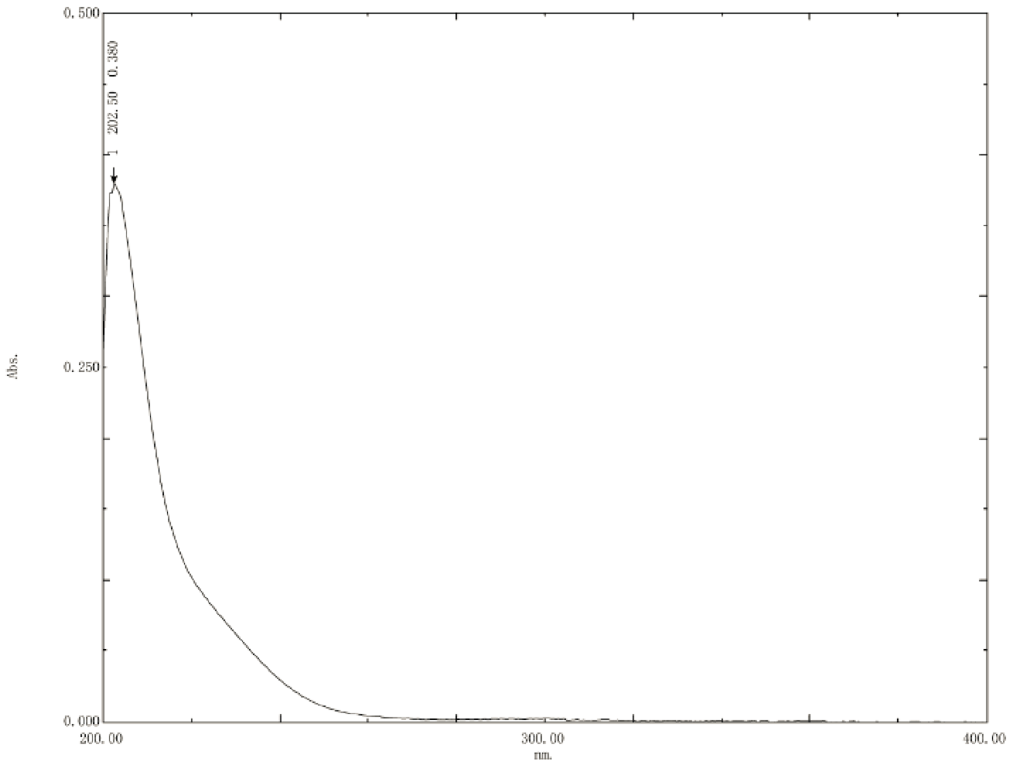

IR spectrum for compound **8**

E:\同济医学院\张勇慧\20150128\AF5-74-2.0

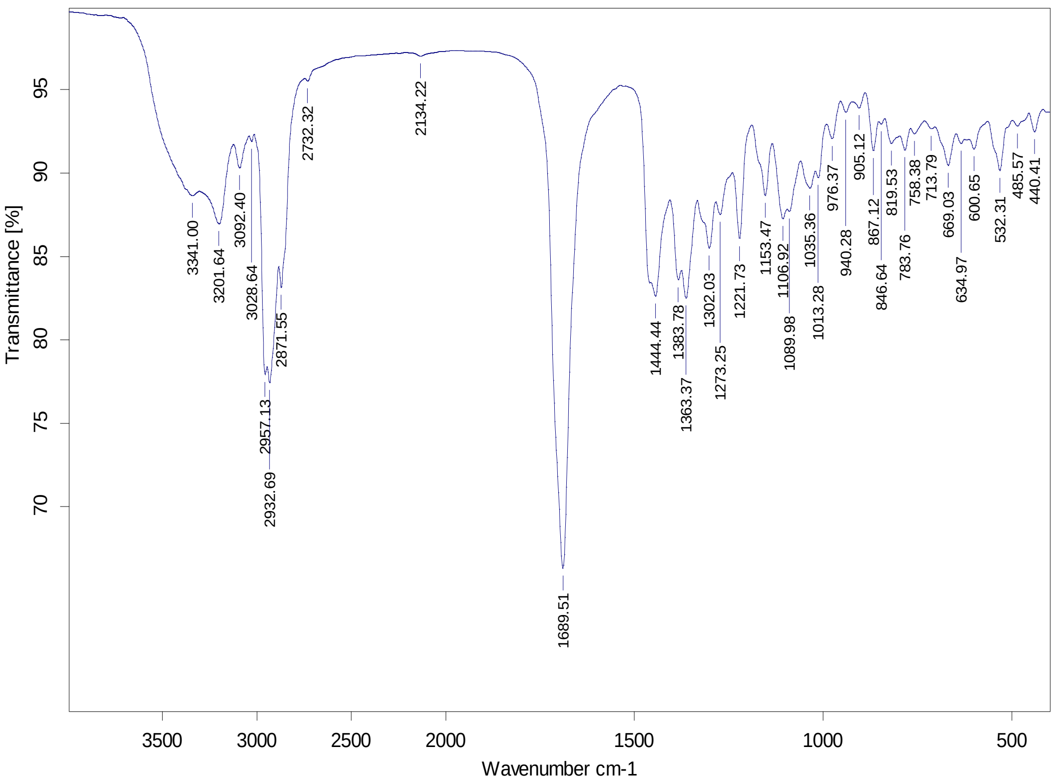

^{1}H NMR for compound **8** (in CD_3OD, 400 MHz)

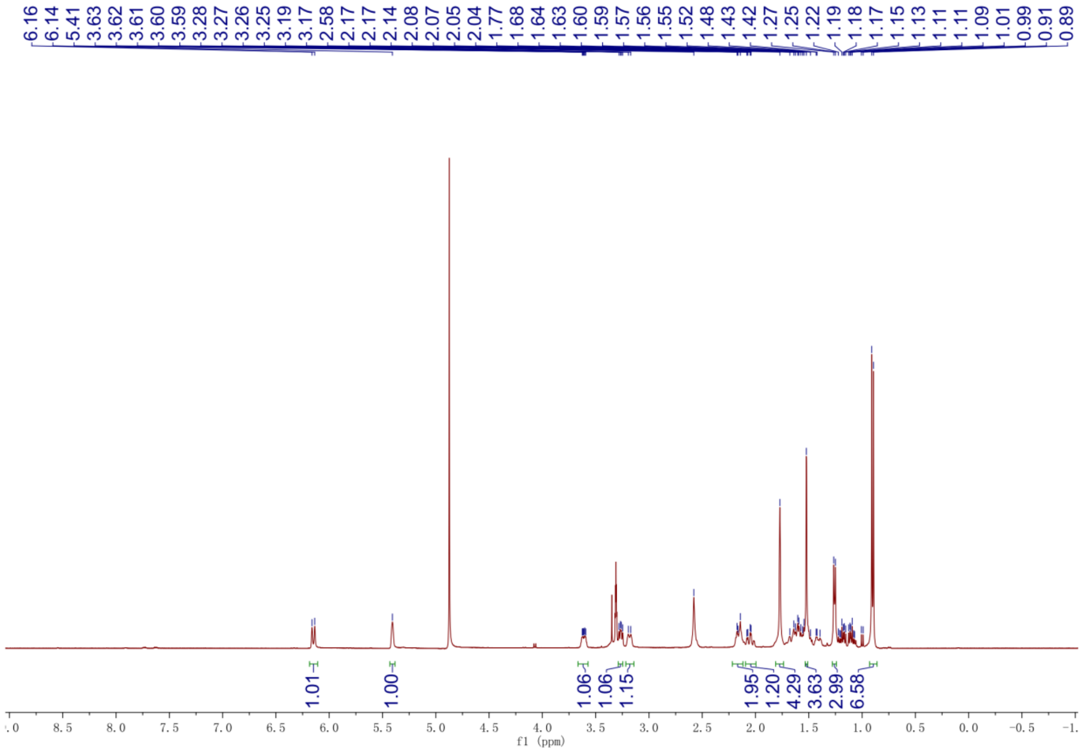

^{13}C NMR for compound **8** (in CD_3OD, 100 MHz)

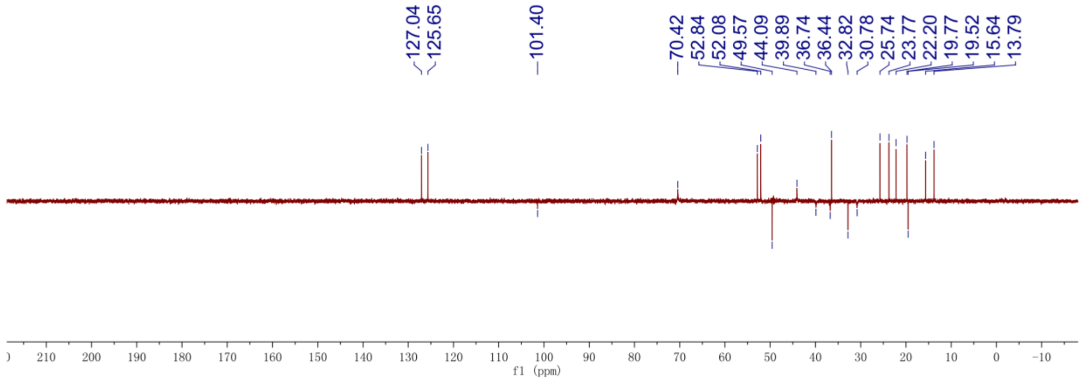

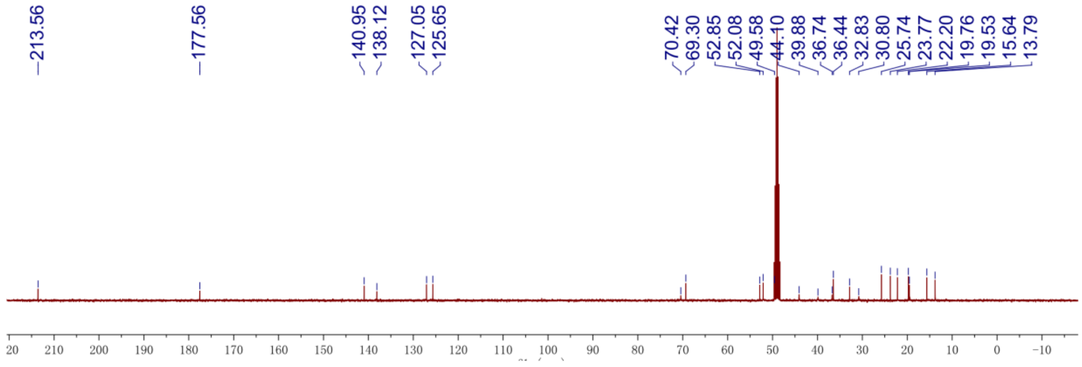

HSQC for compound **8** (in CD_3OD, 400 MHz)

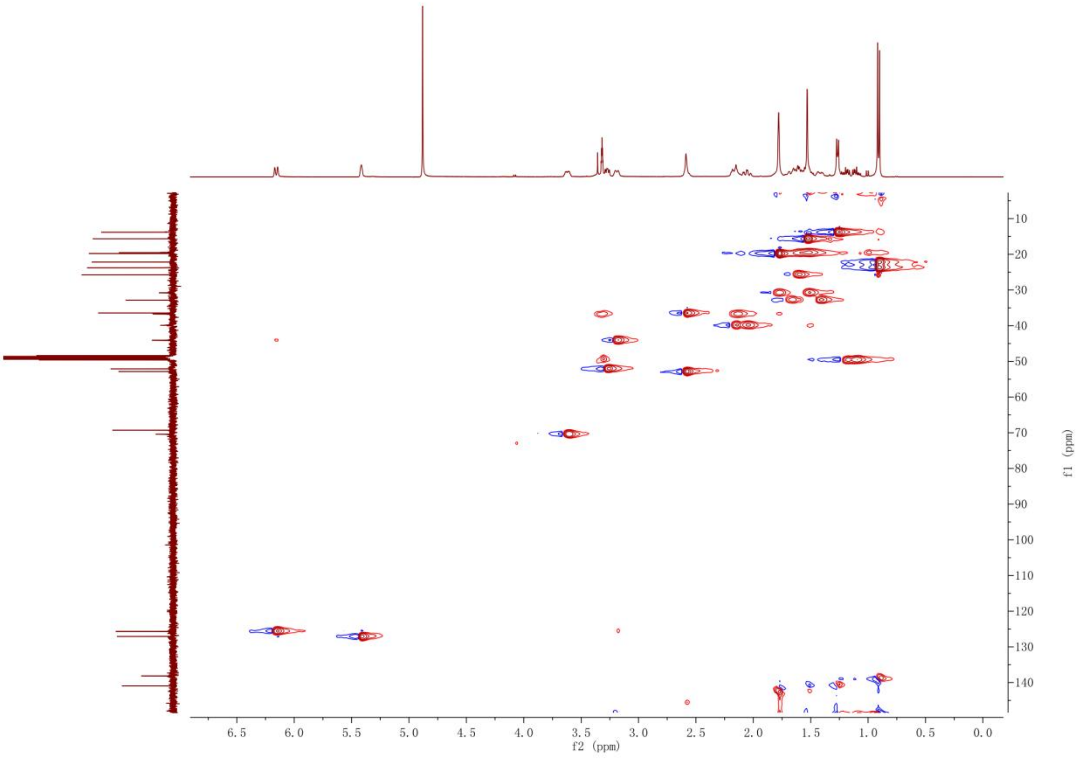

HMBC for compound **8** (in CD_3OD, 400 MHz)

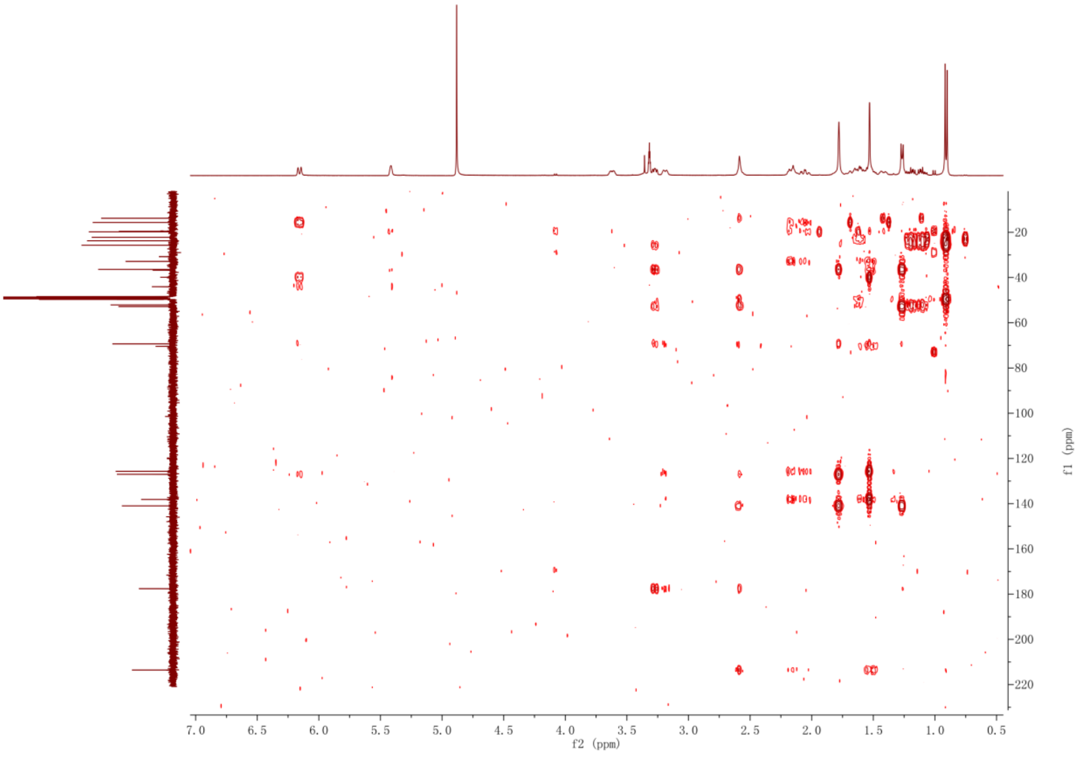

^{1}H–^{1}H COSY for compound **8** (in CD_3OD, 400 MHz)

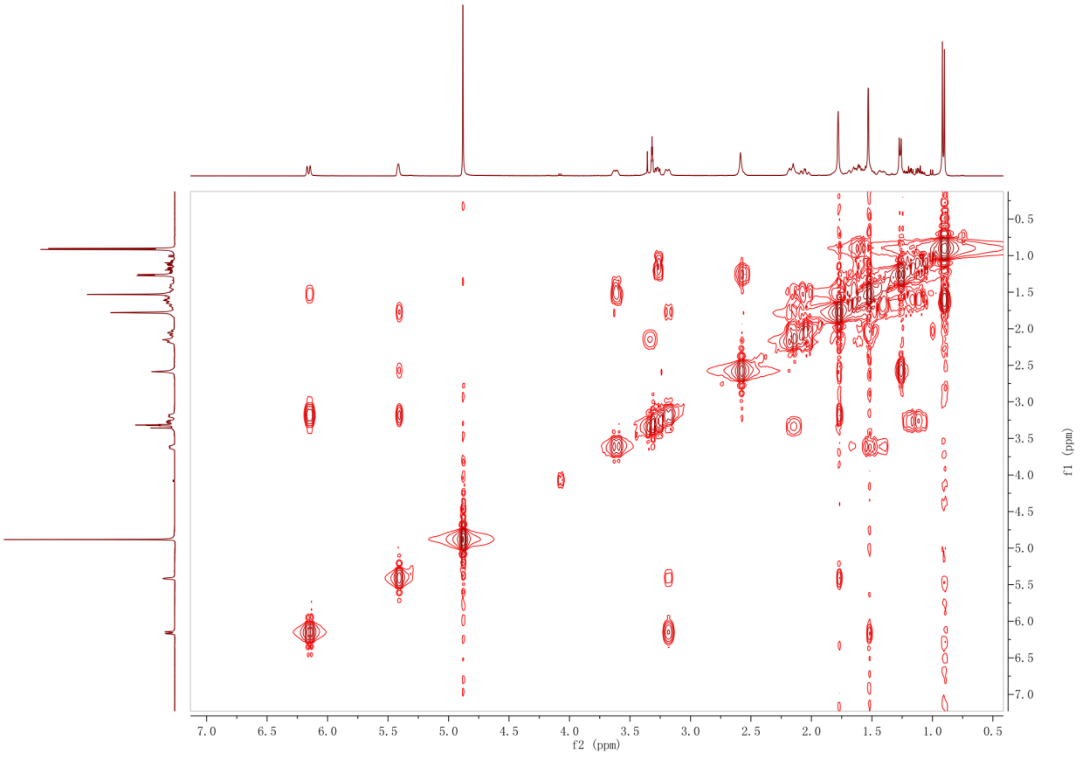

NOESY for compound **8** (in CD_3OD, 400 MHz)

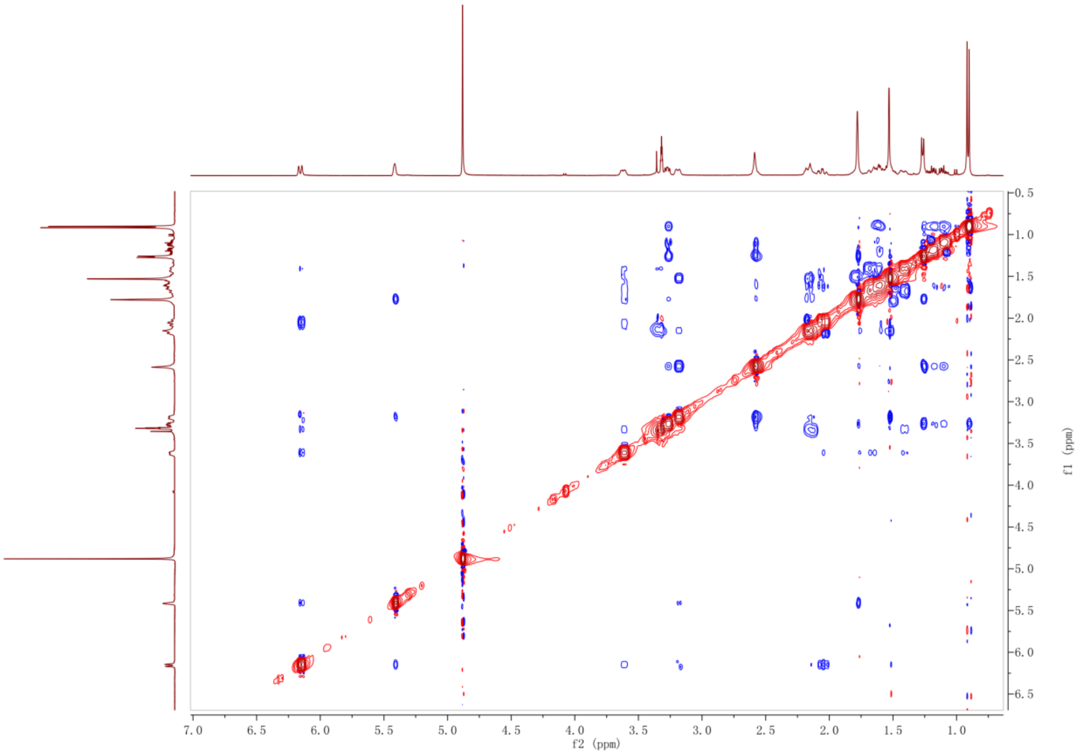

HRESIMS for compound **9**

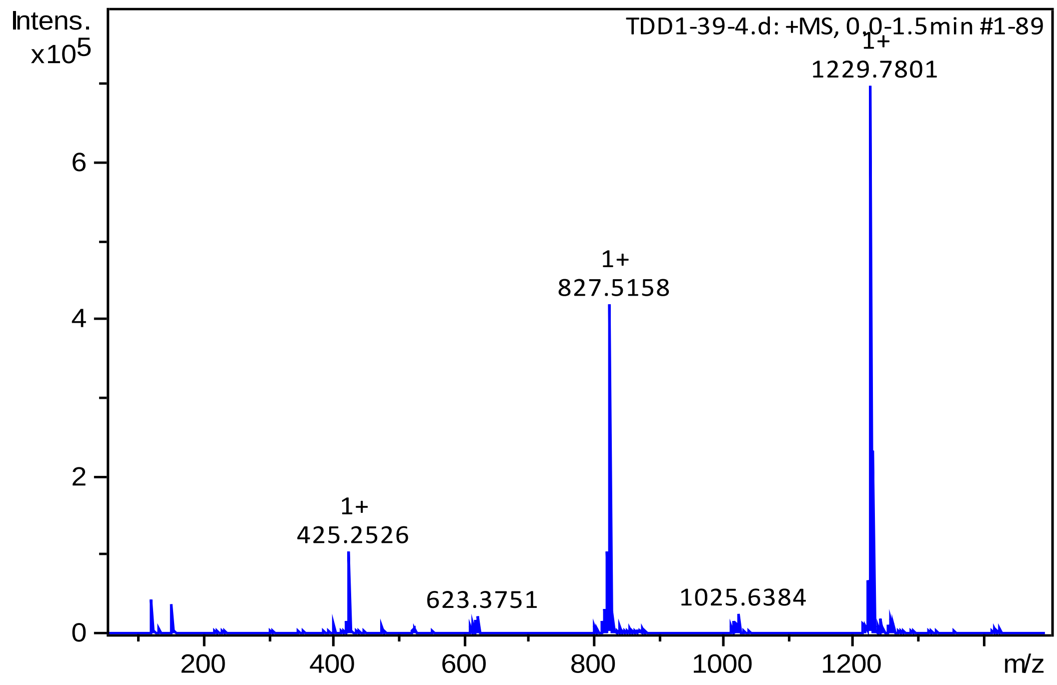

UV spectrum for compound **9**

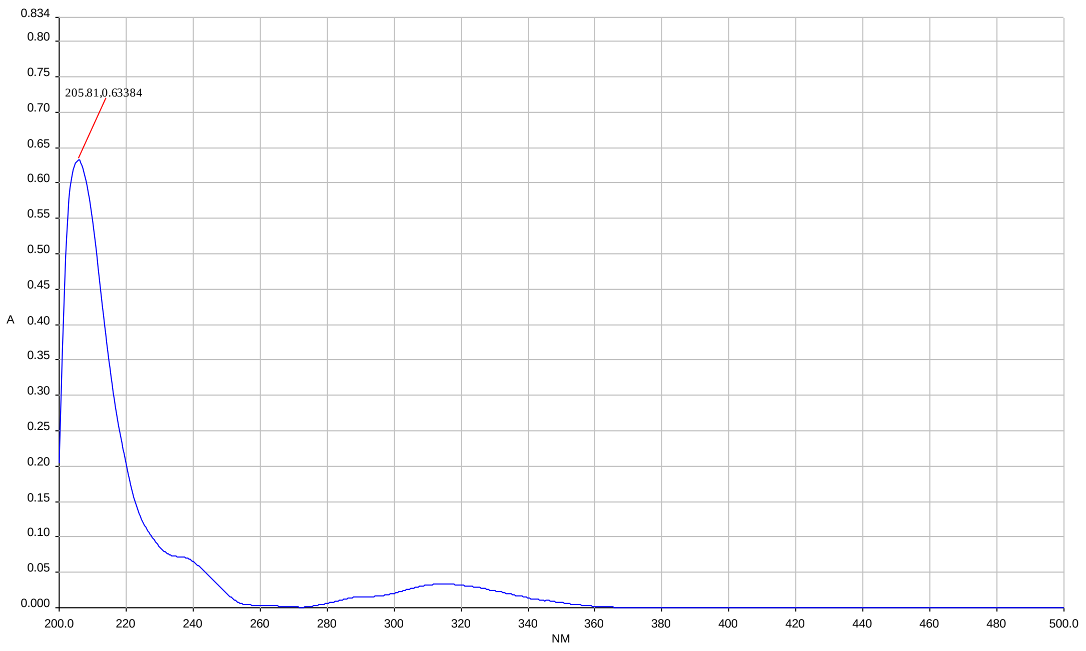

IR spectrum for compound **9**

E:\20160428\20160428谭冬冬\TDD1-39-4.0

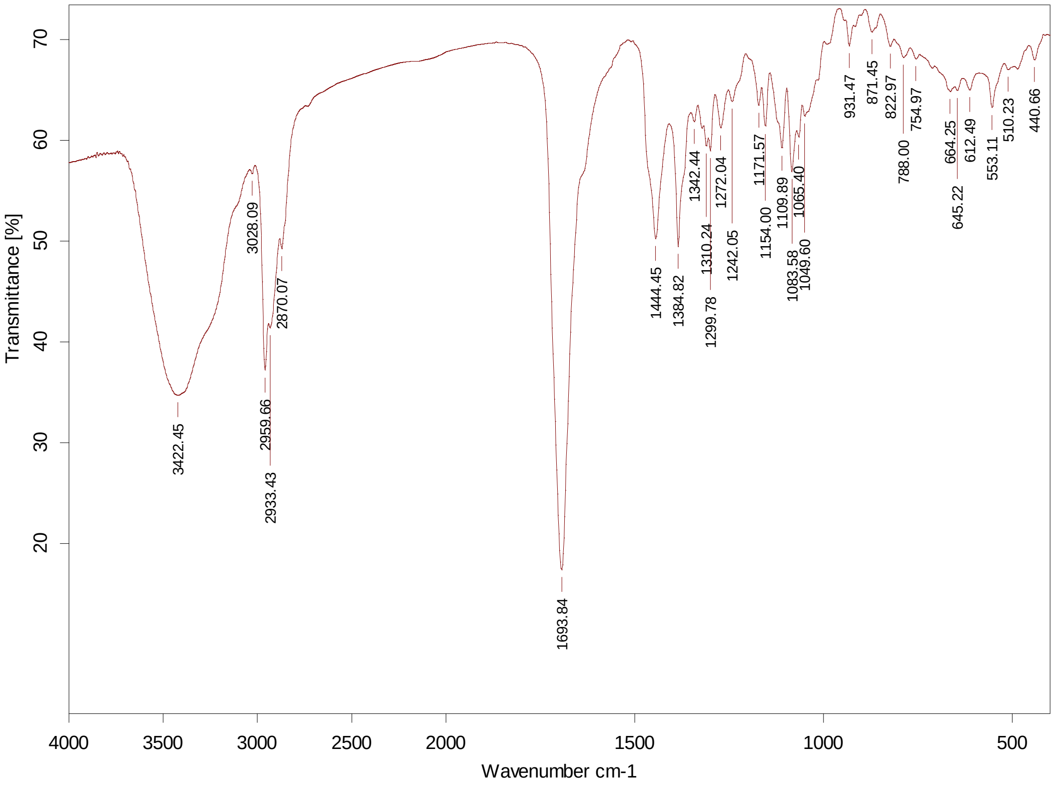

^1H NMR for compound **9** (in CD_3OD, 400 MHz)

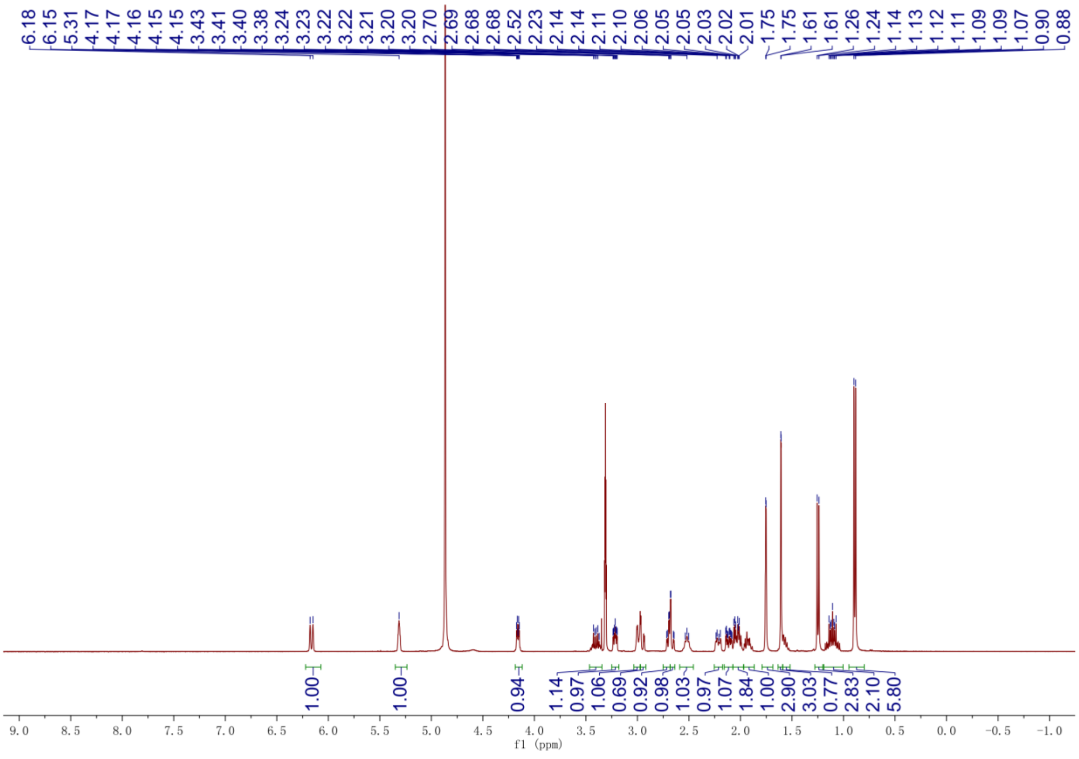

^{13}C NMR for compound **9** (in CD_3OD, 100 MHz)

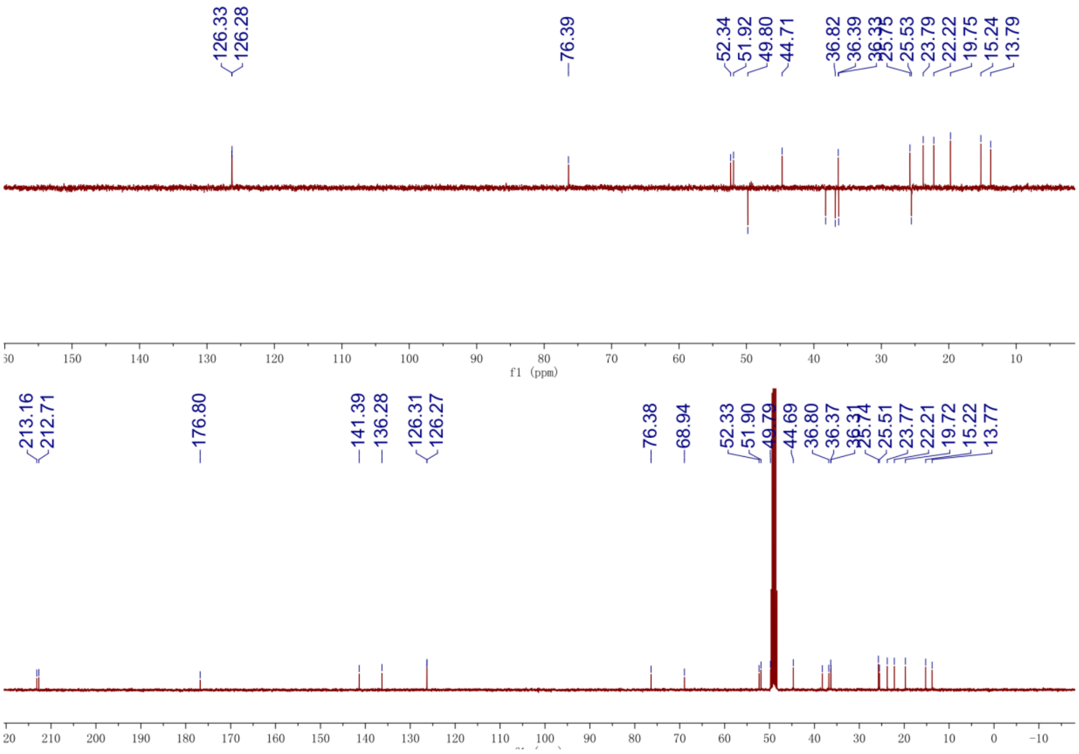

HSQC for compound **9** (in CD_3OD, 400 MHz)

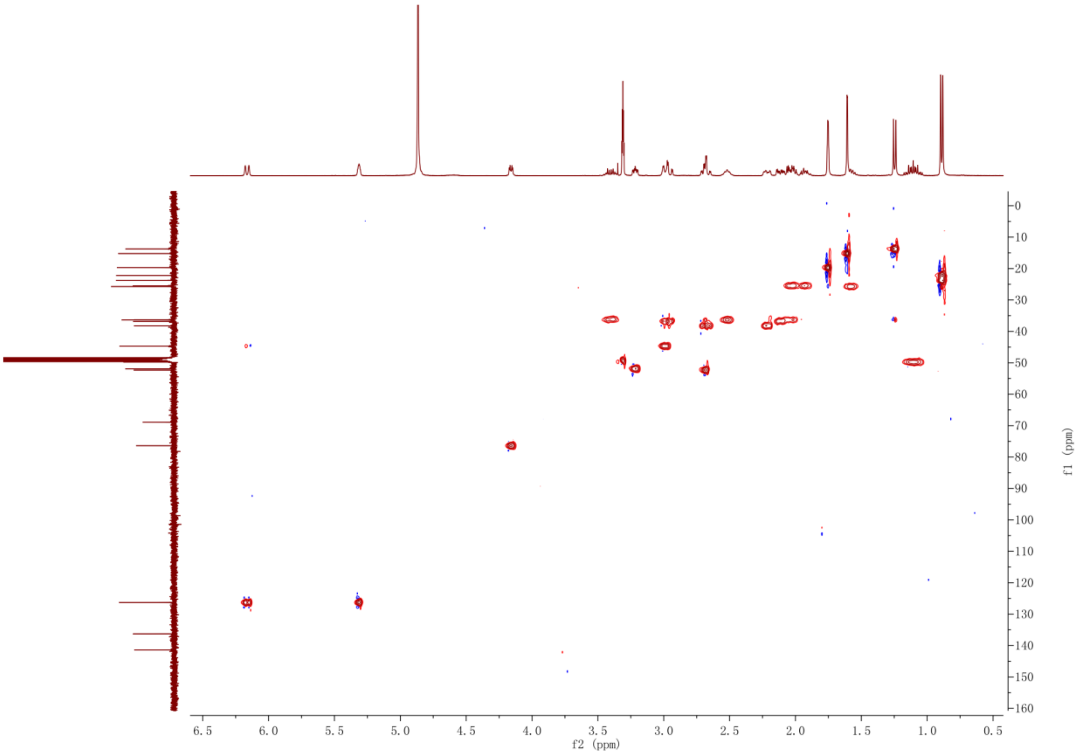

HMBC for compound **9** (in CD_3OD, 400 MHz)

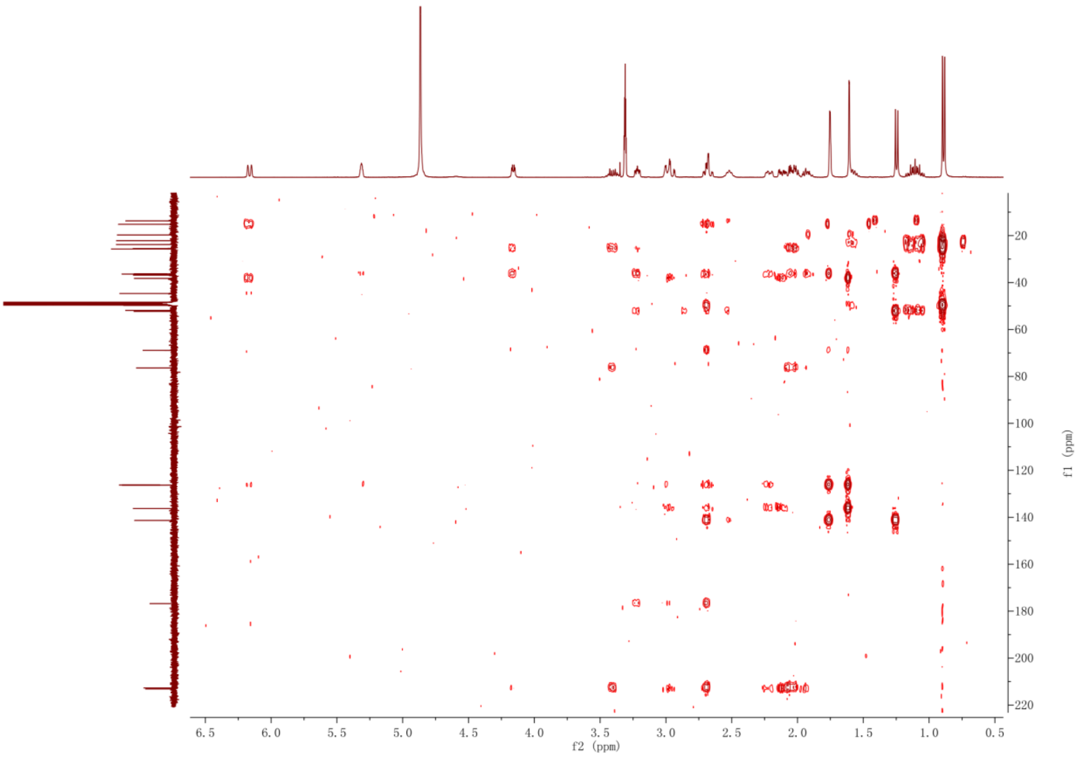

^{1}H–^{1}H COSY for compound **9** (in CD_3OD, 400 MHz)

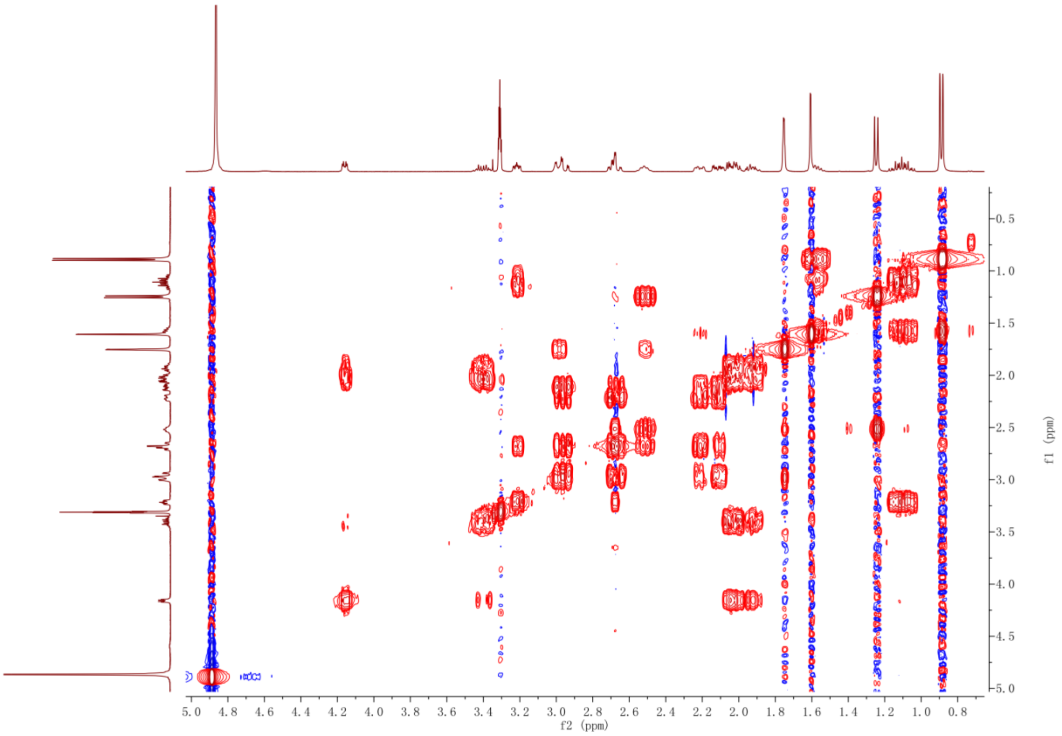

NOESY for compound **9** (in CD_3OD, 400 MHz)

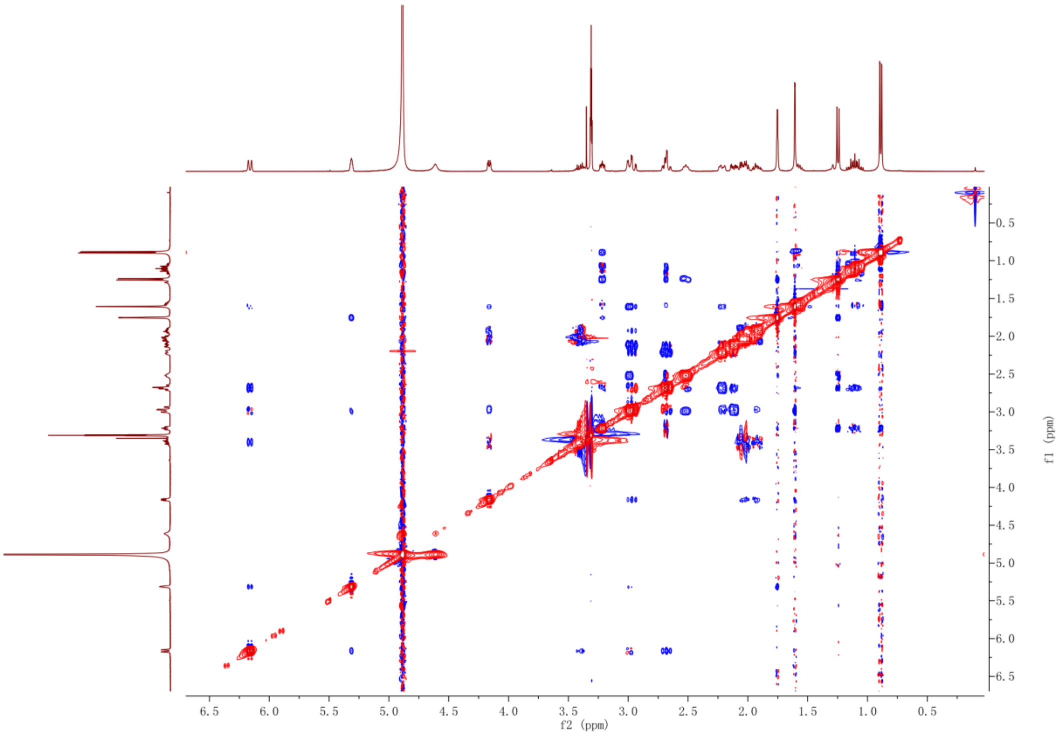

HRESIMS for compound **10**

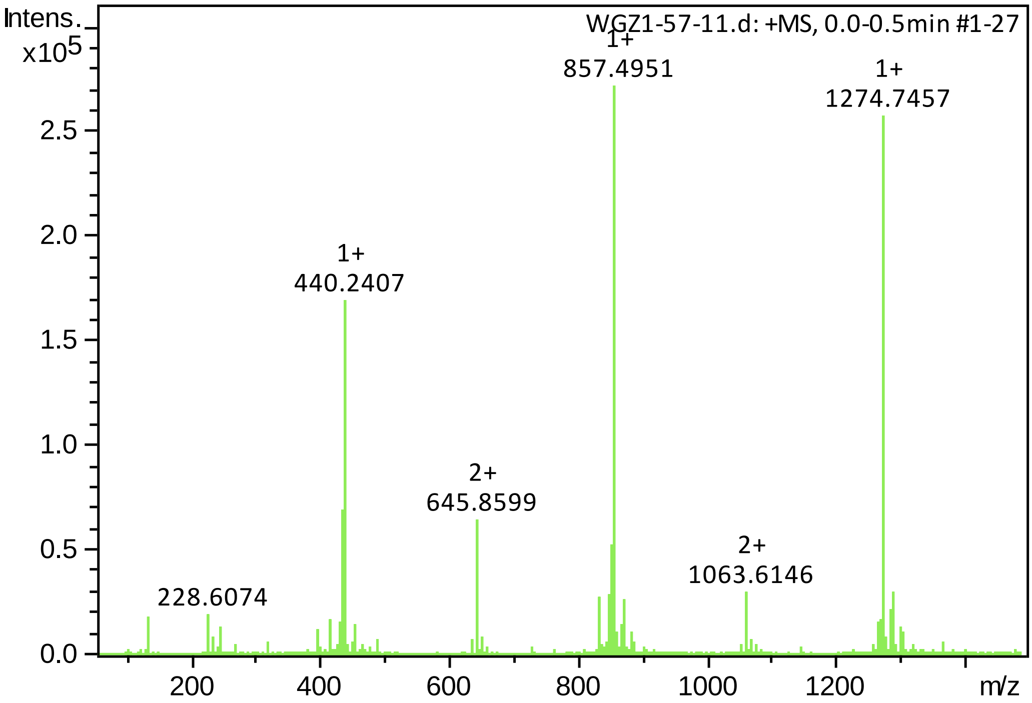

UV spectrum for compound **10**

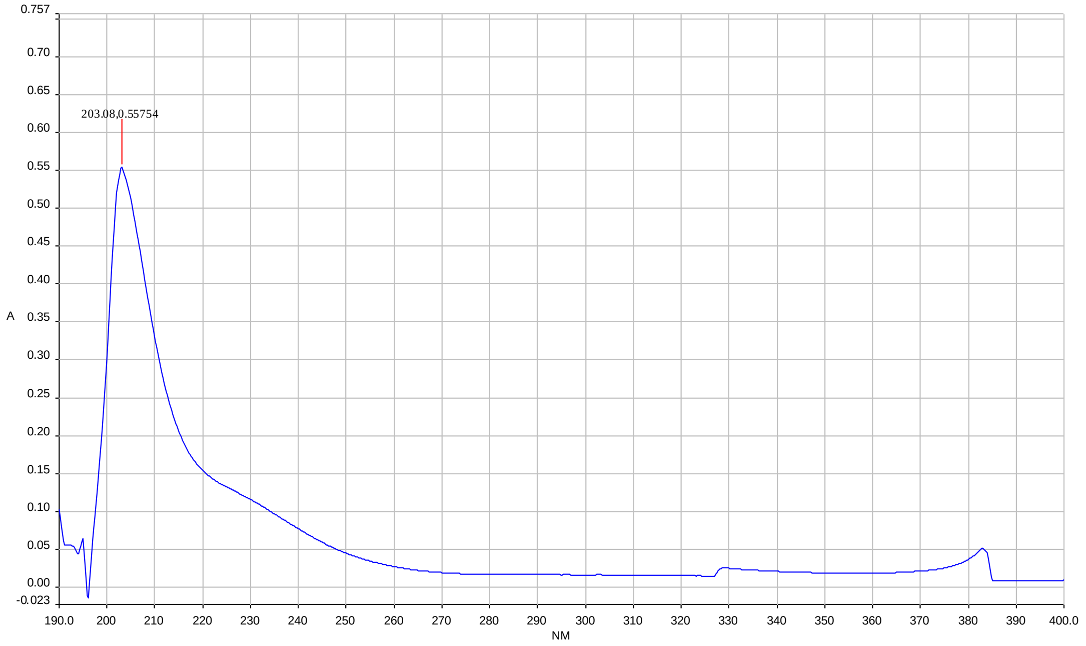

IR spectrum for compound **10**

E:\20160802\魏广正\1-57-11.0

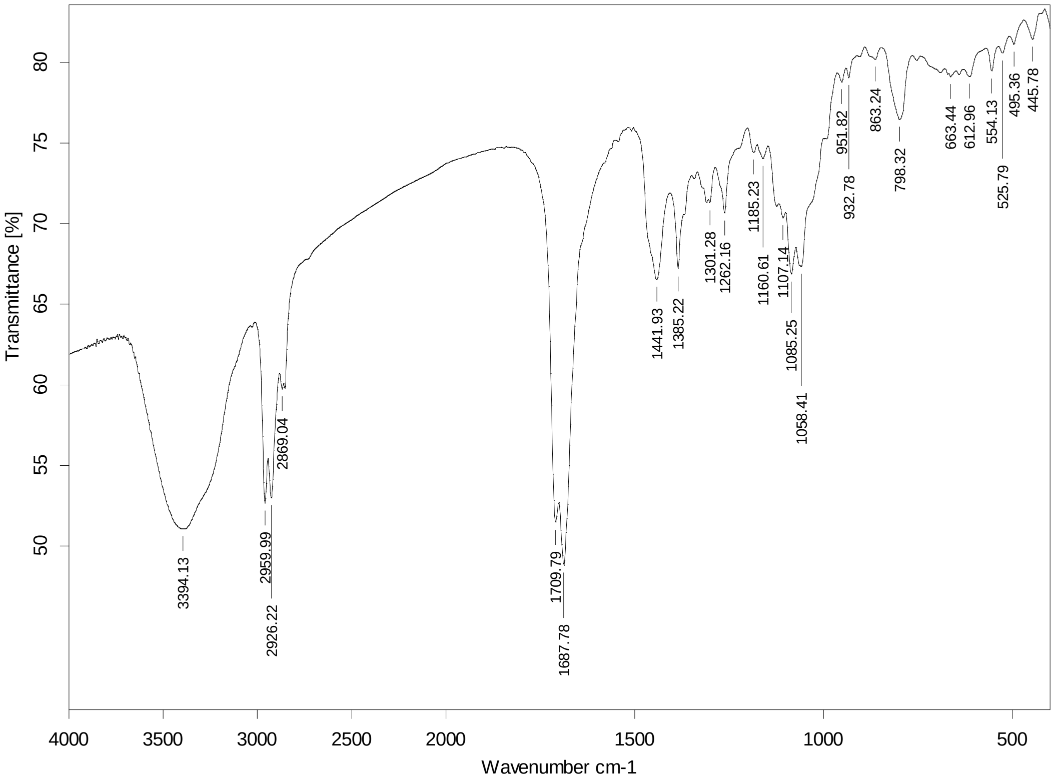

^{1}H NMR for compound **10** (in CD_3OD, 400 MHz)

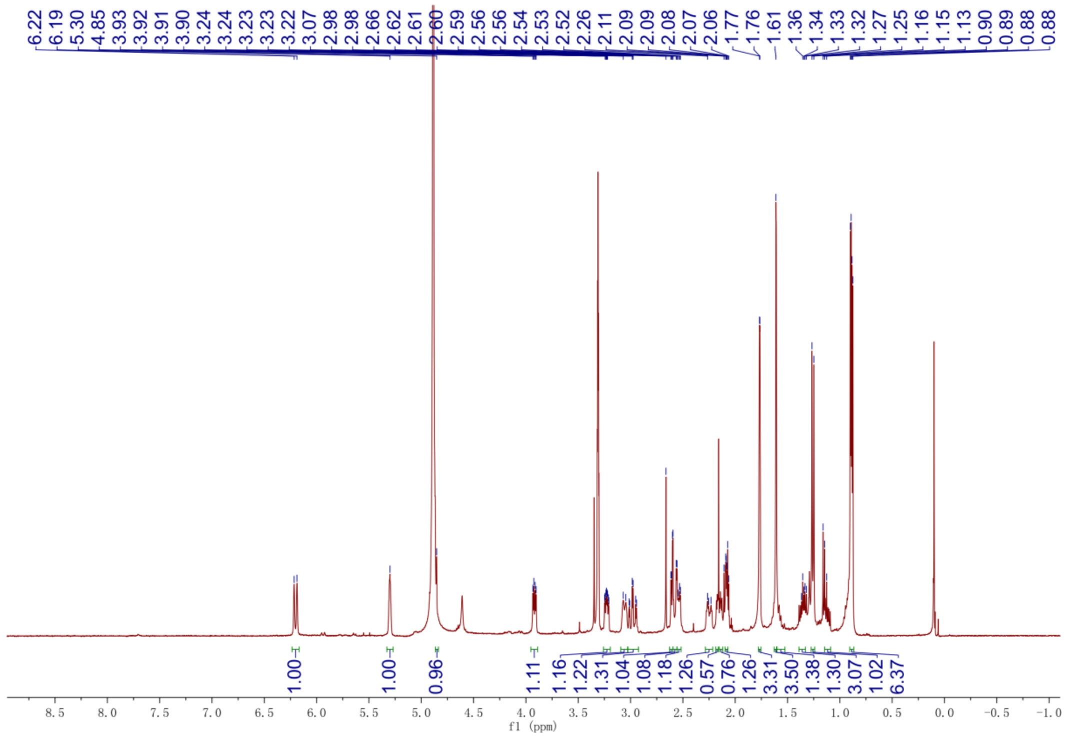

^{13}C NMR for compound **10** (in CD_3OD, 100 MHz)

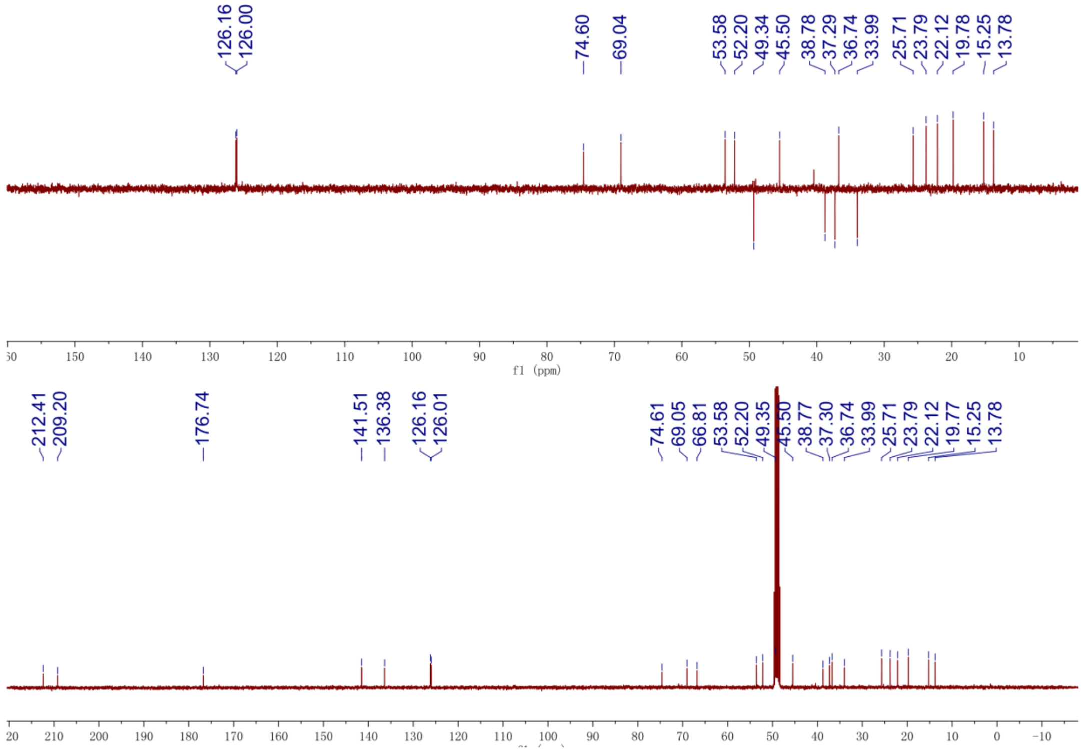

HSQC for compound **10** (in CD_3OD, 400 MHz)

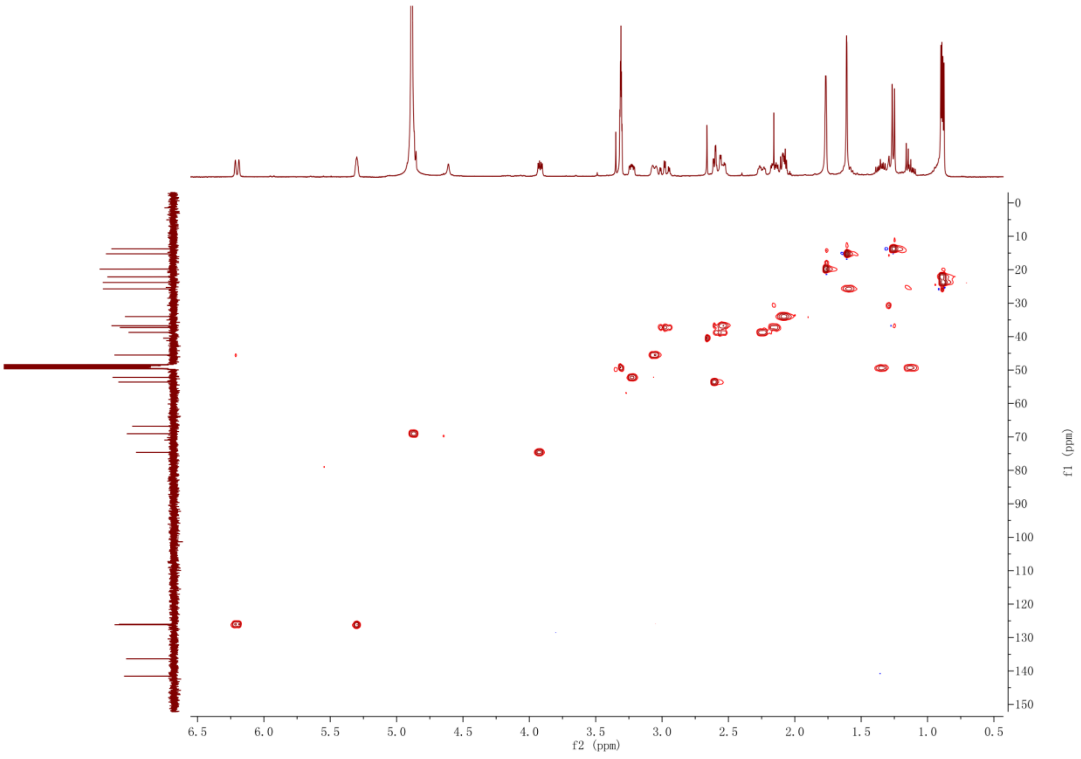

HMBC for compound **10** (in CD_3OD, 400 MHz)

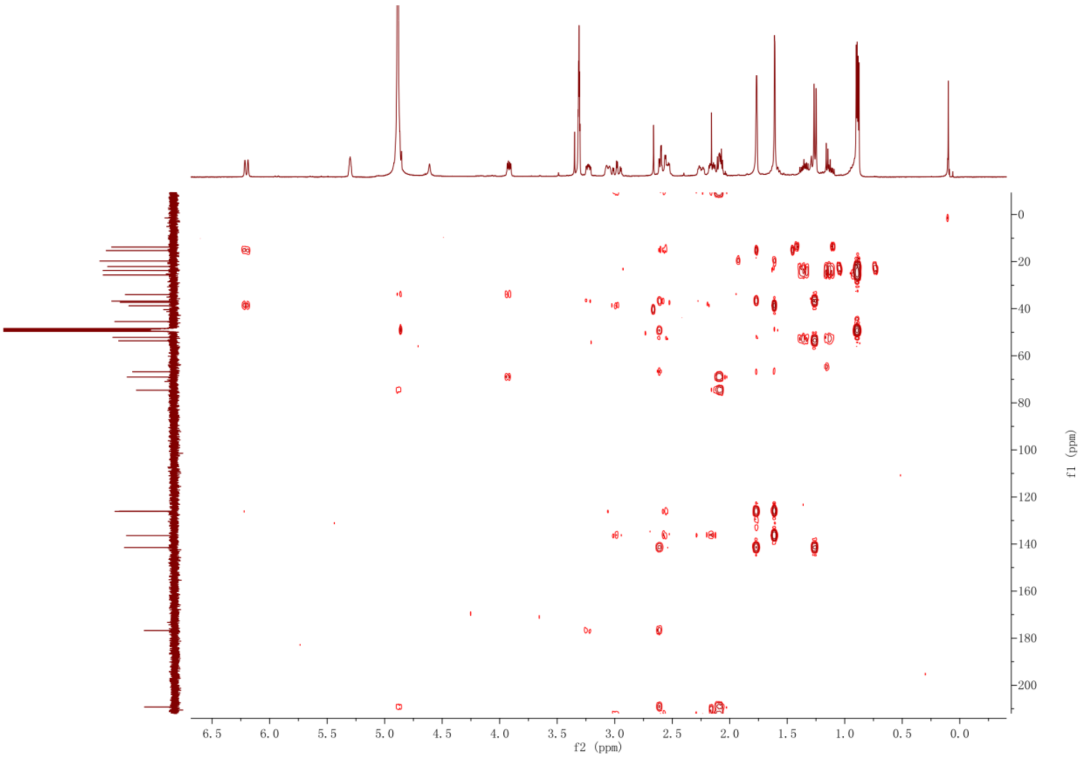

^1H–^1H COSY for compound **10** (in CD_3OD, 400 MHz)

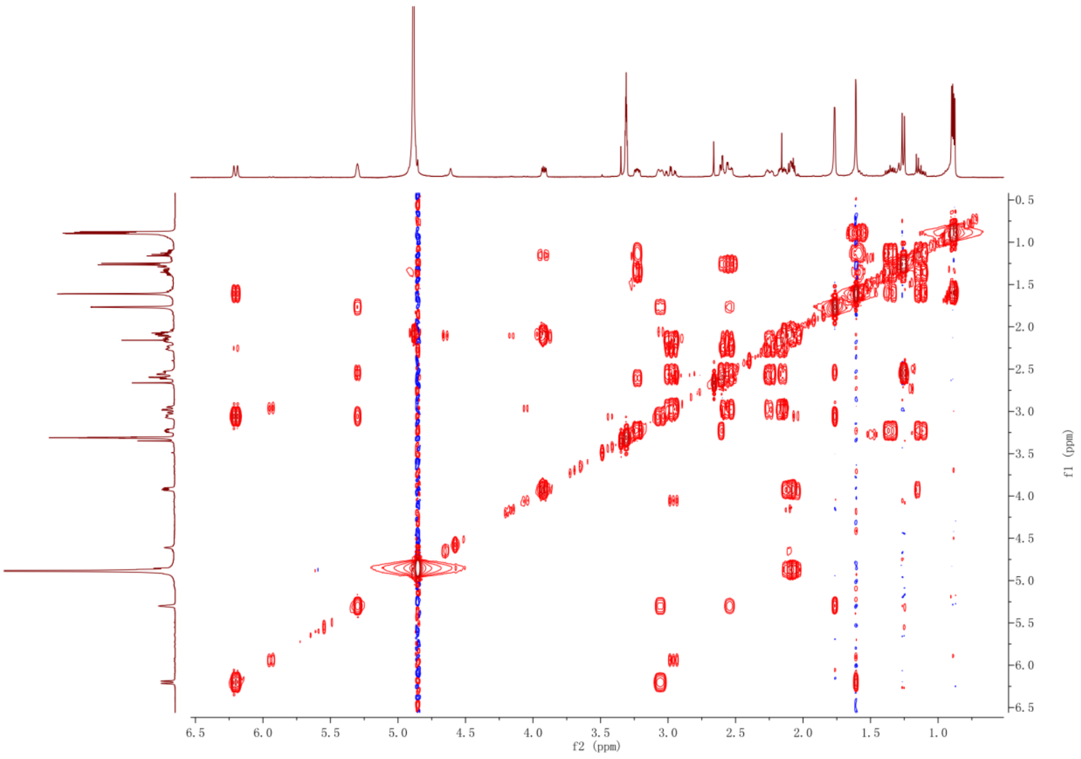

NOESY for compound **10** (in CD_3OD, 400 MHz)

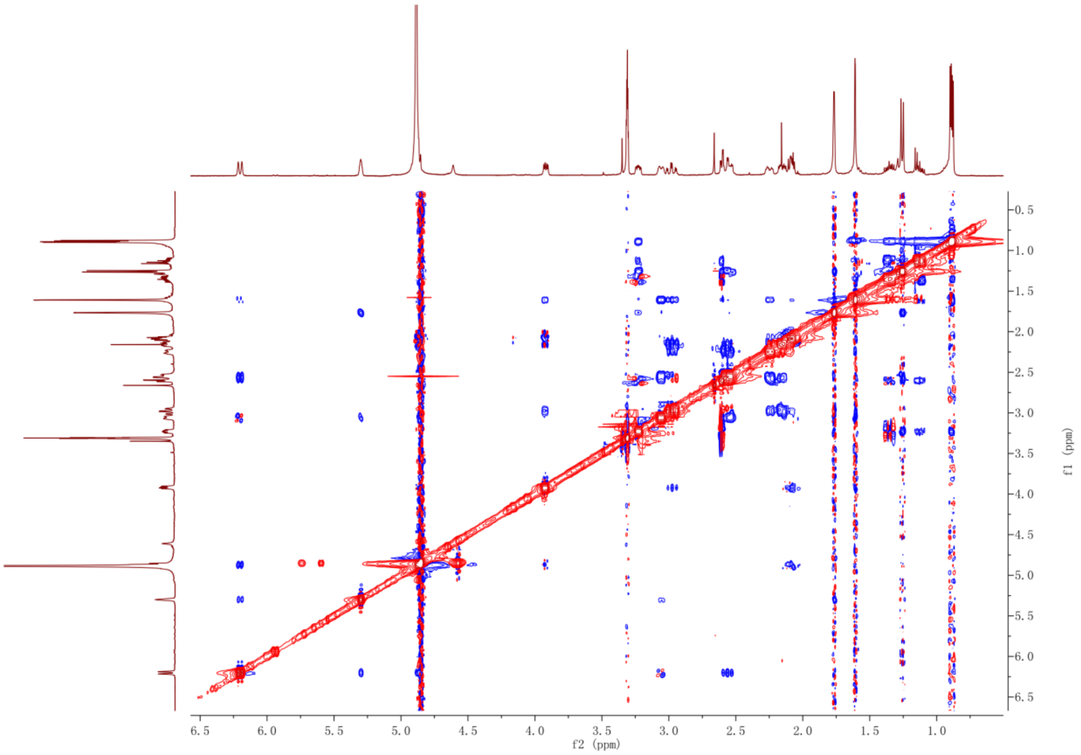

HRESIMS for compound **11**

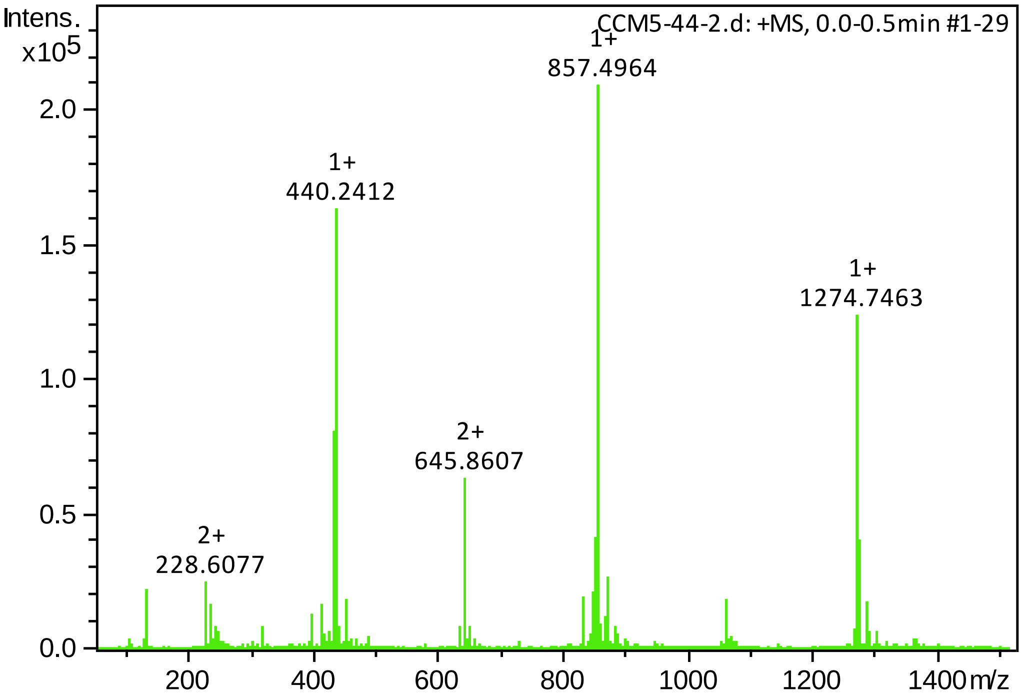

UV spectrum for compound **11**

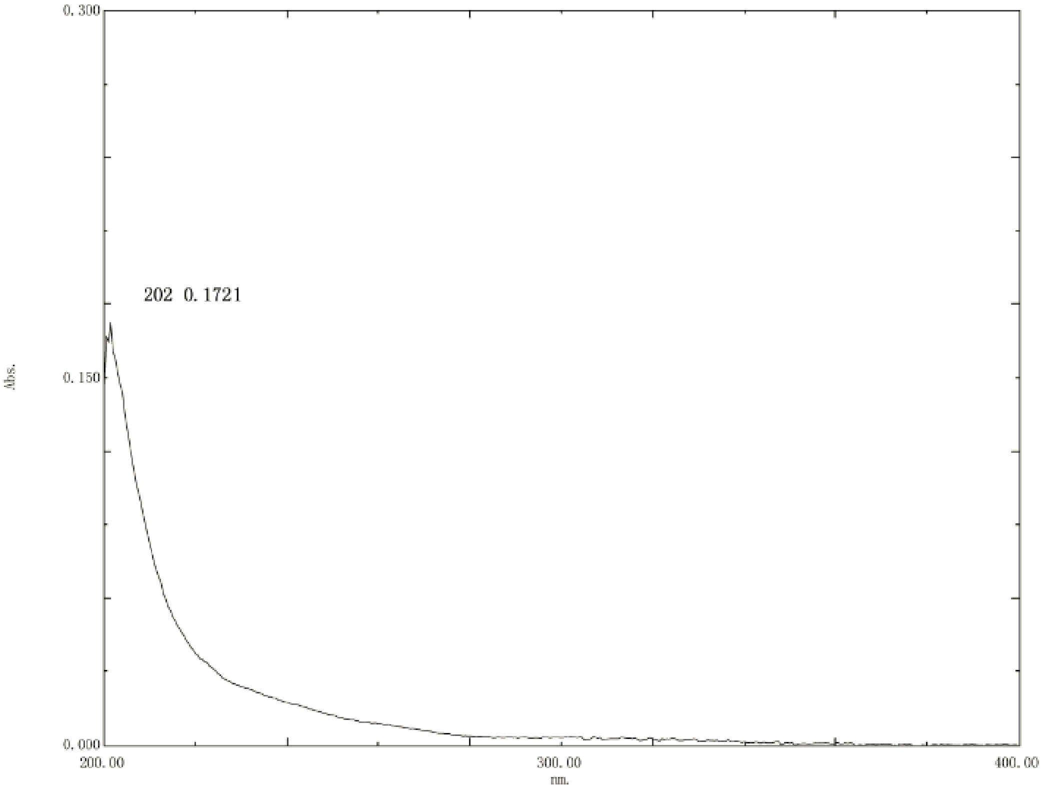

IR spectrum for compound **11**

E:\同济医学院\张勇慧\20150128\AF5-44-2.0

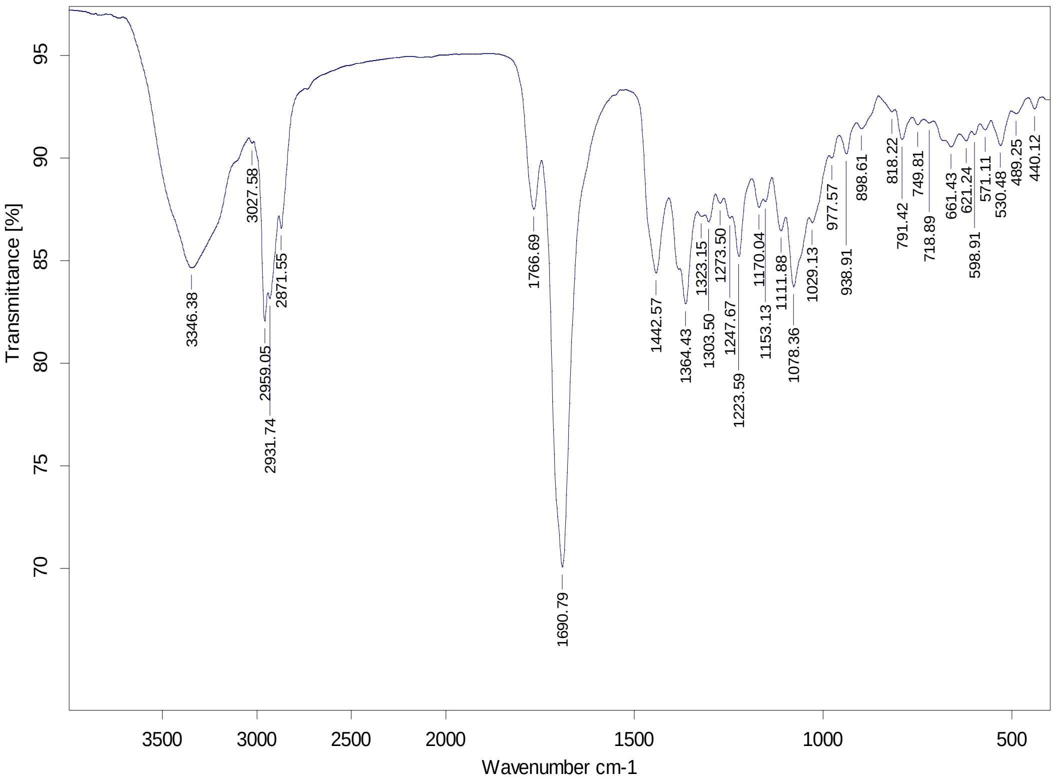

^{1}H NMR for compound **11** (in CD_3OD, 400 MHz)

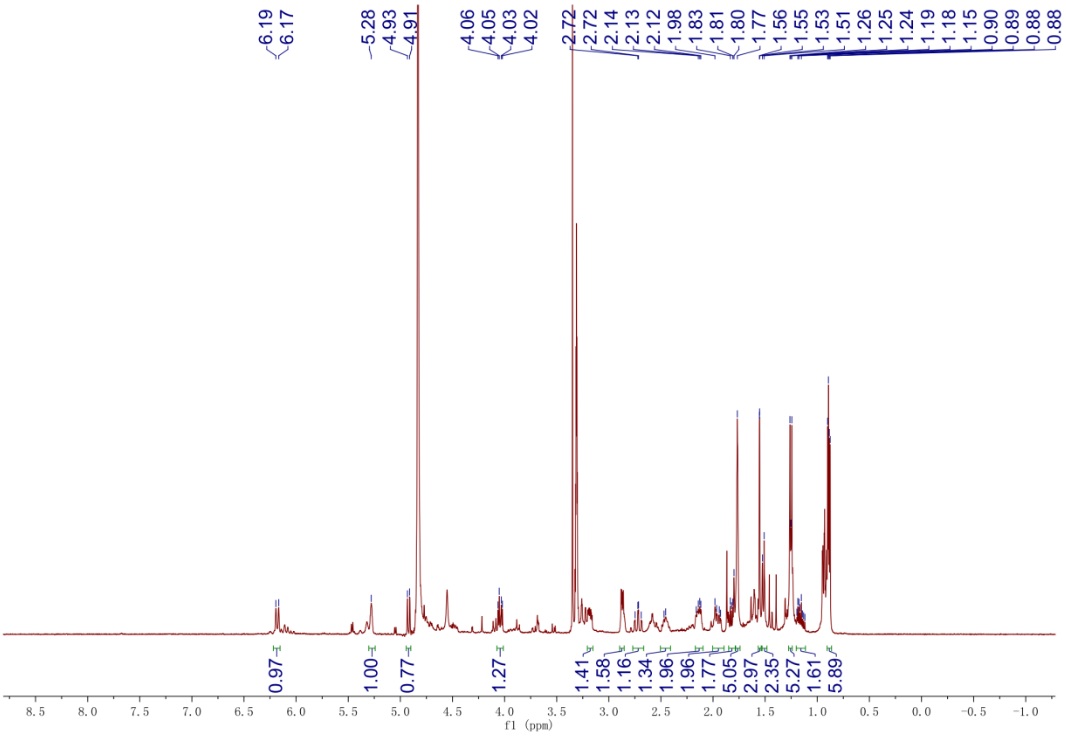

^{13}C NMR for compound **11** (in CD_3OD, 100 MHz)

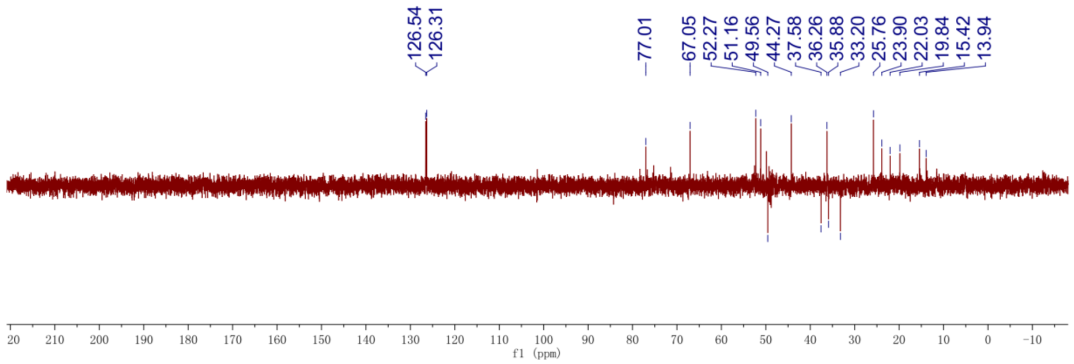

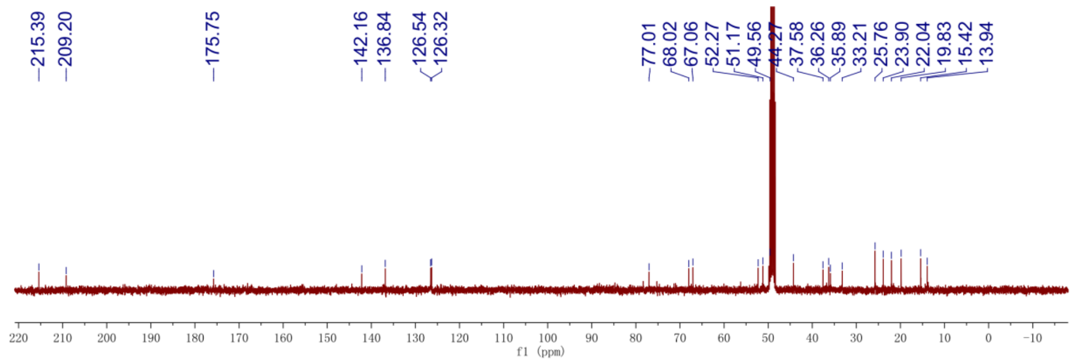

HSQC for compound **11** (in CD_3OD, 400 MHz)

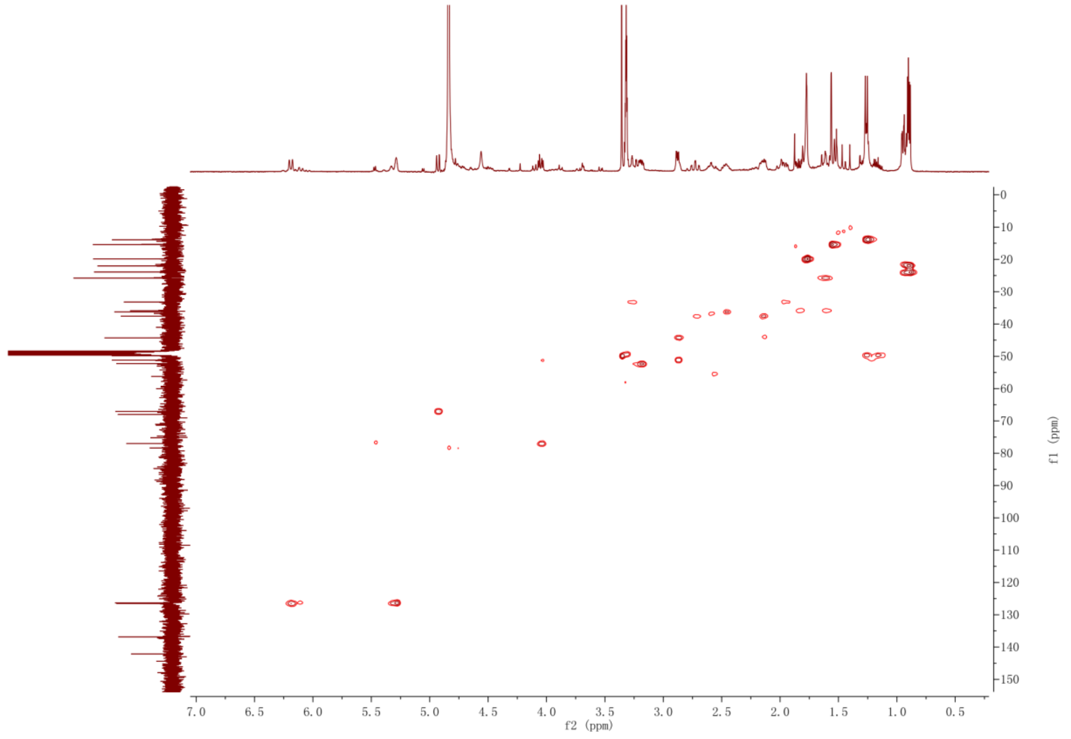

HMBC for compound **11** (in CD_3OD, 400 MHz)

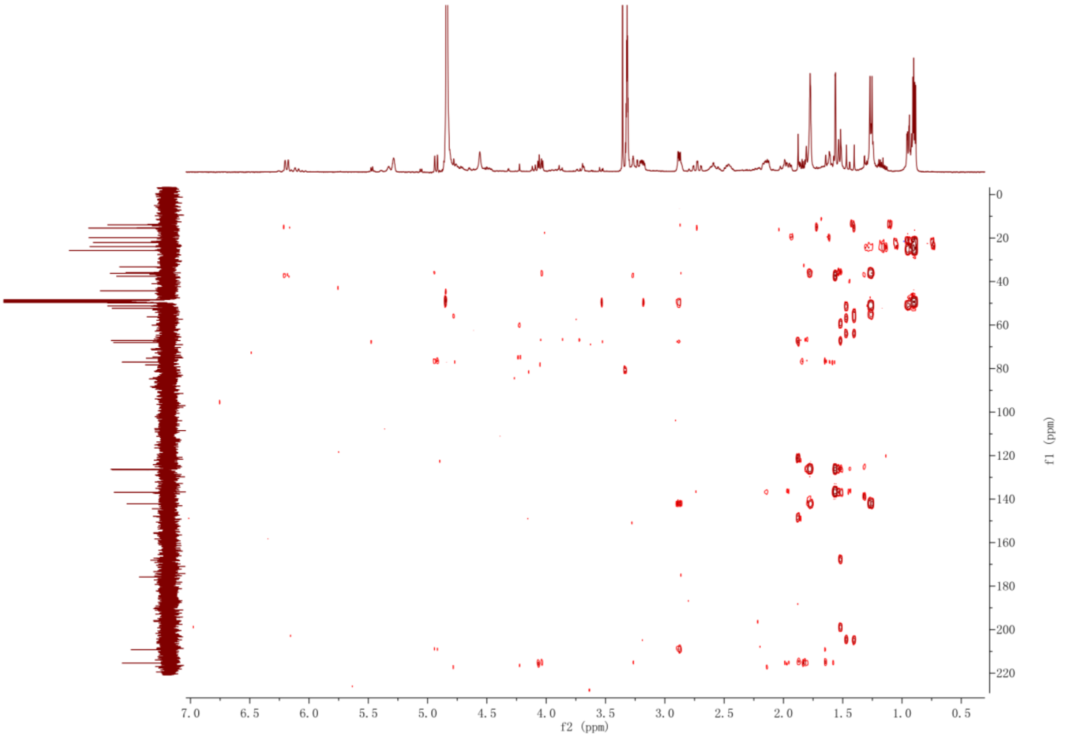

^{1}H–^{1}H COSY for compound **11** (in CD_3OD, 400 MHz)

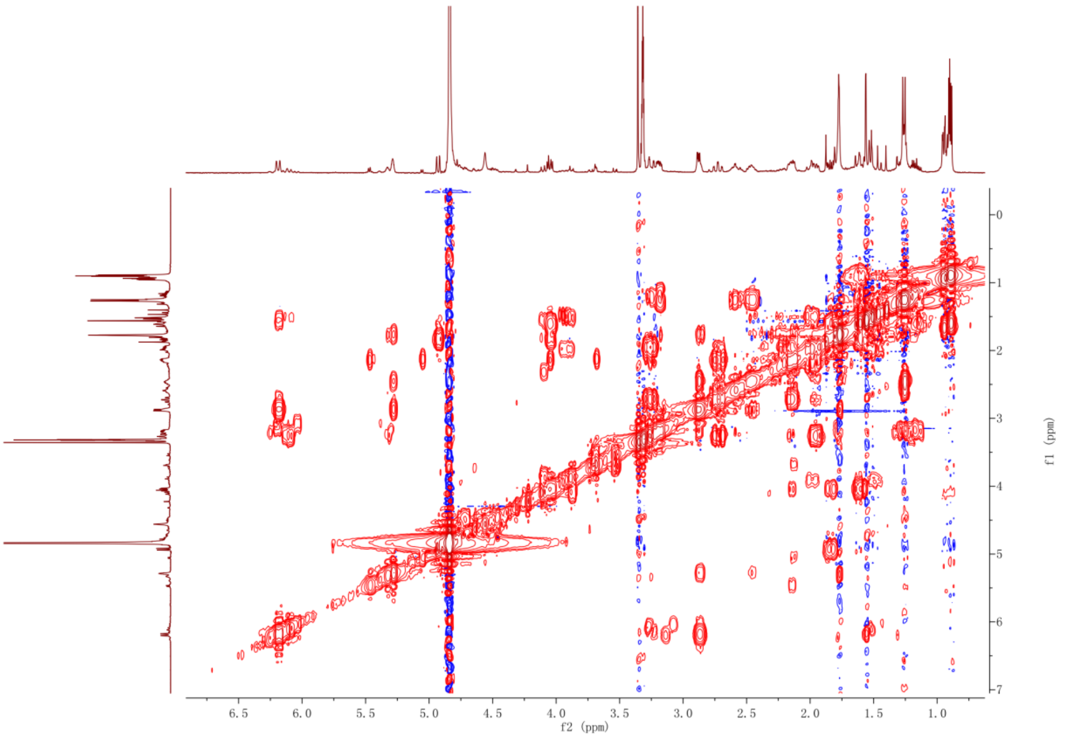

NOESY for compound **11** (in CD_3OD, 400 MHz)

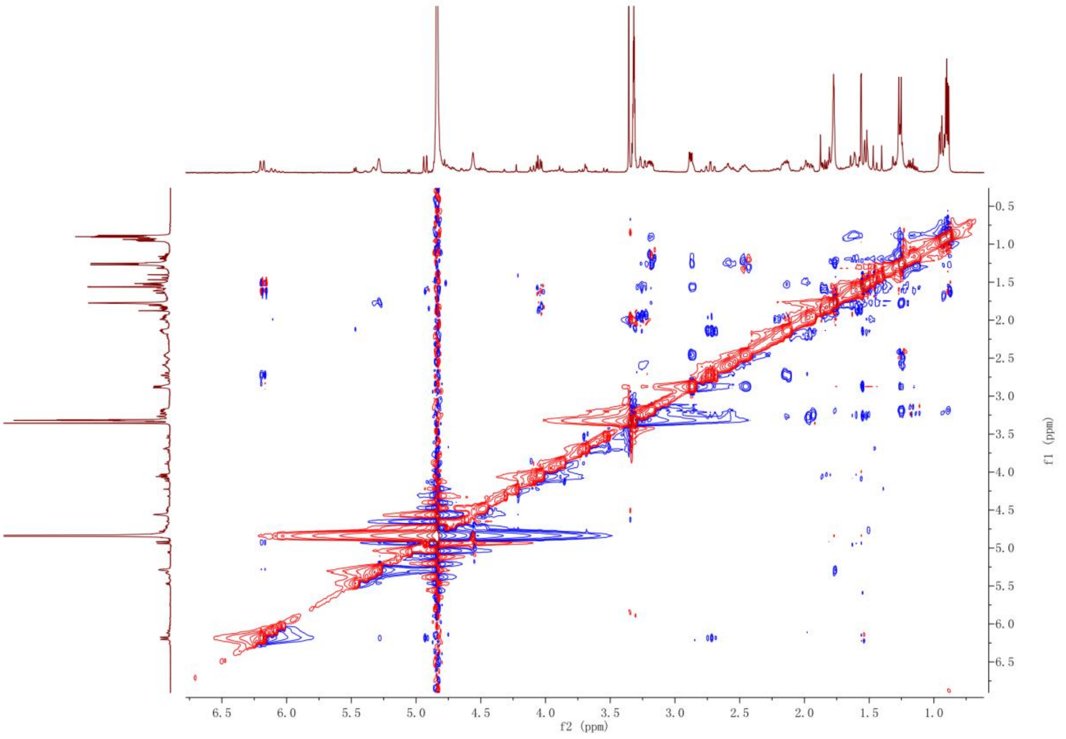

HRESIMS for compound **12**

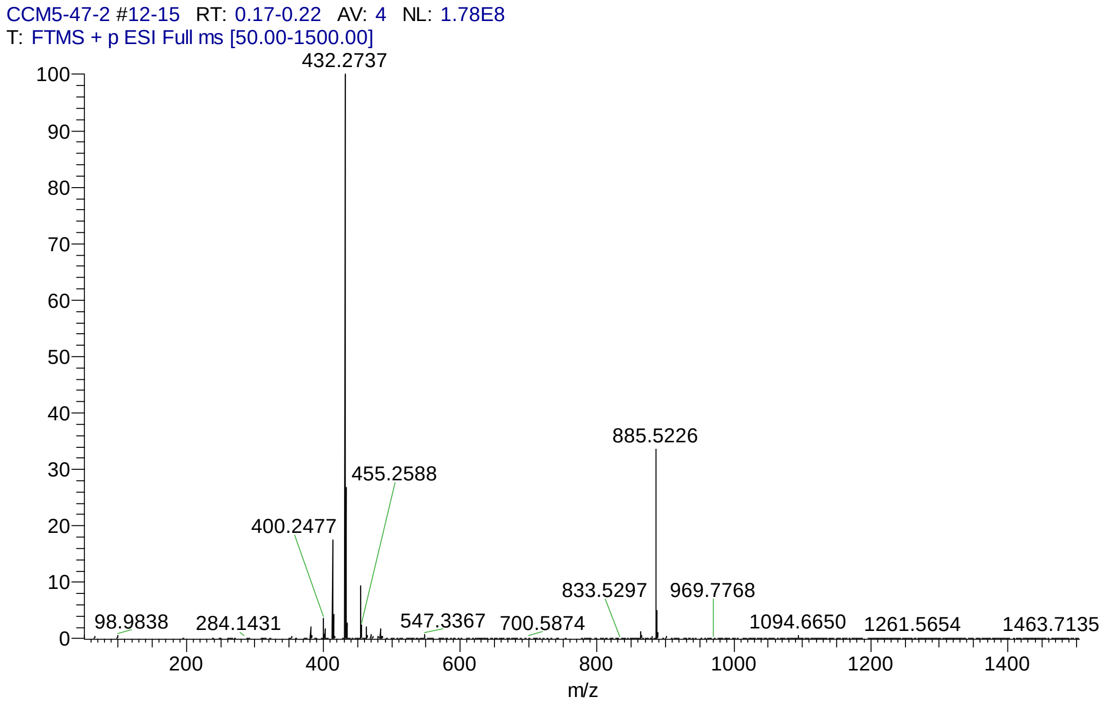

UV spectrum for compound **12**

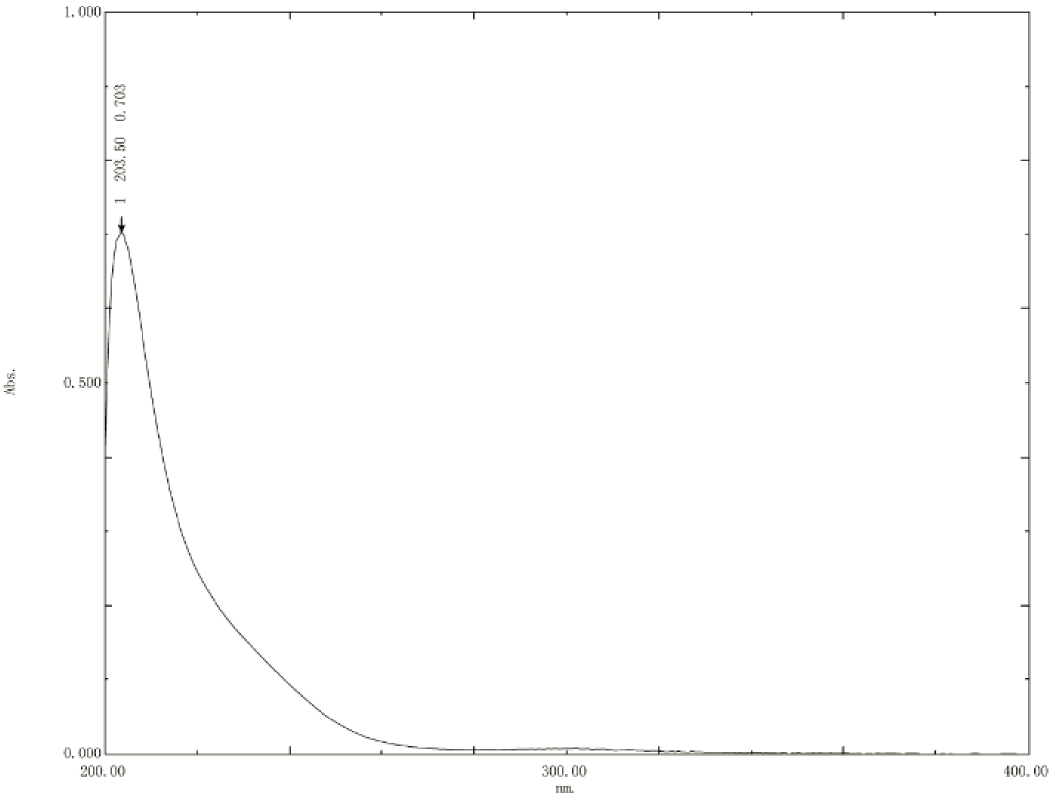

IR spectrum for compound **12**

E:\同济医学院\张勇慧\20150128\AF5-47-2.0

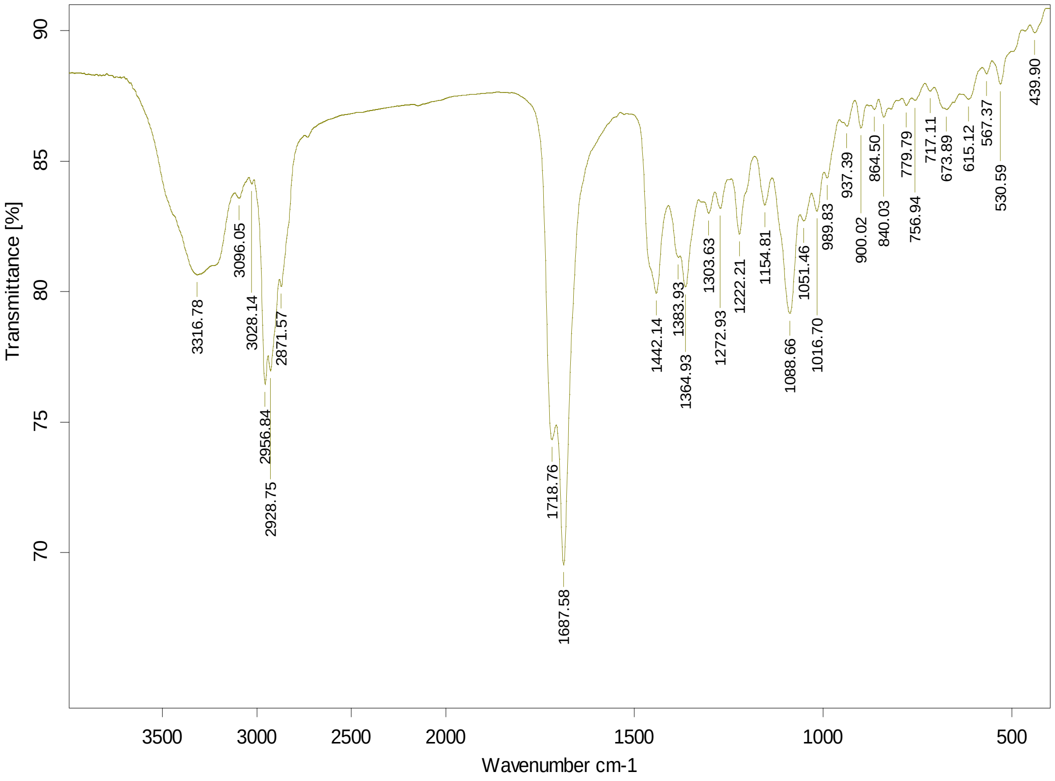

^{1}H NMR for compound **12** (in CD_3OD, 400 MHz)

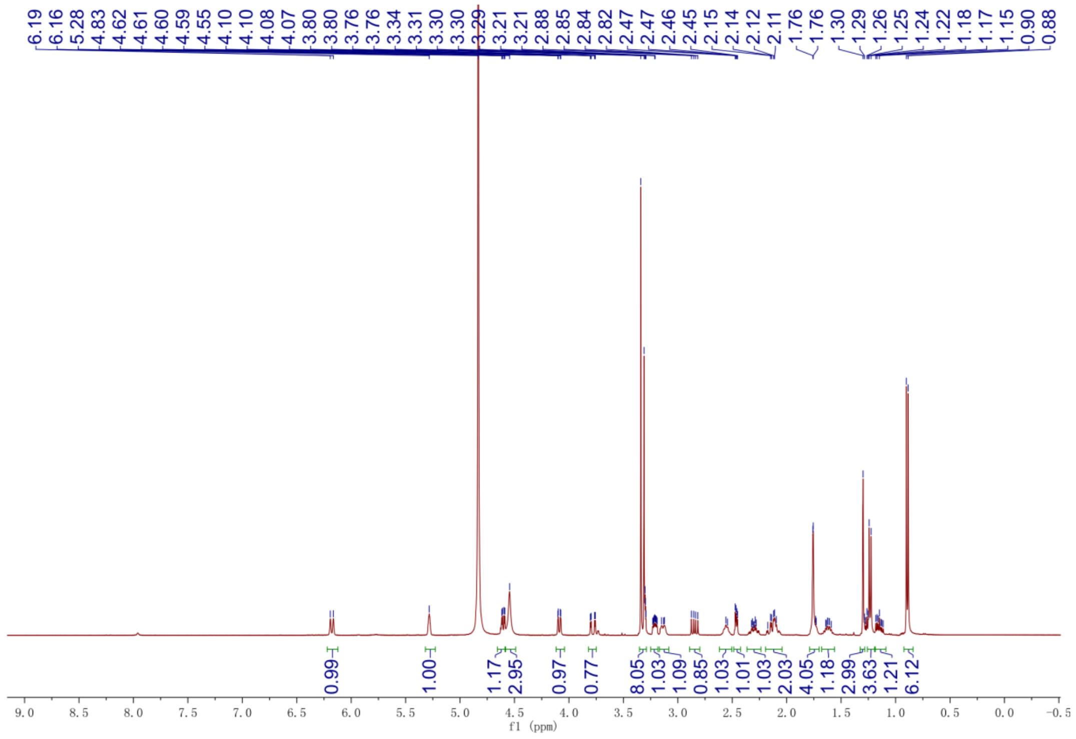

^{13}C NMR for compound **12** (in CD_3OD, 100 MHz)

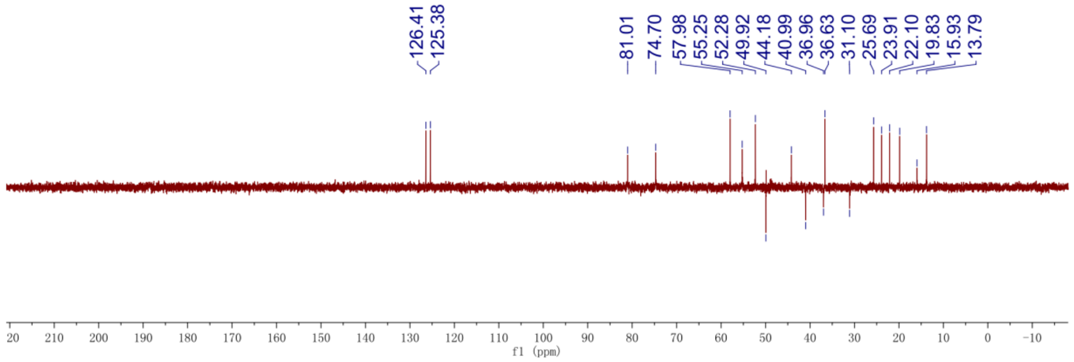

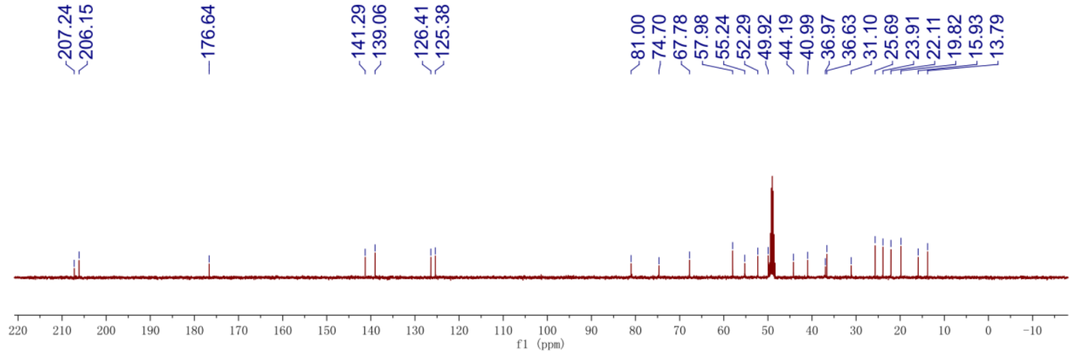

HSQC for compound **12** (in CD_3OD, 400 MHz)

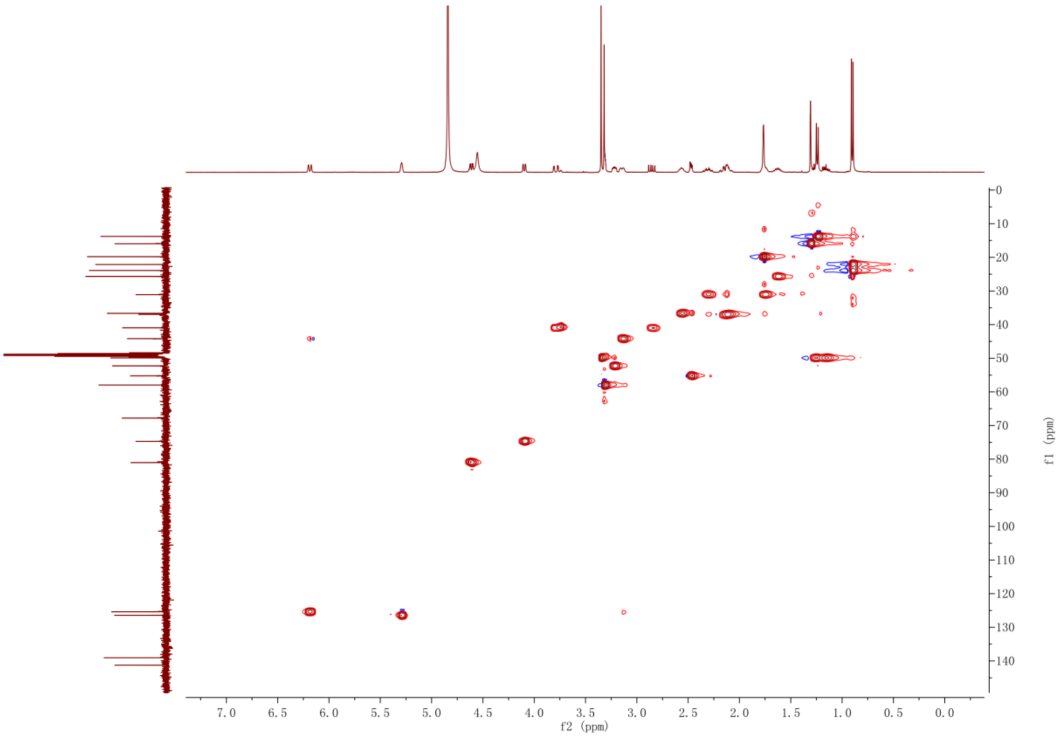

HMBC for compound **12** (in CD_3OD, 400 MHz)

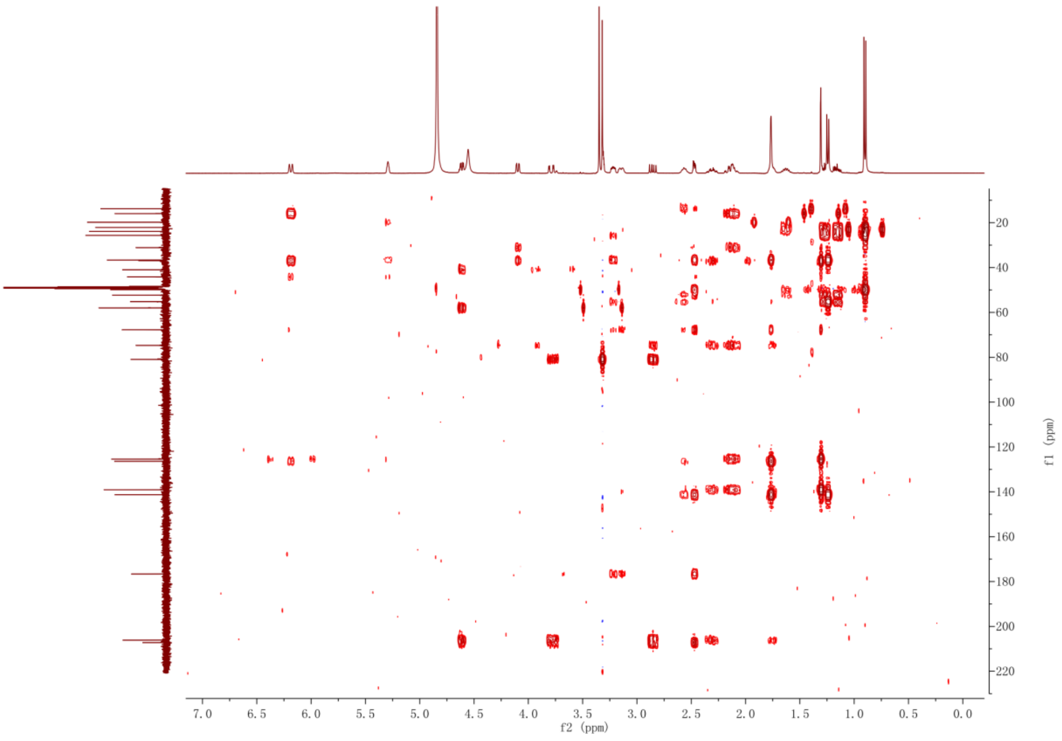

^{1}H–^{1}H COSY for compound **12** (in CD_3OD, 400 MHz)

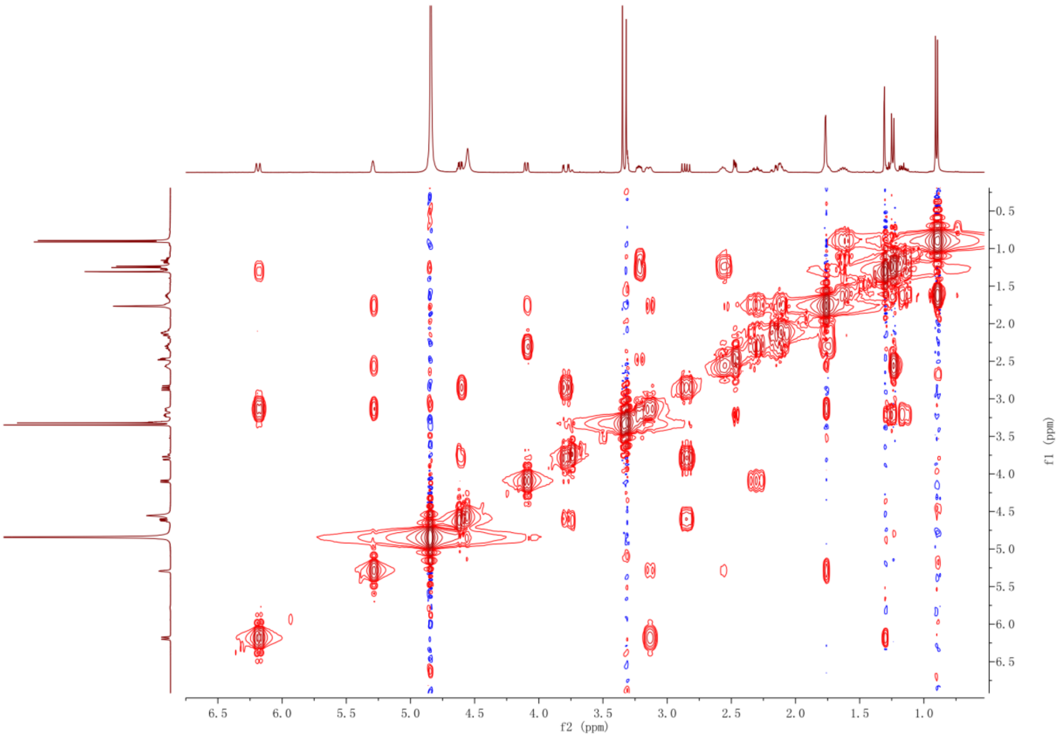

NOESY for compound **12** (in CD_3OD, 400 MHz)

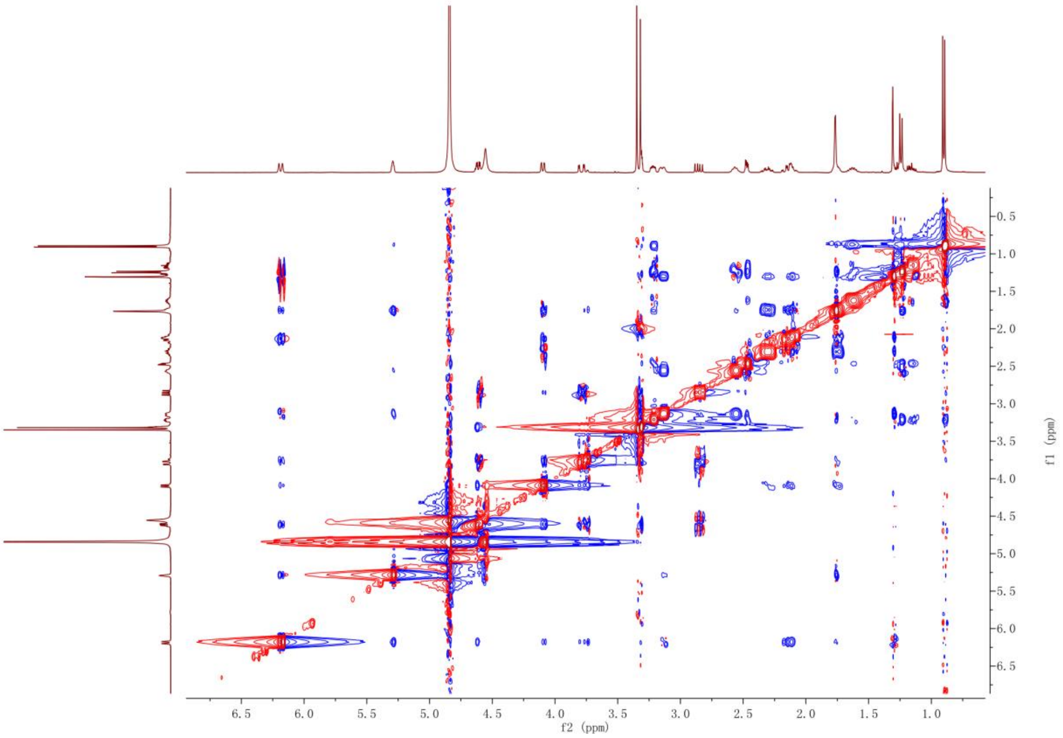

HRESIMS for compound **13**

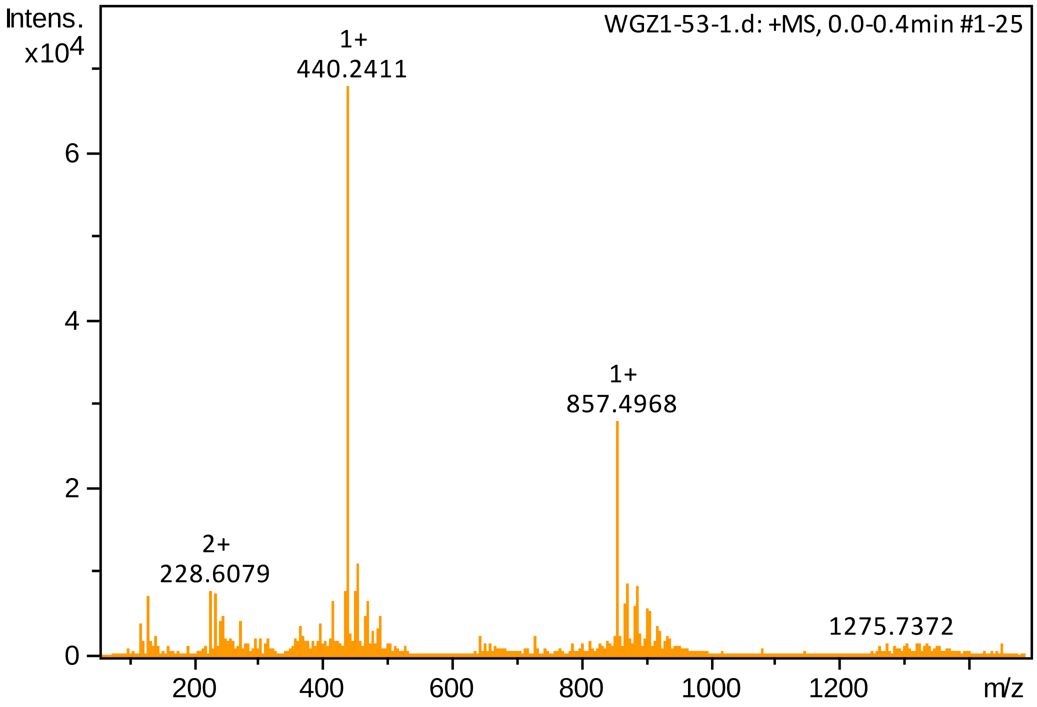

UV spectrum for compound **13**

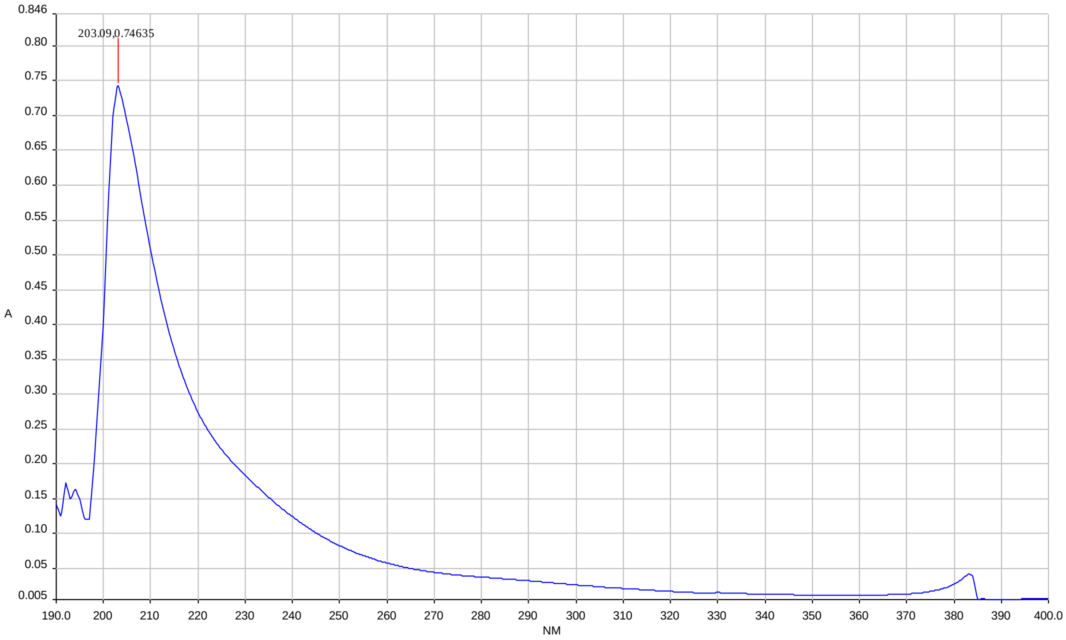

IR spectrum for compound **13**

E:\20160802\魏广正\1-53-1.0

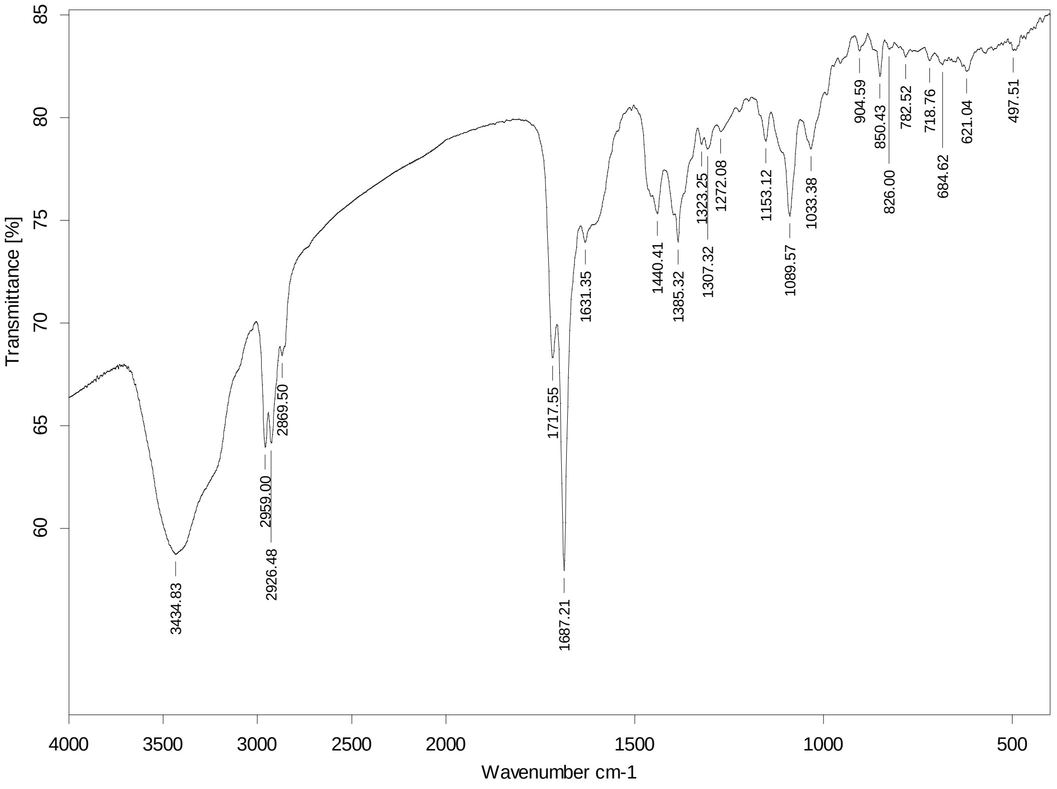

^{1}H NMR for compound **13** (in CD_3OD, 400 MHz)

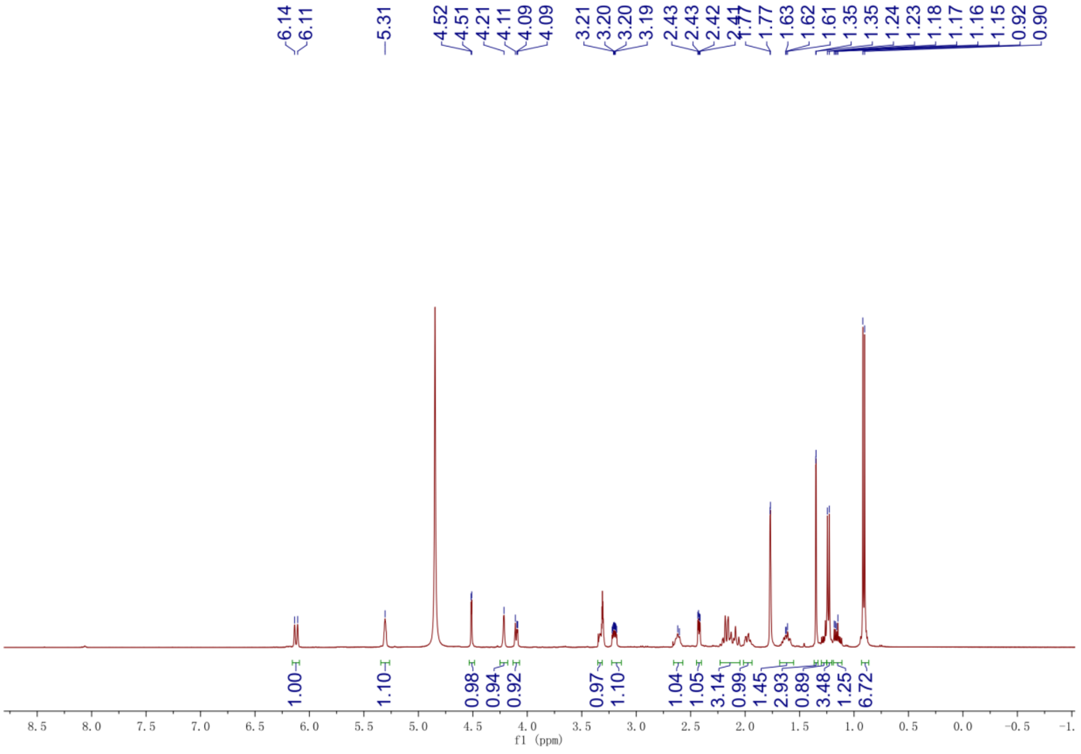

^{13}C NMR for compound **13** (in CD_3OD, 100 MHz)

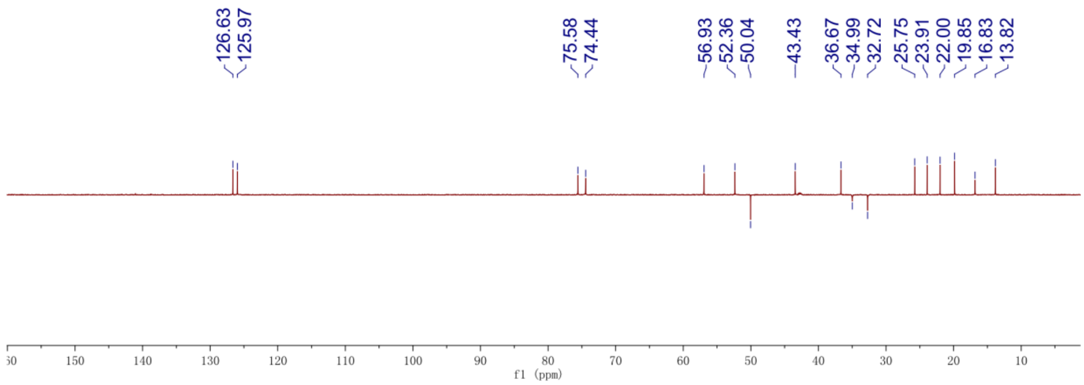

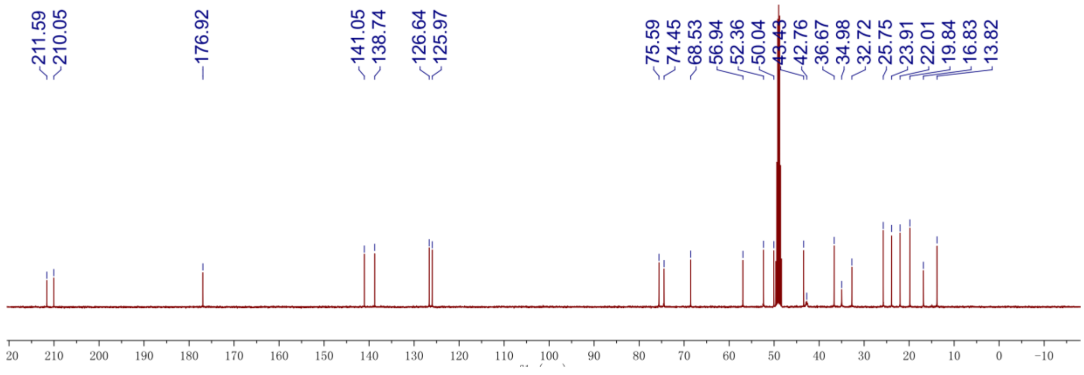

HSQC for compound **13** (in CD_3OD, 400 MHz)

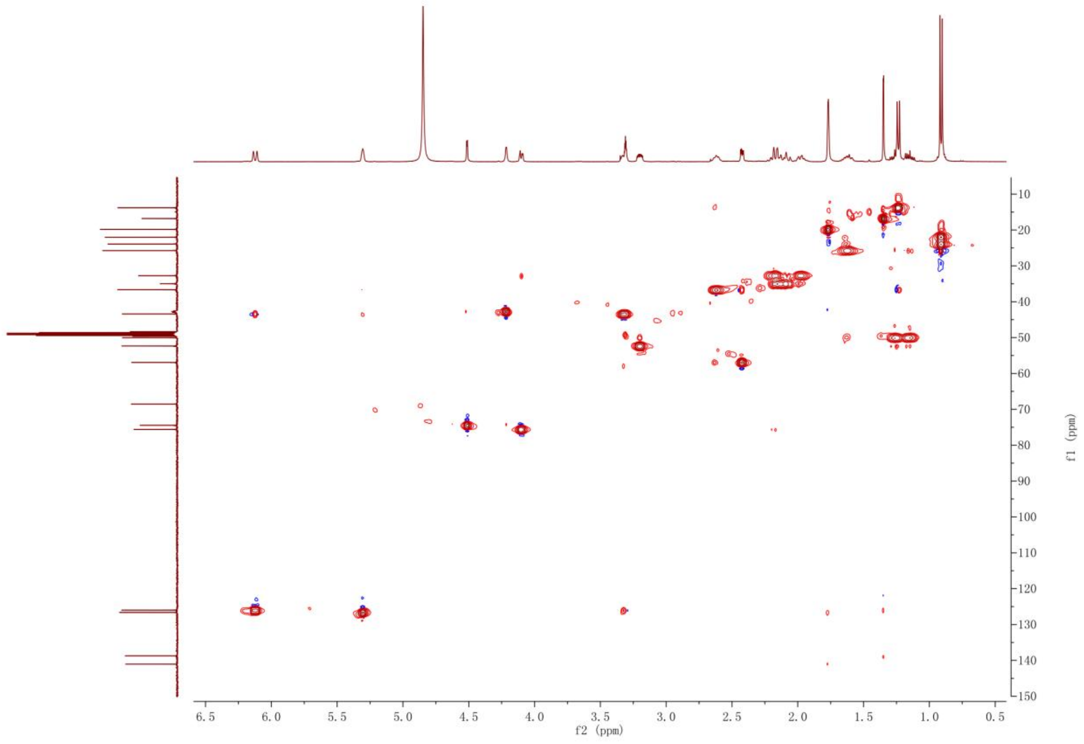

HMBC for compound **13** (in CD_3OD, 400 MHz)

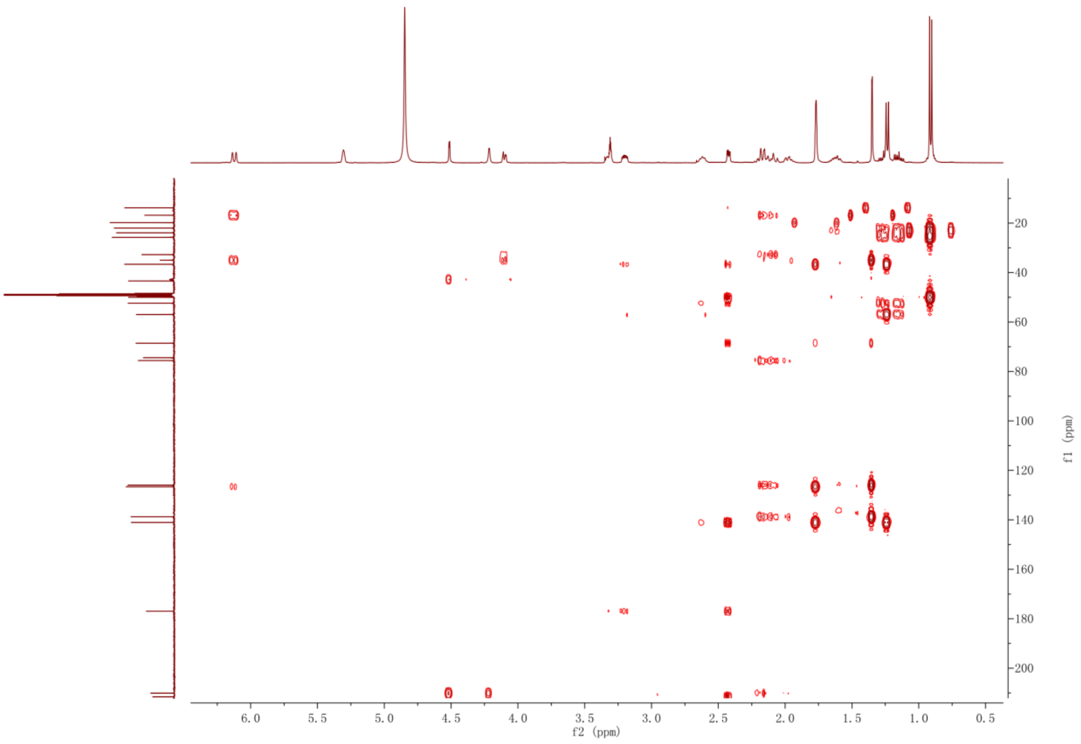

^1H–^1H COSY for compound **13** (in CD_3OD, 400 MHz)

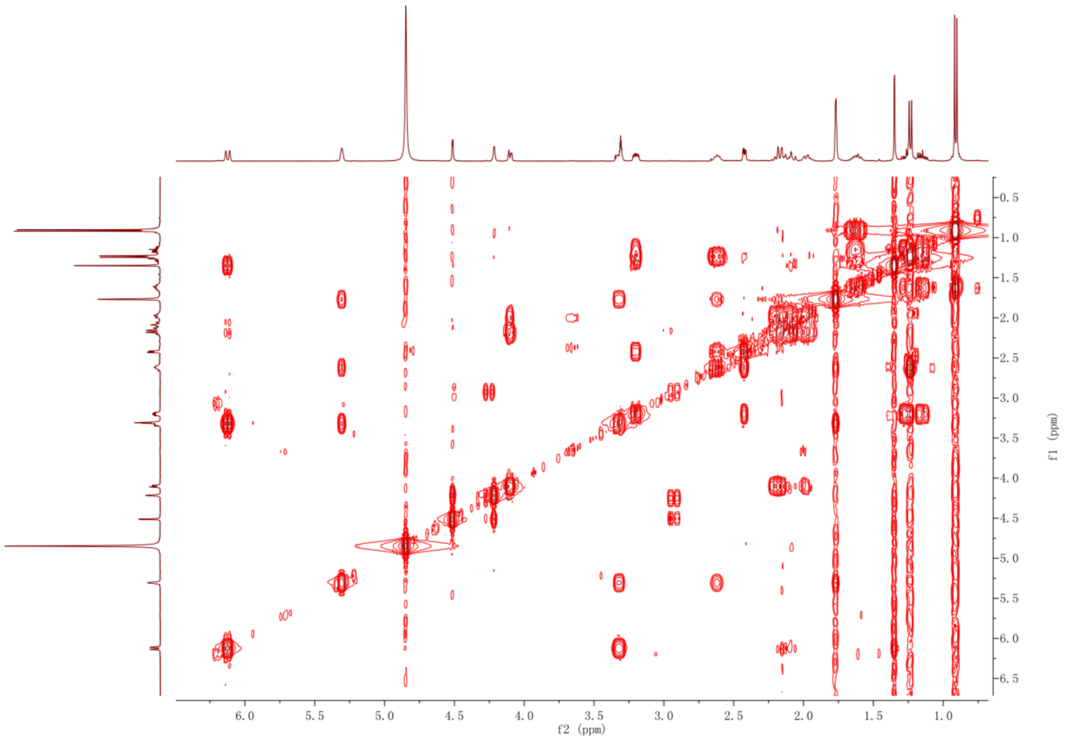

NOESY for compound **13** (in CD_3OD, 400 MHz)

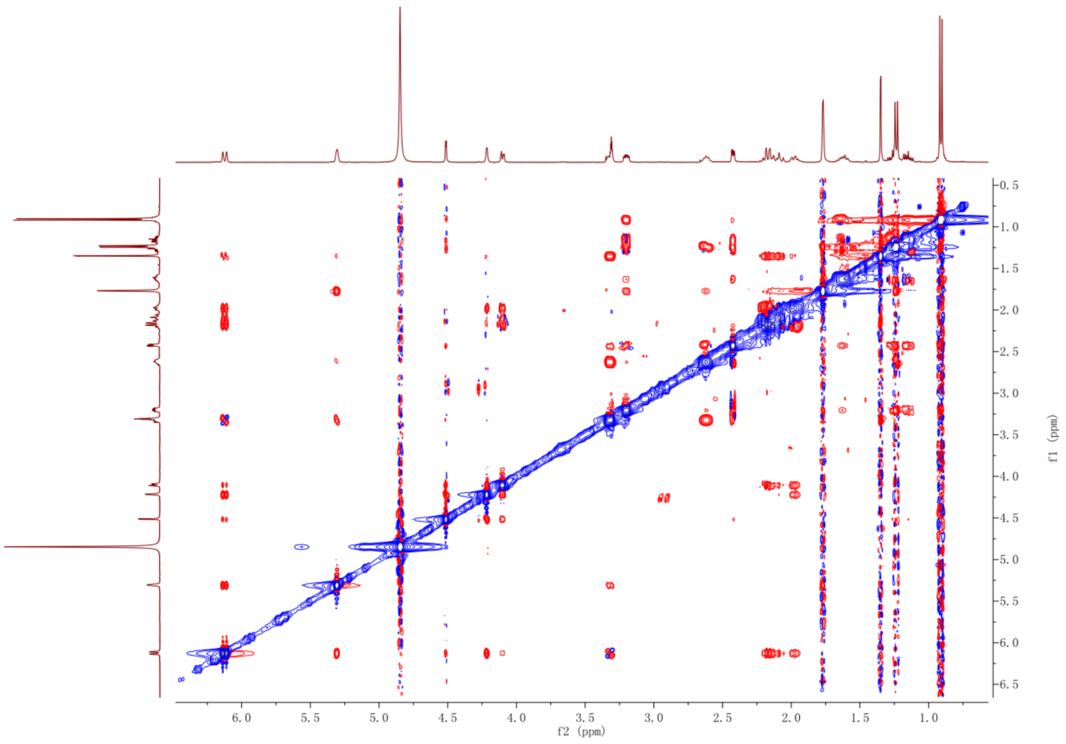

^{1}H NMR for compound **13** (in DMSO, 400 MHz)

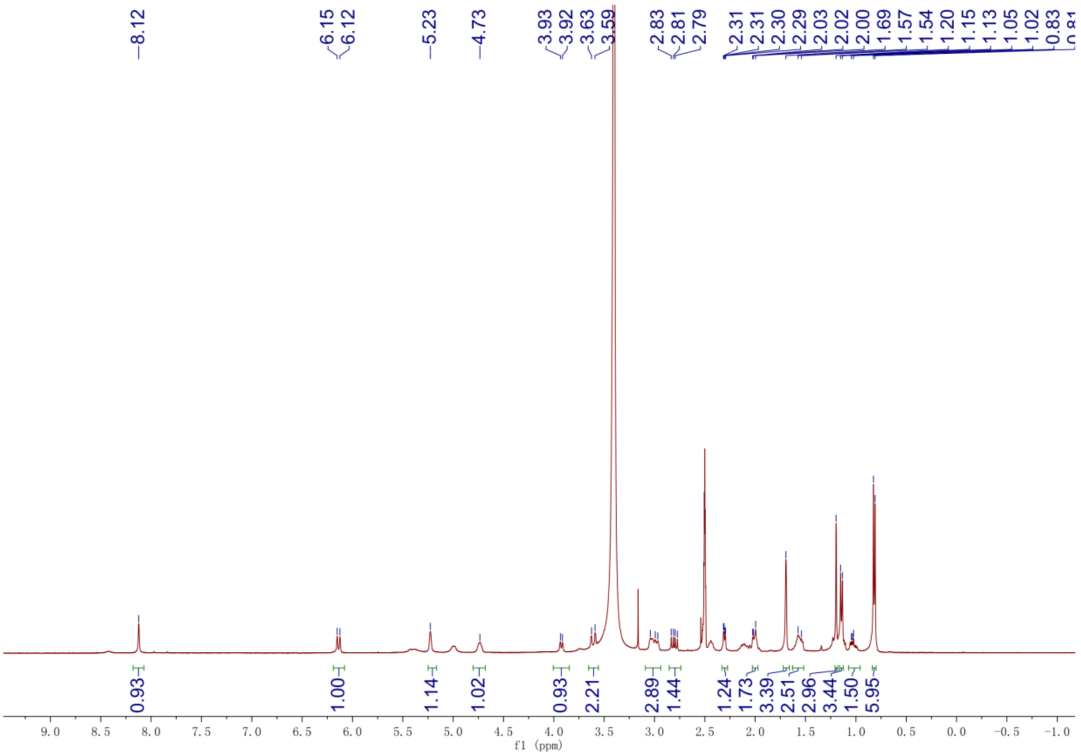

^{13}C NMR for compound **13** (in DMSO, 100 MHz)

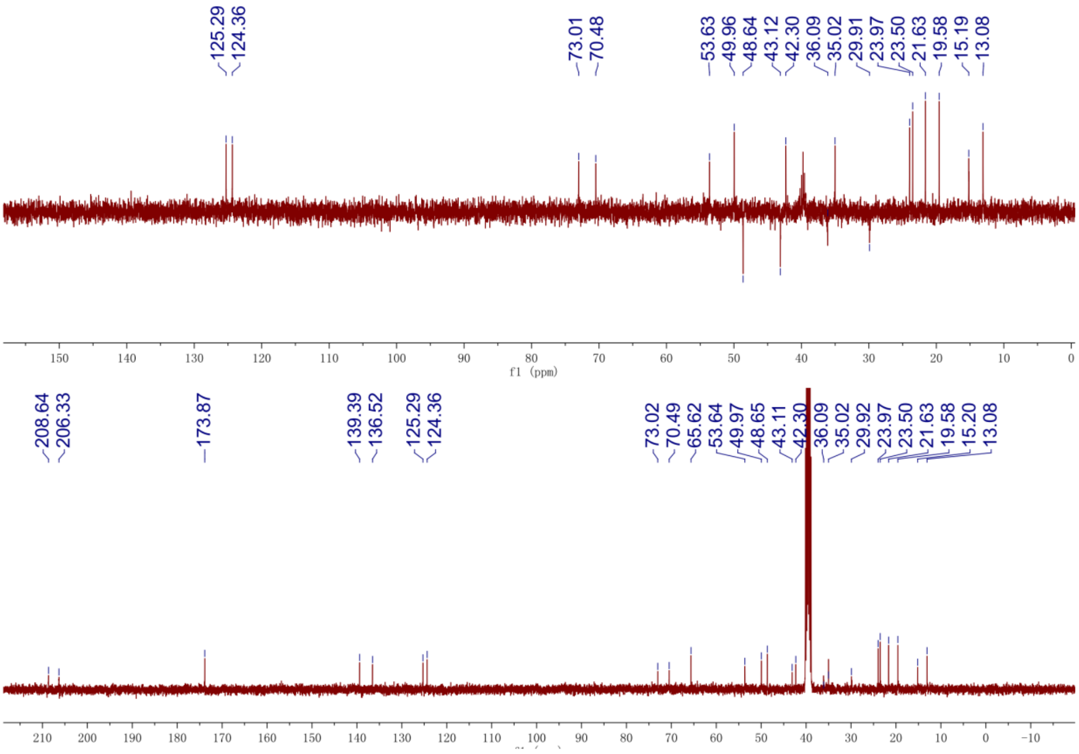

HSQC for compound **13** (in DMSO, 400 MHz)

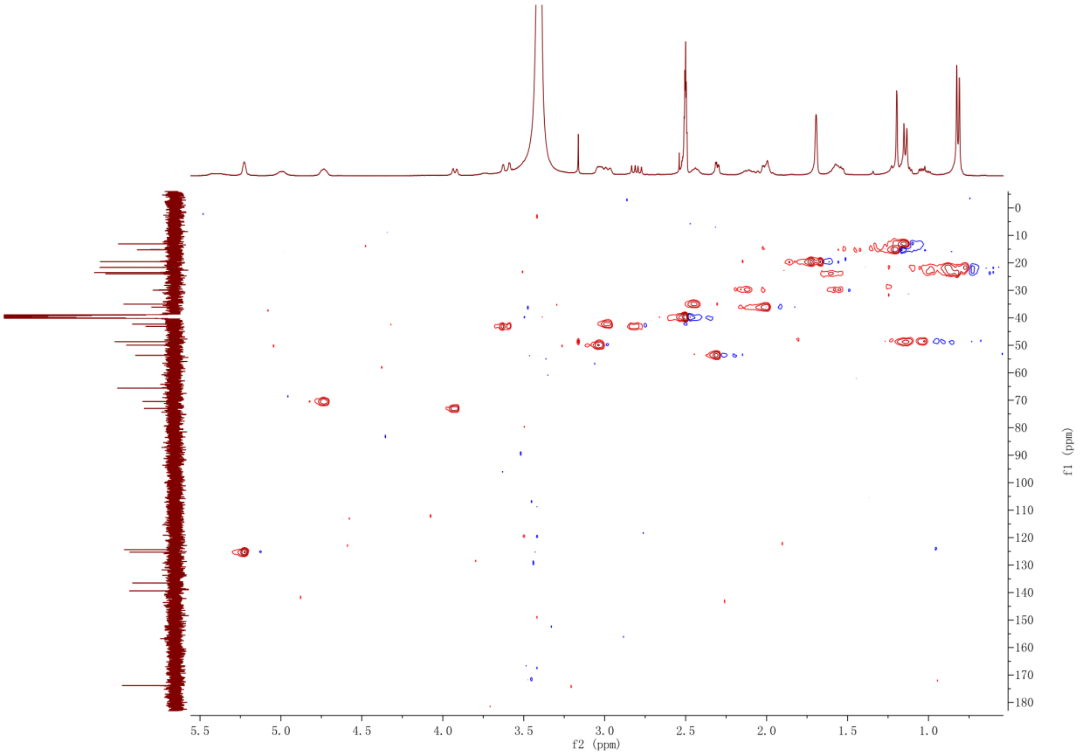

HMBC for compound **13** (in DMSO, 400 MHz)

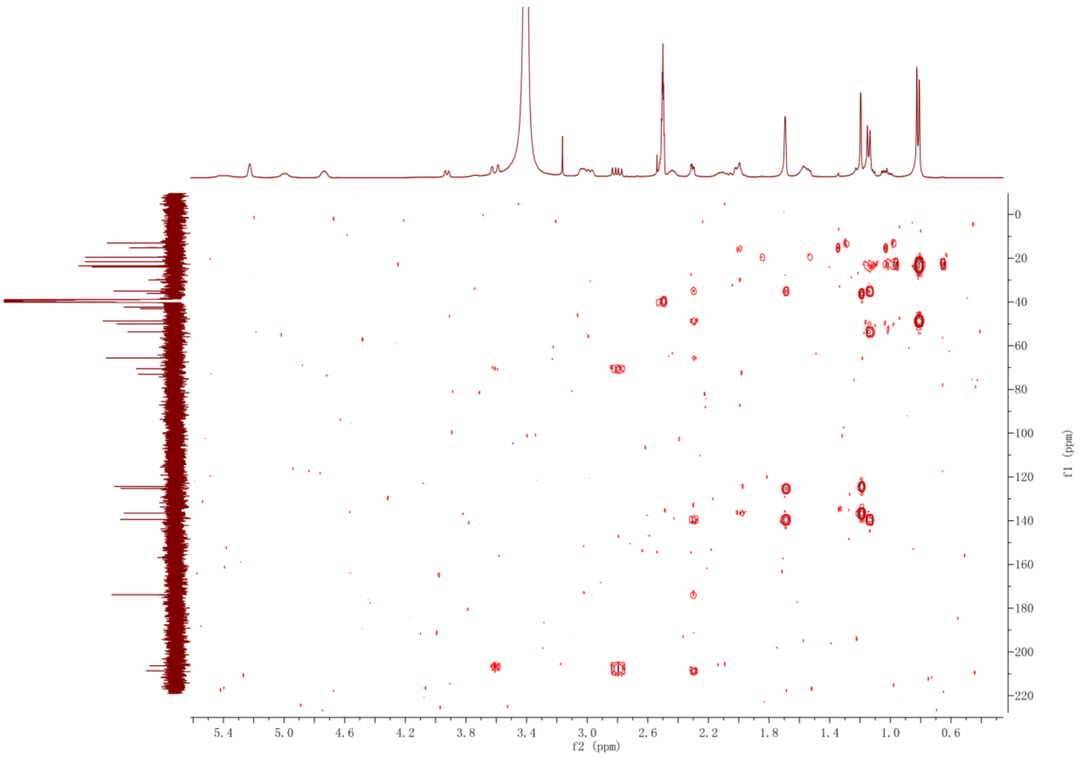

^{1}H–^{1}H COSY for compound **13** (in DMSO, 400 MHz)

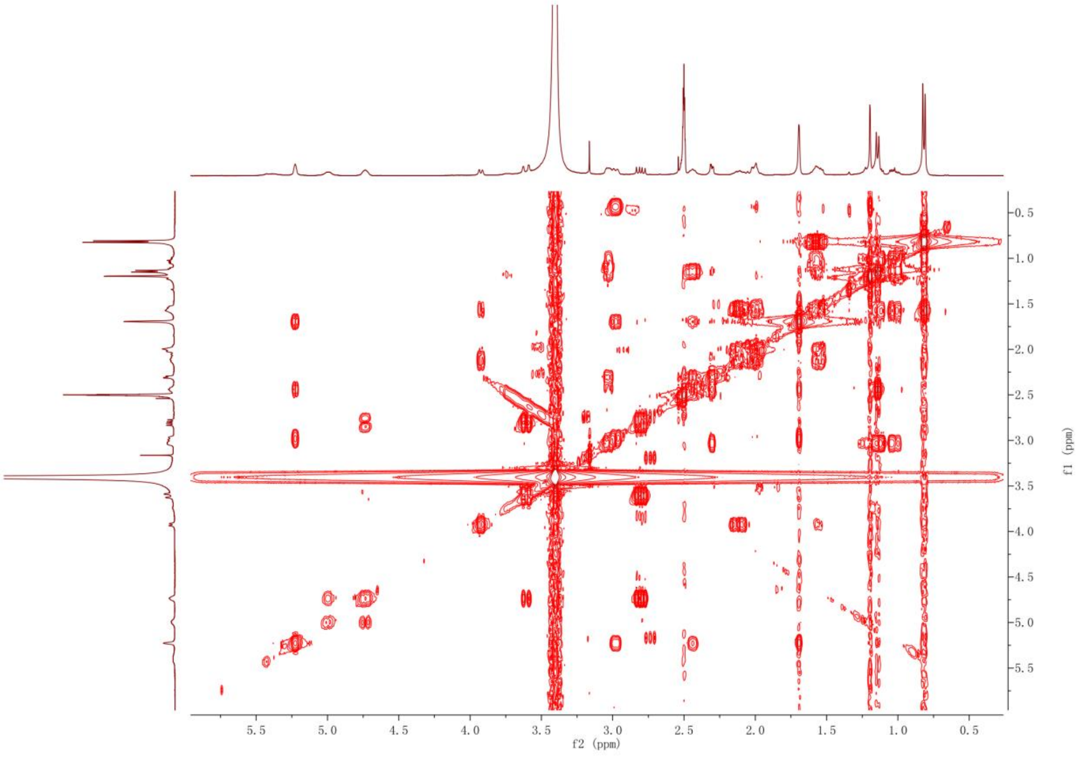

NOESY for compound **13** (in DMSO, 400 MHz)

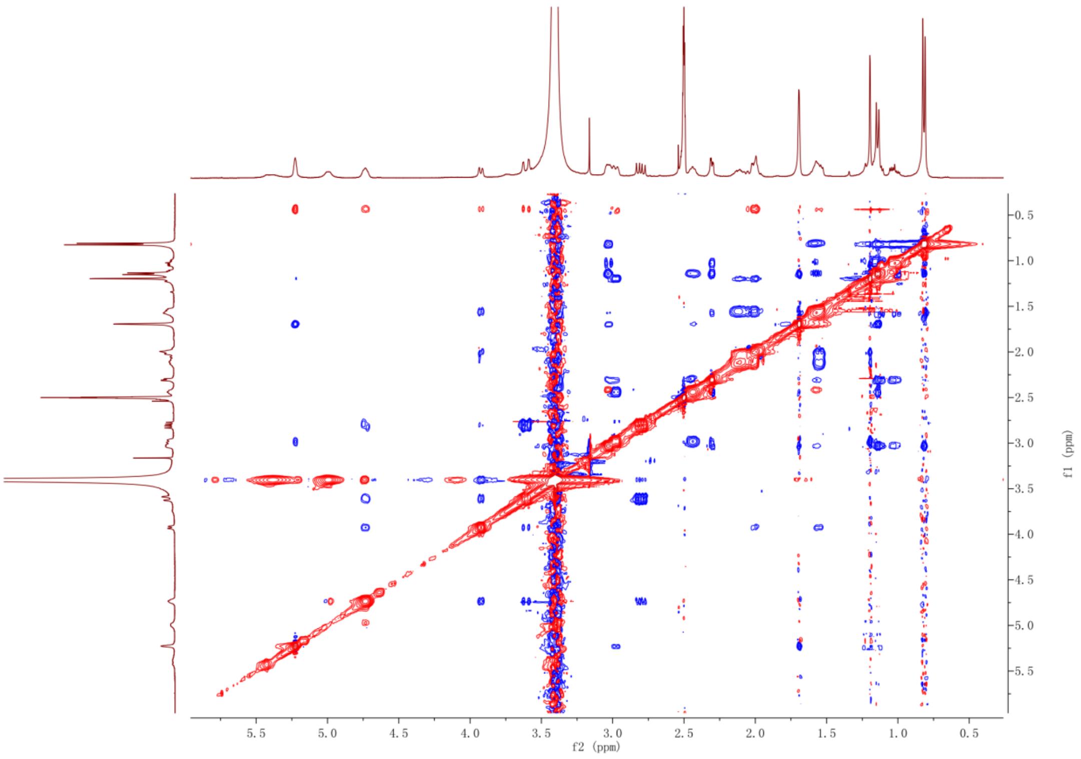

^{1}H NMR for compound **14** (in CD_3OD, 400 MHz)

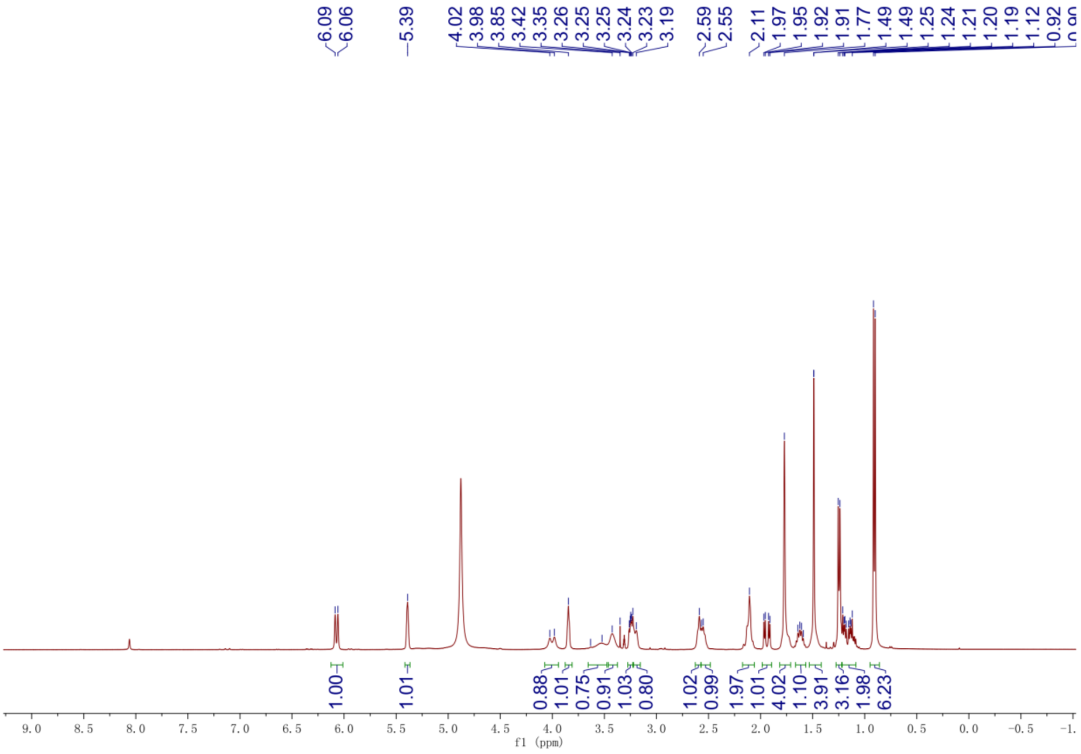

^{13}C NMR for compound **14** (in CD_3OD, 100 MHz)

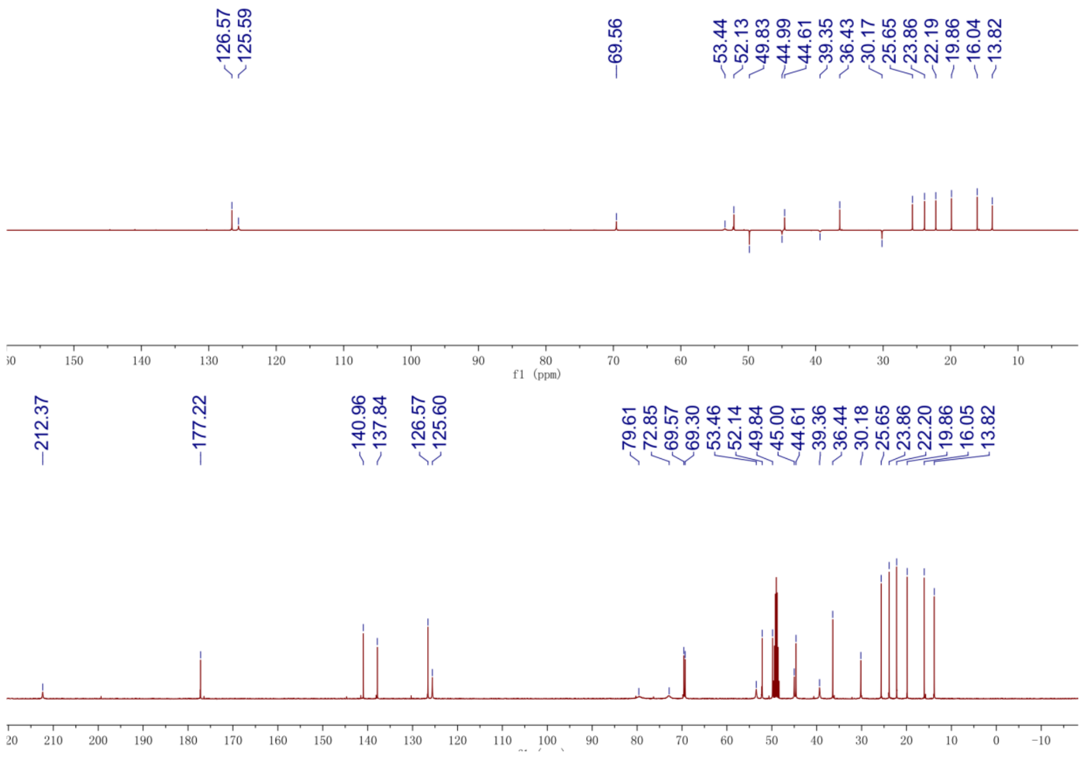

HSQC for compound **14** (in CD_3OD, 400 MHz)

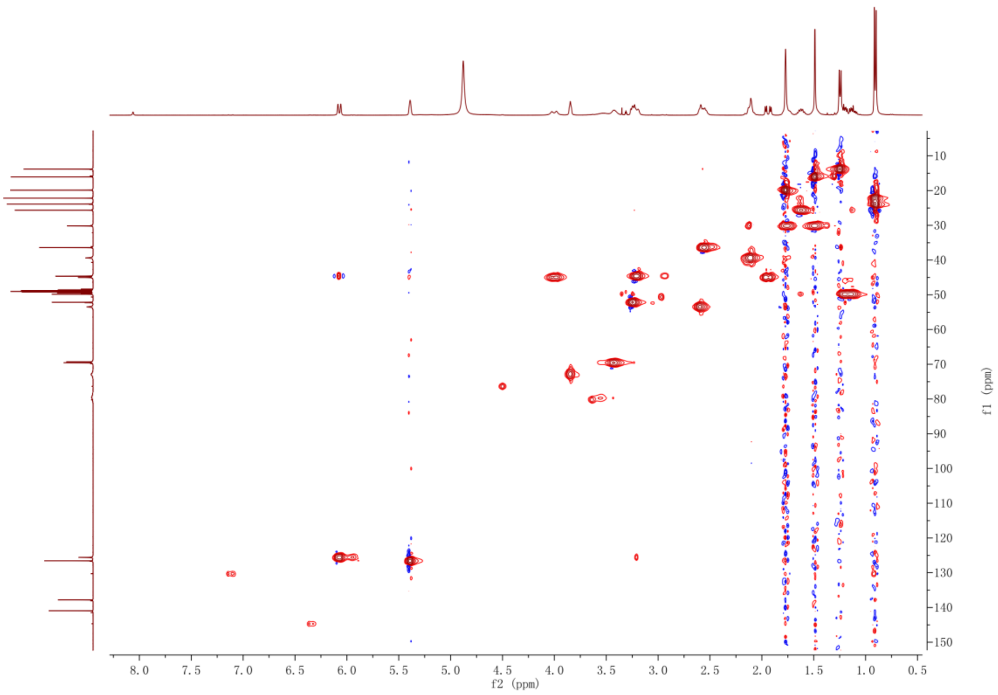

HMBC for compound **14** (in CD_3OD, 400 MHz)

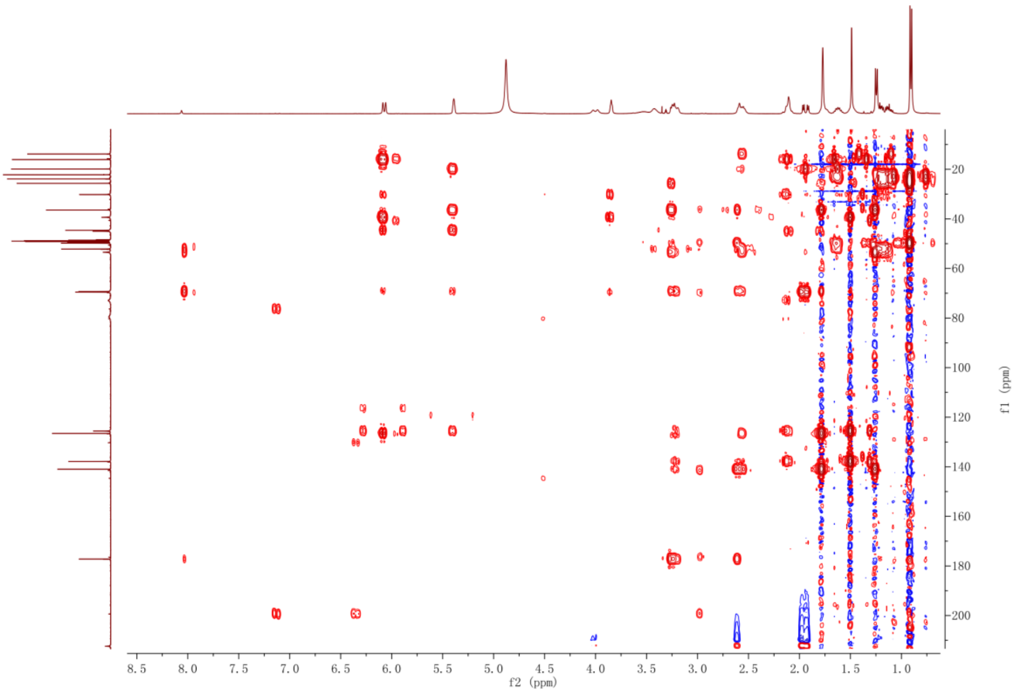

^{1}H–^{1}H COSY for compound **14** (in CD_3OD, 400 MHz)

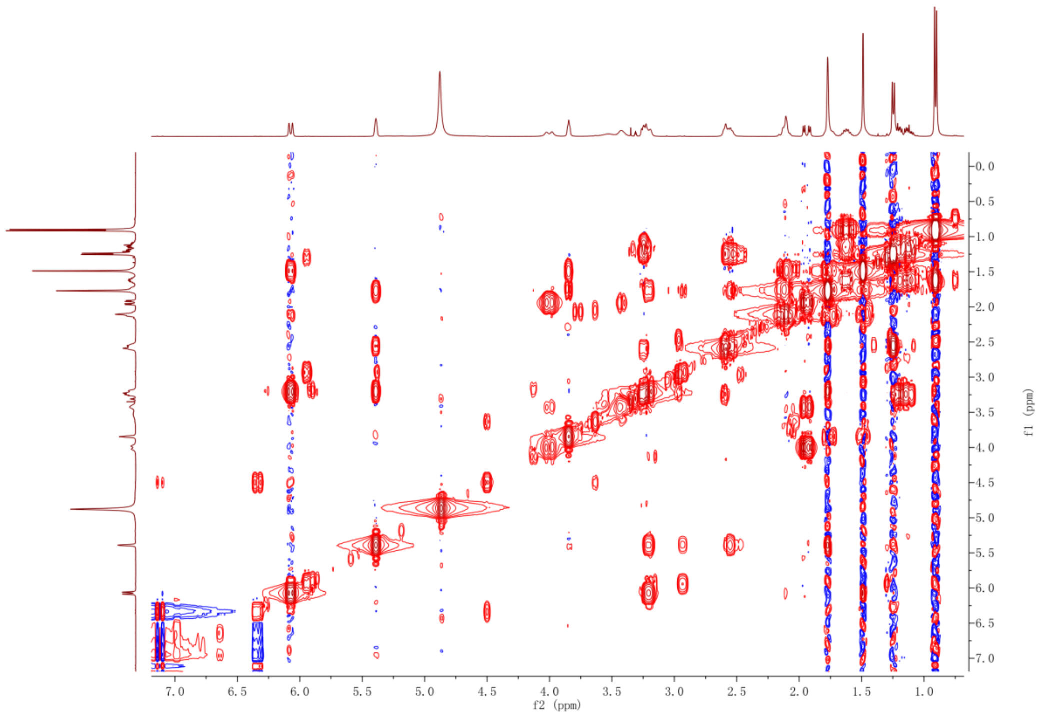

NOESY for compound **14** (in CD_3OD, 400 MHz)

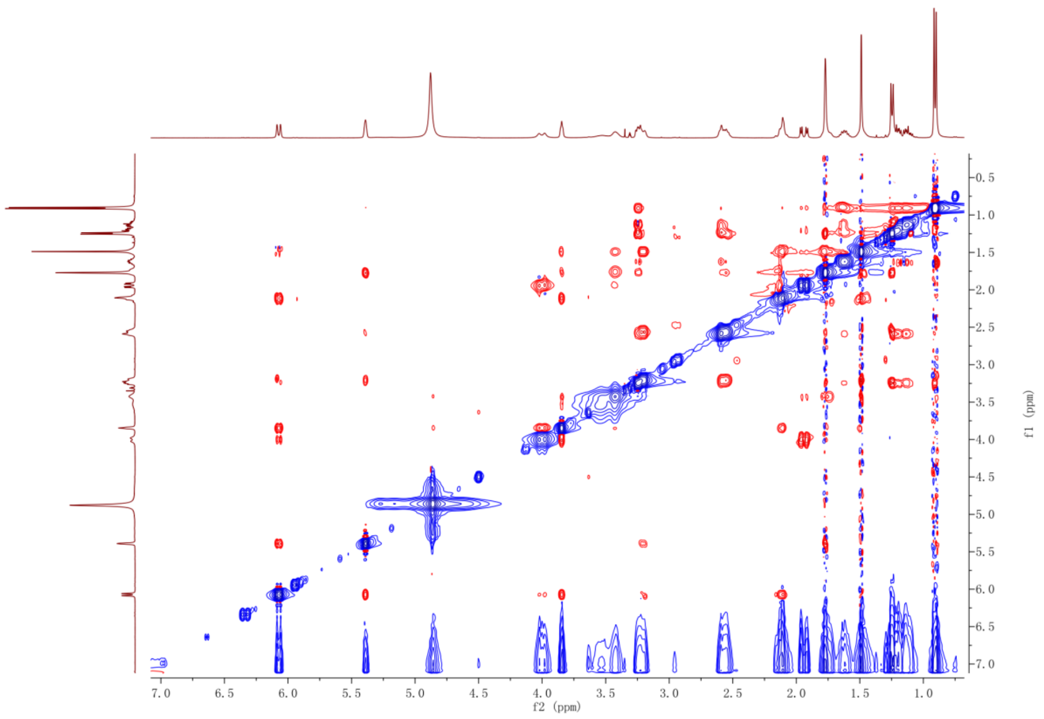

^{1}H NMR for compound **15** (in DMSO, 400 MHz)

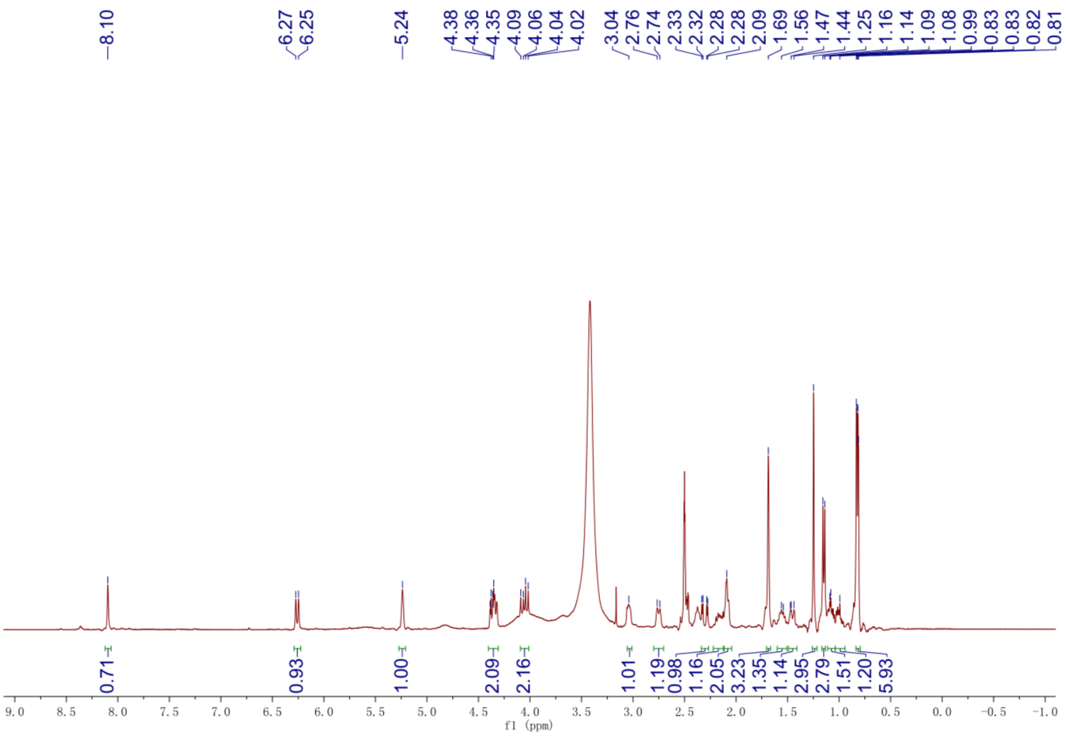

^{13}C NMR for compound **15** (in DMSO, 100 MHz)

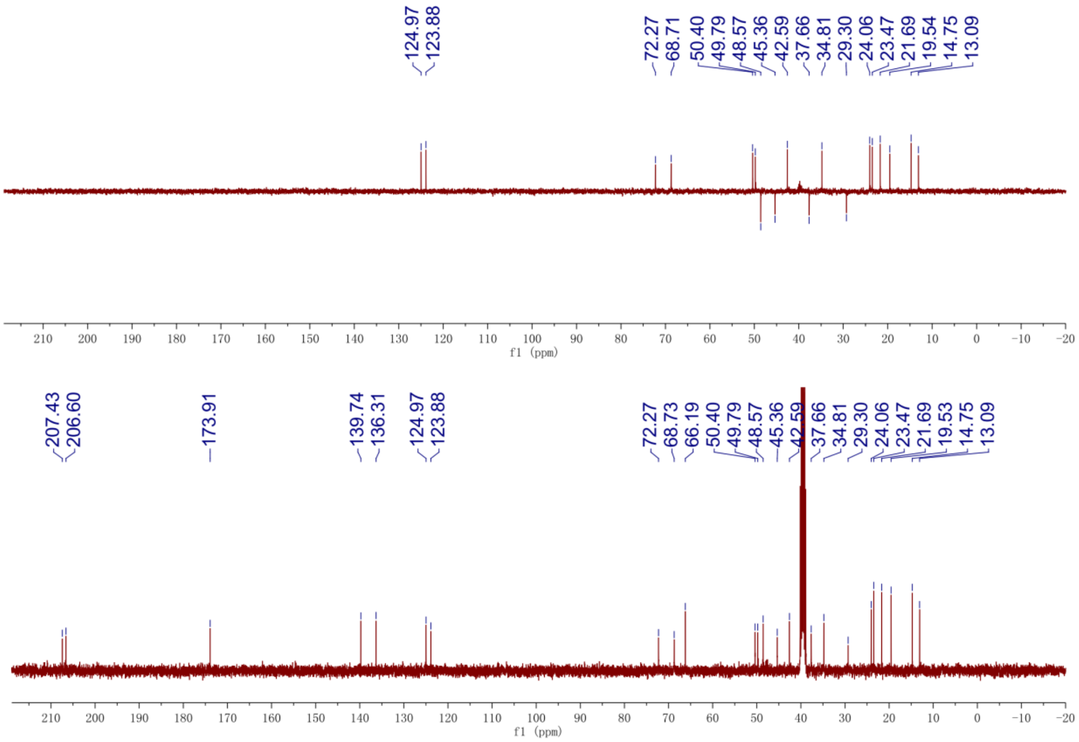

HSQC for compound **15** (in DMSO, 400 MHz)

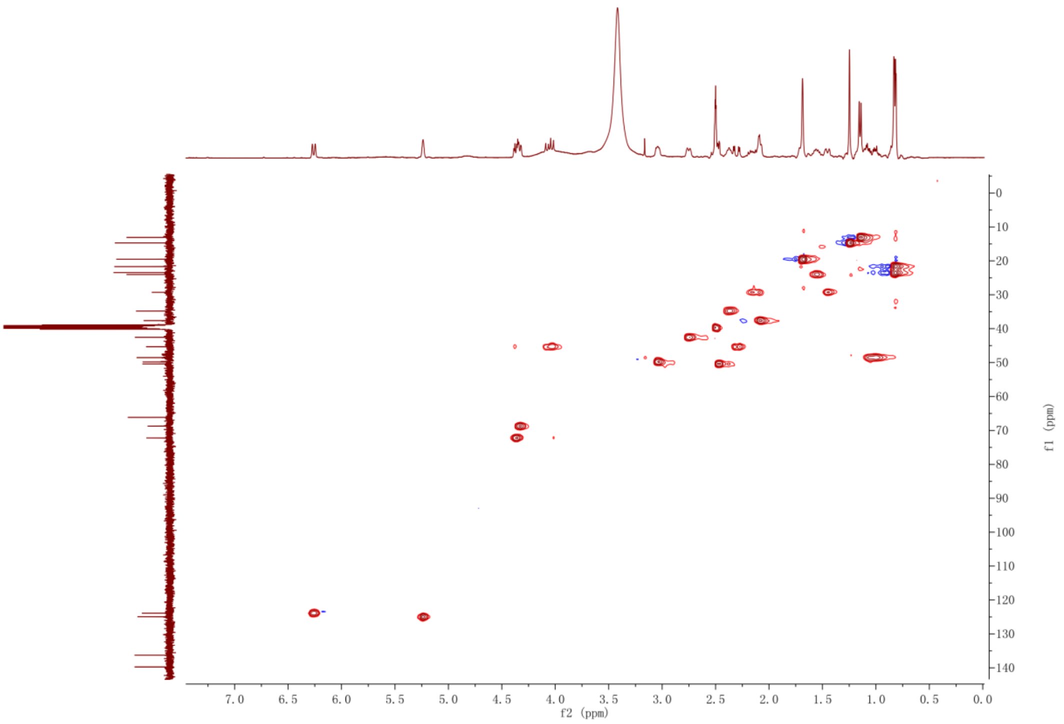

HMBC for compound **15** (in DMSO, 400 MHz)

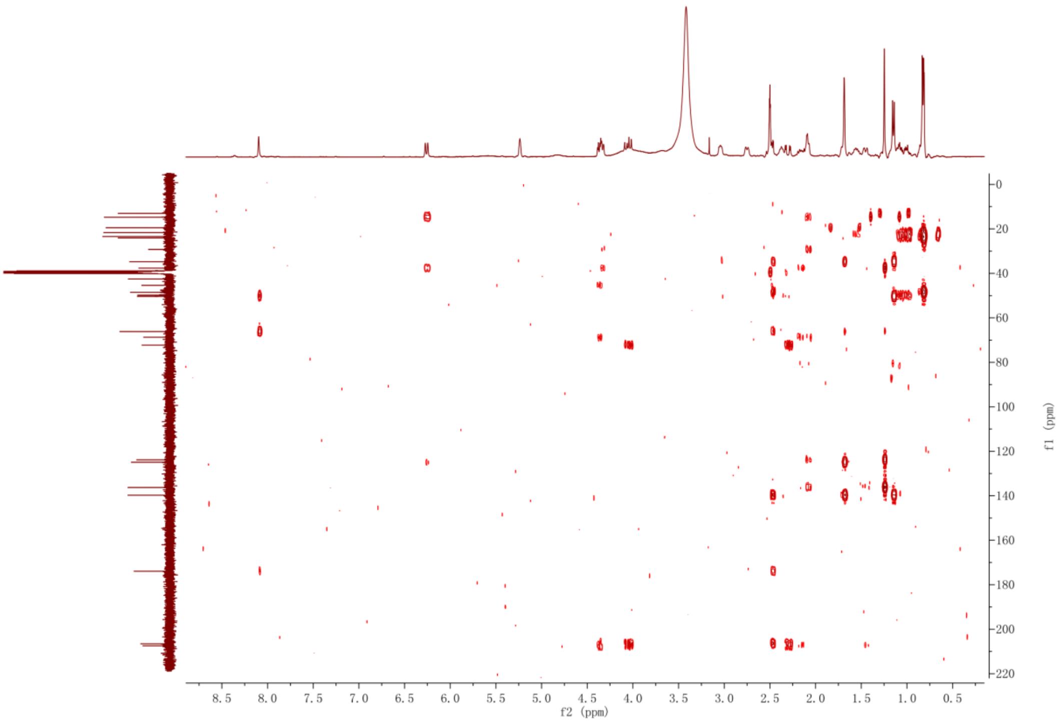

^1H–^1H COSY for compound **15** (in DMSO, 400 MHz)

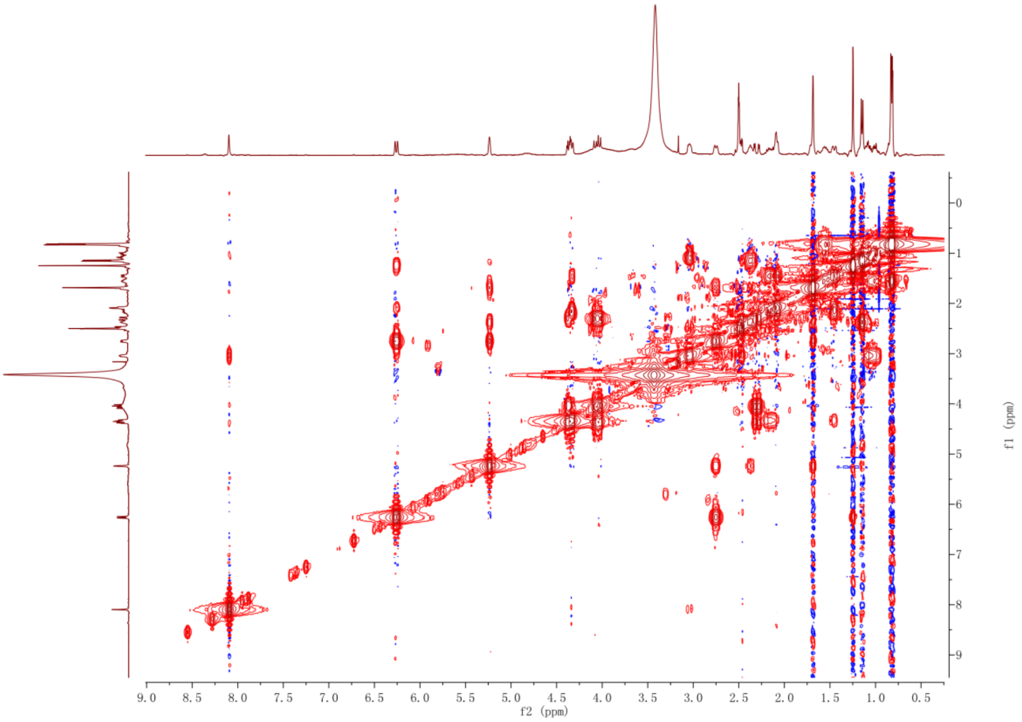

NOESY for compound **15** (in DMSO, 400 MHz)

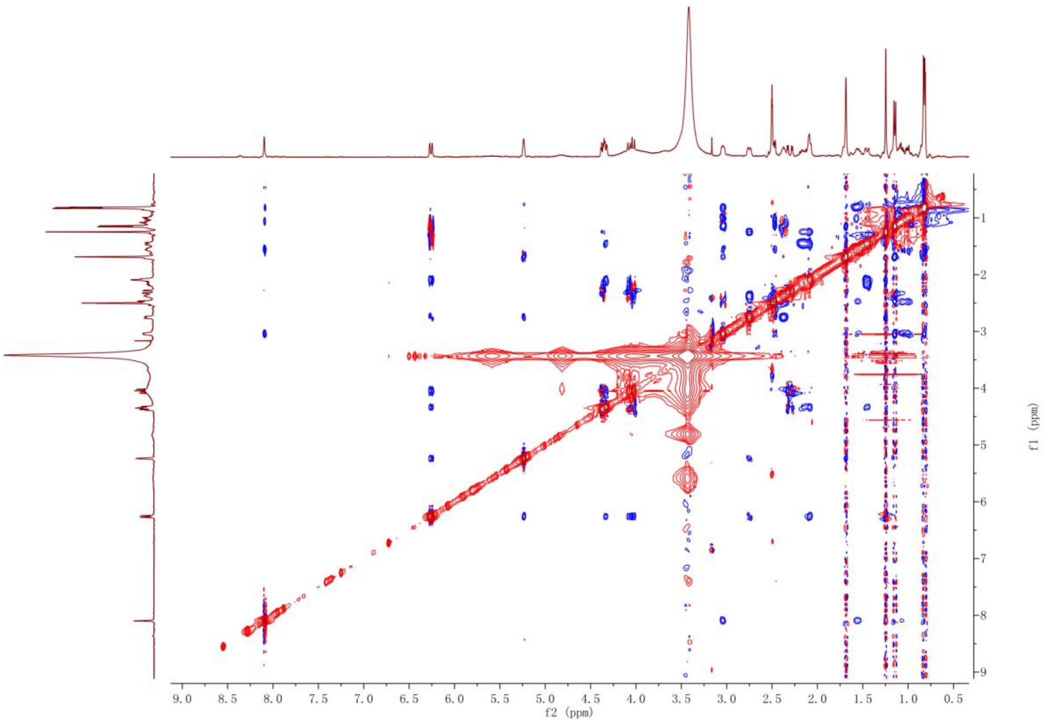

Supplement: Supplementary Information [file srep42434-s1.pdf]
